# Supplementary figures and images for: Epsilon tubulin is an essential determinant of microtubule-based structures in male germ cells (part 1 of 2)
Source: EMBO Rep. 2024 May 21;25(6):14. doi: 10.1038/s44319-024-00159-w (PMC11169422; doi:10.1038/s44319-024-00159-w)

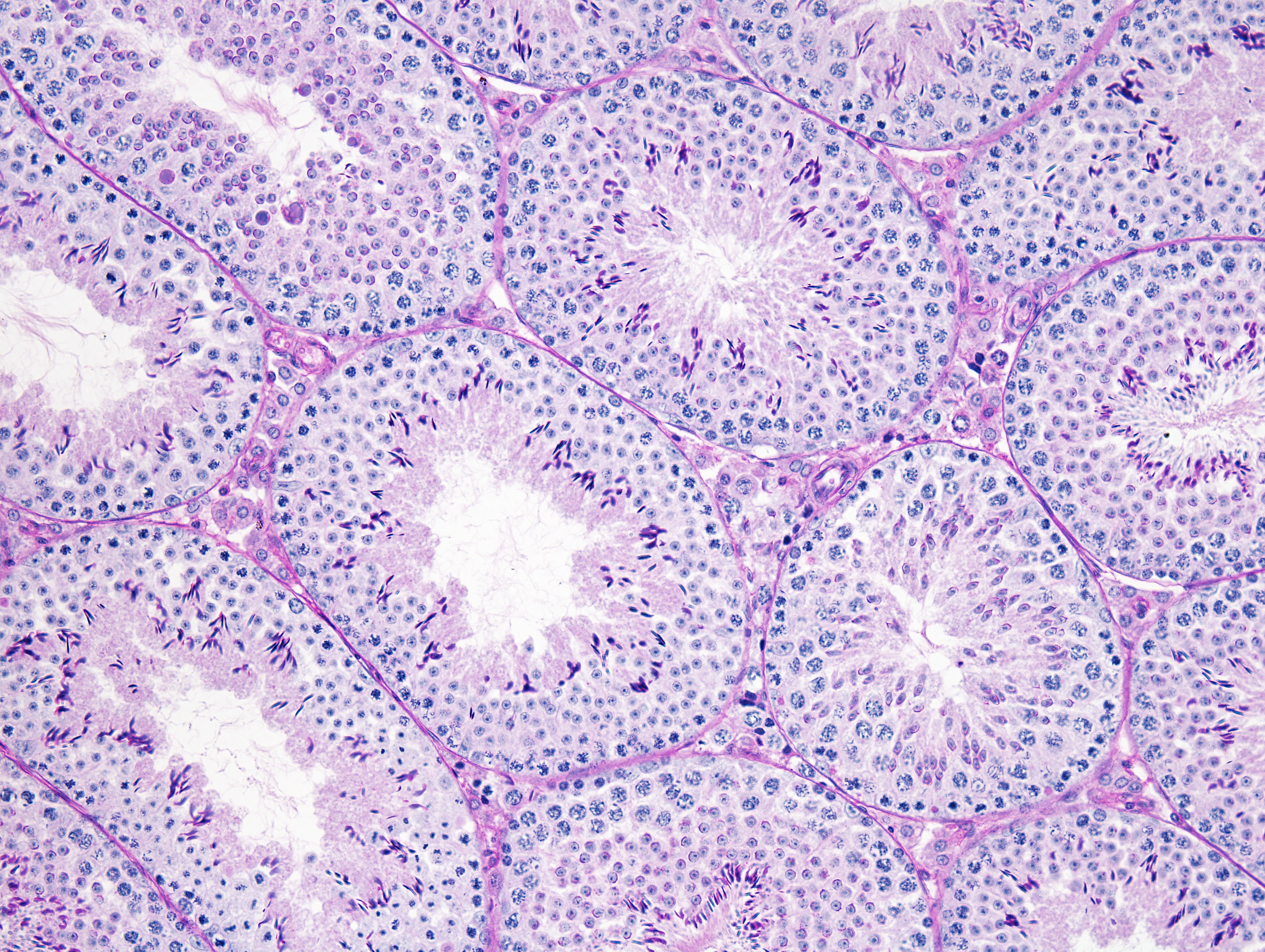

Supplement: Supplementary file 1 — Source data Fig. 1 [file 44319_2024_159_MOESM1_ESM.zip › EMBOR-2023-58207V1_SourceDataForFig1/1F/EMBOR-2023-58207V1_SourceDataForFig1F_Tube1Flox:Flox.tif]

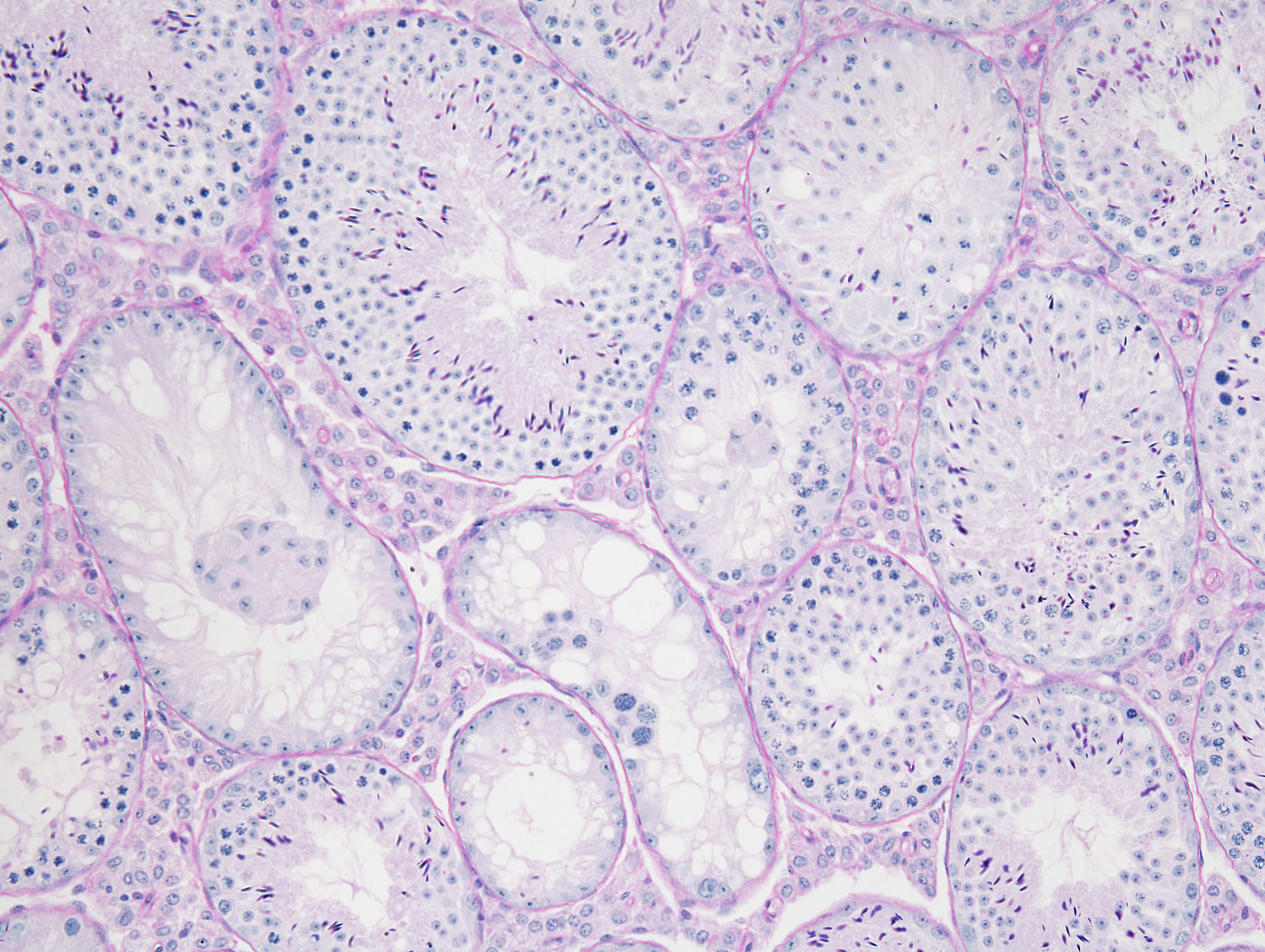

Supplement: Supplementary file 1 — Source data Fig. 1 [file 44319_2024_159_MOESM1_ESM.zip › EMBOR-2023-58207V1_SourceDataForFig1/1F/EMBOR-2023-58207V1_SourceDataForFig1F_Tube1GCKO:GCKO.tif]

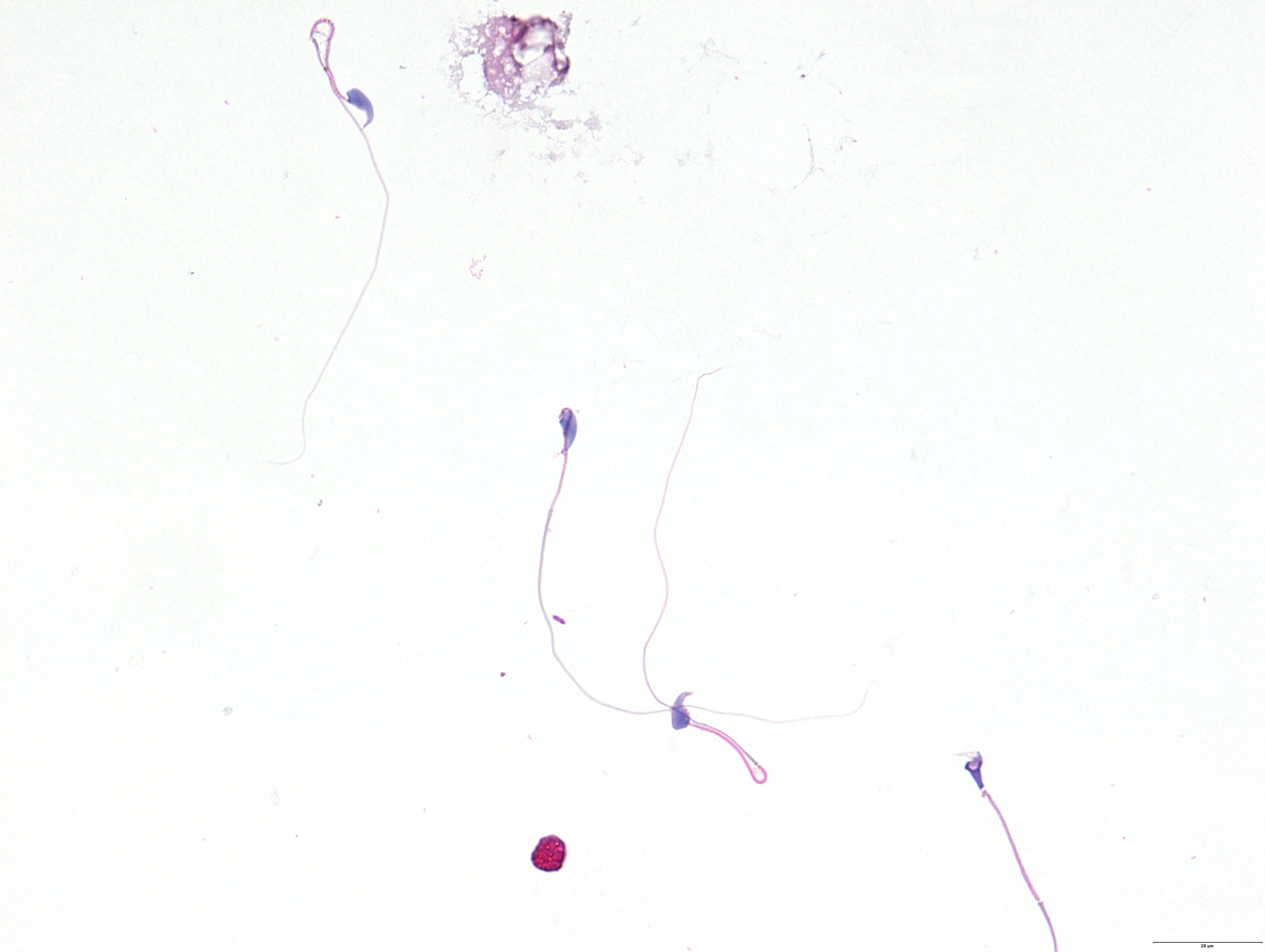

Supplement: Supplementary file 1 — Source data Fig. 1 [file 44319_2024_159_MOESM1_ESM.zip › EMBOR-2023-58207V1_SourceDataForFig1/1I/EMBOR-2023-58207V1_SourceDataForFig1i_narrow.jpg]

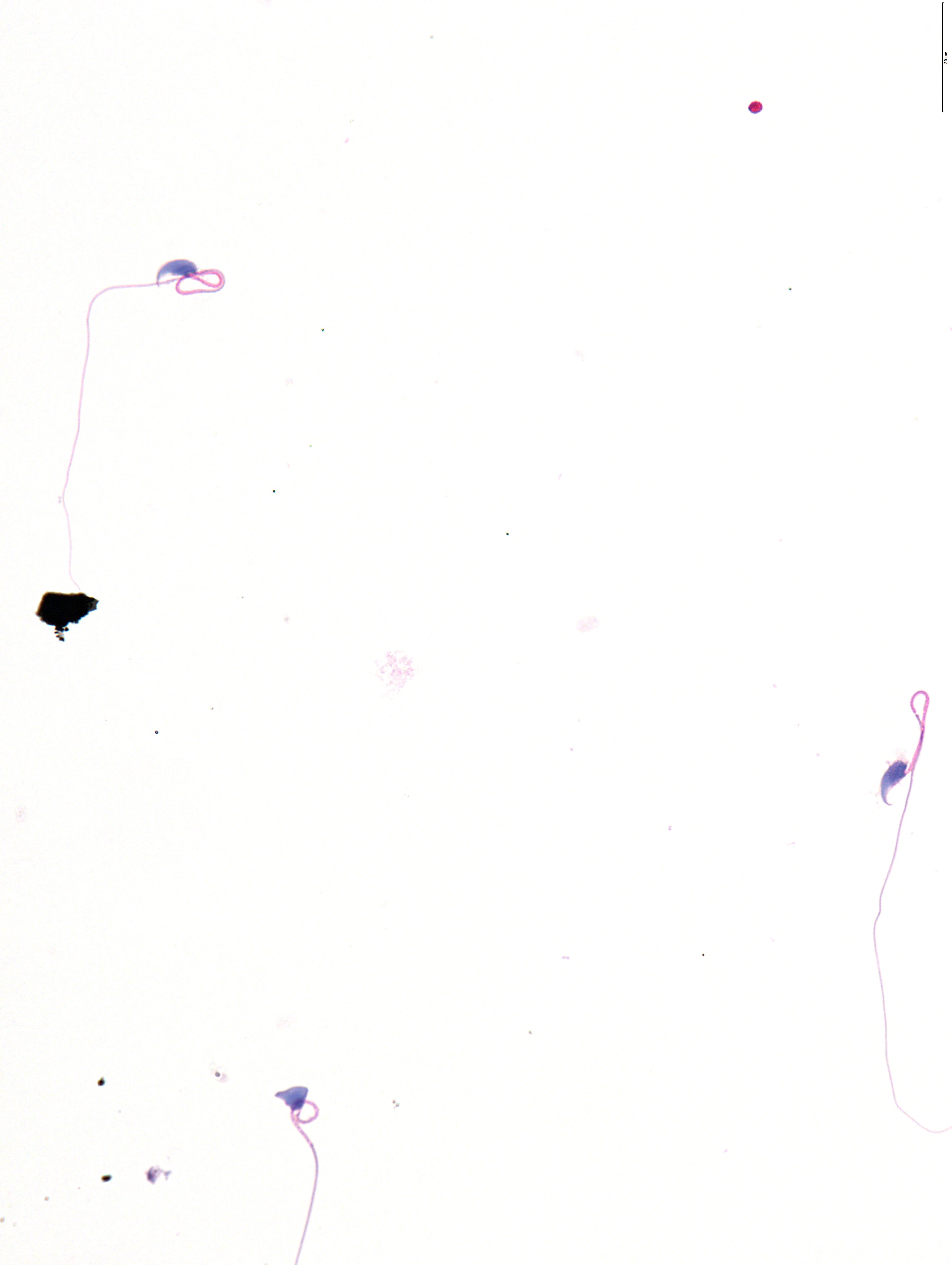

Supplement: Supplementary file 1 — Source data Fig. 1 [file 44319_2024_159_MOESM1_ESM.zip › EMBOR-2023-58207V1_SourceDataForFig1/1I/EMBOR-2023-58207V1_SourceDataForFig1i_wide.jpg]

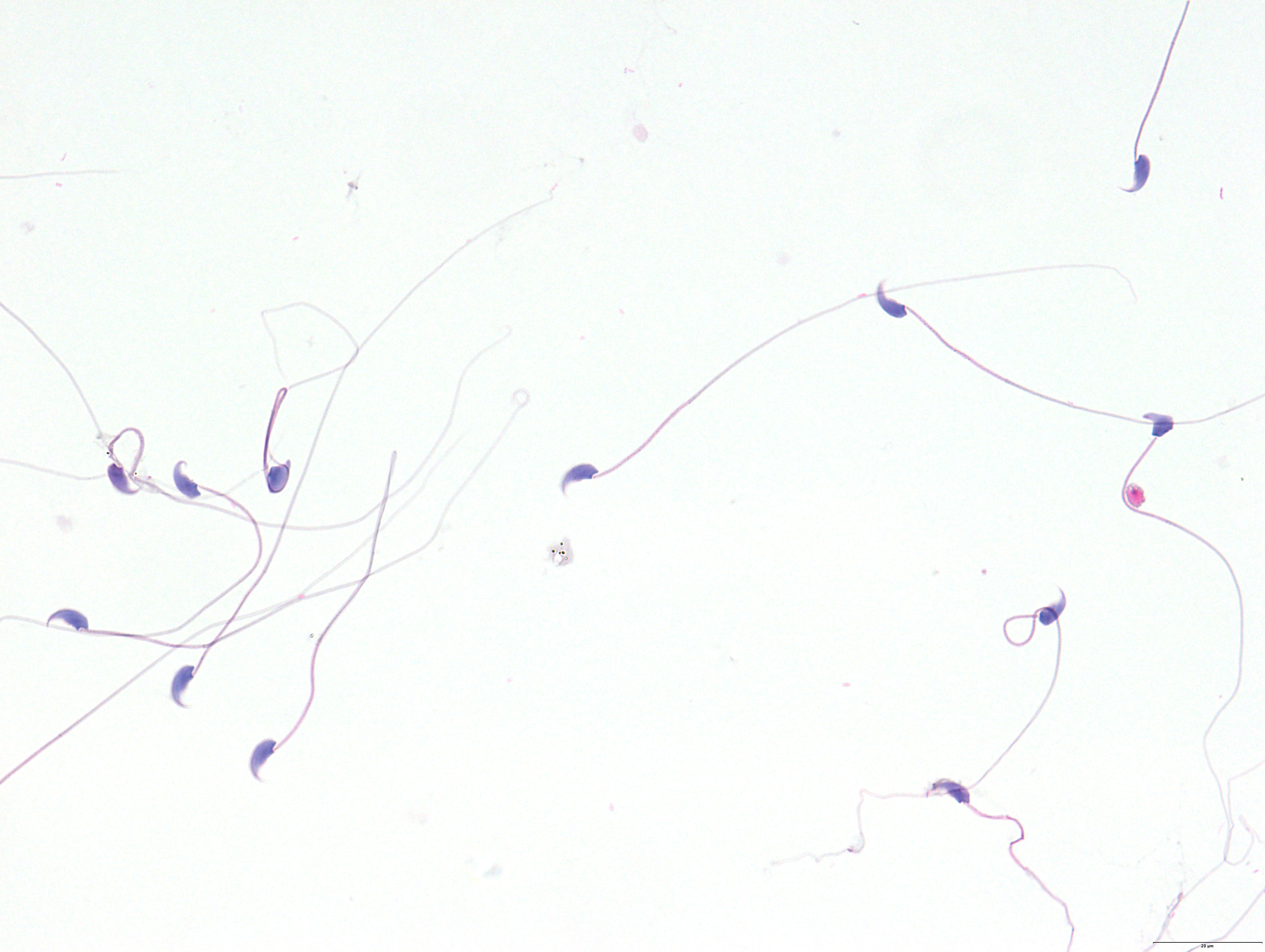

Supplement: Supplementary file 1 — Source data Fig. 1 [file 44319_2024_159_MOESM1_ESM.zip › EMBOR-2023-58207V1_SourceDataForFig1/1I/EMBOR-2023-58207V1_SourceDataForFig1i_normal.jpg]

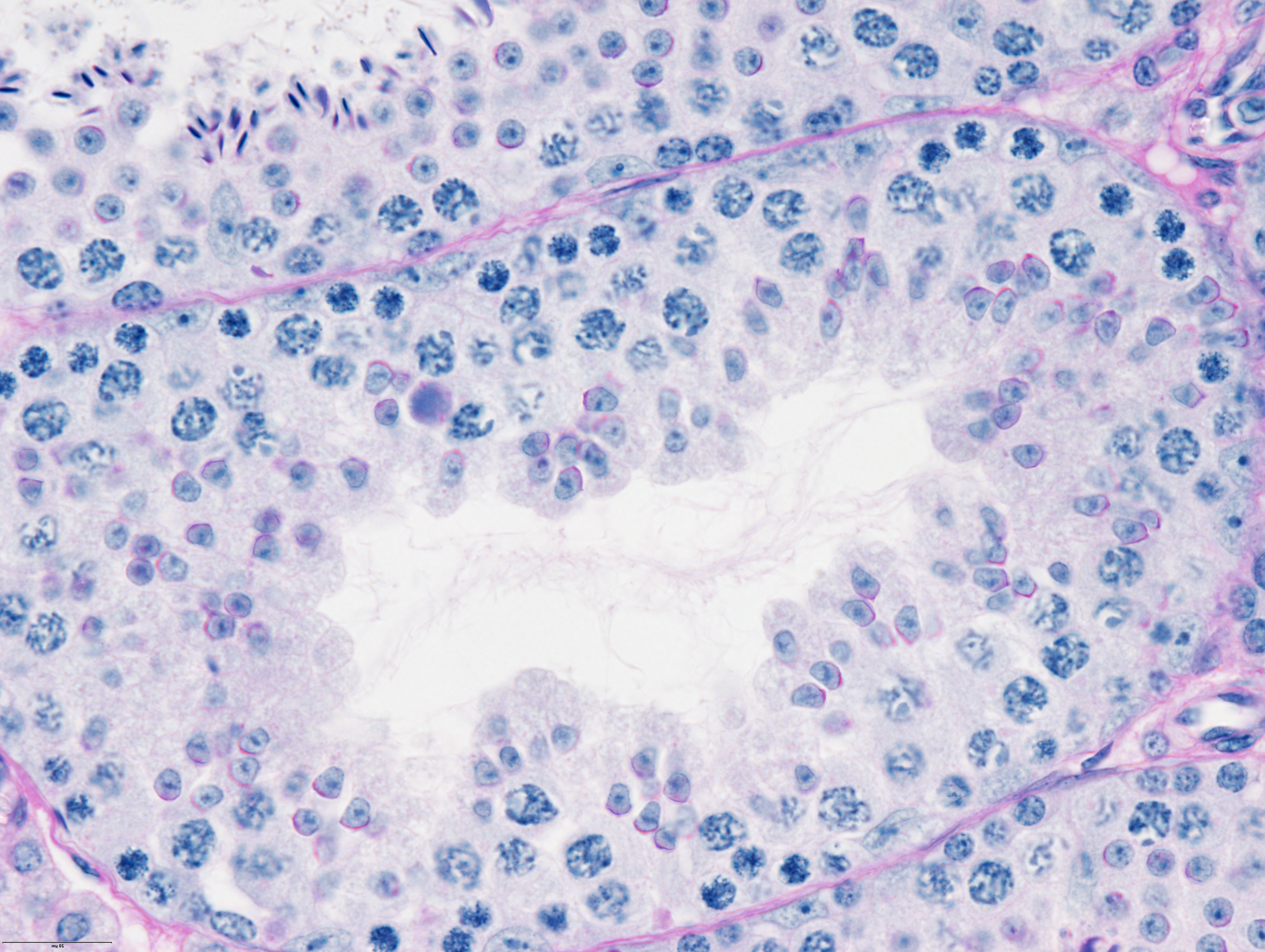

Supplement: Supplementary file 1 — Source data Fig. 1 [file 44319_2024_159_MOESM1_ESM.zip › EMBOR-2023-58207V1_SourceDataForFig1/1E/EMBOR-2023-58207V1_SourceDataForFig1E_Tube1Flox:Flox.tif]

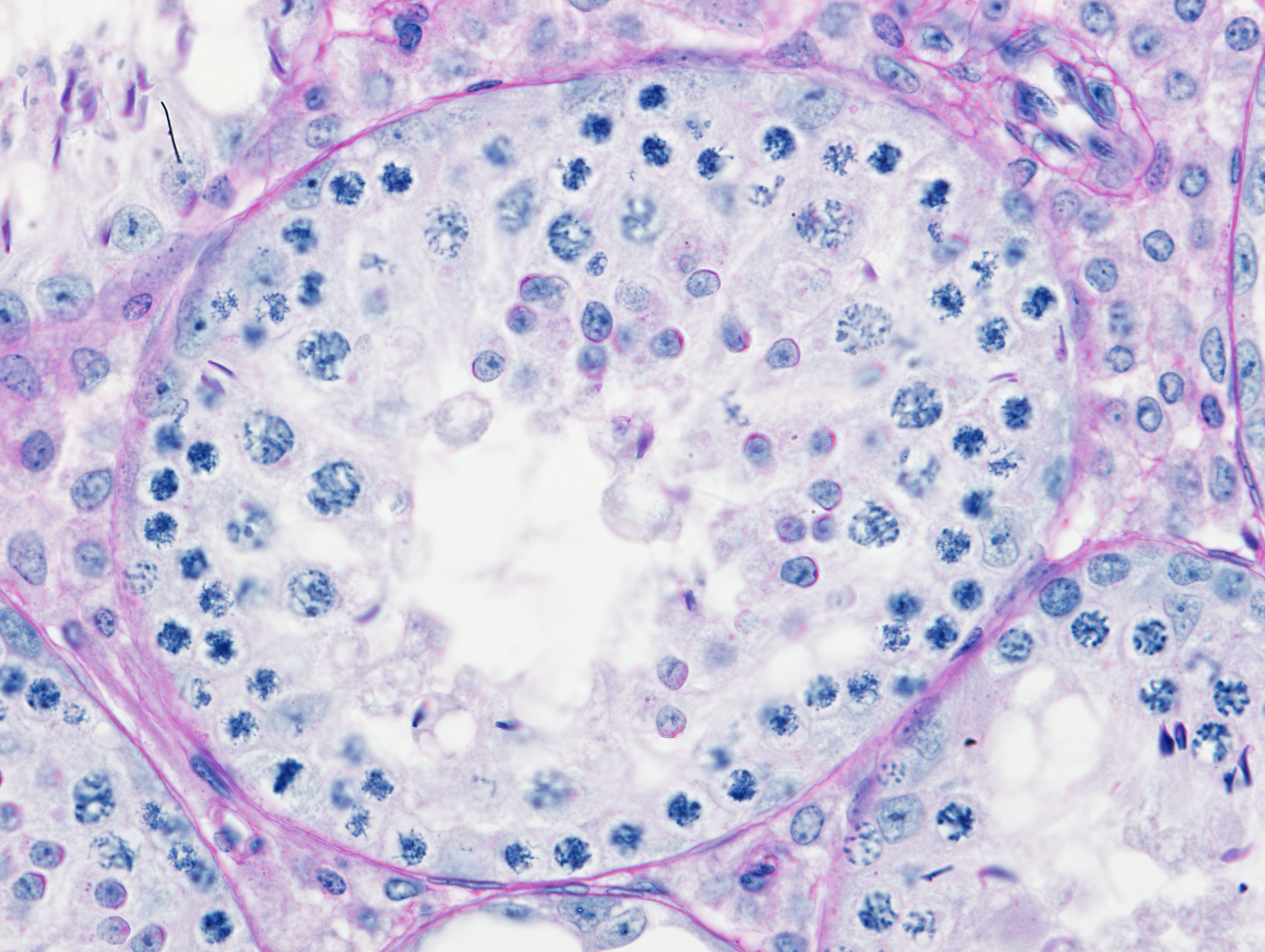

Supplement: Supplementary file 1 — Source data Fig. 1 [file 44319_2024_159_MOESM1_ESM.zip › EMBOR-2023-58207V1_SourceDataForFig1/1E/EMBOR-2023-58207V1_SourceDataForFig1E_Tube1GCKO:GCKO.tif]

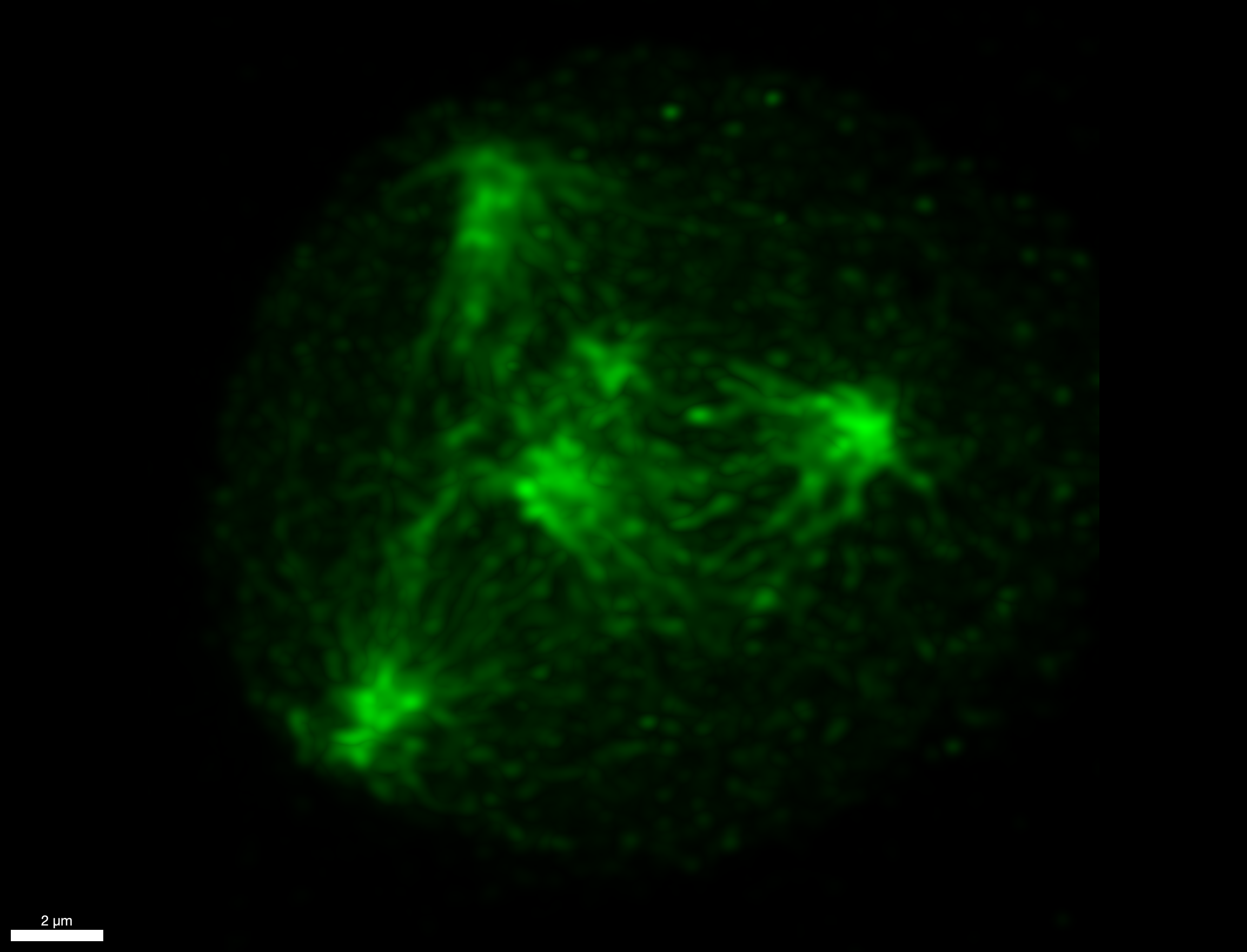

Supplement: Supplementary file 2 — Source data Fig. 2 [file 44319_2024_159_MOESM2_ESM.zip › EMBOR-2023-58207V1_SourceDataForFig2/2G/EMBOR-2023-58207V1_SourceDataForFig2G_beta tubulin.tif]

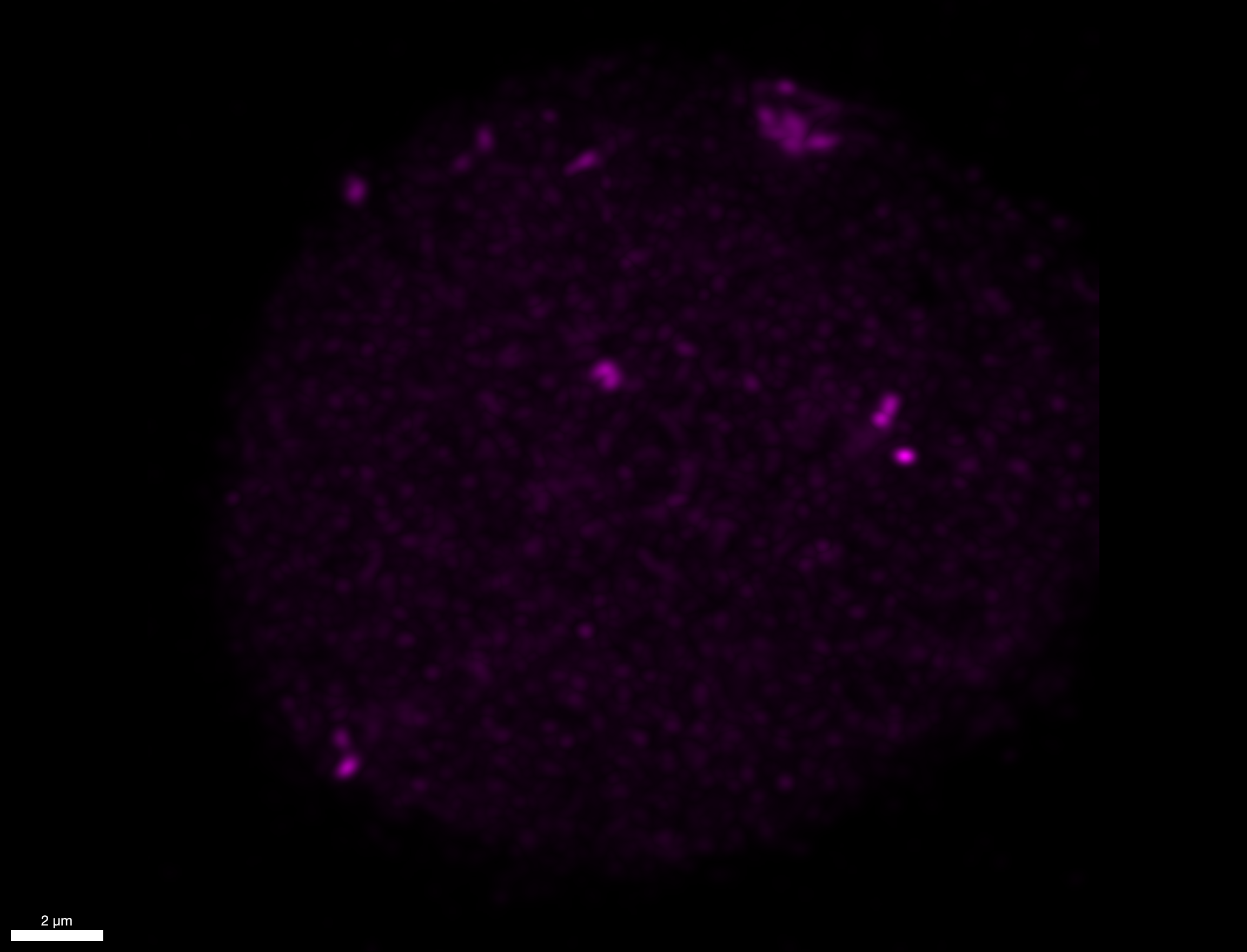

Supplement: Supplementary file 2 — Source data Fig. 2 [file 44319_2024_159_MOESM2_ESM.zip › EMBOR-2023-58207V1_SourceDataForFig2/2G/EMBOR-2023-58207V1_SourceDataForFig2G_centrin.tif]

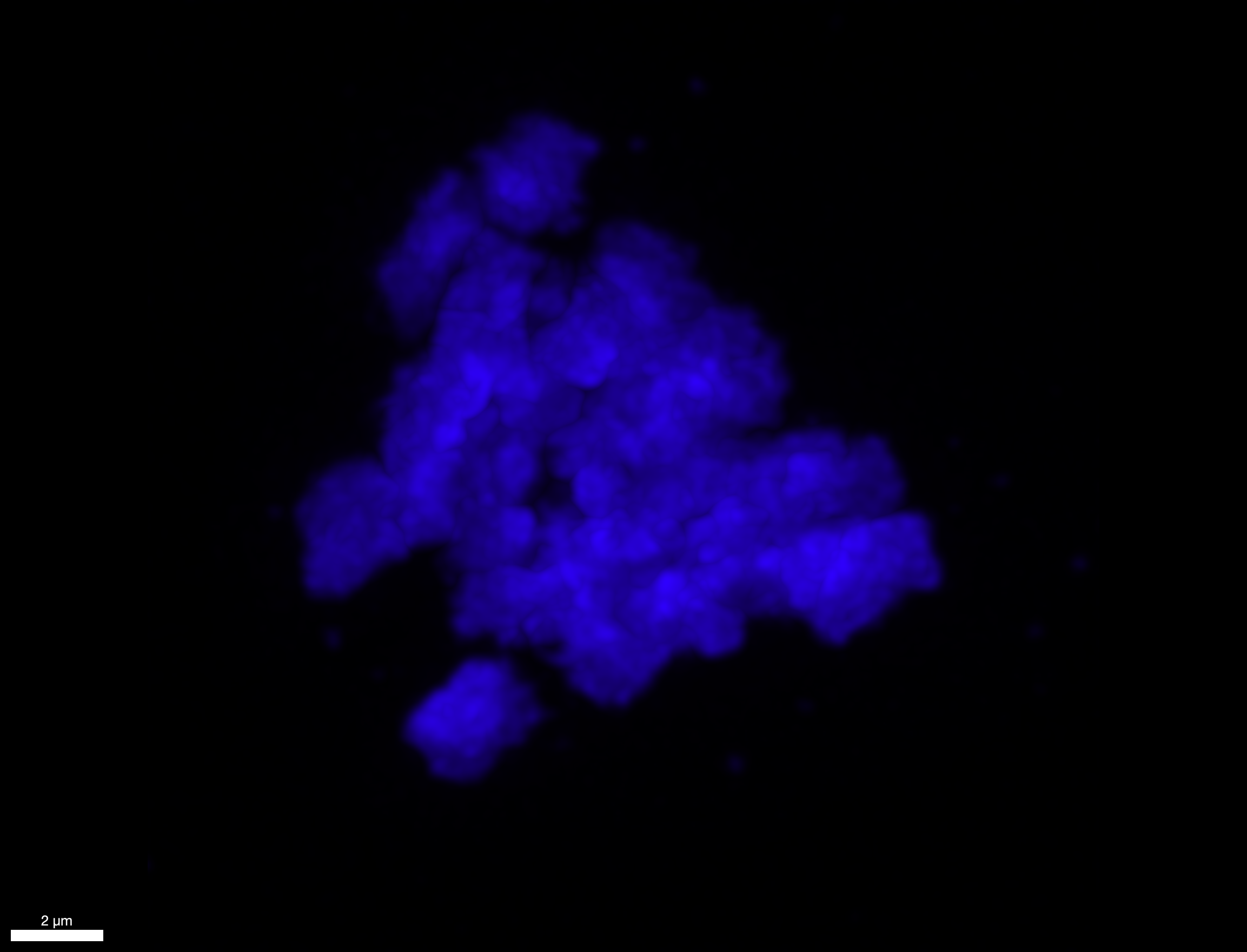

Supplement: Supplementary file 2 — Source data Fig. 2 [file 44319_2024_159_MOESM2_ESM.zip › EMBOR-2023-58207V1_SourceDataForFig2/2G/EMBOR-2023-58207V1_SourceDataForFig2G_DAPI.tif]

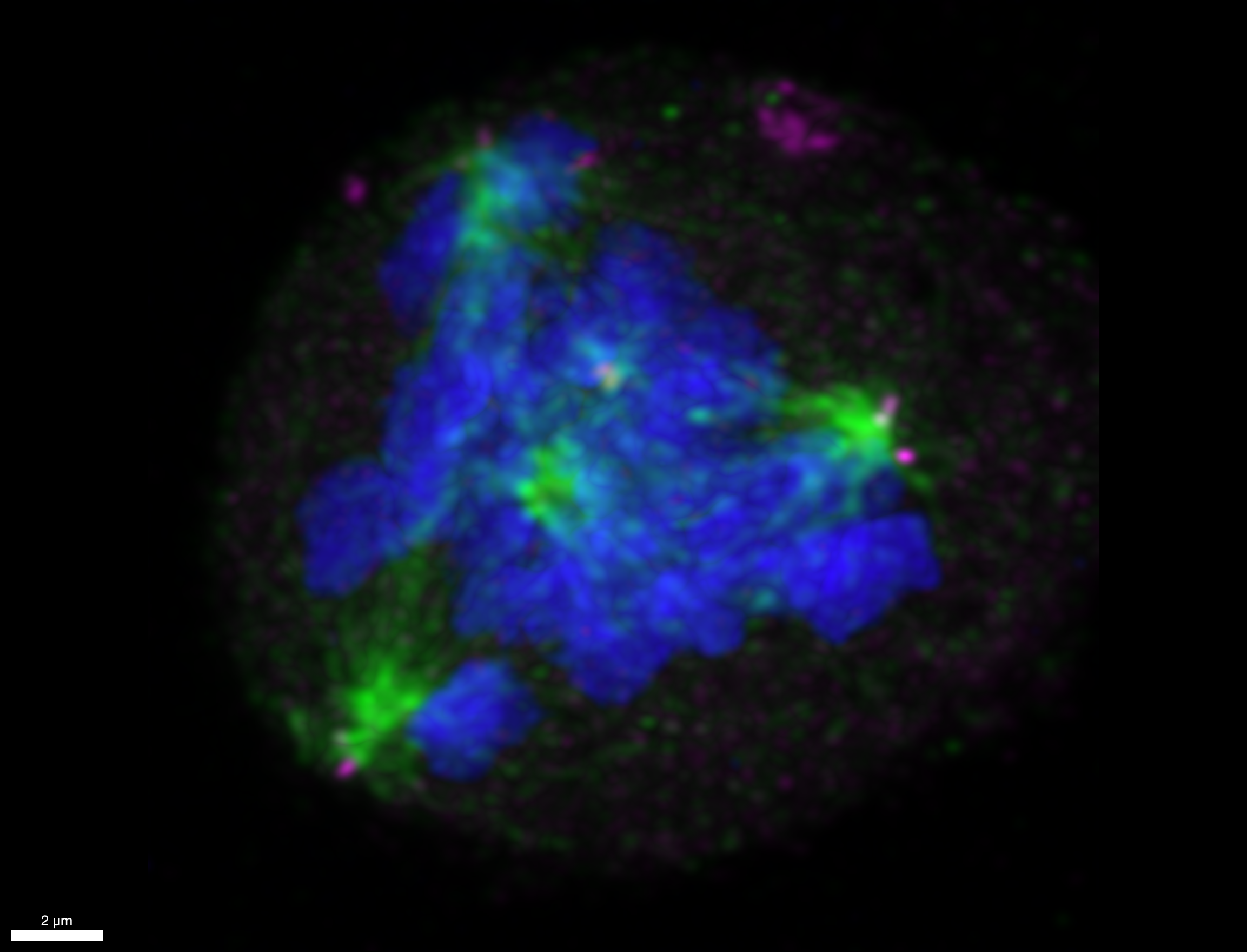

Supplement: Supplementary file 2 — Source data Fig. 2 [file 44319_2024_159_MOESM2_ESM.zip › EMBOR-2023-58207V1_SourceDataForFig2/2G/EMBOR-2023-58207V1_SourceDataForFig2G_merge.tif]

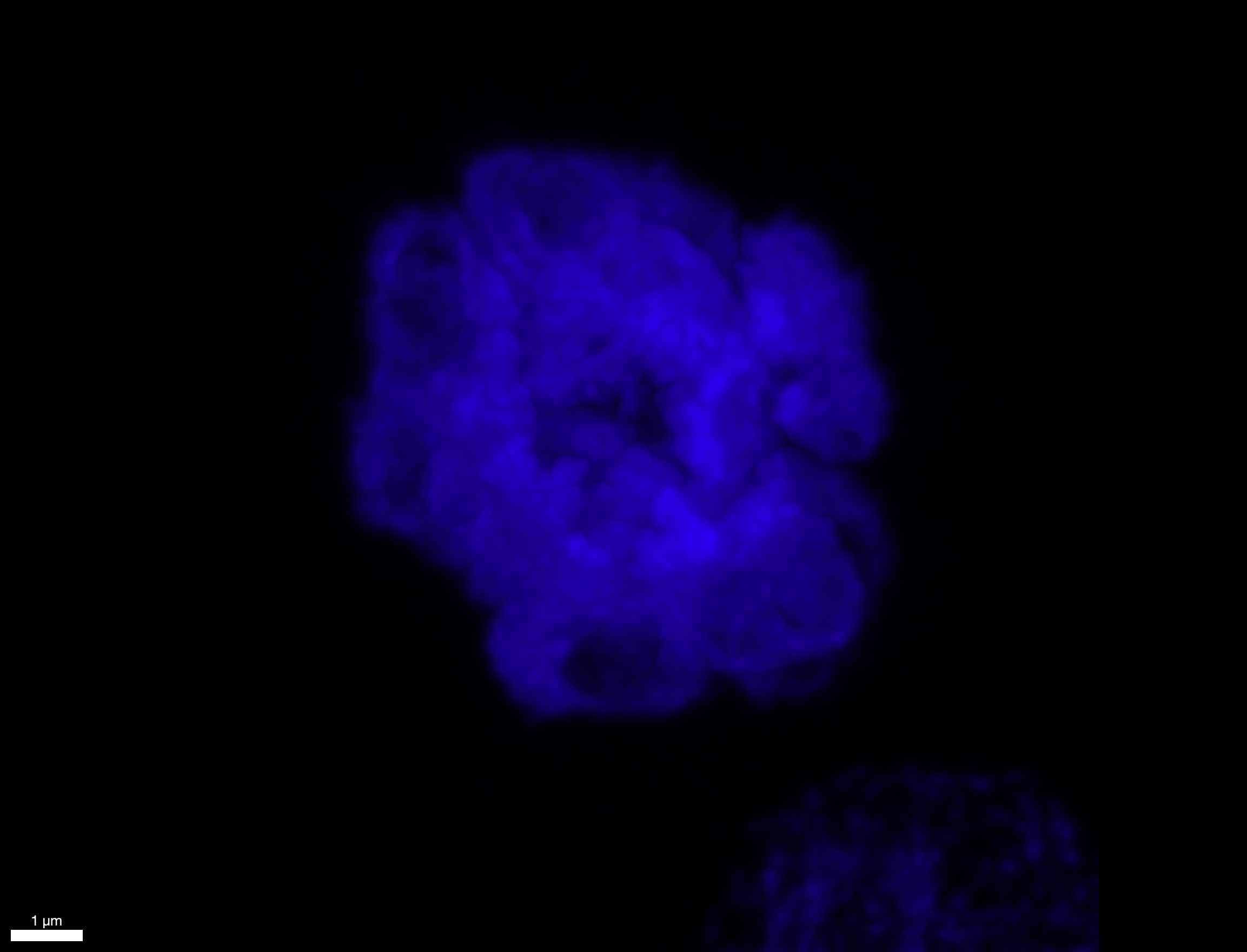

Supplement: Supplementary file 2 — Source data Fig. 2 [file 44319_2024_159_MOESM2_ESM.zip › EMBOR-2023-58207V1_SourceDataForFig2/2H/EMBOR-2023-58207V1_SourceDataForFig2H_DAPI.tif]

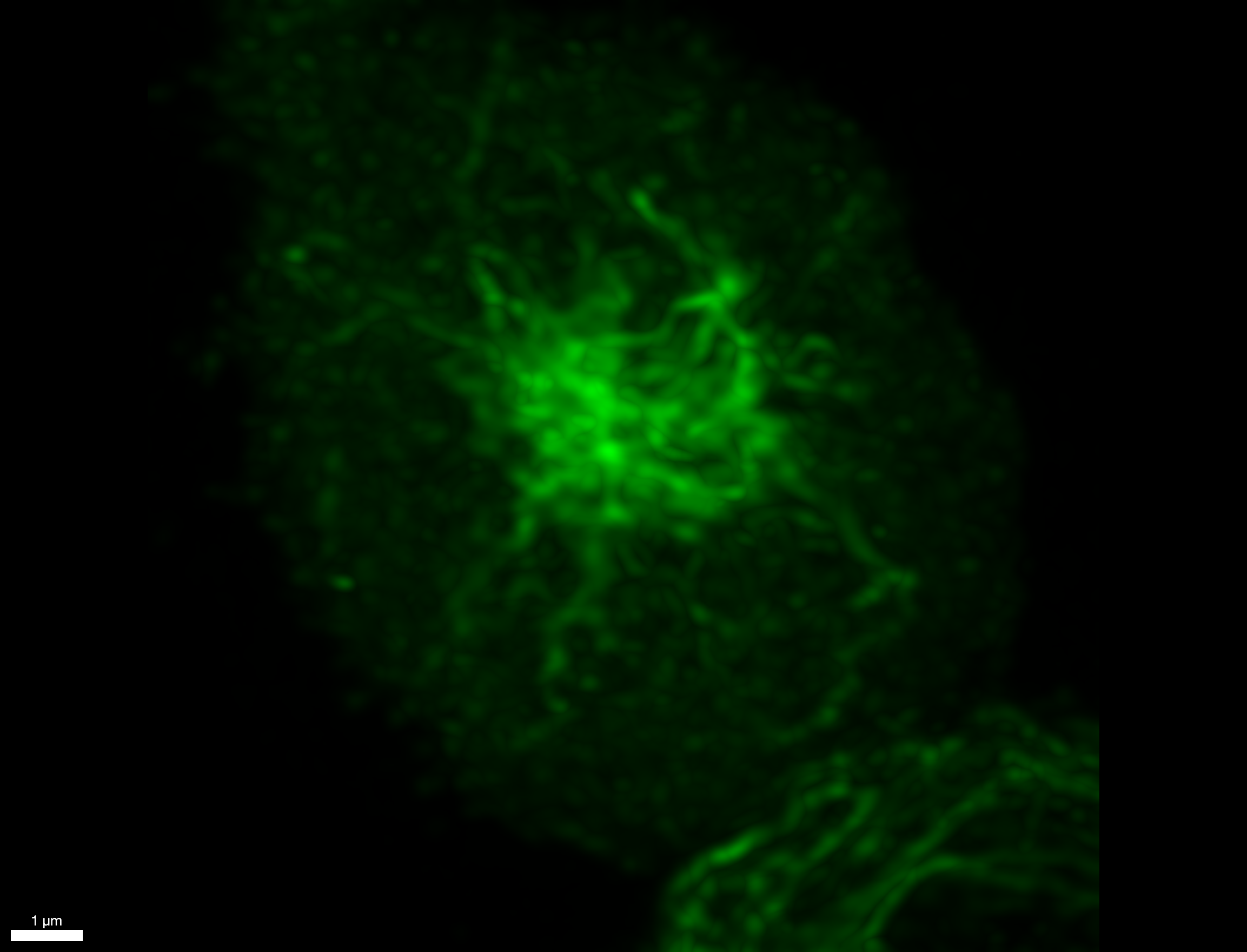

Supplement: Supplementary file 2 — Source data Fig. 2 [file 44319_2024_159_MOESM2_ESM.zip › EMBOR-2023-58207V1_SourceDataForFig2/2H/EMBOR-2023-58207V1_SourceDataForFig2H_beta tubulin.tif]

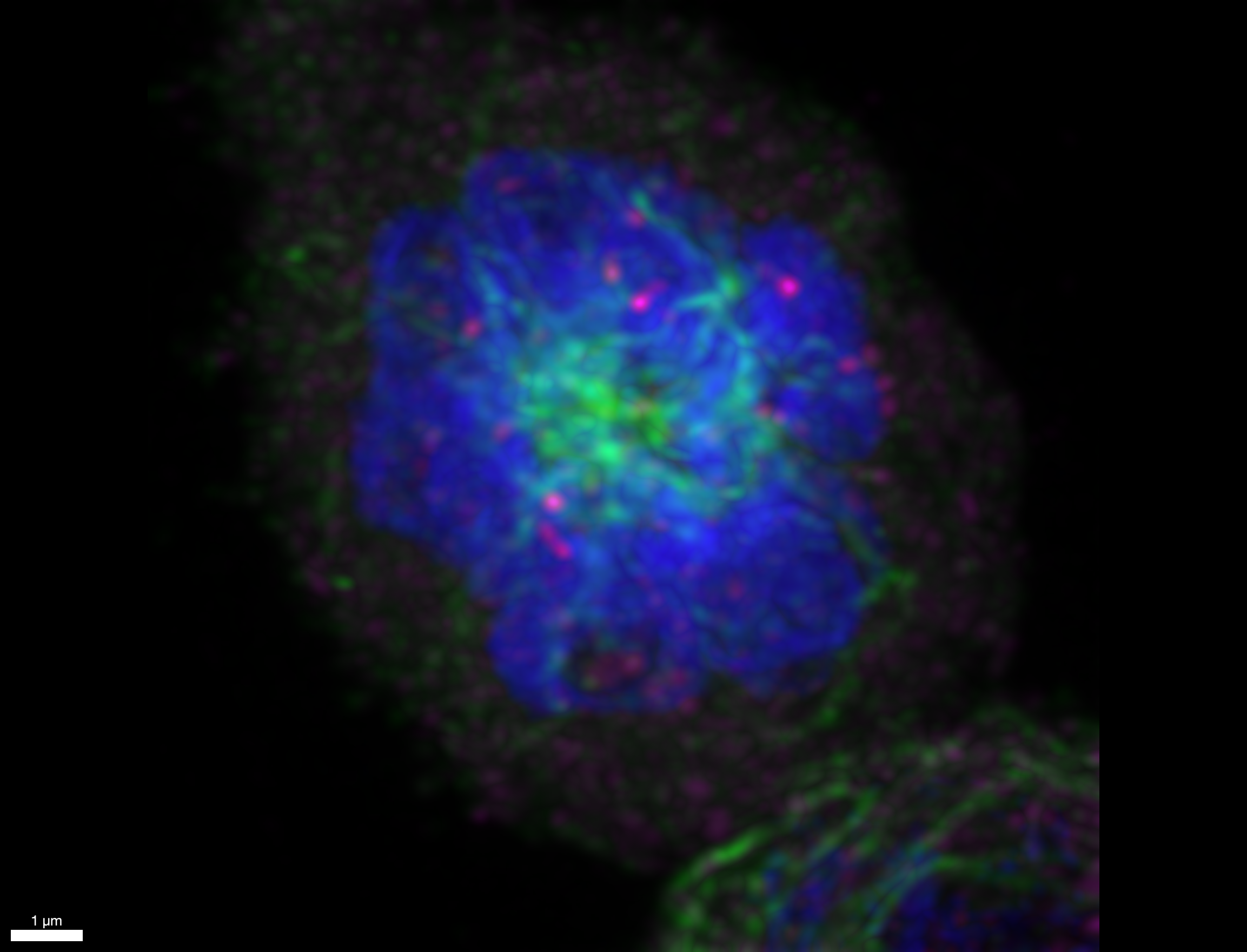

Supplement: Supplementary file 2 — Source data Fig. 2 [file 44319_2024_159_MOESM2_ESM.zip › EMBOR-2023-58207V1_SourceDataForFig2/2H/EMBOR-2023-58207V1_SourceDataForFig2H_merge.tif]

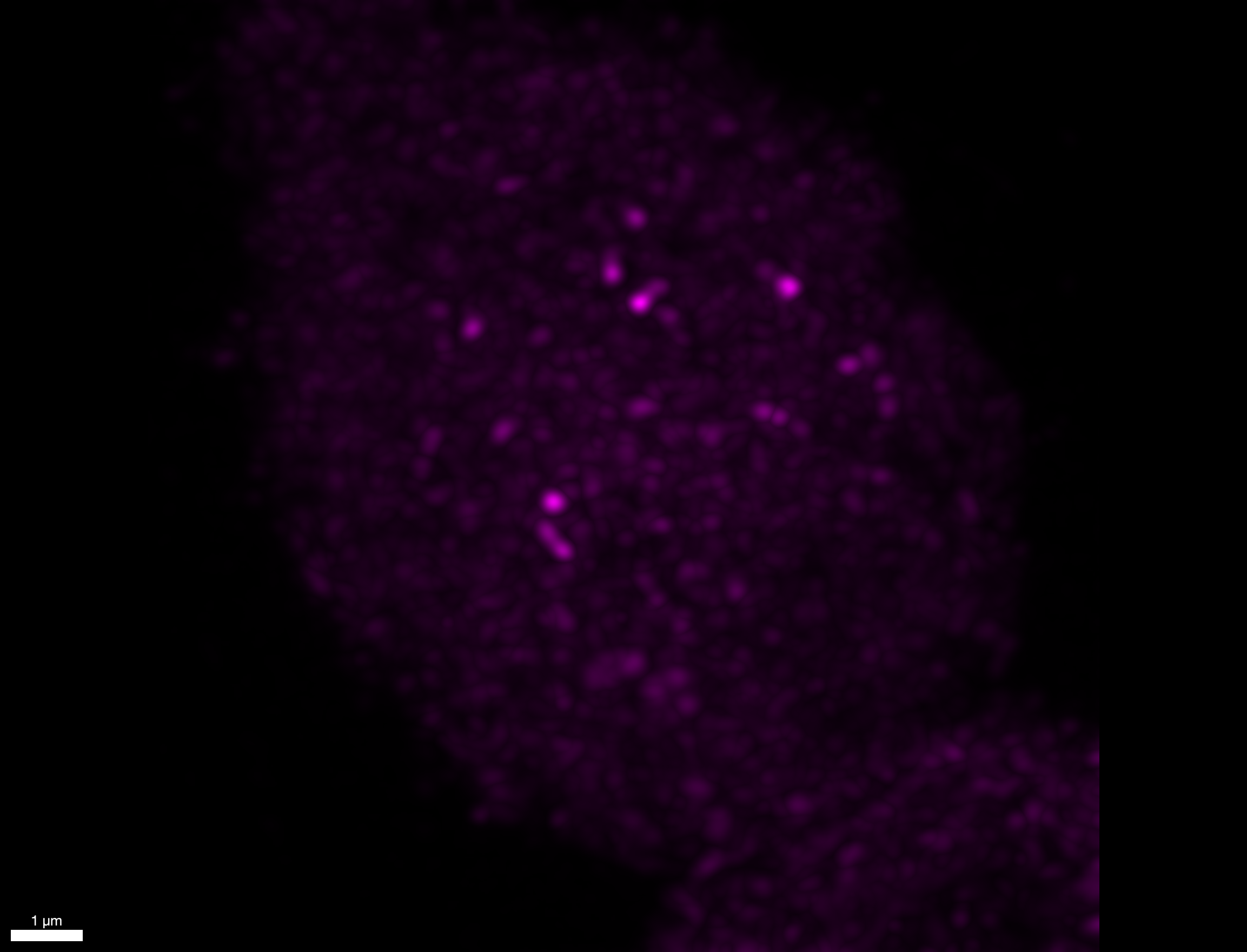

Supplement: Supplementary file 2 — Source data Fig. 2 [file 44319_2024_159_MOESM2_ESM.zip › EMBOR-2023-58207V1_SourceDataForFig2/2H/EMBOR-2023-58207V1_SourceDataForFig2H_centrin.tif]

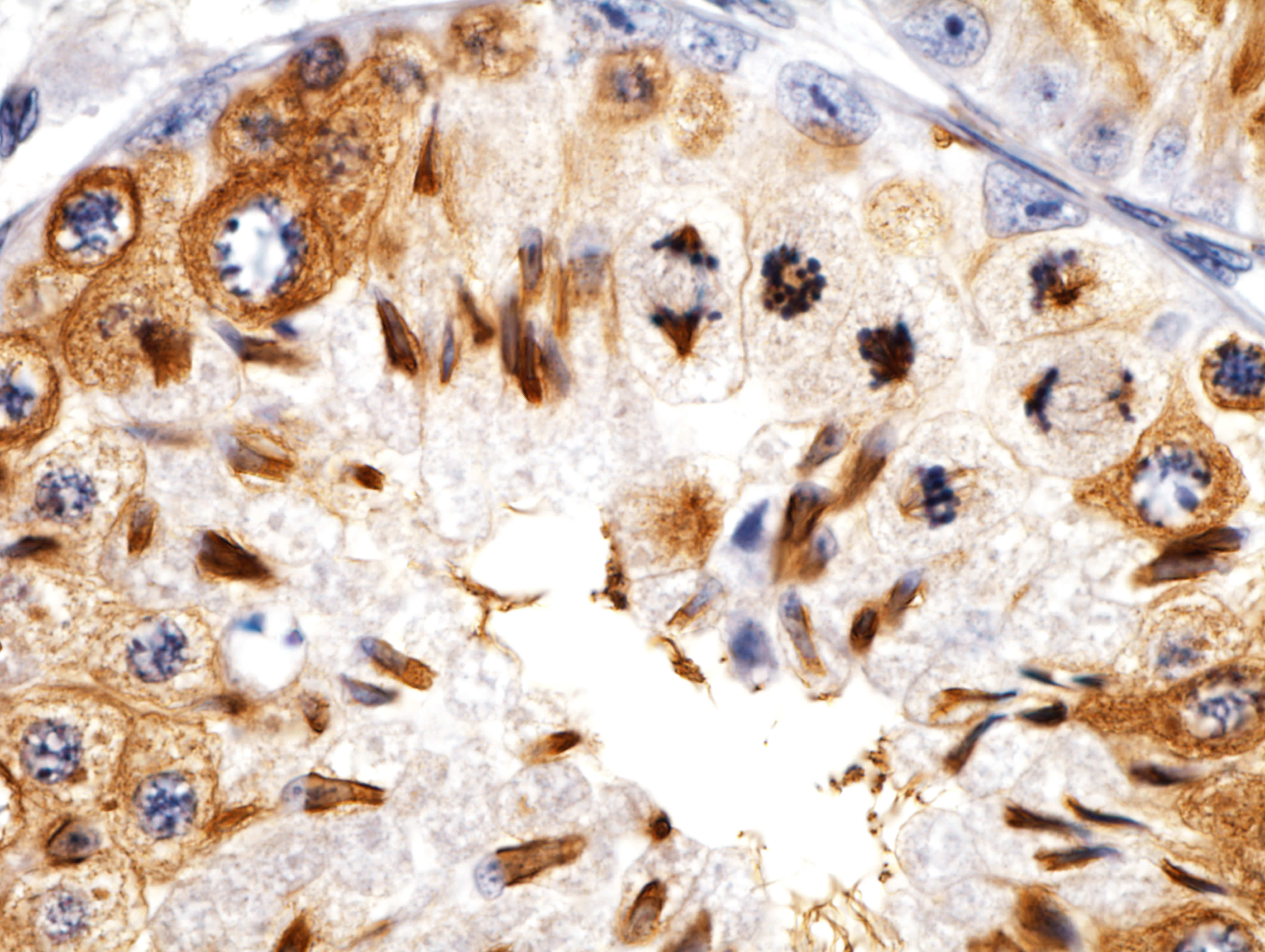

Supplement: Supplementary file 2 — Source data Fig. 2 [file 44319_2024_159_MOESM2_ESM.zip › EMBOR-2023-58207V1_SourceDataForFig2/2A/EMBOR-2023-58207V1_SourceDataForFig2a_ii_v_vi.tif]

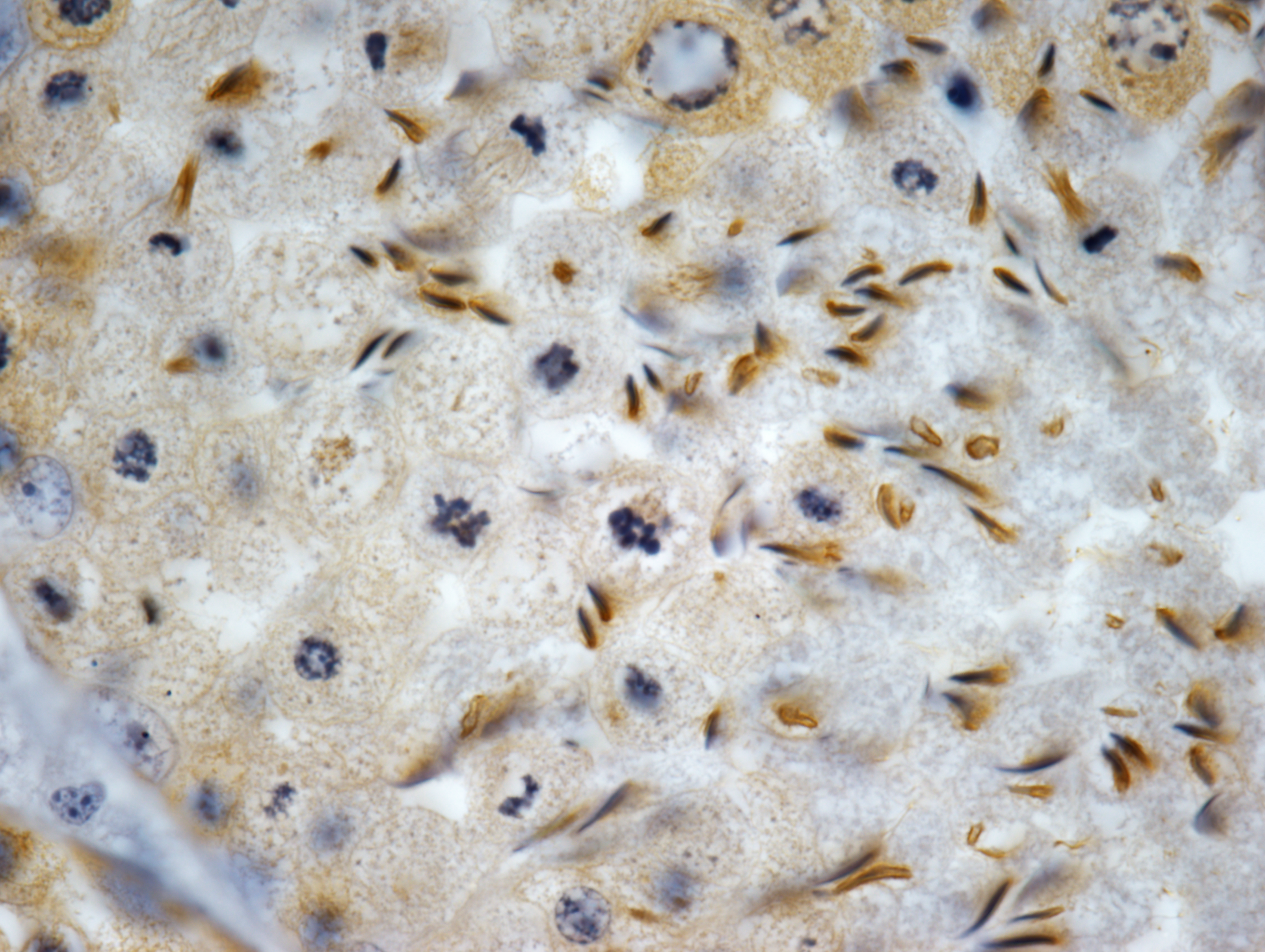

Supplement: Supplementary file 2 — Source data Fig. 2 [file 44319_2024_159_MOESM2_ESM.zip › EMBOR-2023-58207V1_SourceDataForFig2/2A/EMBOR-2023-58207V1_SourceDataForFig2a_iv.tif]

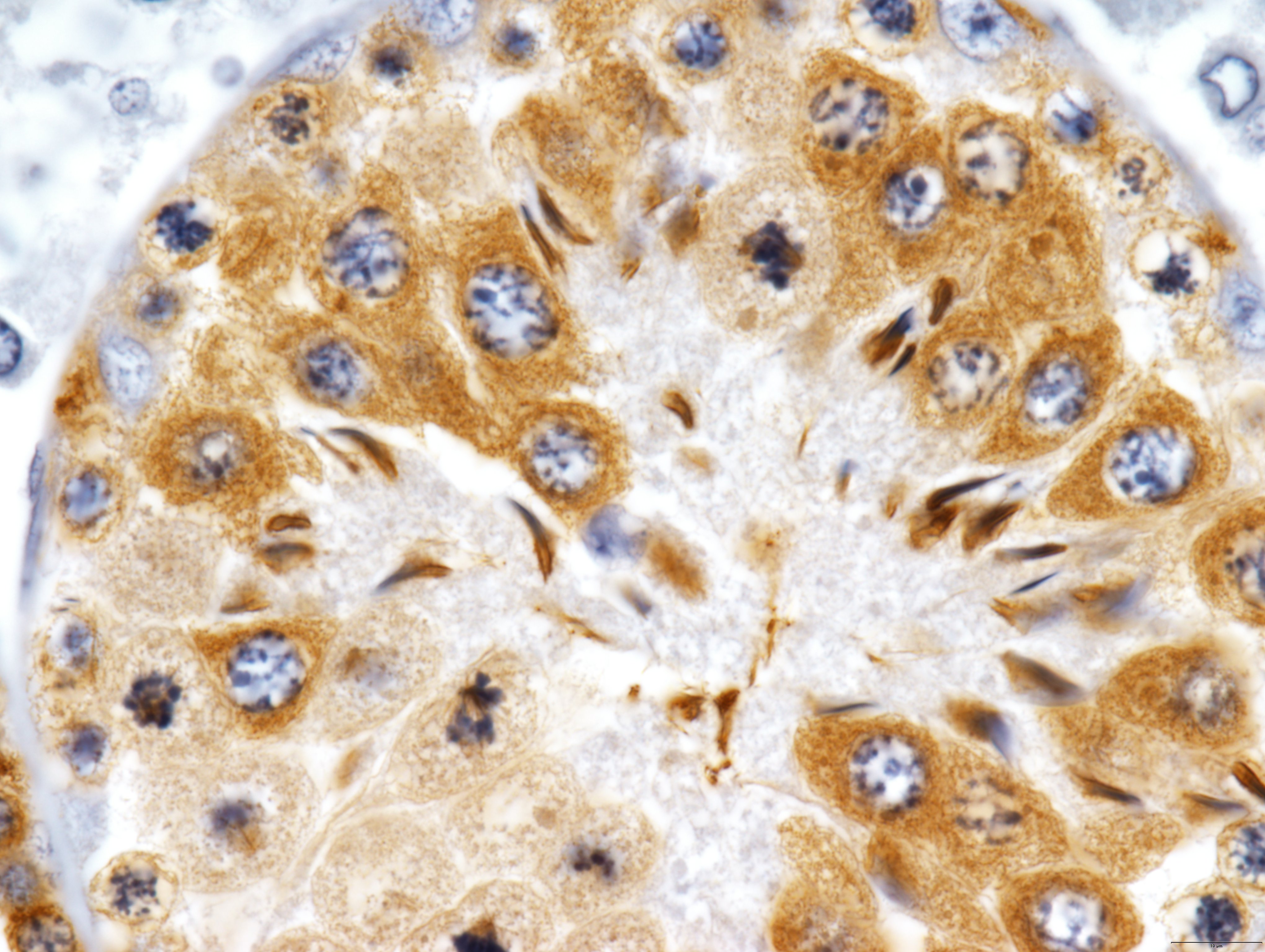

Supplement: Supplementary file 2 — Source data Fig. 2 [file 44319_2024_159_MOESM2_ESM.zip › EMBOR-2023-58207V1_SourceDataForFig2/2A/EMBOR-2023-58207V1_SourceDataForFig2a_vii.jpg]

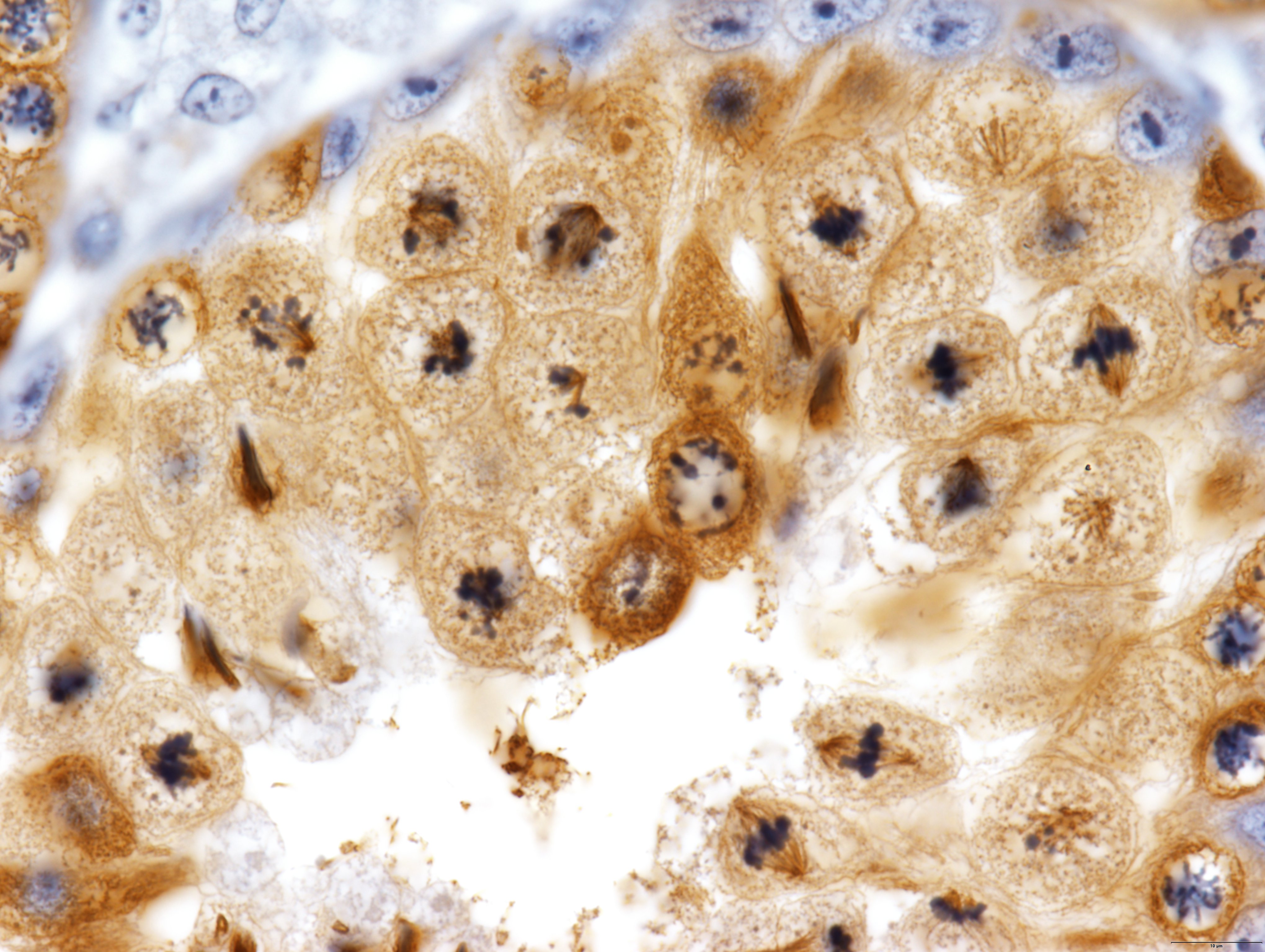

Supplement: Supplementary file 2 — Source data Fig. 2 [file 44319_2024_159_MOESM2_ESM.zip › EMBOR-2023-58207V1_SourceDataForFig2/2A/EMBOR-2023-58207V1_SourceDataForFig2a_viii.jpg]

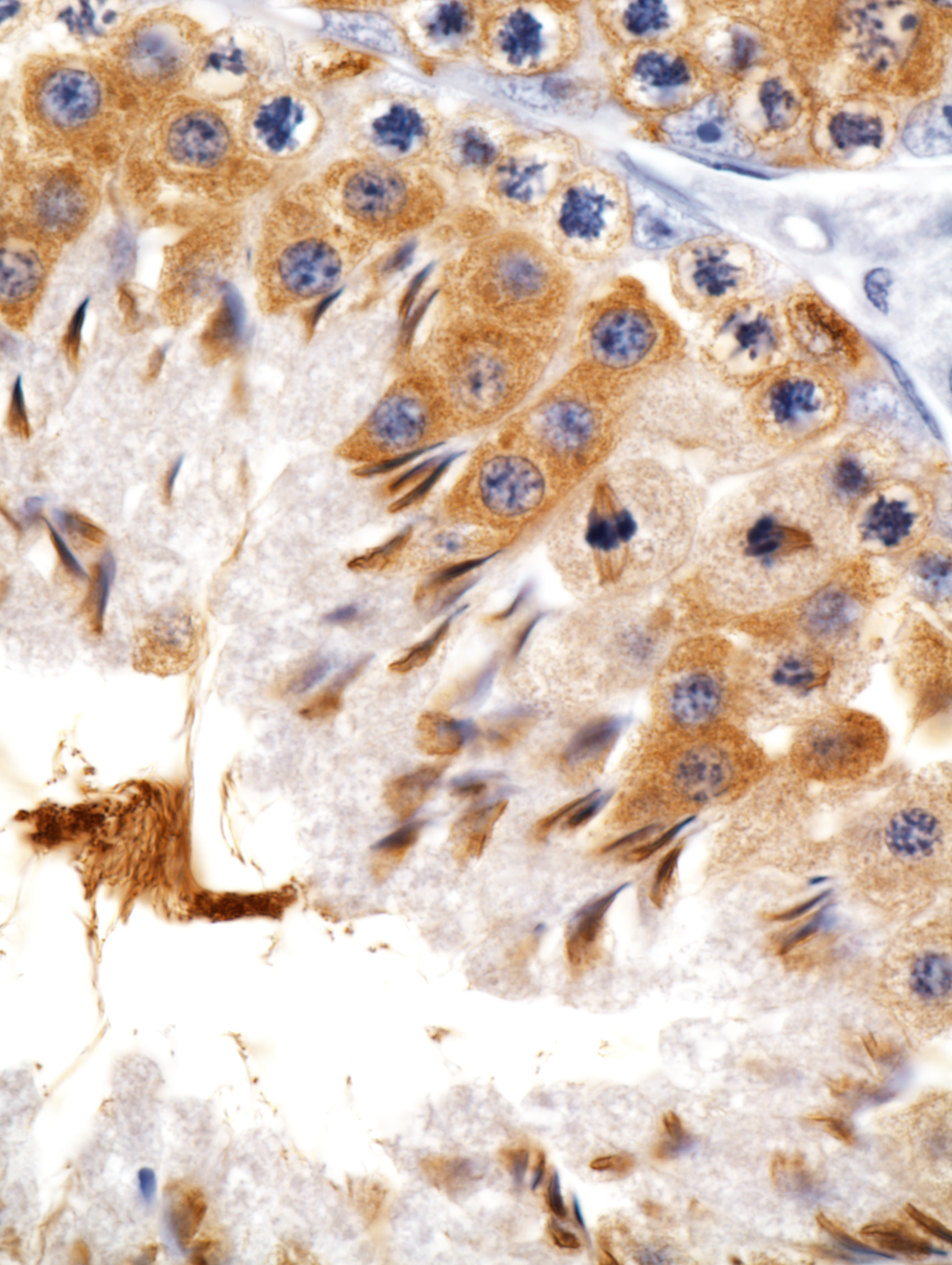

Supplement: Supplementary file 2 — Source data Fig. 2 [file 44319_2024_159_MOESM2_ESM.zip › EMBOR-2023-58207V1_SourceDataForFig2/2A/EMBOR-2023-58207V1_SourceDataForFig2a_i_iii.tif]

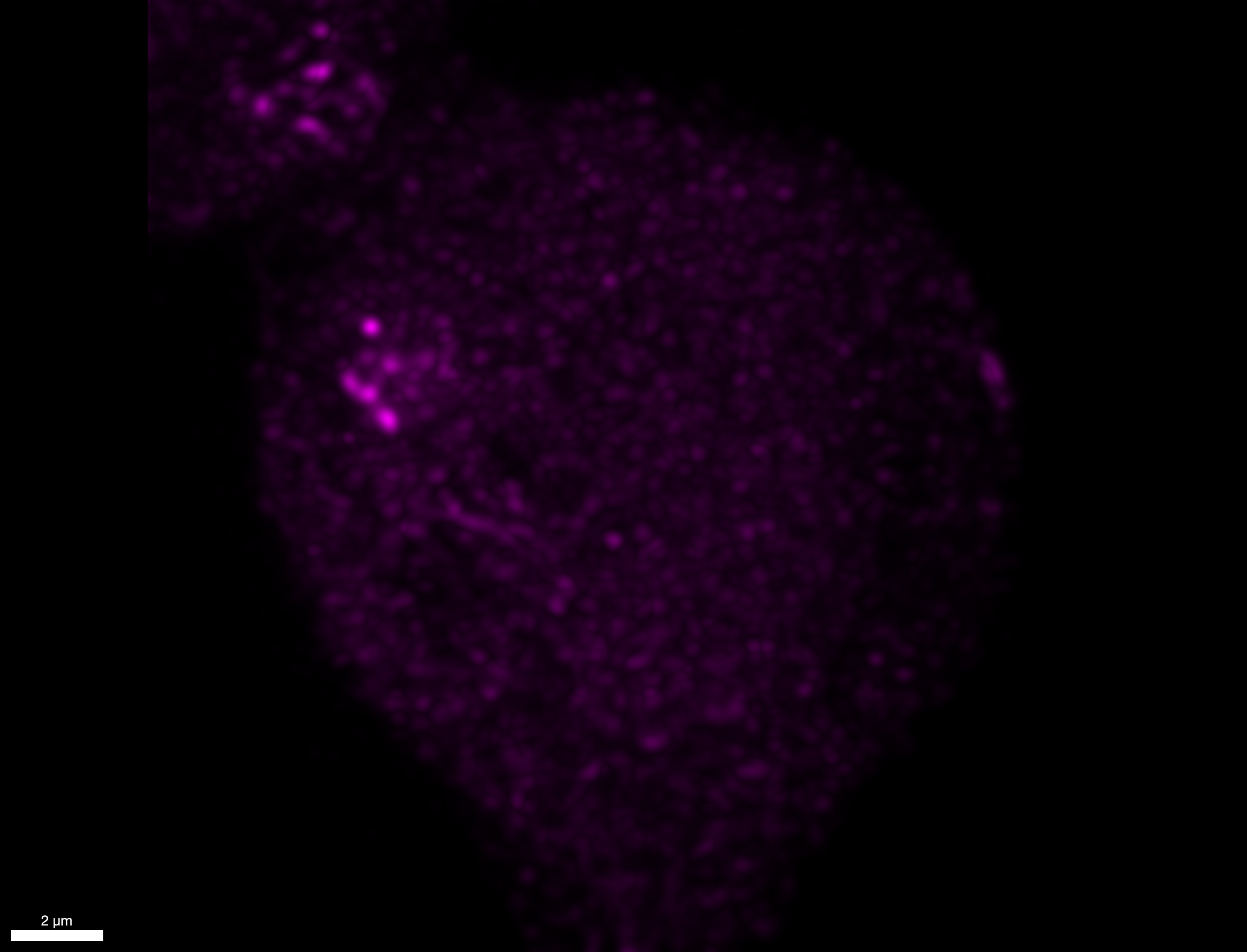

Supplement: Supplementary file 2 — Source data Fig. 2 [file 44319_2024_159_MOESM2_ESM.zip › EMBOR-2023-58207V1_SourceDataForFig2/2F/EMBOR-2023-58207V1_SourceDataForFig2F_centrin.tif]

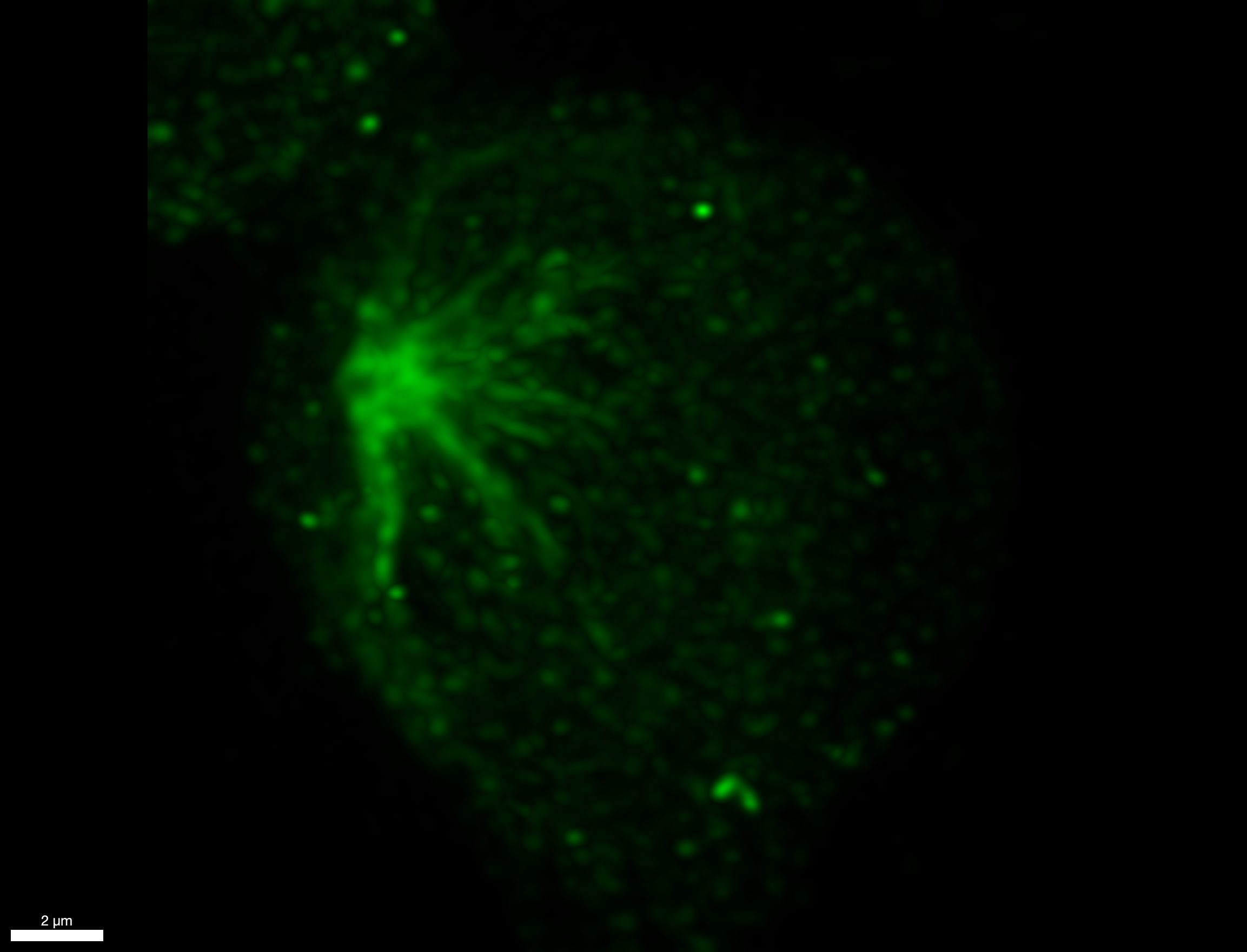

Supplement: Supplementary file 2 — Source data Fig. 2 [file 44319_2024_159_MOESM2_ESM.zip › EMBOR-2023-58207V1_SourceDataForFig2/2F/EMBOR-2023-58207V1_SourceDataForFig2F_beta tubulin.tif]

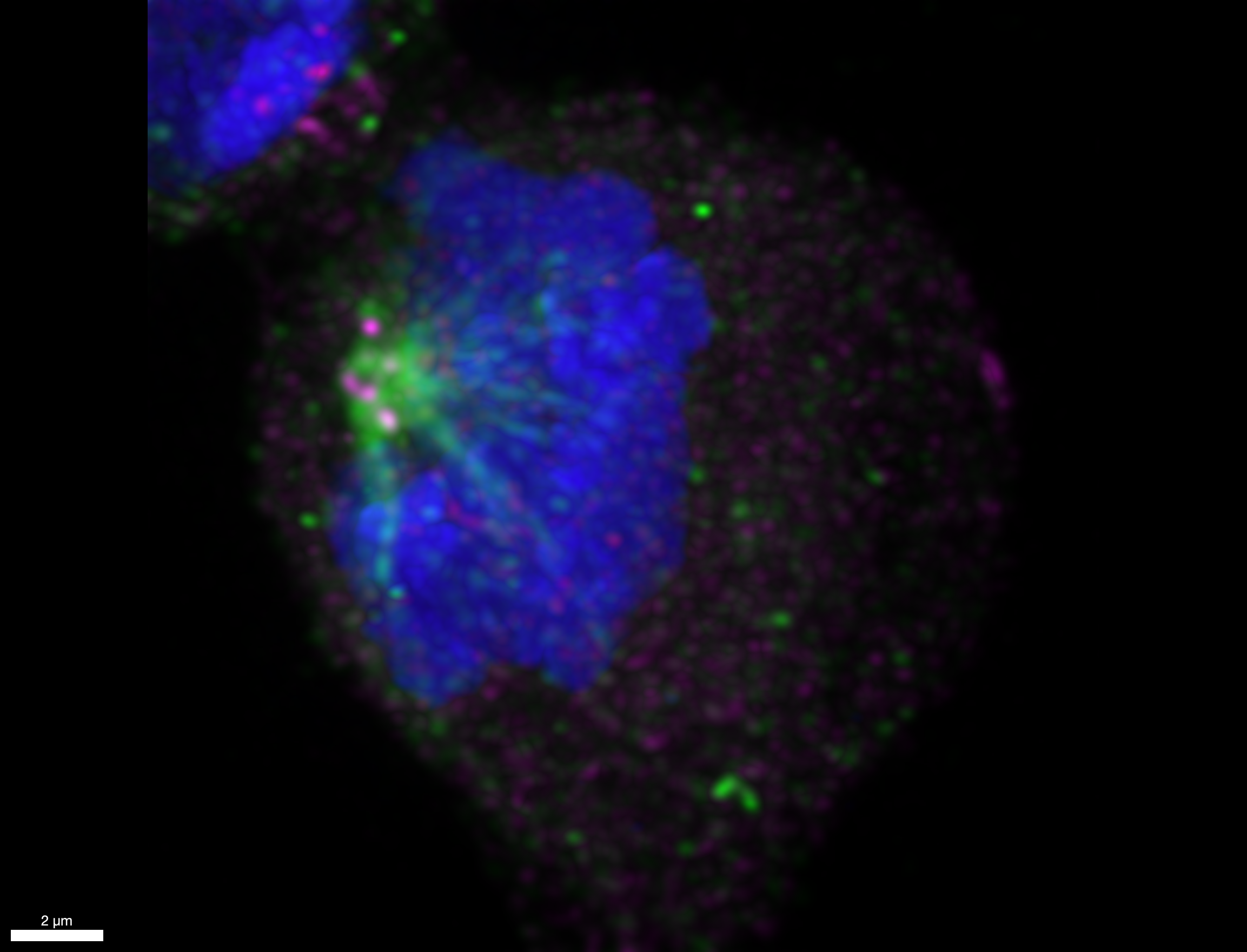

Supplement: Supplementary file 2 — Source data Fig. 2 [file 44319_2024_159_MOESM2_ESM.zip › EMBOR-2023-58207V1_SourceDataForFig2/2F/EMBOR-2023-58207V1_SourceDataForFig2F_merge.tif]

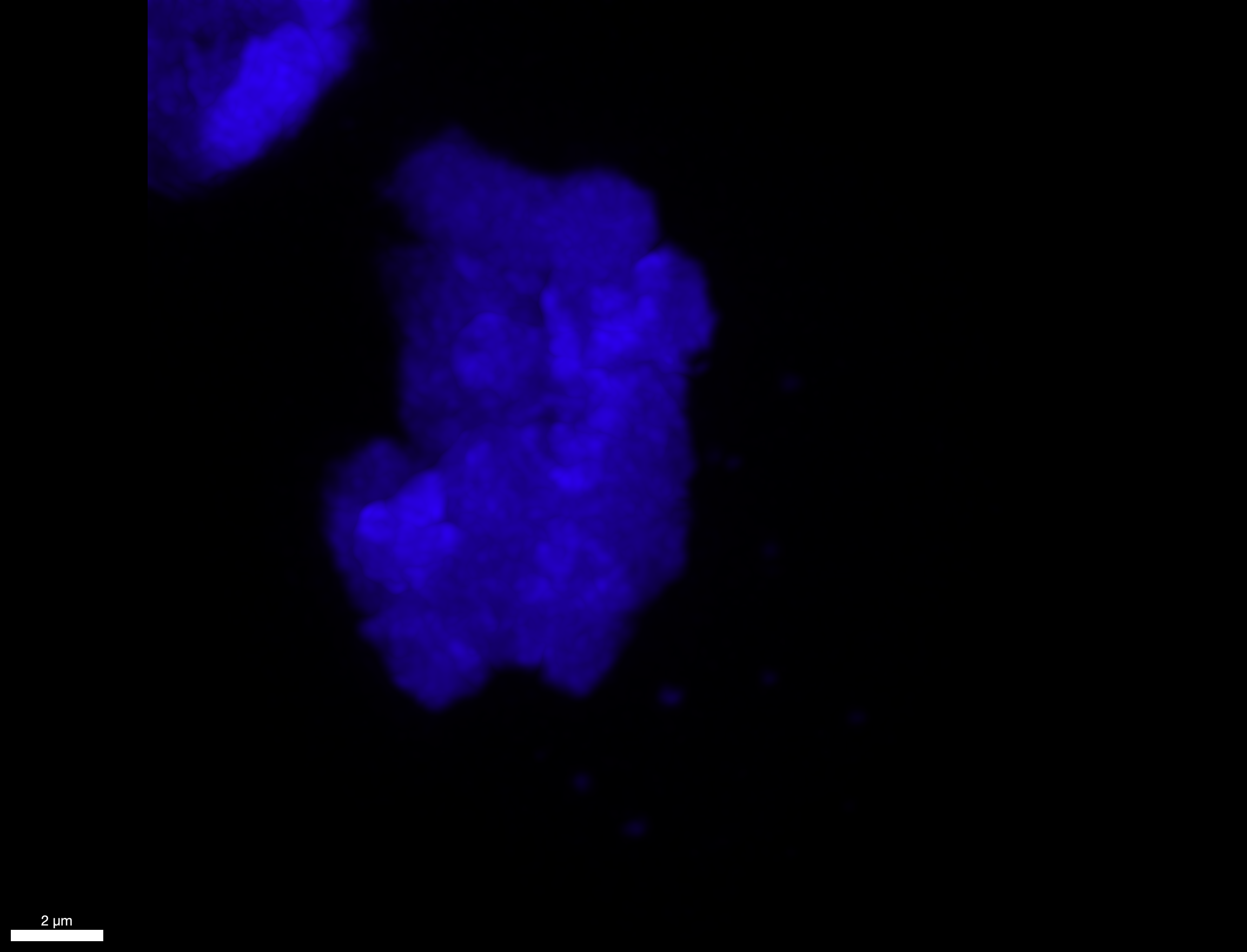

Supplement: Supplementary file 2 — Source data Fig. 2 [file 44319_2024_159_MOESM2_ESM.zip › EMBOR-2023-58207V1_SourceDataForFig2/2F/EMBOR-2023-58207V1_SourceDataForFig2F_DAPI.tif]

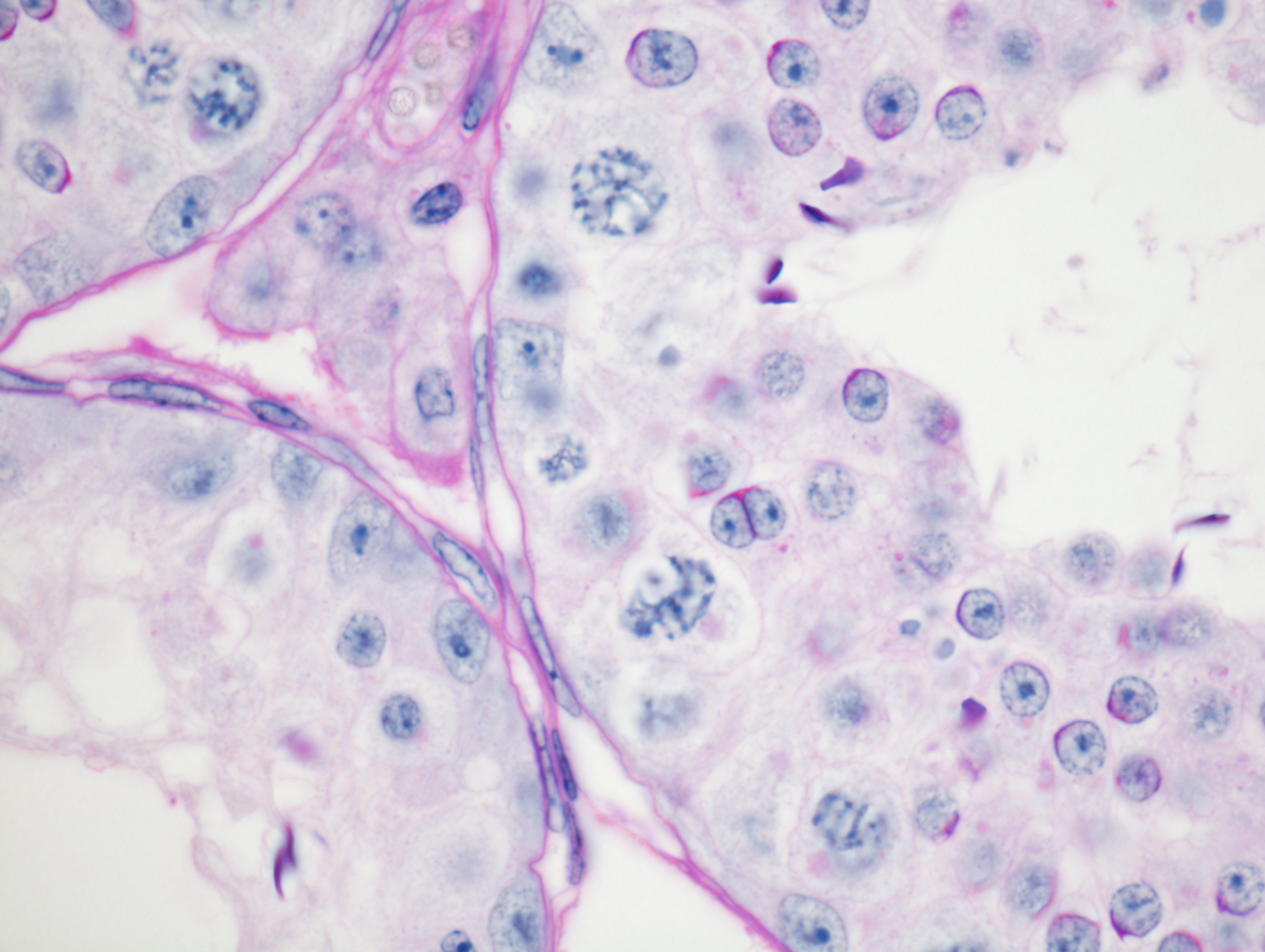

Supplement: Supplementary file 2 — Source data Fig. 2 [file 44319_2024_159_MOESM2_ESM.zip › EMBOR-2023-58207V1_SourceDataForFig2/2C/EMBOR-2023-58207V1_SourceDataForFig2c_Tube1GCKO:GCKO.tif]

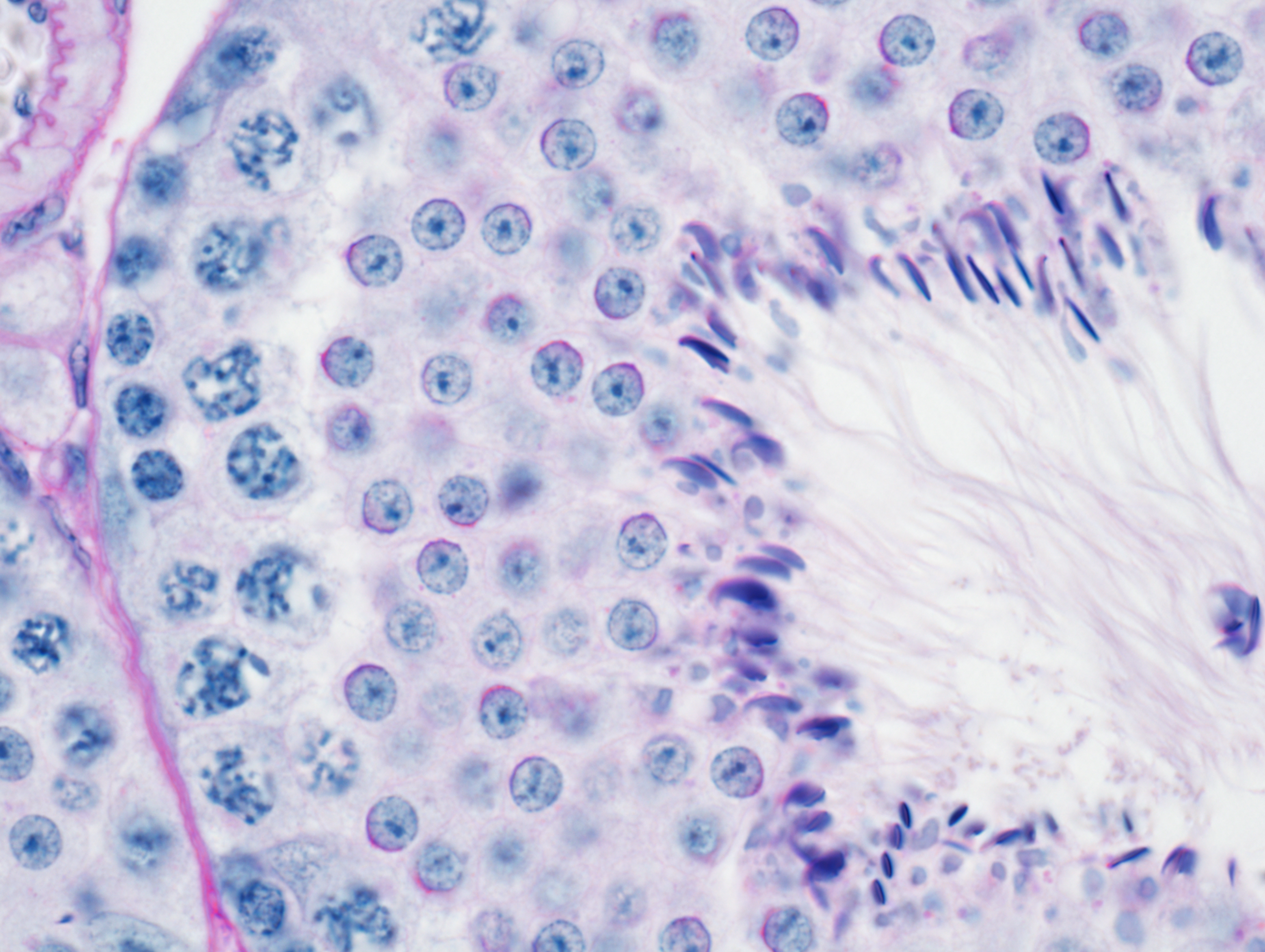

Supplement: Supplementary file 2 — Source data Fig. 2 [file 44319_2024_159_MOESM2_ESM.zip › EMBOR-2023-58207V1_SourceDataForFig2/2C/EMBOR-2023-58207V1_SourceDataForFig2c_Tube1Flox:Flox.tif]

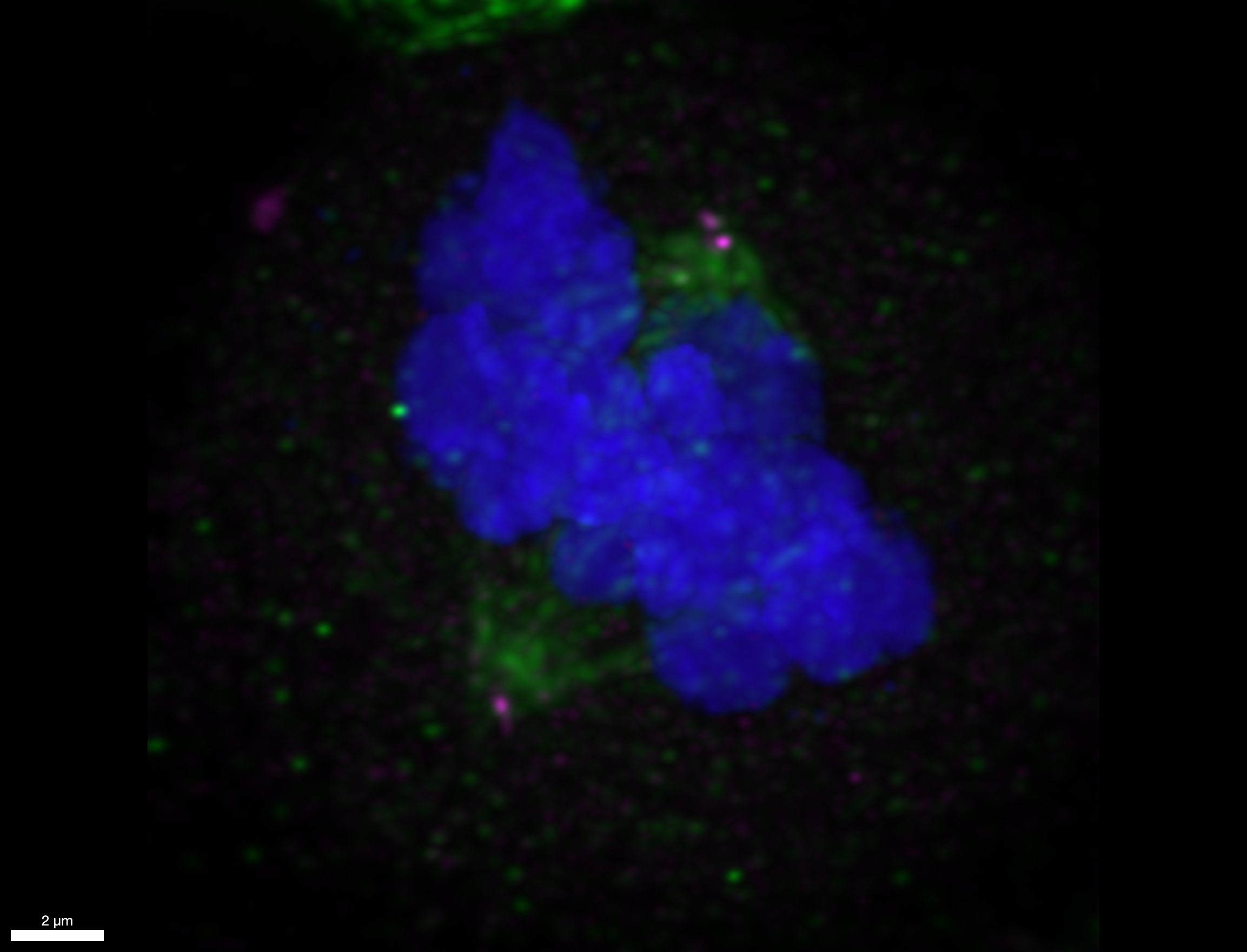

Supplement: Supplementary file 2 — Source data Fig. 2 [file 44319_2024_159_MOESM2_ESM.zip › EMBOR-2023-58207V1_SourceDataForFig2/2D/EMBOR-2023-58207V1_SourceDataForFig2D_merge.tif]

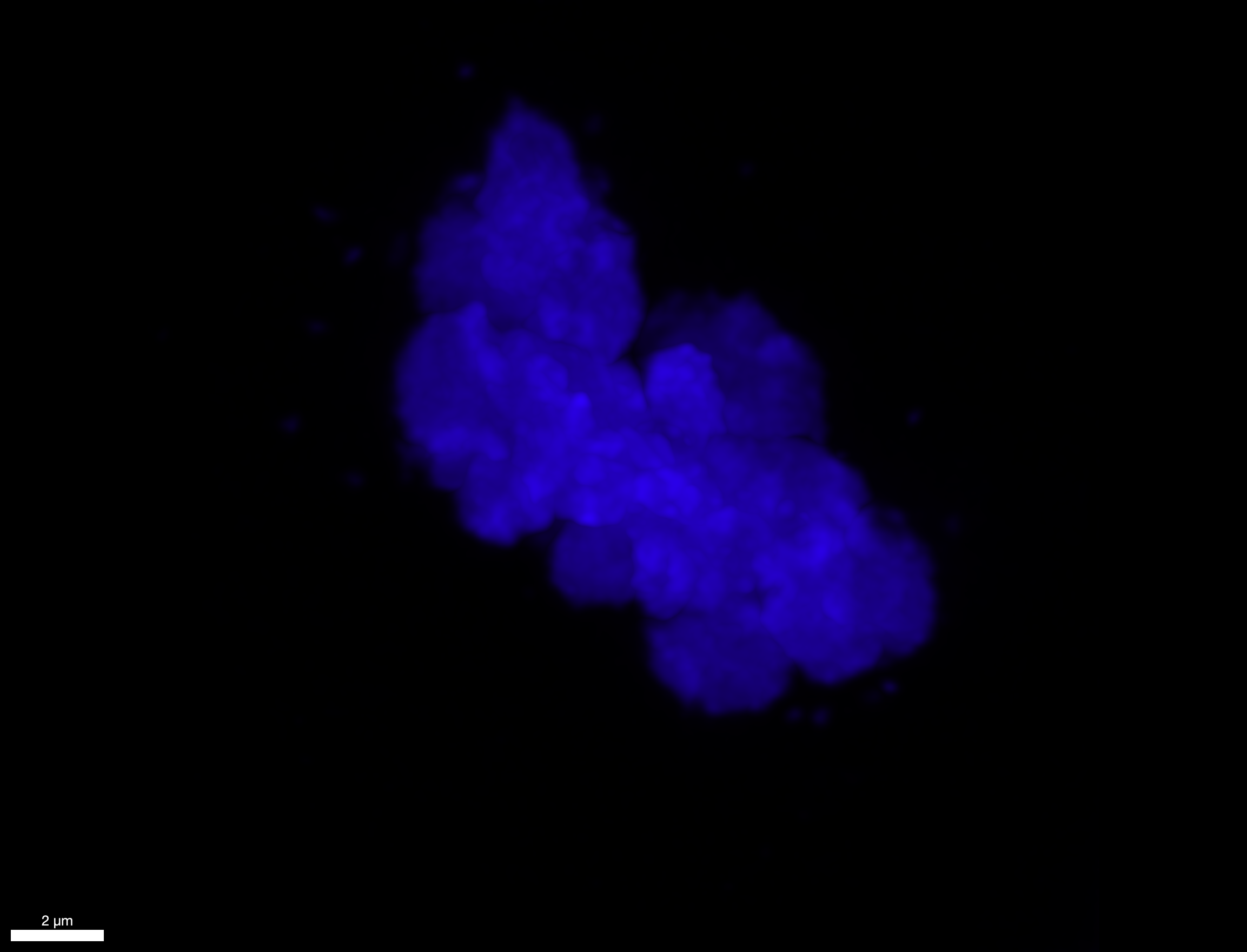

Supplement: Supplementary file 2 — Source data Fig. 2 [file 44319_2024_159_MOESM2_ESM.zip › EMBOR-2023-58207V1_SourceDataForFig2/2D/EMBOR-2023-58207V1_SourceDataForFig2D_DAPI.tif]

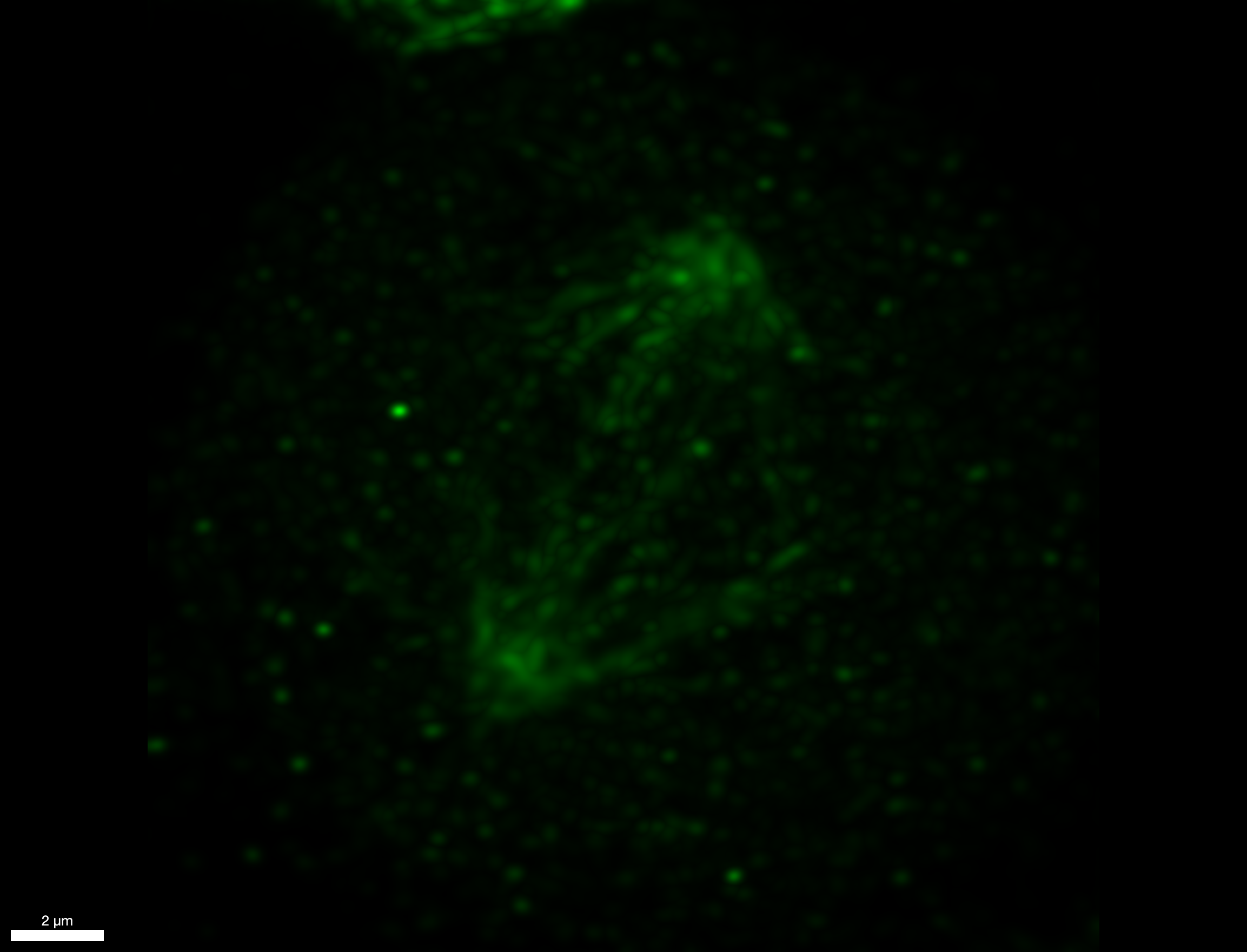

Supplement: Supplementary file 2 — Source data Fig. 2 [file 44319_2024_159_MOESM2_ESM.zip › EMBOR-2023-58207V1_SourceDataForFig2/2D/EMBOR-2023-58207V1_SourceDataForFig2D_beta tubulin.tif]

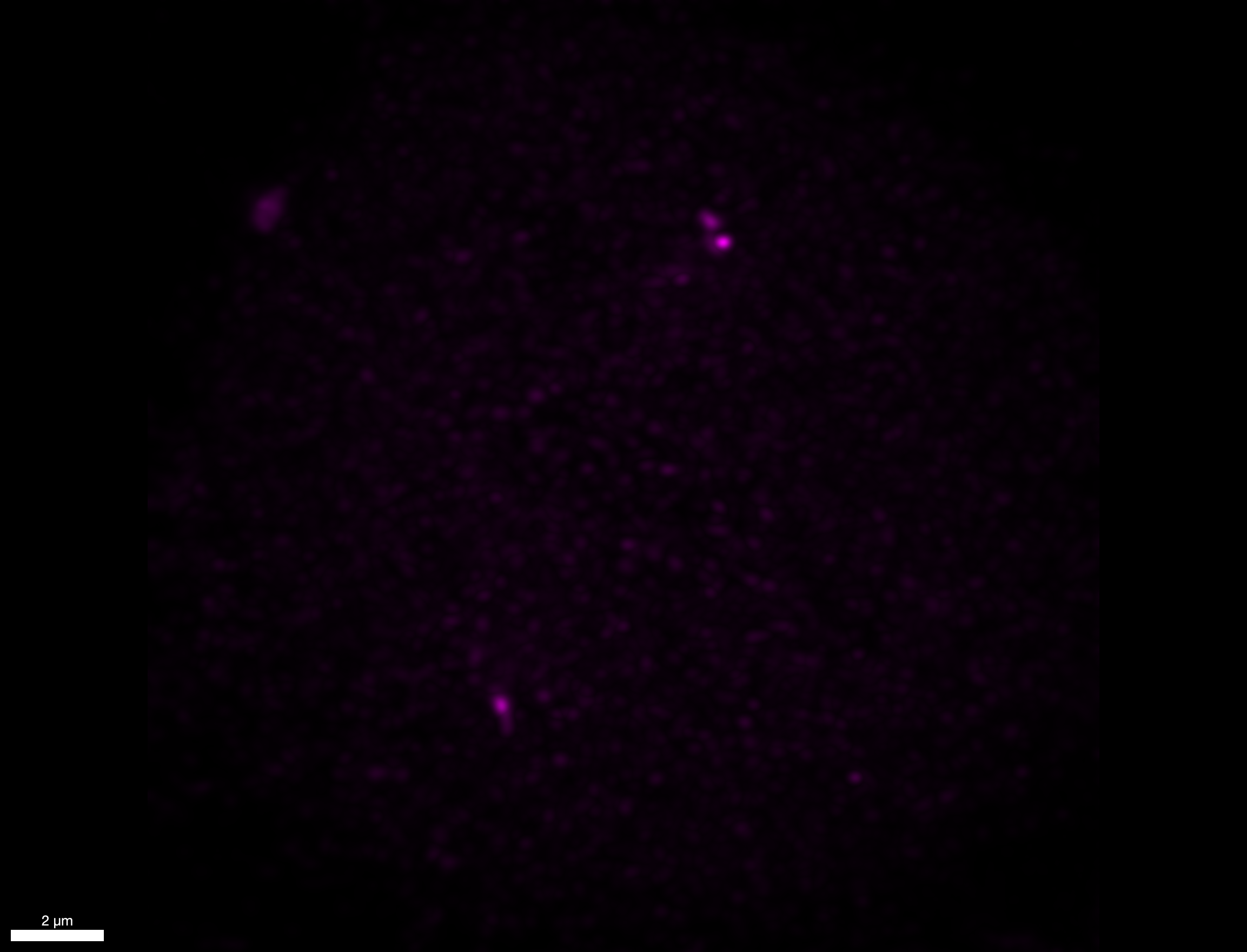

Supplement: Supplementary file 2 — Source data Fig. 2 [file 44319_2024_159_MOESM2_ESM.zip › EMBOR-2023-58207V1_SourceDataForFig2/2D/EMBOR-2023-58207V1_SourceDataForFig2D_centrin.tif]

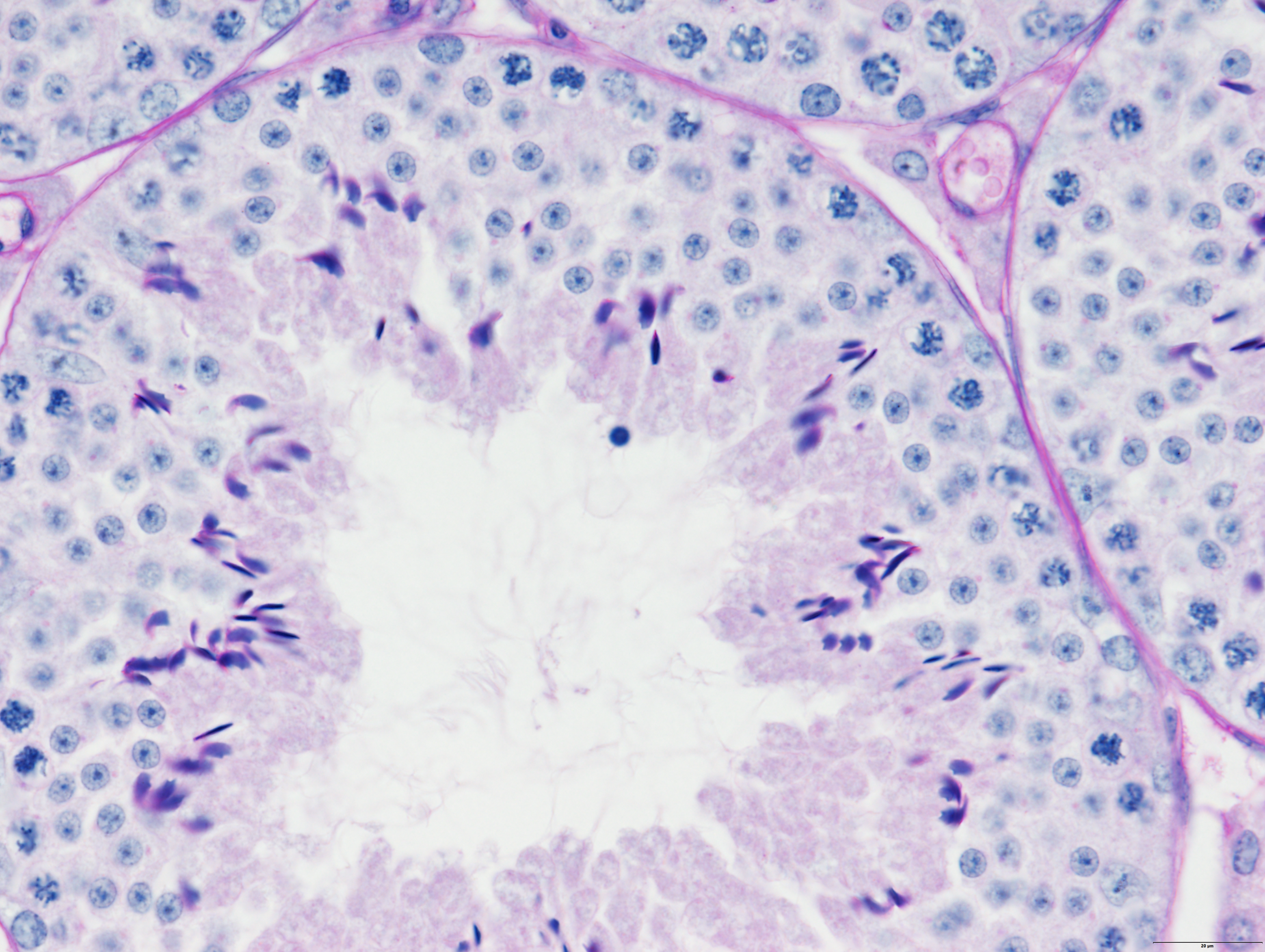

Supplement: Supplementary file 2 — Source data Fig. 2 [file 44319_2024_159_MOESM2_ESM.zip › EMBOR-2023-58207V1_SourceDataForFig2/2K/EMBOR-2023-58207V1_SourceDataForFig2k_Tube1Flox:Flox.tif]

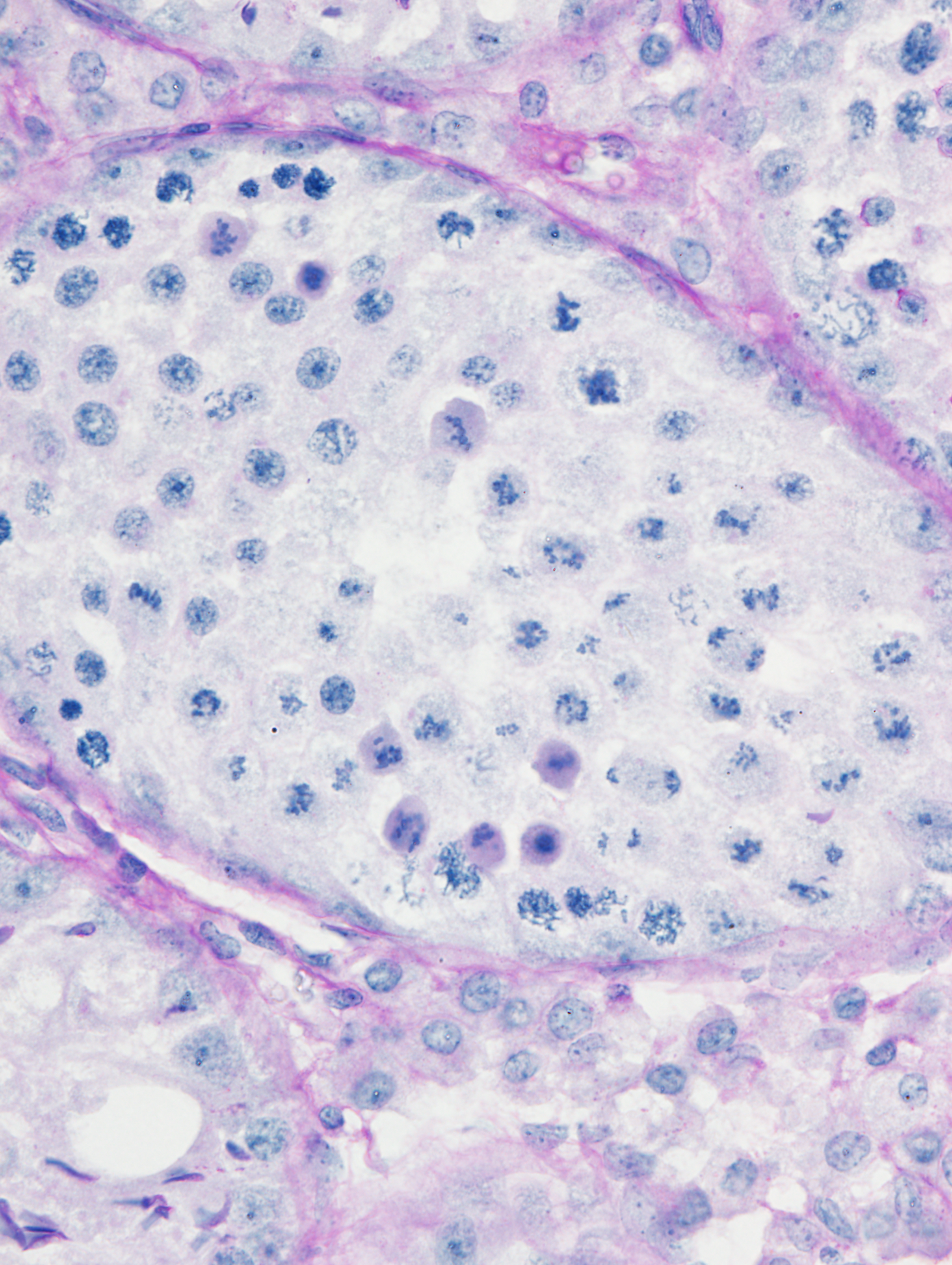

Supplement: Supplementary file 2 — Source data Fig. 2 [file 44319_2024_159_MOESM2_ESM.zip › EMBOR-2023-58207V1_SourceDataForFig2/2K/EMBOR-2023-58207V1_SourceDataForFig2k_Tube1GCKO:GCKO.tif]

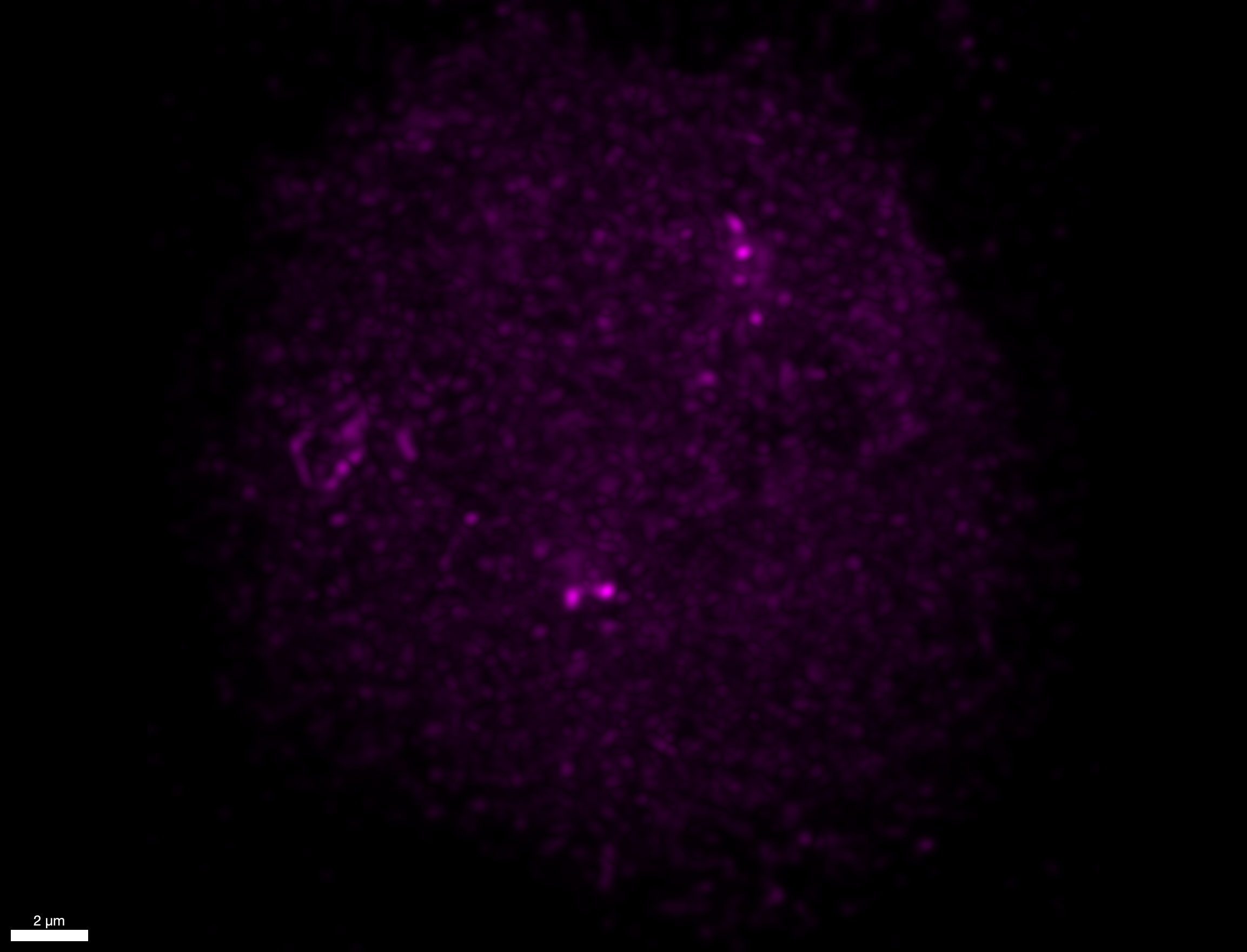

Supplement: Supplementary file 2 — Source data Fig. 2 [file 44319_2024_159_MOESM2_ESM.zip › EMBOR-2023-58207V1_SourceDataForFig2/2E/EMBOR-2023-58207V1_SourceDataForFig2E_centrin.tif]

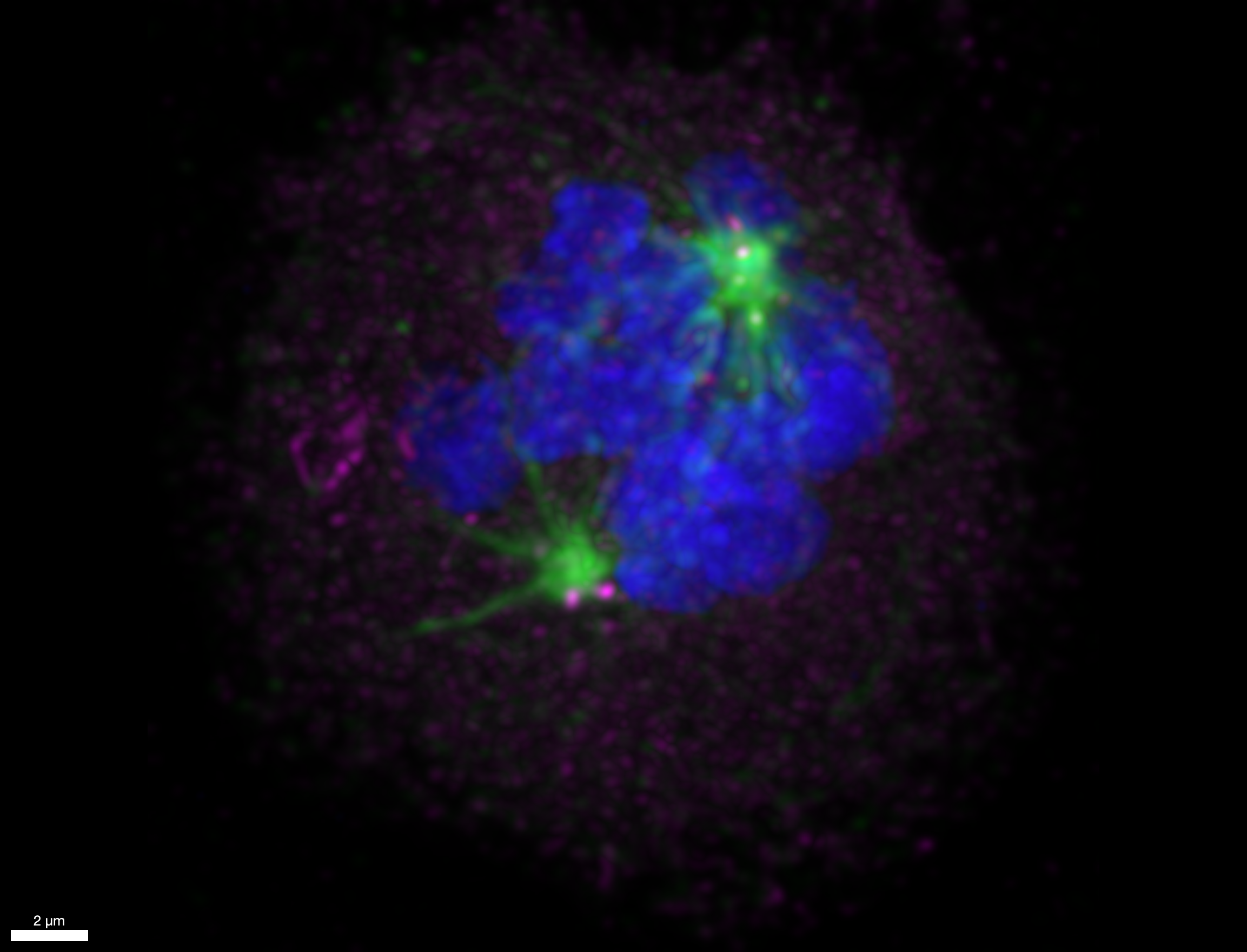

Supplement: Supplementary file 2 — Source data Fig. 2 [file 44319_2024_159_MOESM2_ESM.zip › EMBOR-2023-58207V1_SourceDataForFig2/2E/EMBOR-2023-58207V1_SourceDataForFig2E_merge.tif]

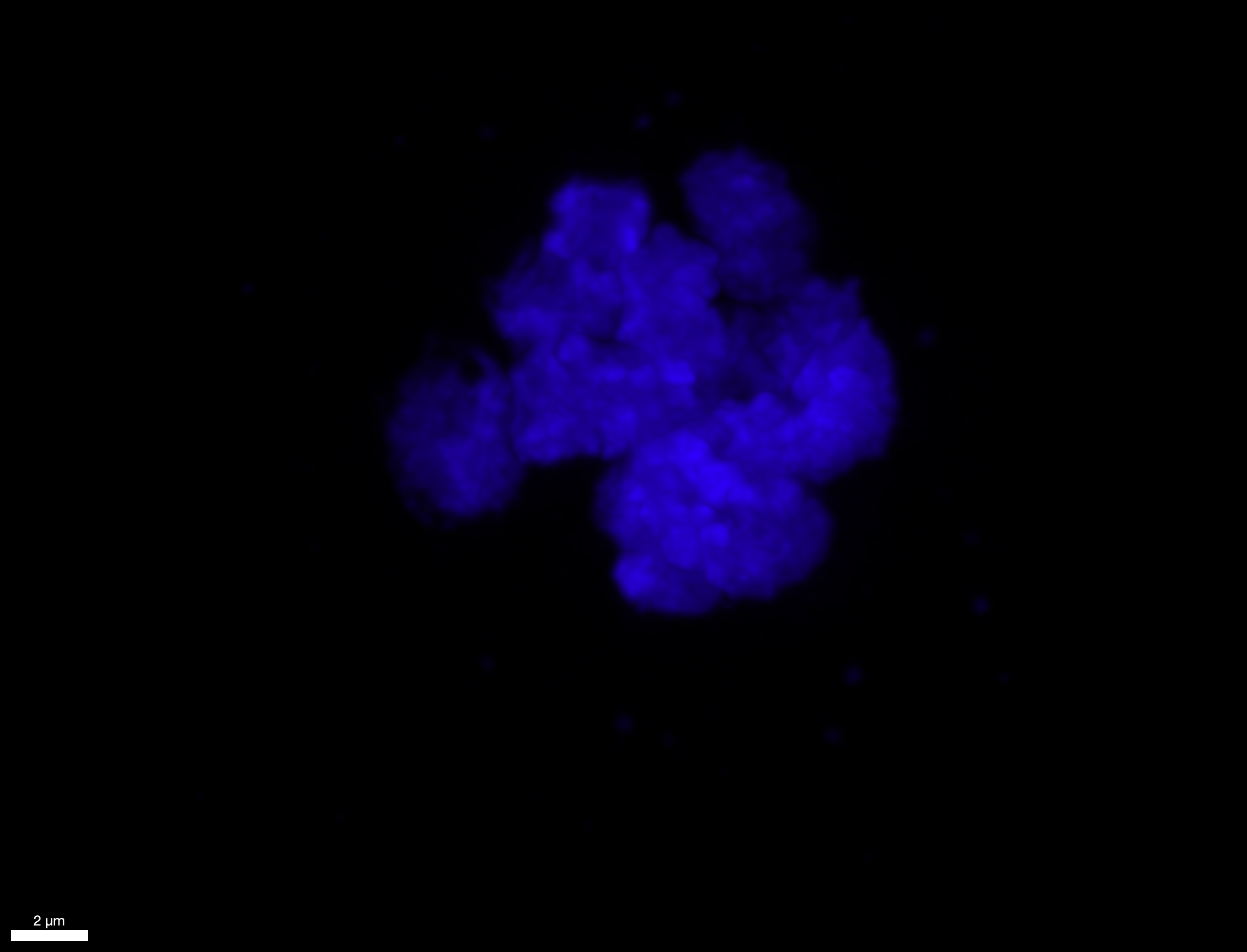

Supplement: Supplementary file 2 — Source data Fig. 2 [file 44319_2024_159_MOESM2_ESM.zip › EMBOR-2023-58207V1_SourceDataForFig2/2E/EMBOR-2023-58207V1_SourceDataForFig2E_DAPI.tif]

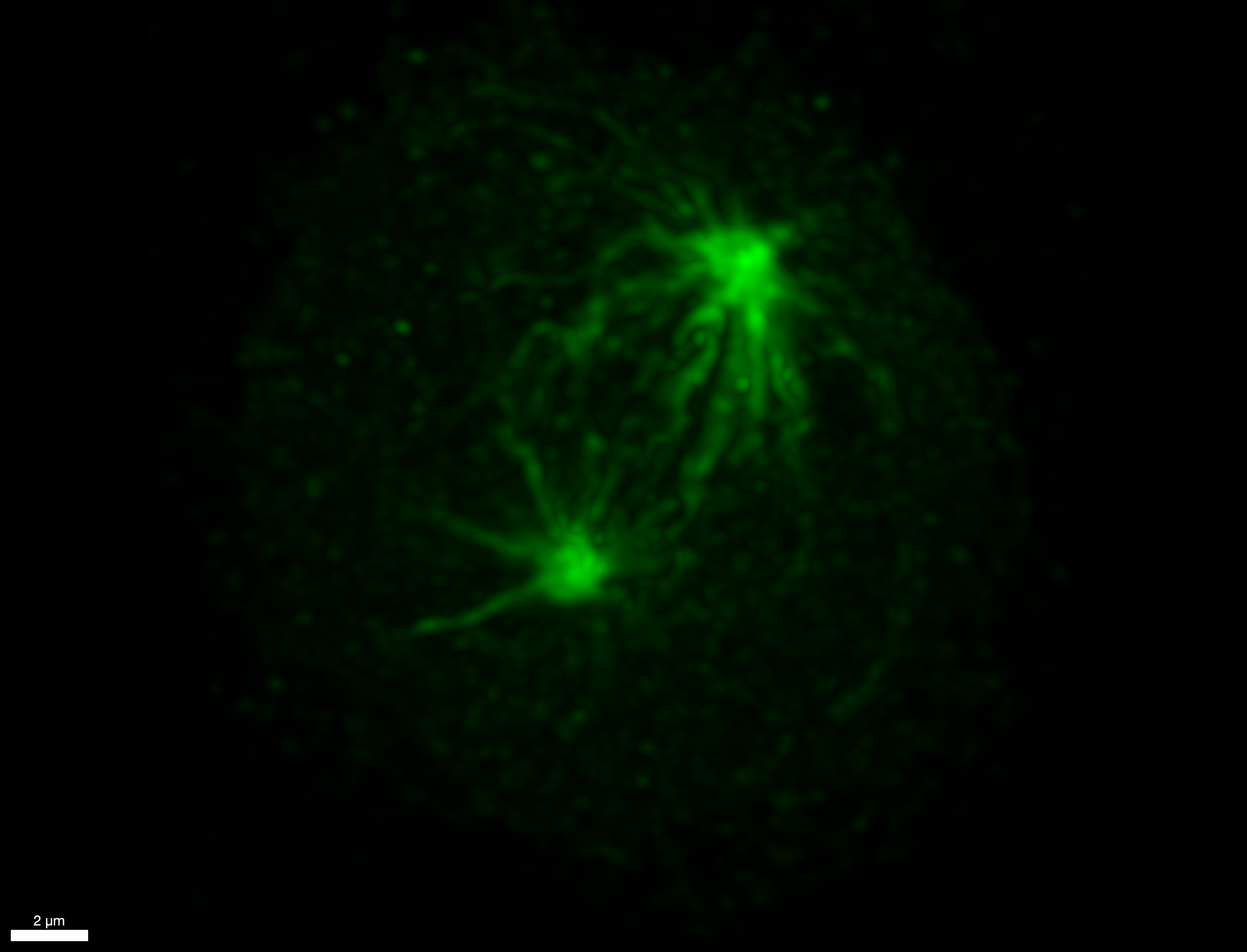

Supplement: Supplementary file 2 — Source data Fig. 2 [file 44319_2024_159_MOESM2_ESM.zip › EMBOR-2023-58207V1_SourceDataForFig2/2E/EMBOR-2023-58207V1_SourceDataForFig2E_beta tubulin.tif]

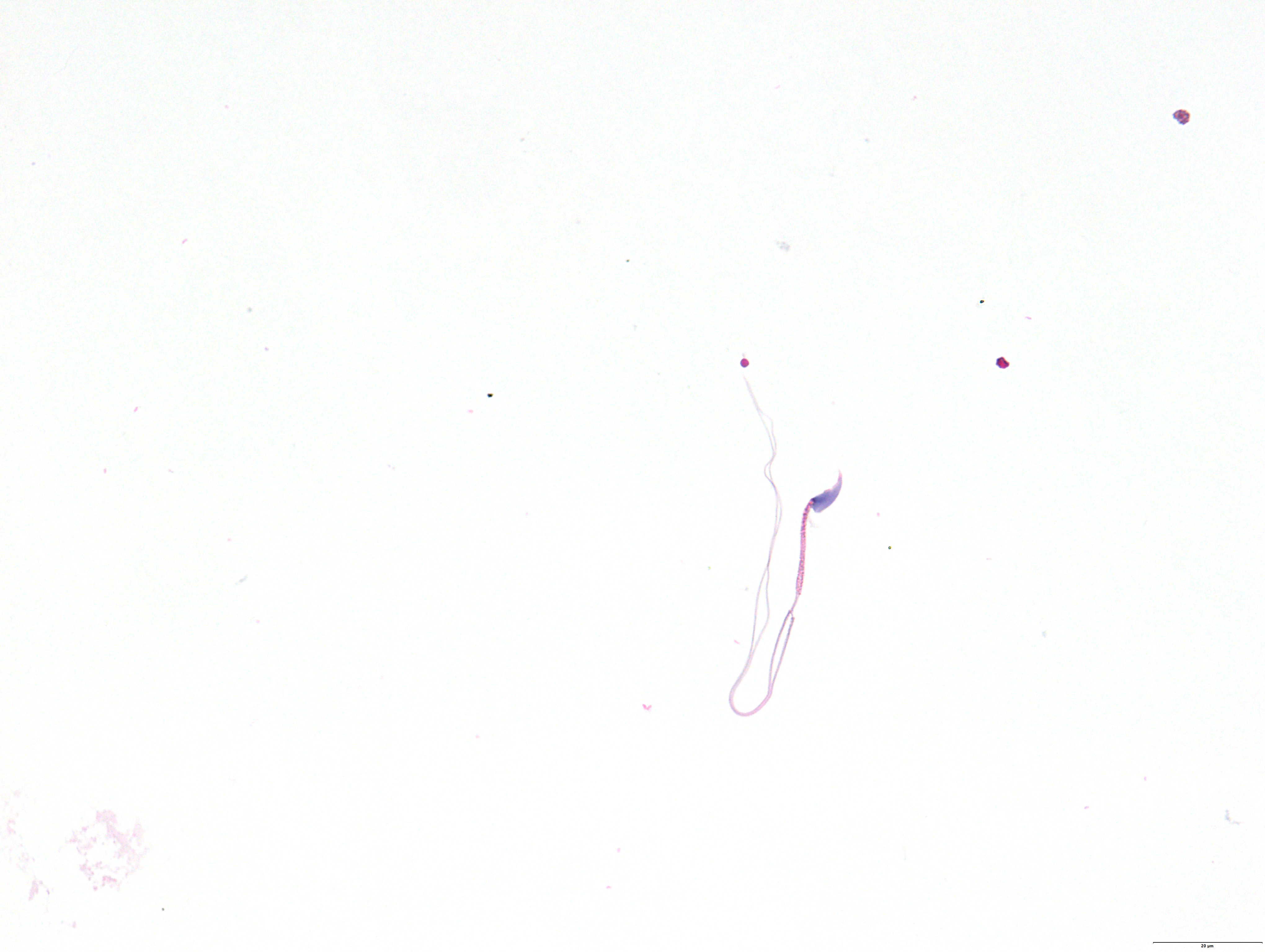

Supplement: Supplementary file 3 — Source data Fig. 3 [file 44319_2024_159_MOESM3_ESM.zip › EMBOR-2023-58207V1_SourceDataForFig3/3B/EMBOR-2023-58207V1_SourceDataForFig3b_Tube1GCKO:GCKO.jpg]

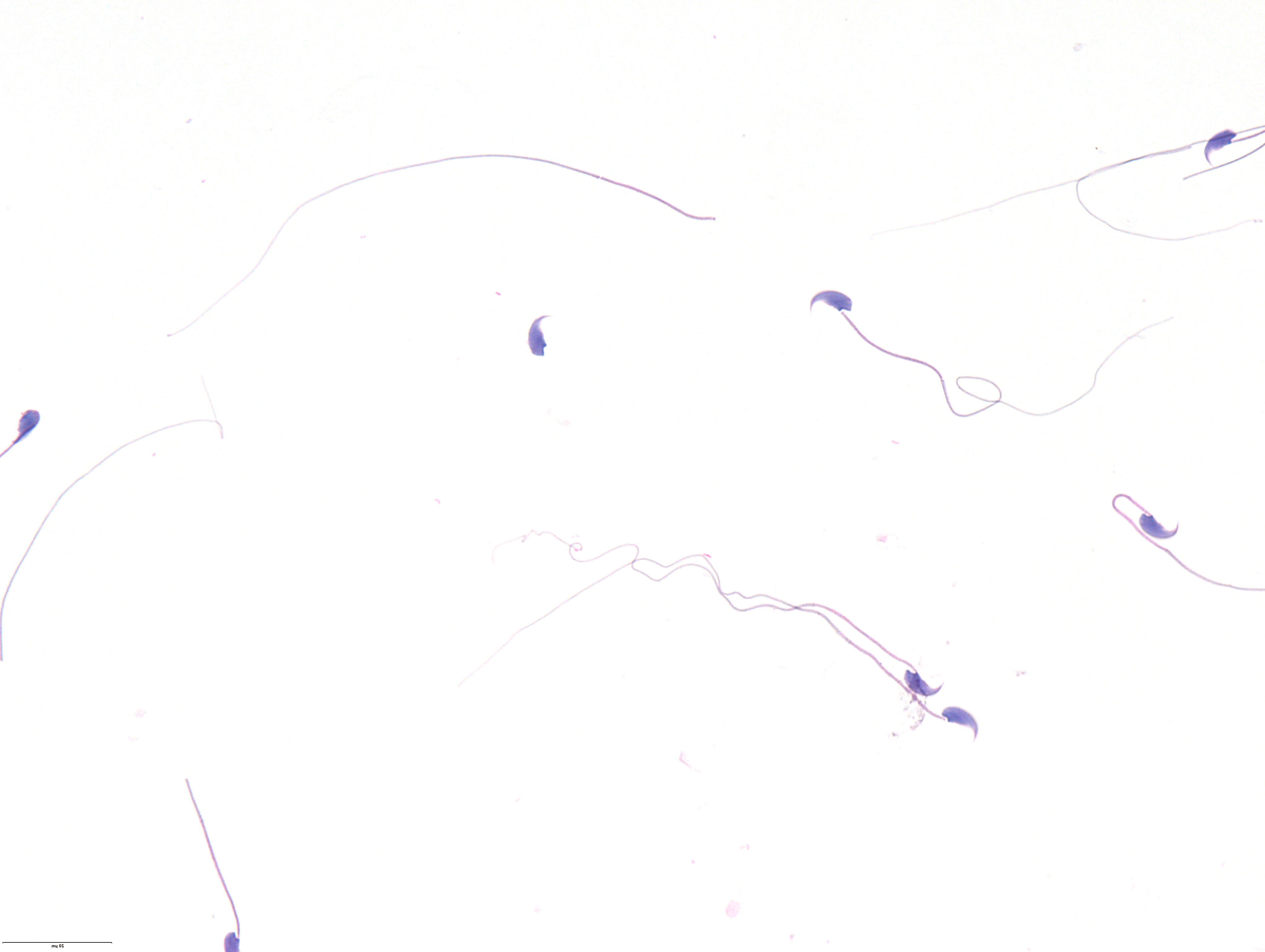

Supplement: Supplementary file 3 — Source data Fig. 3 [file 44319_2024_159_MOESM3_ESM.zip › EMBOR-2023-58207V1_SourceDataForFig3/3B/EMBOR-2023-58207V1_SourceDataForFig3b_Tube1Flox:Flox.jpg]

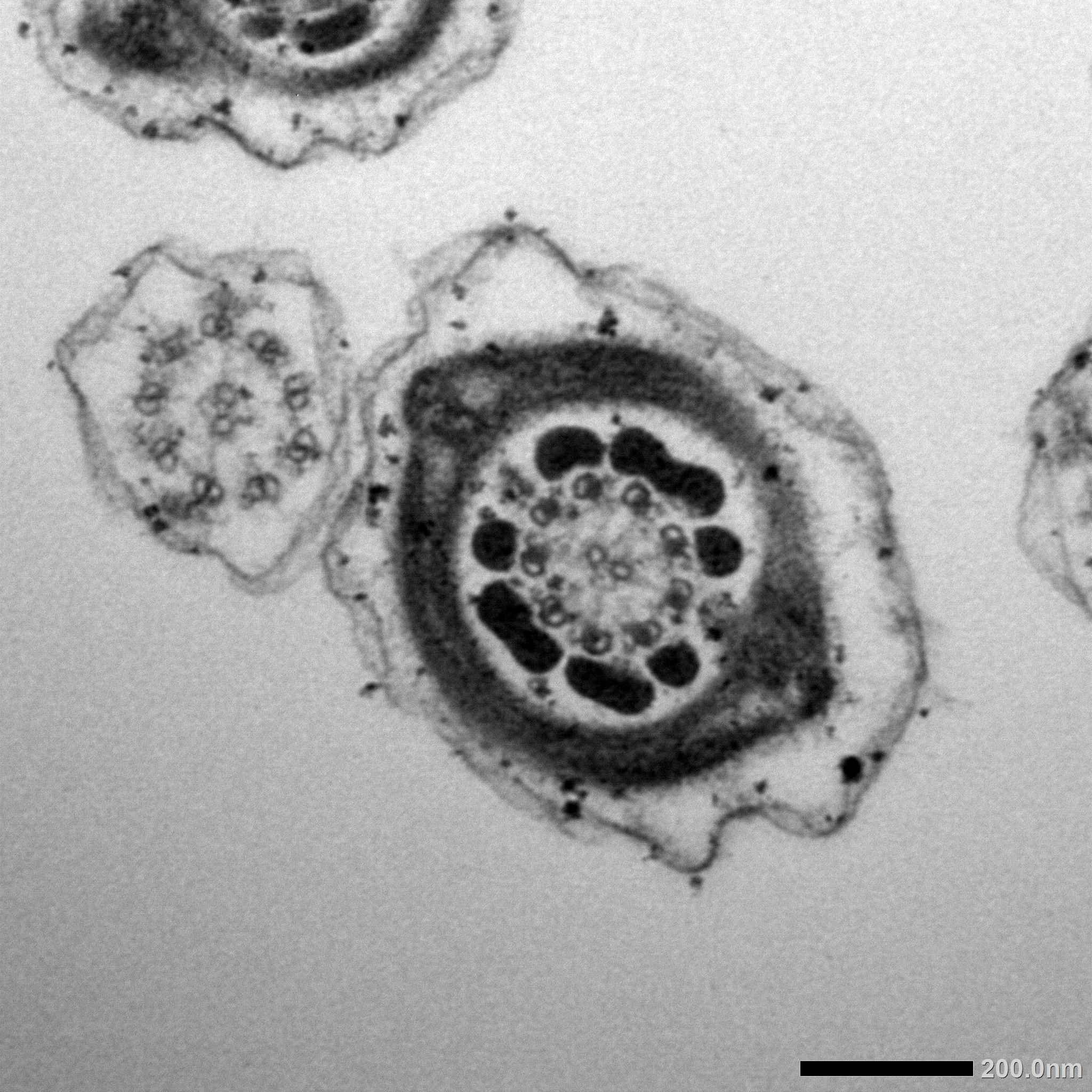

Supplement: Supplementary file 3 — Source data Fig. 3 [file 44319_2024_159_MOESM3_ESM.zip › EMBOR-2023-58207V1_SourceDataForFig3/3C/EMBOR-2023-58207V1_SourceDataForFig3c_Flox_end piece.jpg]

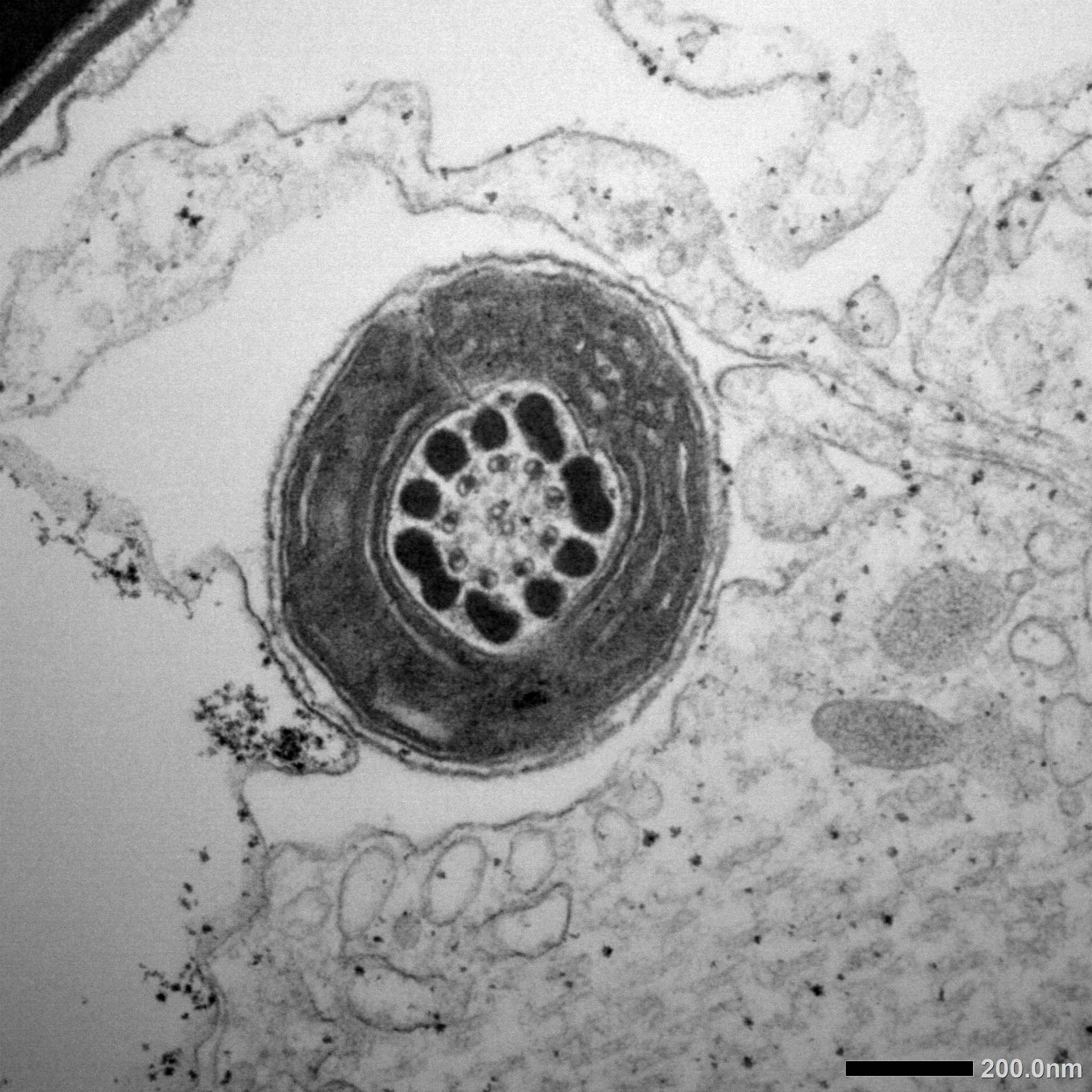

Supplement: Supplementary file 3 — Source data Fig. 3 [file 44319_2024_159_MOESM3_ESM.zip › EMBOR-2023-58207V1_SourceDataForFig3/3C/EMBOR-2023-58207V1_SourceDataForFig3c_GCKO_midpiece.jpg]

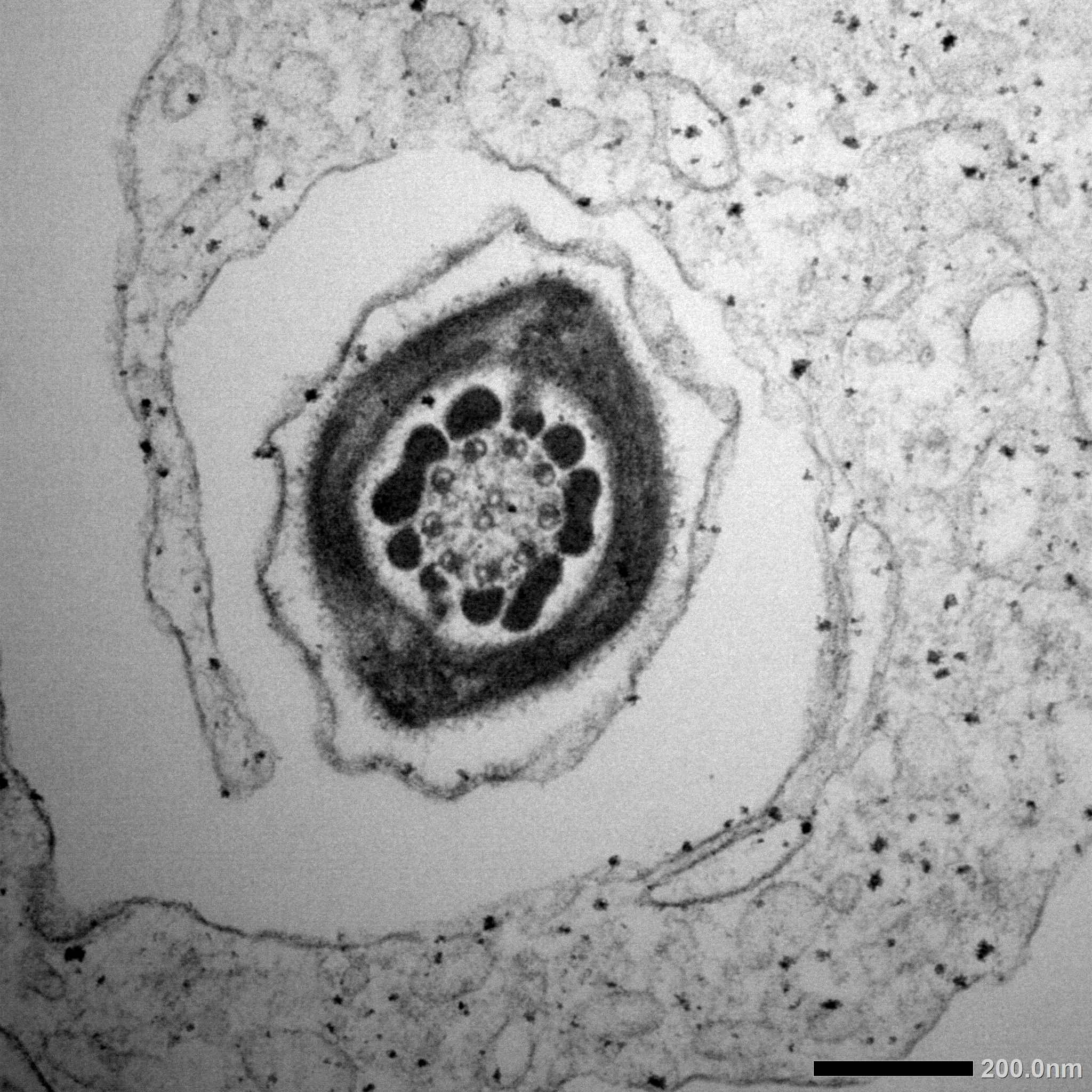

Supplement: Supplementary file 3 — Source data Fig. 3 [file 44319_2024_159_MOESM3_ESM.zip › EMBOR-2023-58207V1_SourceDataForFig3/3C/EMBOR-2023-58207V1_SourceDataForFig3c_GCKO_principal piece.jpg]

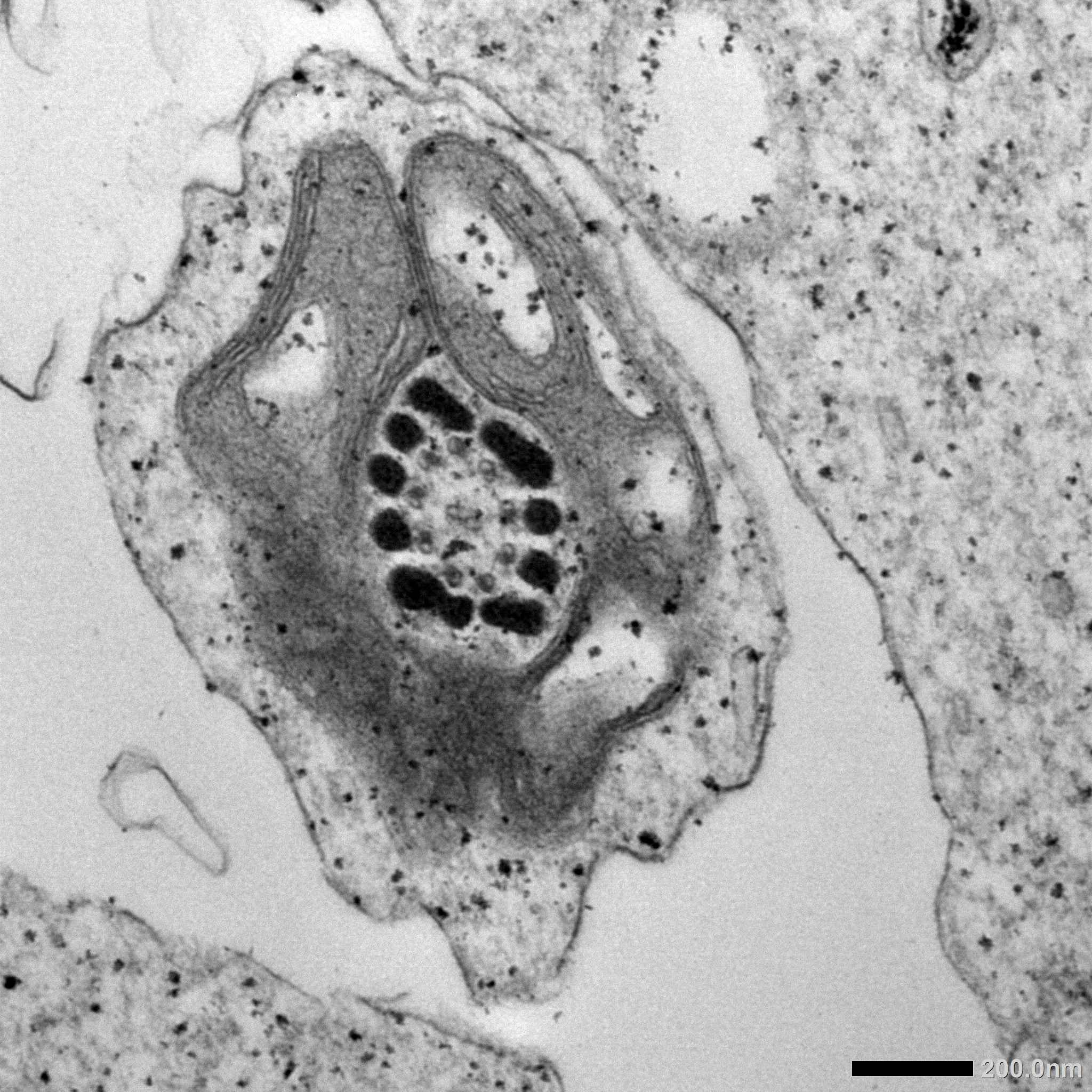

Supplement: Supplementary file 3 — Source data Fig. 3 [file 44319_2024_159_MOESM3_ESM.zip › EMBOR-2023-58207V1_SourceDataForFig3/3C/EMBOR-2023-58207V1_SourceDataForFig3c_Flox_midpiece.jpg]

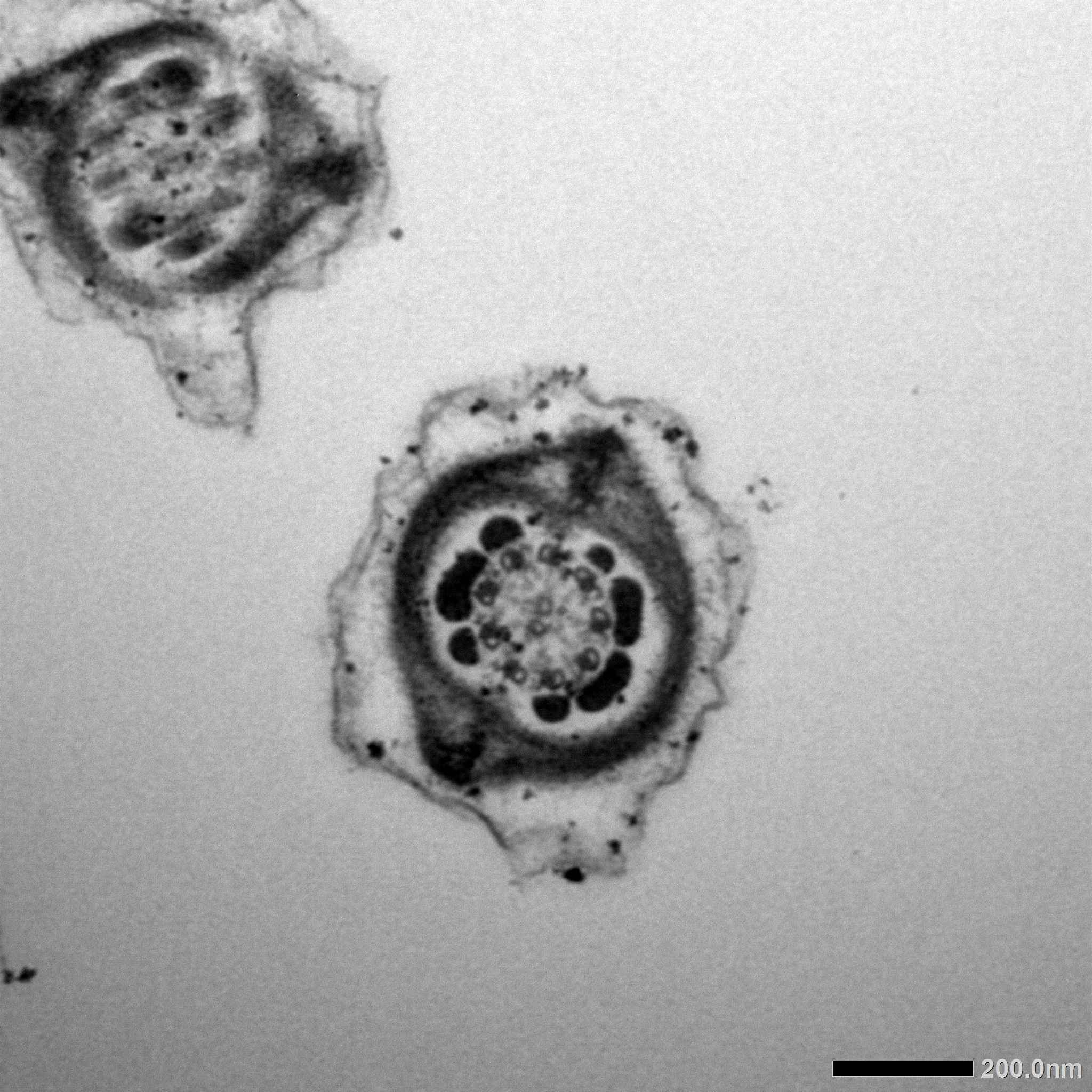

Supplement: Supplementary file 3 — Source data Fig. 3 [file 44319_2024_159_MOESM3_ESM.zip › EMBOR-2023-58207V1_SourceDataForFig3/3C/EMBOR-2023-58207V1_SourceDataForFig3c_Flox_principal piece.jpg]

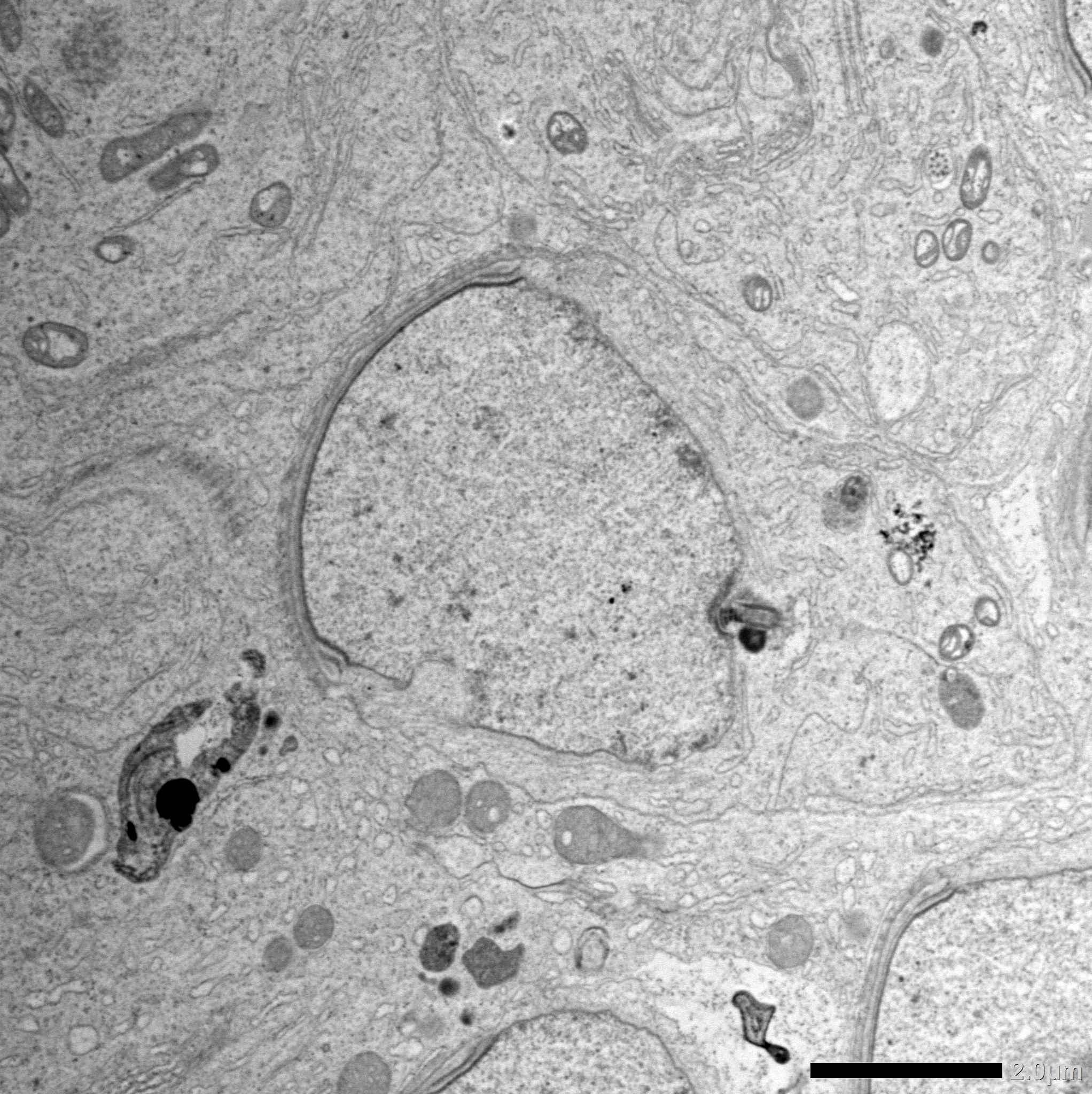

Supplement: Supplementary file 3 — Source data Fig. 3 [file 44319_2024_159_MOESM3_ESM.zip › EMBOR-2023-58207V1_SourceDataForFig3/3A/EMBOR-2023-58207V1_SourceDataForFig3a_GCKO_s9.jpg]

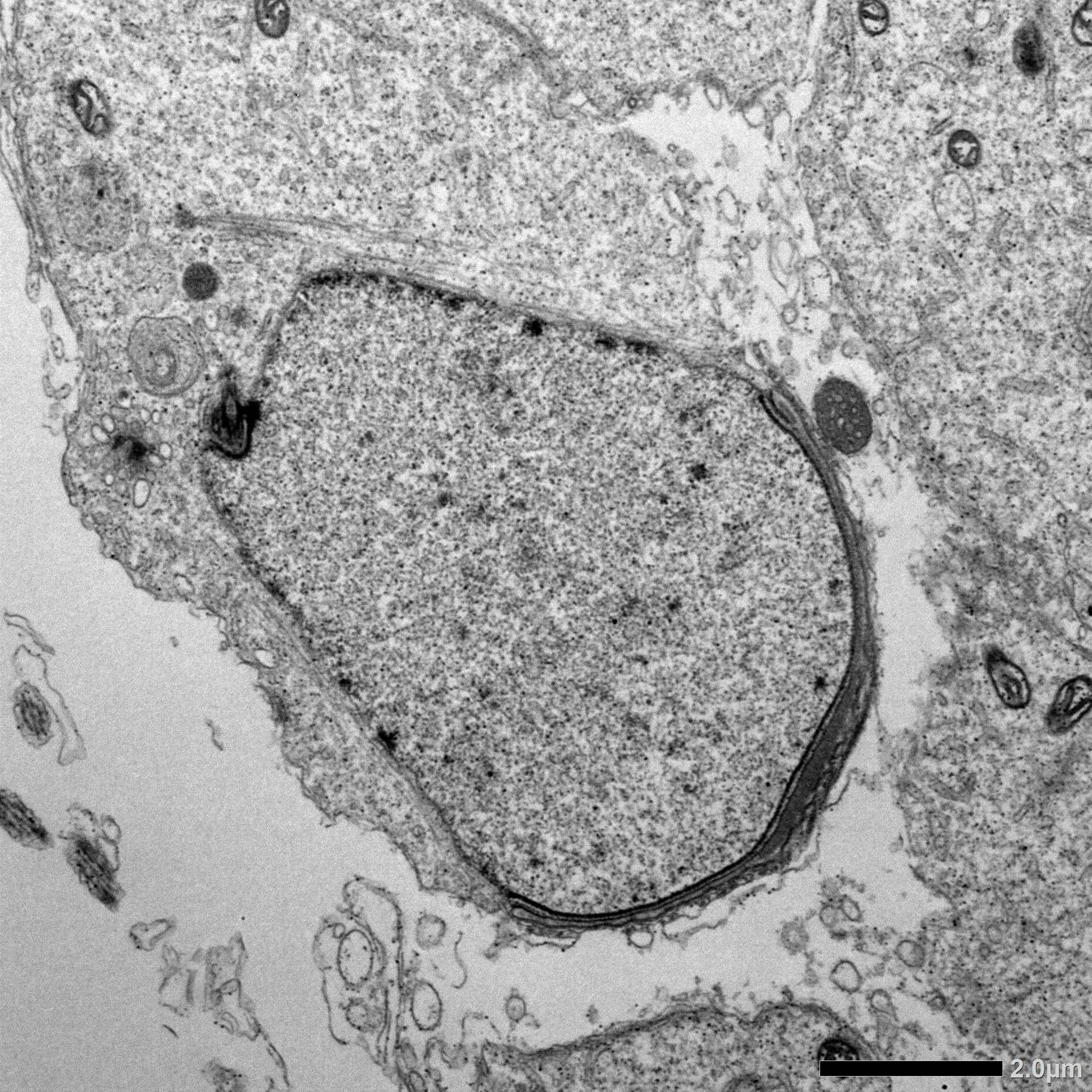

Supplement: Supplementary file 3 — Source data Fig. 3 [file 44319_2024_159_MOESM3_ESM.zip › EMBOR-2023-58207V1_SourceDataForFig3/3A/EMBOR-2023-58207V1_SourceDataForFig3a_Flox_s9.jpg]

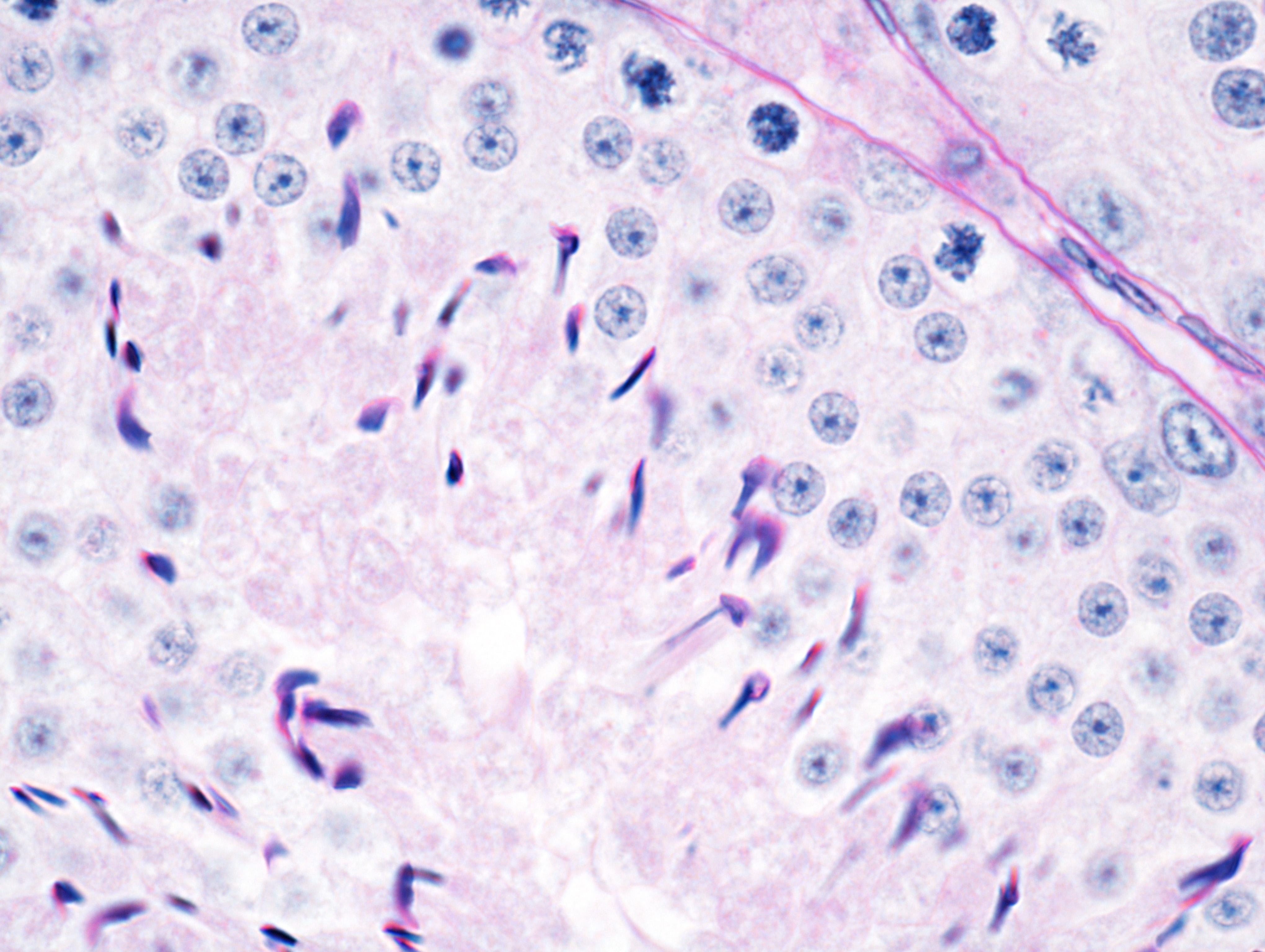

Supplement: Supplementary file 4 — Source data Fig. 4 [file 44319_2024_159_MOESM4_ESM.zip › EMBOR-2023-58207V1_SourceDataForFig4/4B/EMBOR-2023-58207V1_SourceDataForFig4b_GCKO.jpg]

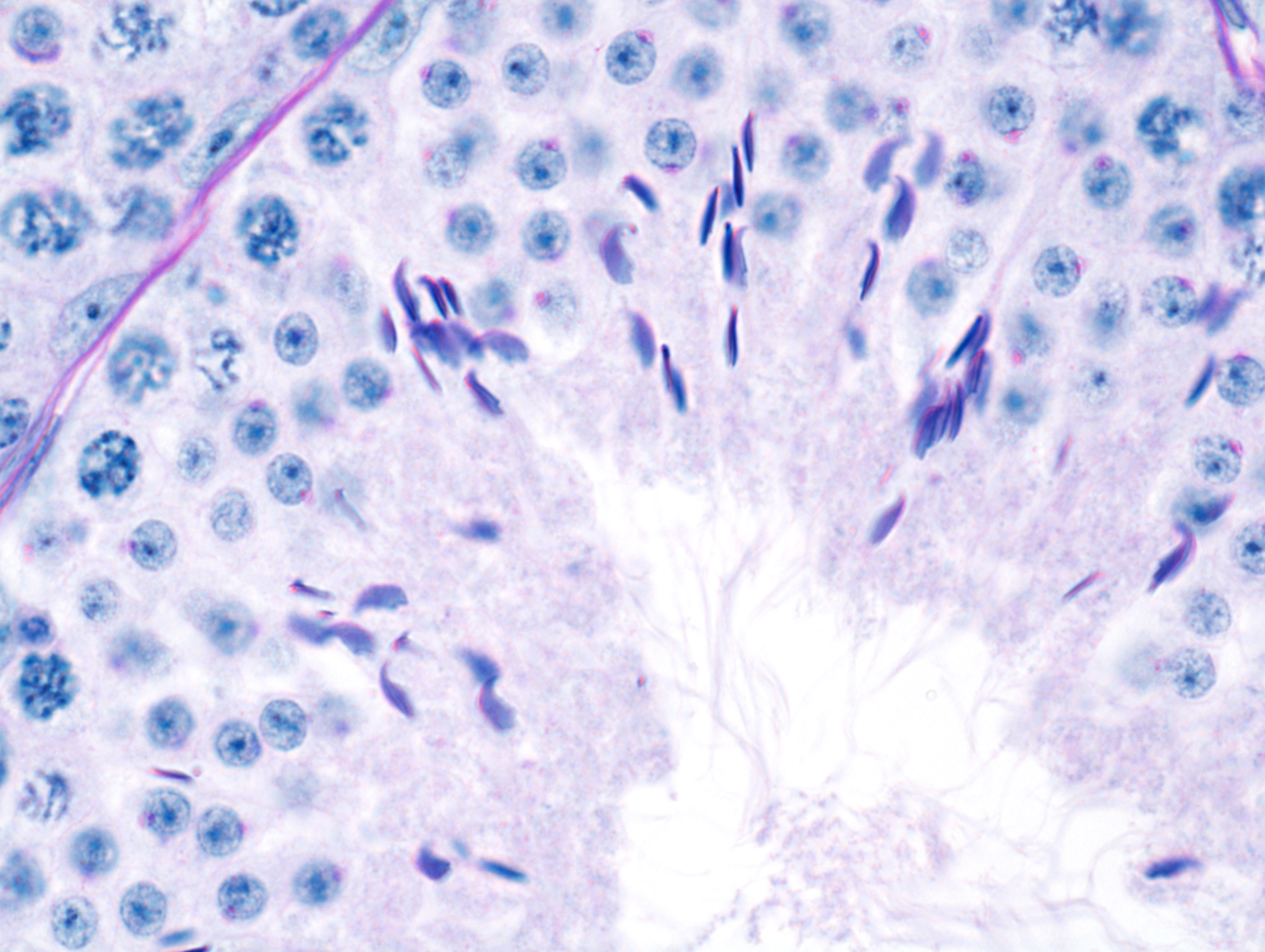

Supplement: Supplementary file 4 — Source data Fig. 4 [file 44319_2024_159_MOESM4_ESM.zip › EMBOR-2023-58207V1_SourceDataForFig4/4B/EMBOR-2023-58207V1_SourceDataForFig4b_Flox.jpg]

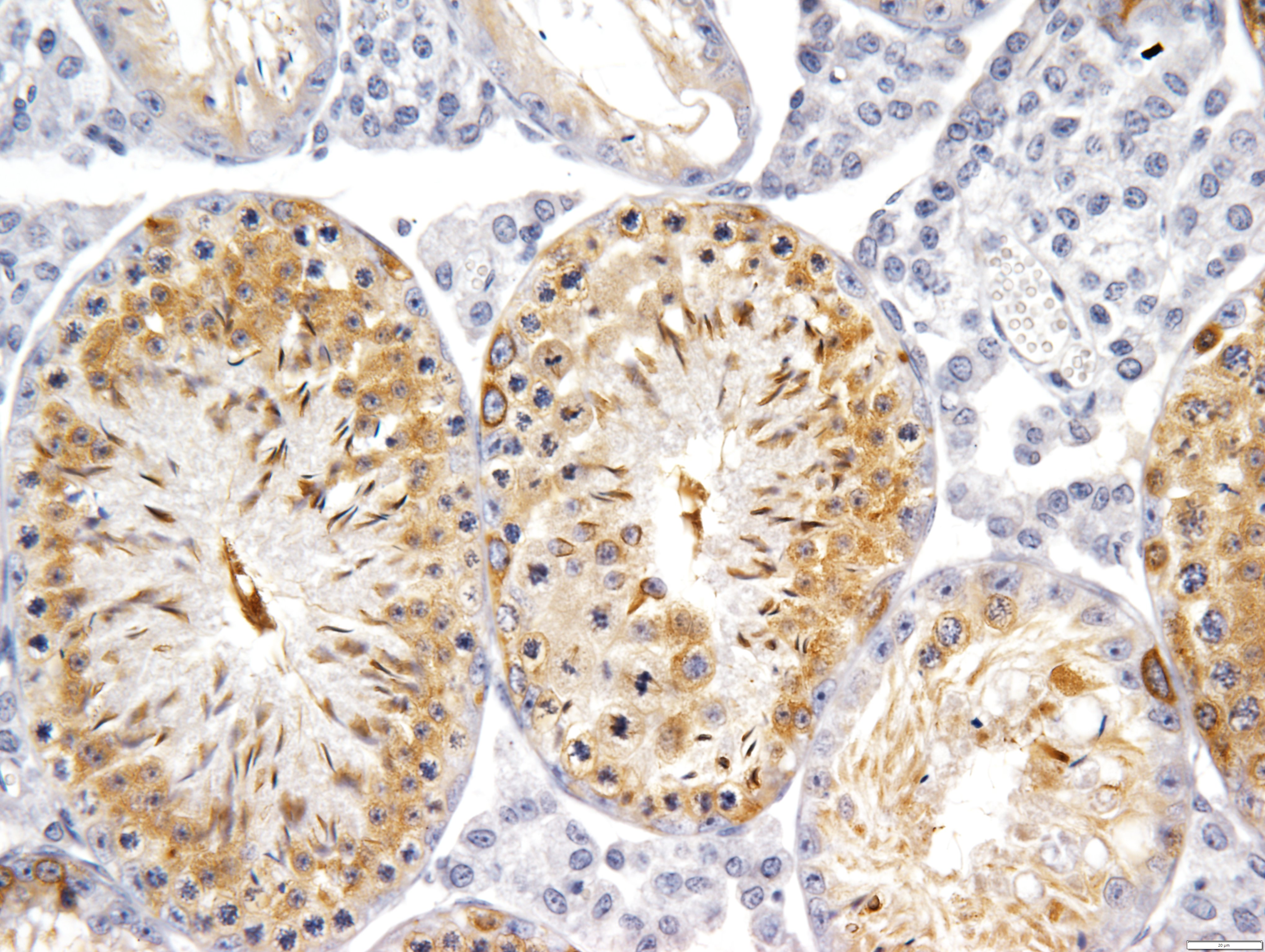

Supplement: Supplementary file 4 — Source data Fig. 4 [file 44319_2024_159_MOESM4_ESM.zip › EMBOR-2023-58207V1_SourceDataForFig4/4A/EMBOR-2023-58207V1_SourceDataForFig4a_GCKO_Stage XII.jpg]

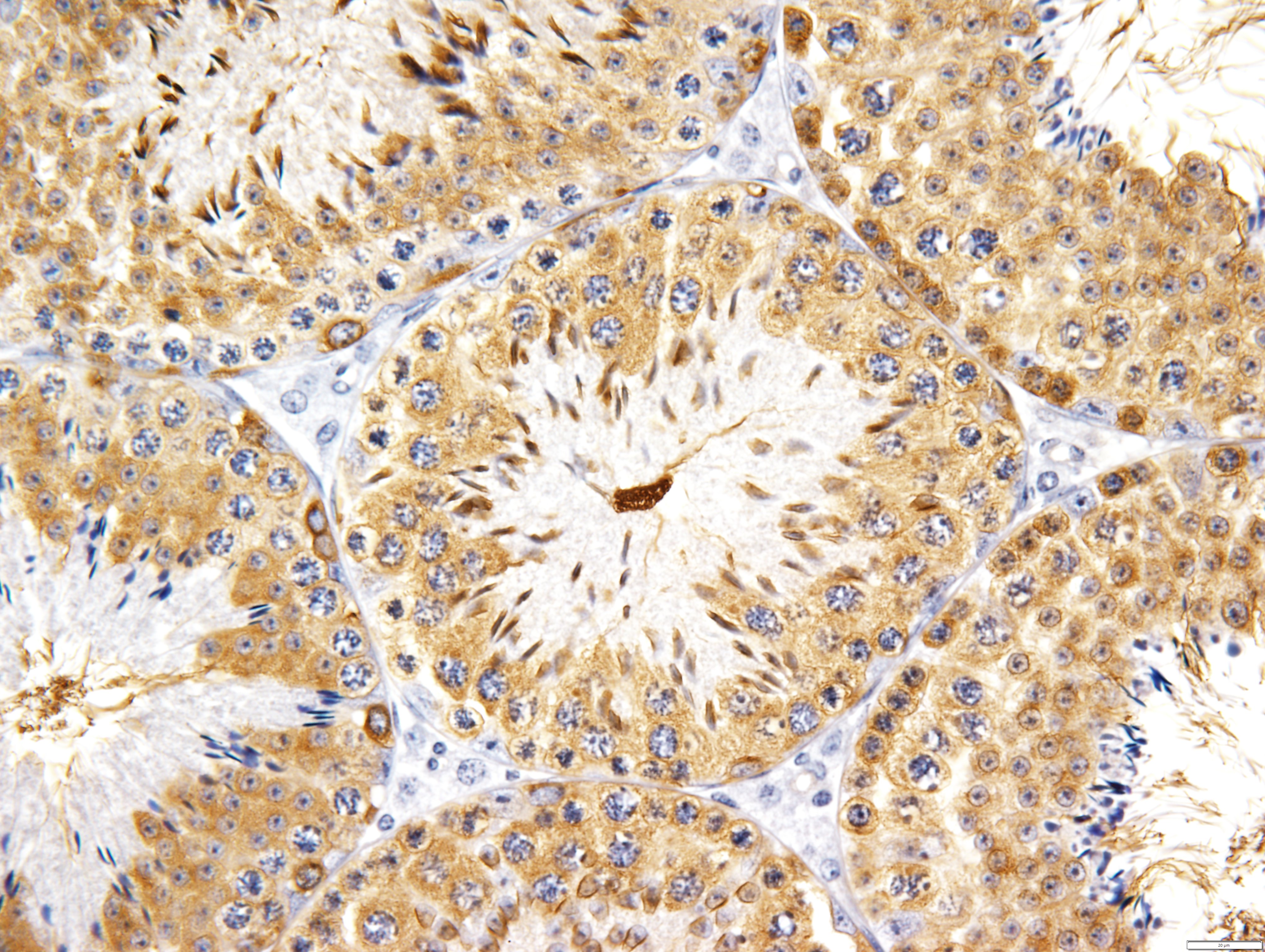

Supplement: Supplementary file 4 — Source data Fig. 4 [file 44319_2024_159_MOESM4_ESM.zip › EMBOR-2023-58207V1_SourceDataForFig4/4A/EMBOR-2023-58207V1_SourceDataForFig4a_Flox_Stage X.jpg]

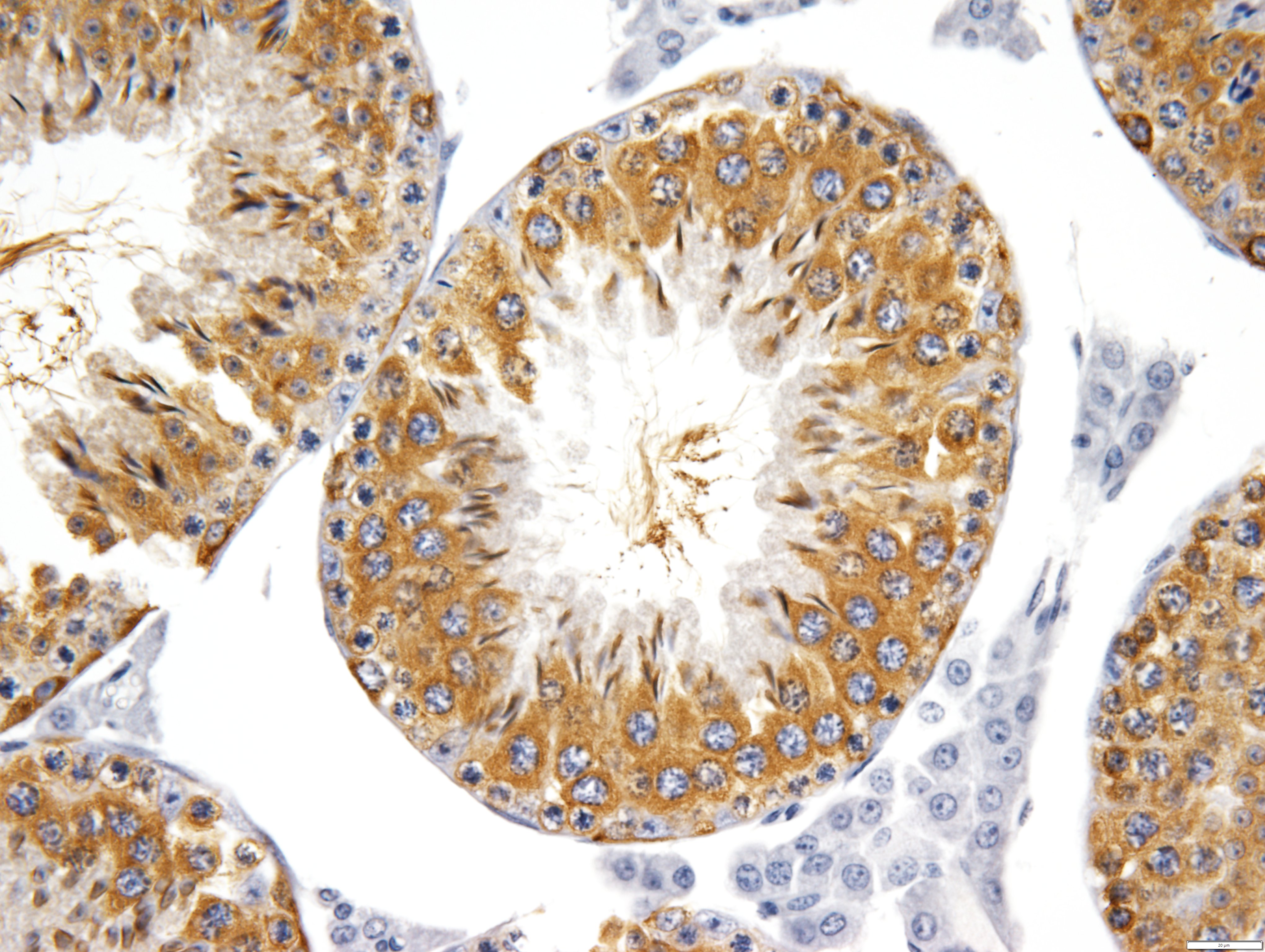

Supplement: Supplementary file 4 — Source data Fig. 4 [file 44319_2024_159_MOESM4_ESM.zip › EMBOR-2023-58207V1_SourceDataForFig4/4A/EMBOR-2023-58207V1_SourceDataForFig4a_Flox_Stage XI.jpg]

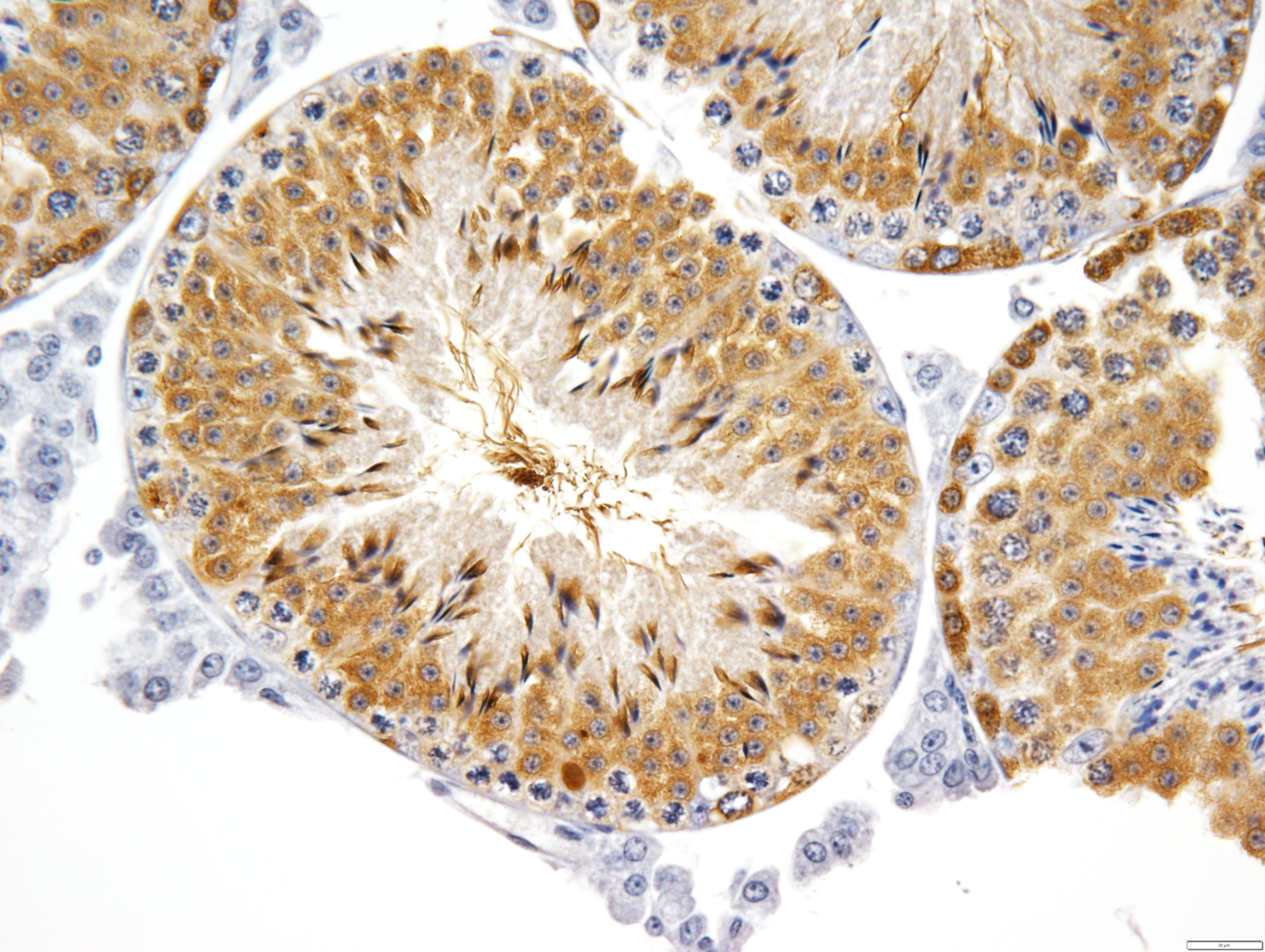

Supplement: Supplementary file 4 — Source data Fig. 4 [file 44319_2024_159_MOESM4_ESM.zip › EMBOR-2023-58207V1_SourceDataForFig4/4A/EMBOR-2023-58207V1_SourceDataForFig4a_Flox_Stage I.jpg]

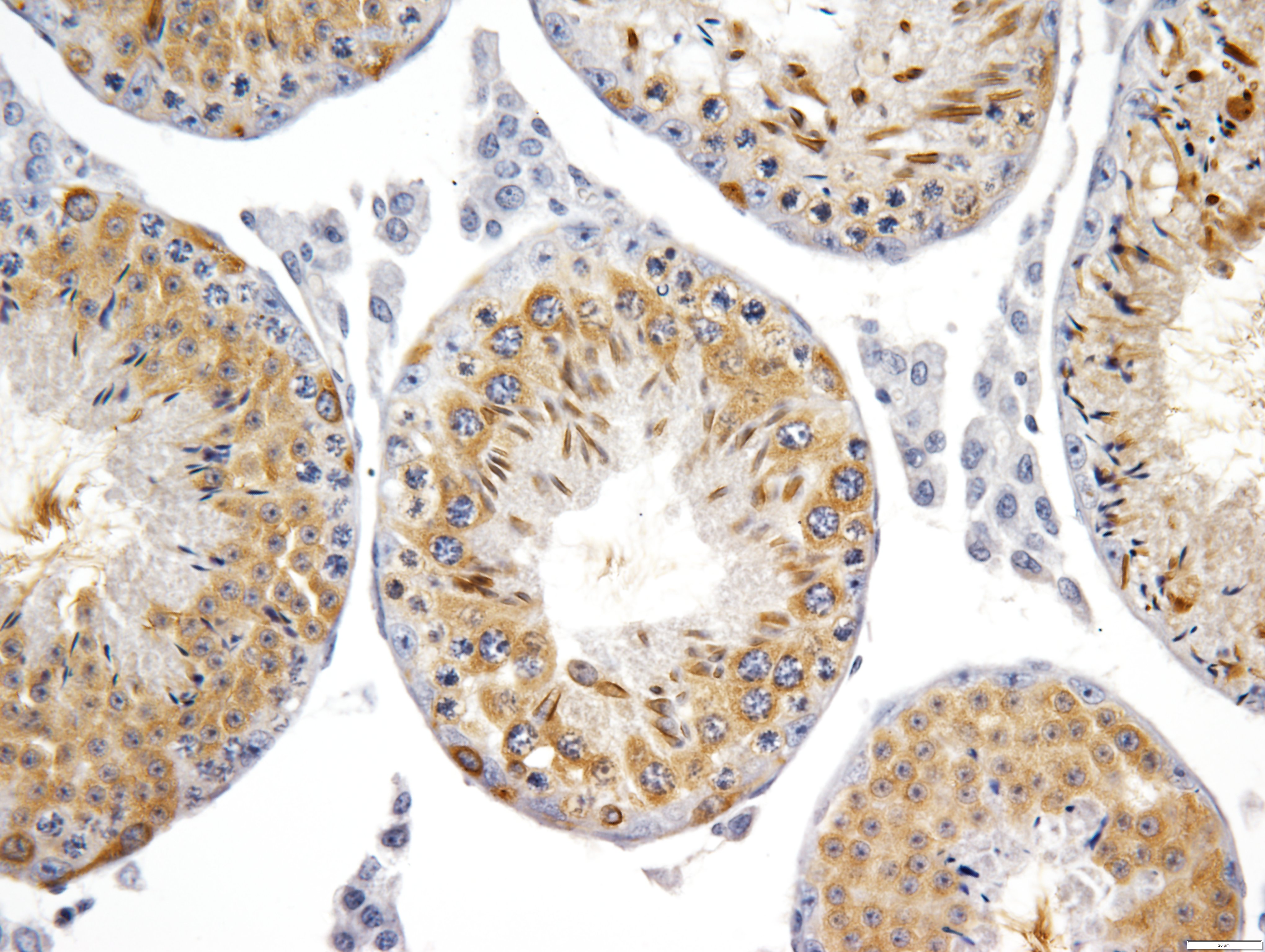

Supplement: Supplementary file 4 — Source data Fig. 4 [file 44319_2024_159_MOESM4_ESM.zip › EMBOR-2023-58207V1_SourceDataForFig4/4A/EMBOR-2023-58207V1_SourceDataForFig4a_GCKO_Stage X.jpg]

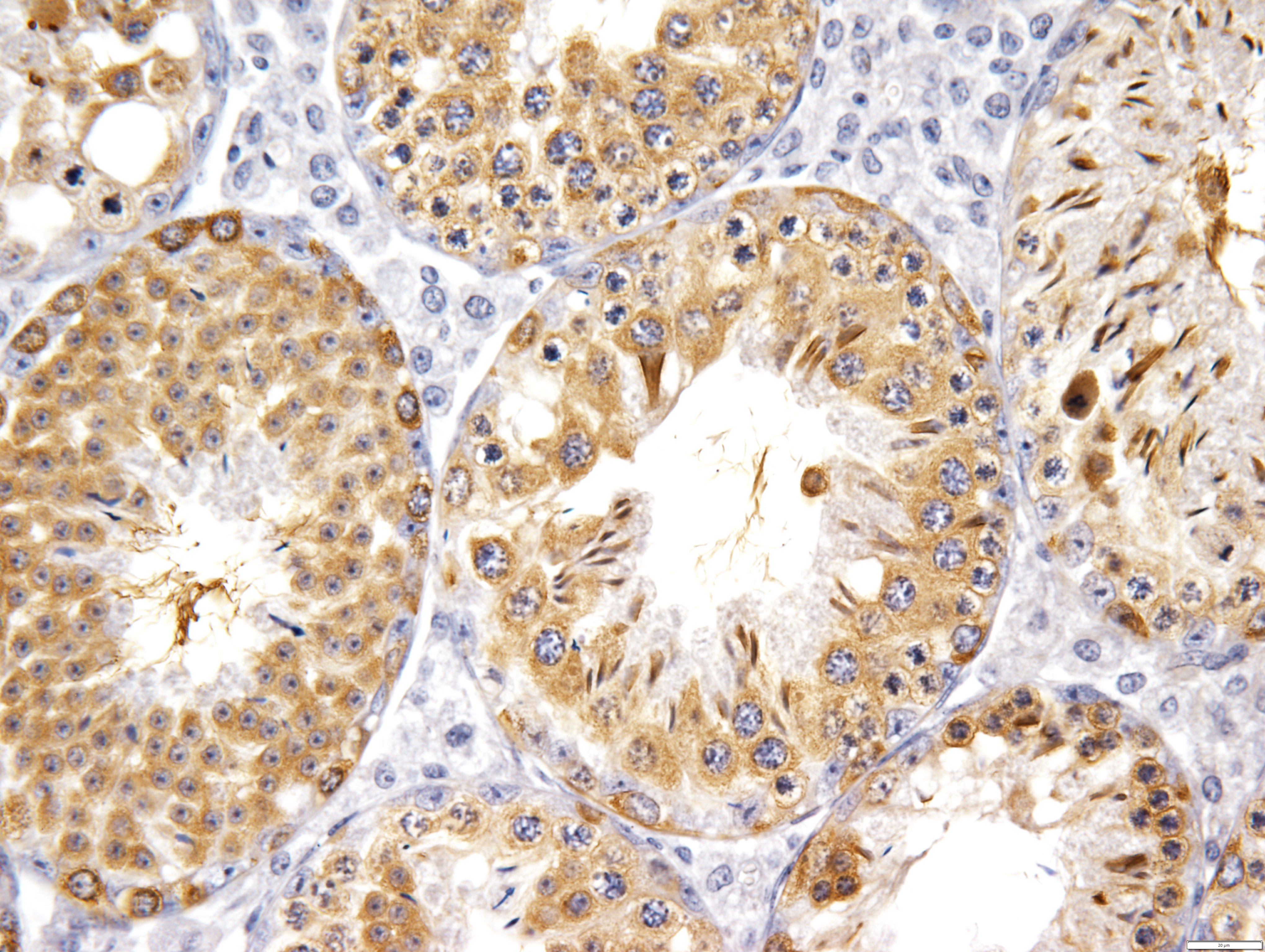

Supplement: Supplementary file 4 — Source data Fig. 4 [file 44319_2024_159_MOESM4_ESM.zip › EMBOR-2023-58207V1_SourceDataForFig4/4A/EMBOR-2023-58207V1_SourceDataForFig4a_GCKO_Stage XI.jpg]

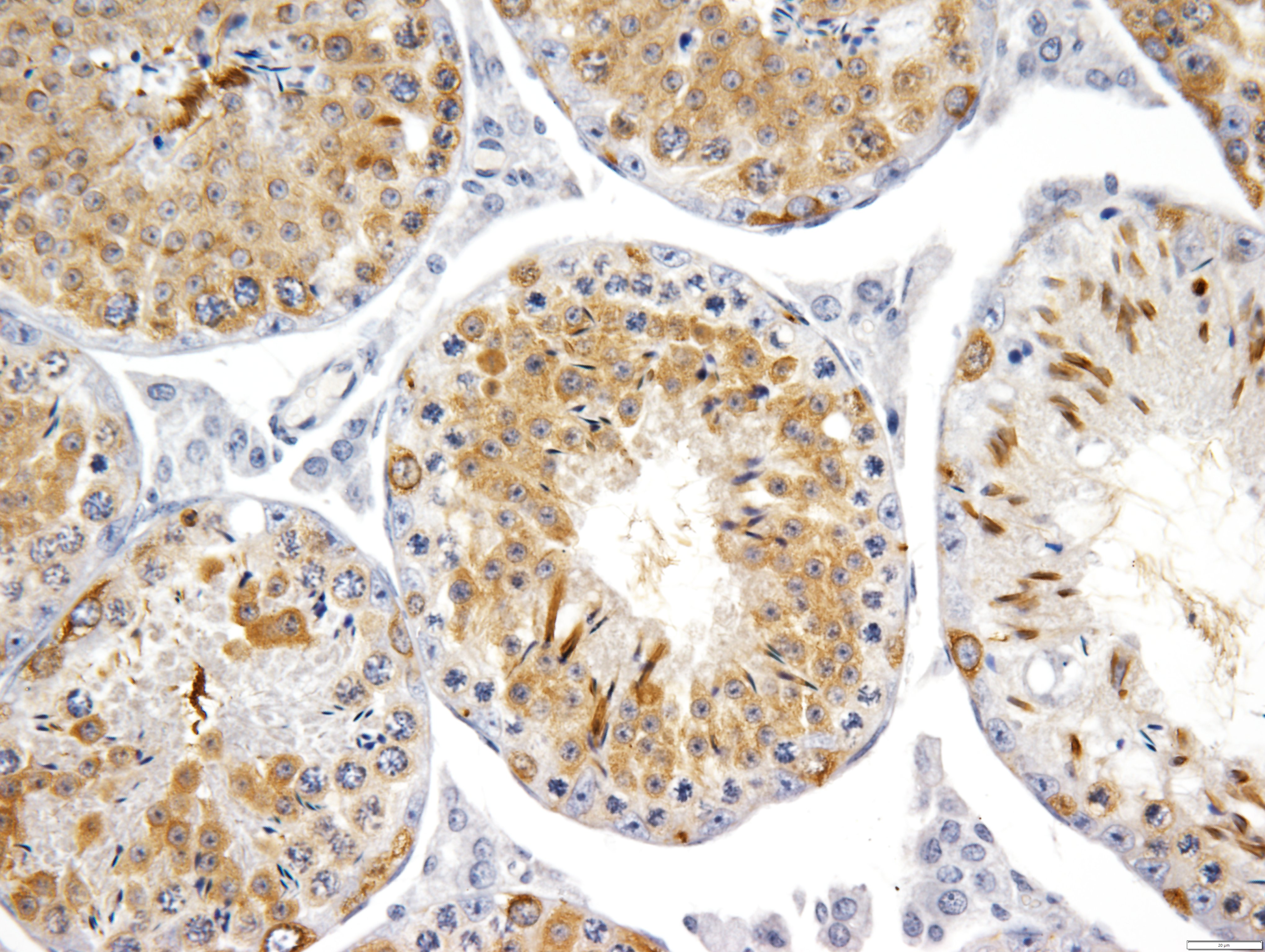

Supplement: Supplementary file 4 — Source data Fig. 4 [file 44319_2024_159_MOESM4_ESM.zip › EMBOR-2023-58207V1_SourceDataForFig4/4A/EMBOR-2023-58207V1_SourceDataForFig4a_GCKO_Stage I.jpg]

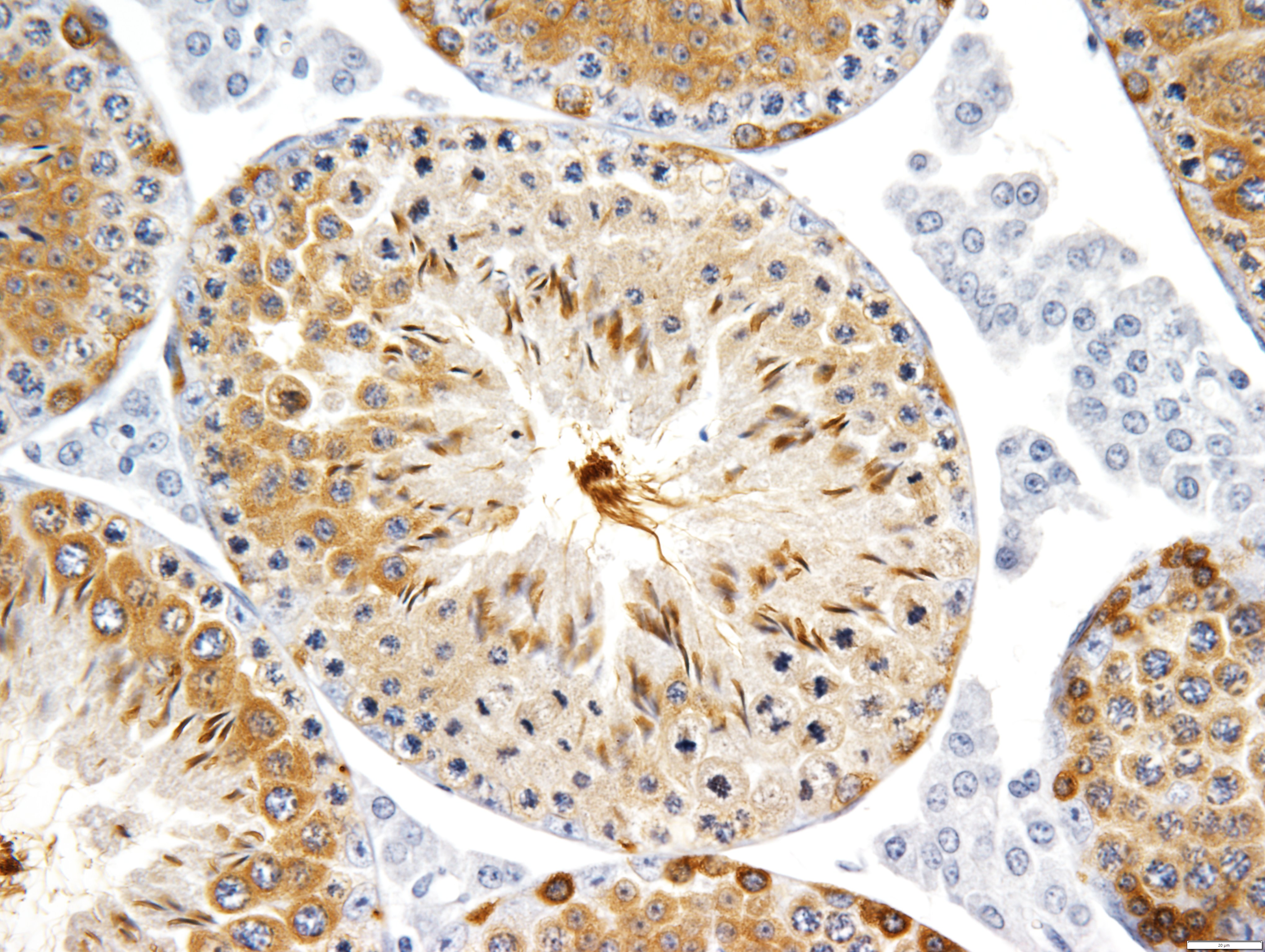

Supplement: Supplementary file 4 — Source data Fig. 4 [file 44319_2024_159_MOESM4_ESM.zip › EMBOR-2023-58207V1_SourceDataForFig4/4A/EMBOR-2023-58207V1_SourceDataForFig4a_Flox_Stage XII.jpg]

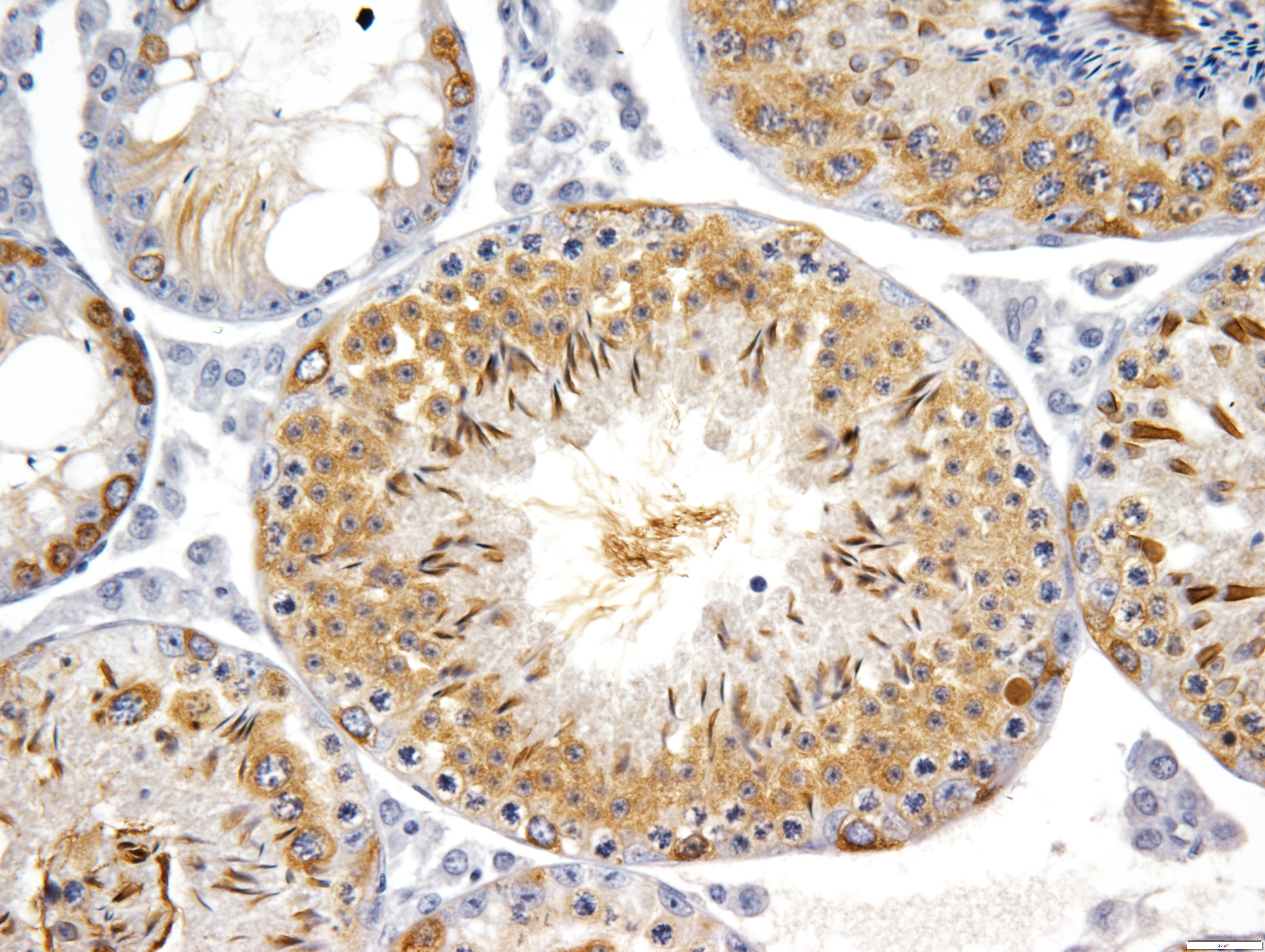

Supplement: Supplementary file 4 — Source data Fig. 4 [file 44319_2024_159_MOESM4_ESM.zip › EMBOR-2023-58207V1_SourceDataForFig4/4A/EMBOR-2023-58207V1_SourceDataForFig4a_GCKO_Stage II-III.jpg]

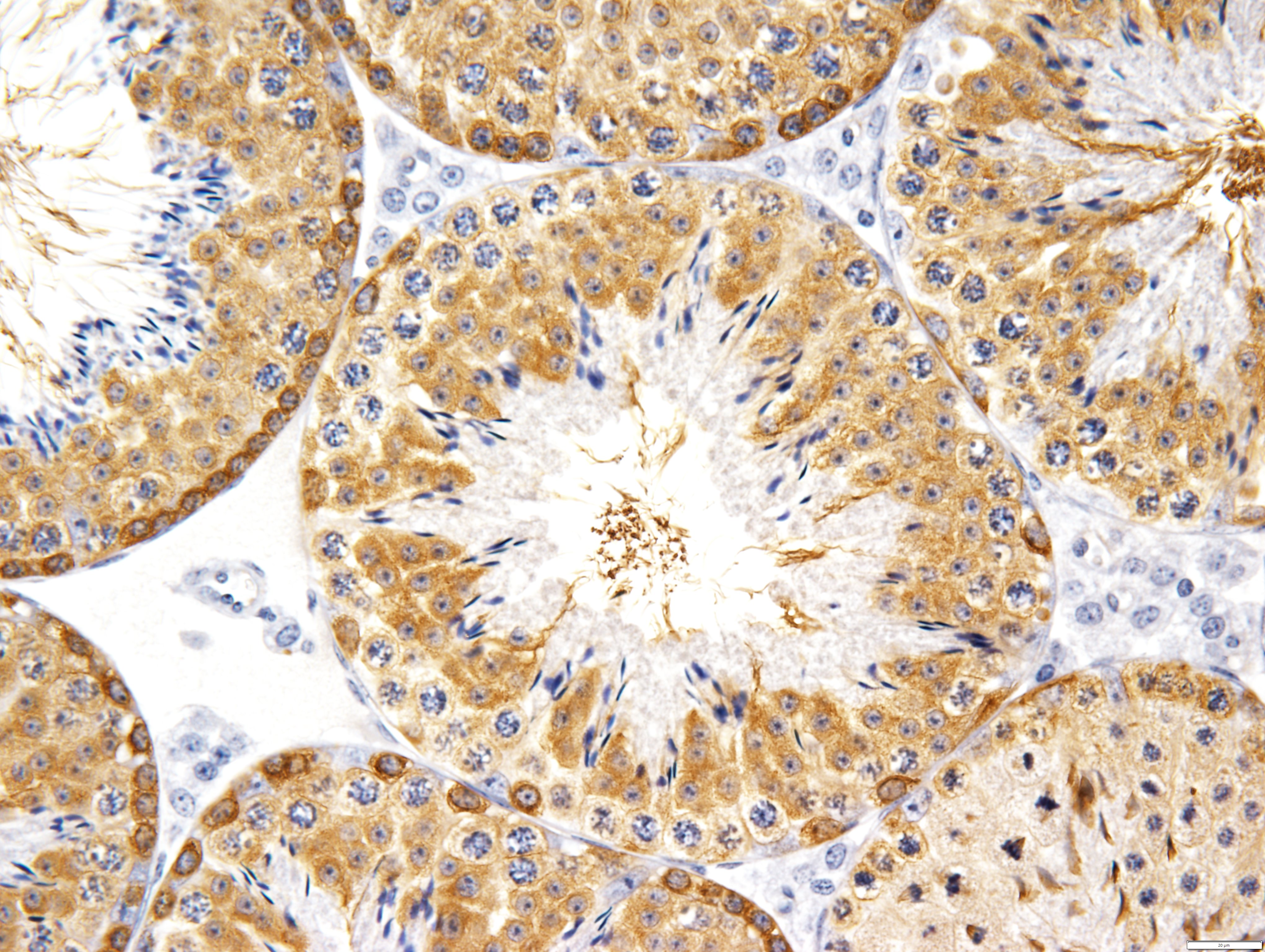

Supplement: Supplementary file 4 — Source data Fig. 4 [file 44319_2024_159_MOESM4_ESM.zip › EMBOR-2023-58207V1_SourceDataForFig4/4A/EMBOR-2023-58207V1_SourceDataForFig4a_Flox_Stage II-III.jpg]

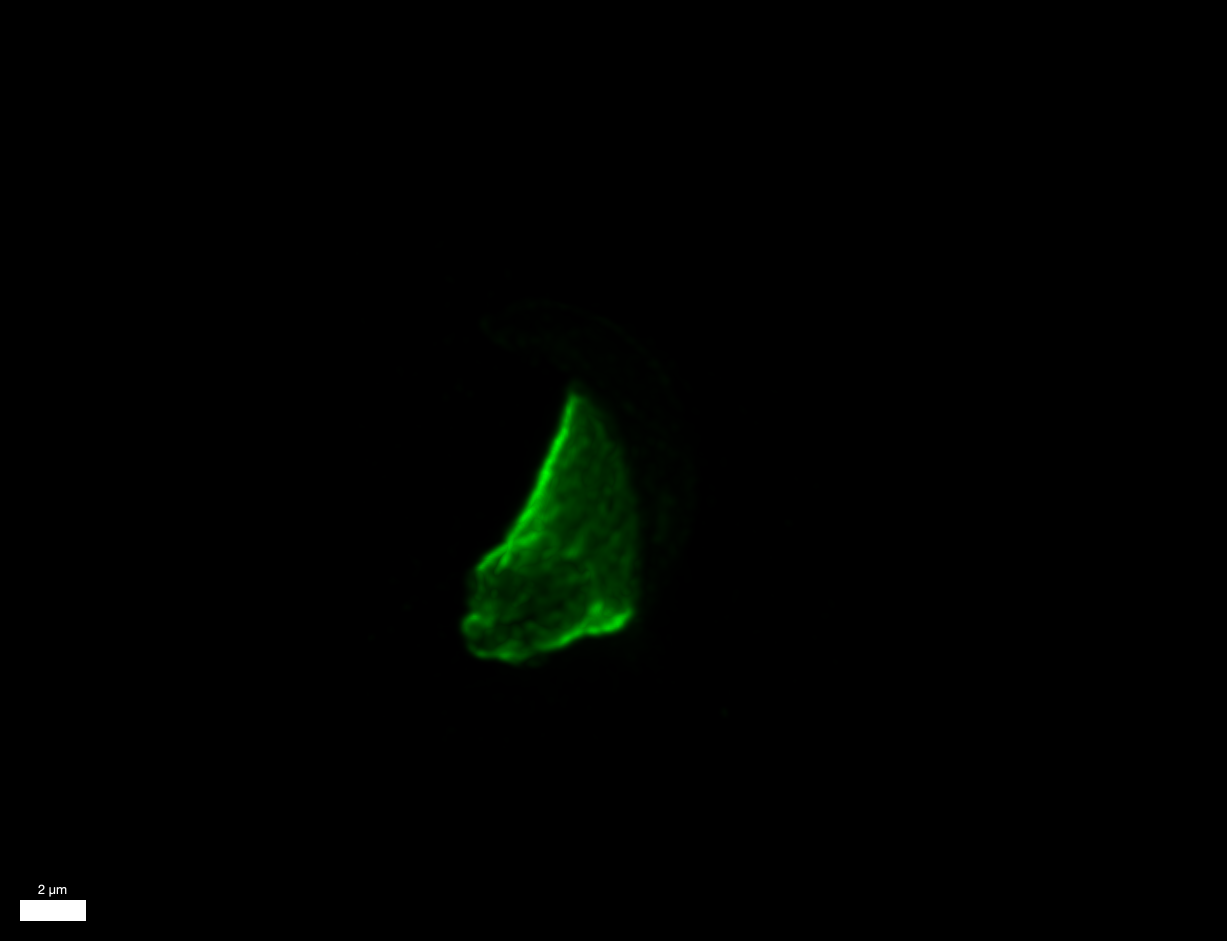

Supplement: Supplementary file 5 — Source data Fig. 5 [file 44319_2024_159_MOESM5_ESM.zip › EMBOR-2023-58207V1_SourceDataForFig5/5G/Tube1Flox:Flox/EMBOR-2023-58207V1_SourceDataForFig5GMid_alpha tubulin.png]

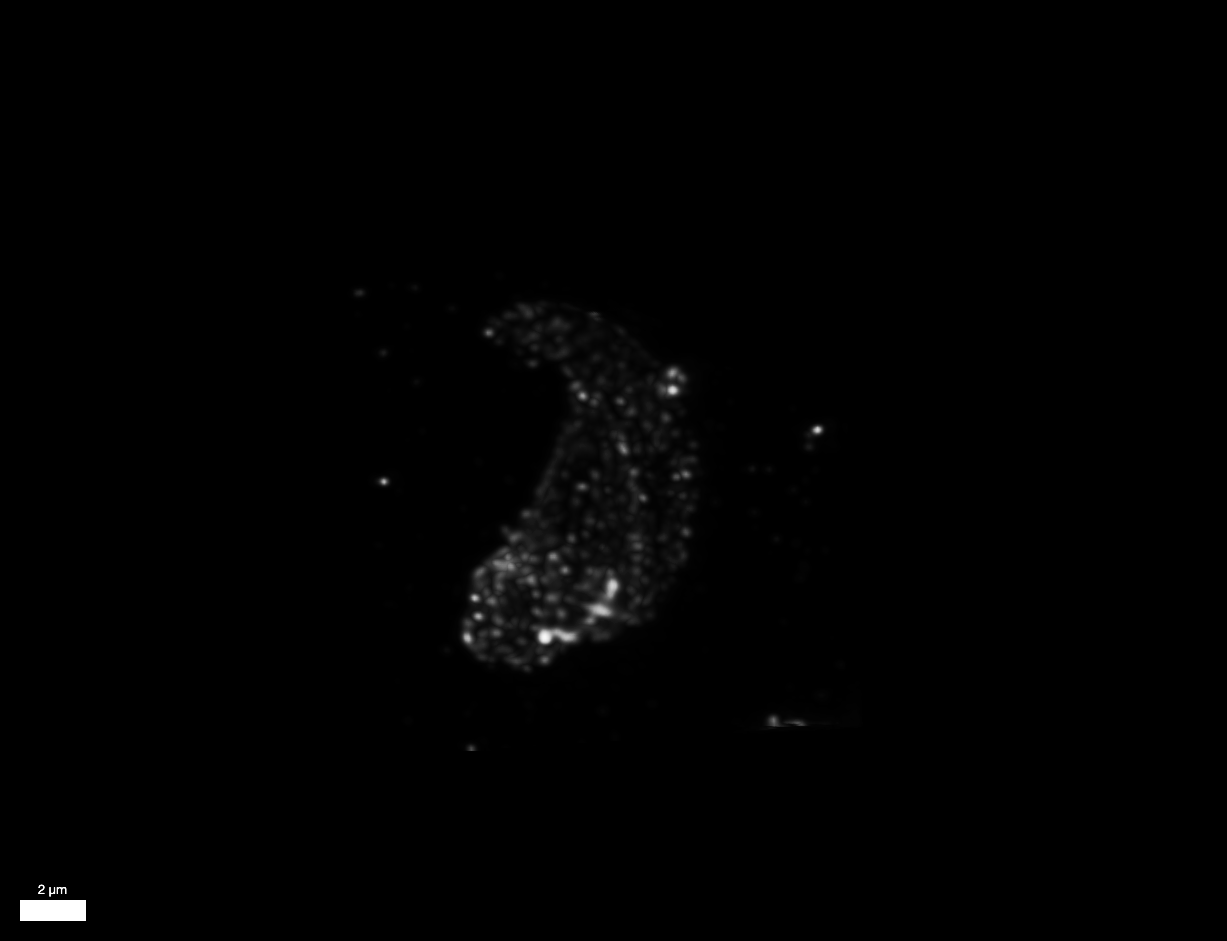

Supplement: Supplementary file 5 — Source data Fig. 5 [file 44319_2024_159_MOESM5_ESM.zip › EMBOR-2023-58207V1_SourceDataForFig5/5G/Tube1Flox:Flox/EMBOR-2023-58207V1_SourceDataForFig5GMid_KATNB1.png]

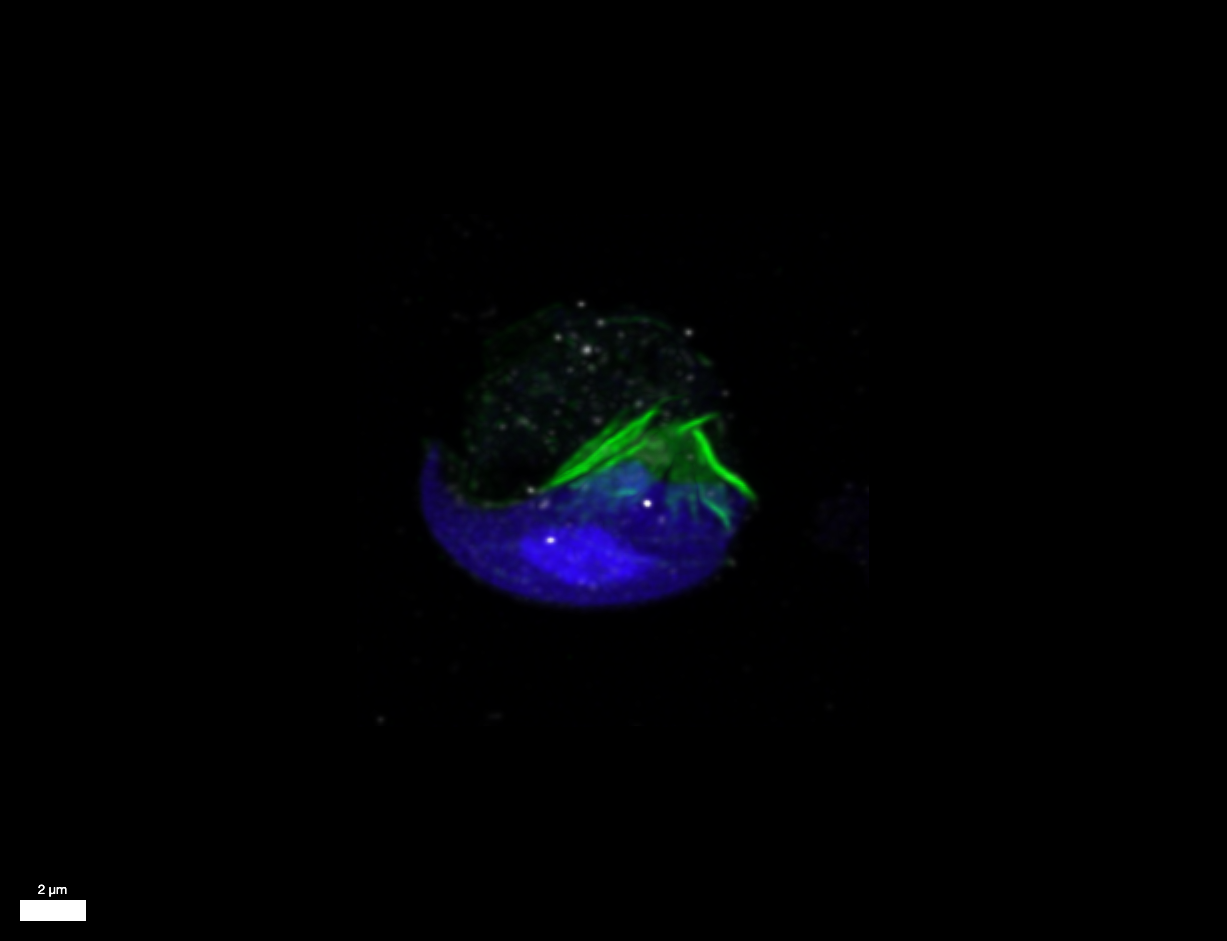

Supplement: Supplementary file 5 — Source data Fig. 5 [file 44319_2024_159_MOESM5_ESM.zip › EMBOR-2023-58207V1_SourceDataForFig5/5G/Tube1Flox:Flox/EMBOR-2023-58207V1_SourceDataForFig5GLate_DAPI:Merged.tif]

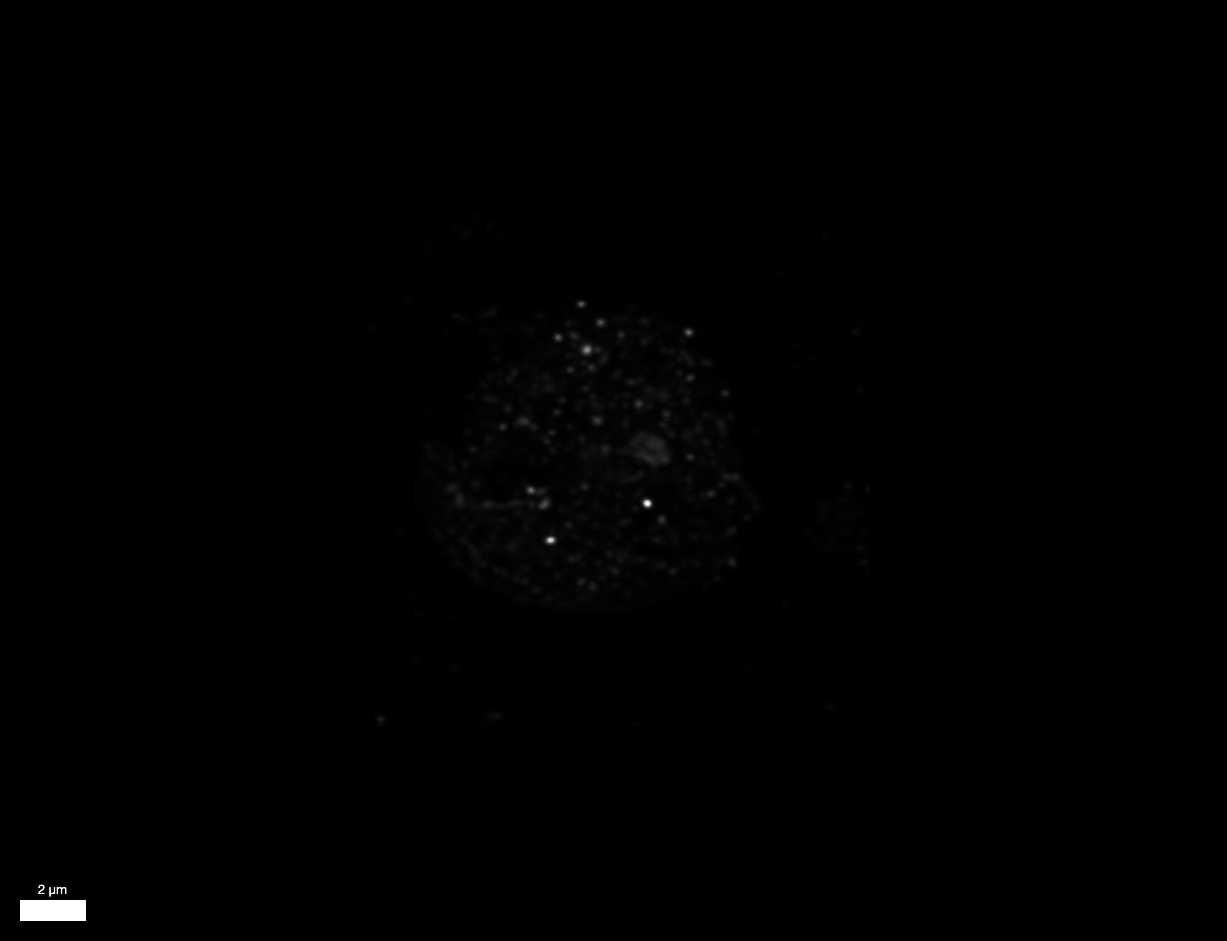

Supplement: Supplementary file 5 — Source data Fig. 5 [file 44319_2024_159_MOESM5_ESM.zip › EMBOR-2023-58207V1_SourceDataForFig5/5G/Tube1Flox:Flox/EMBOR-2023-58207V1_SourceDataForFig5GLate_KATNB1.png]

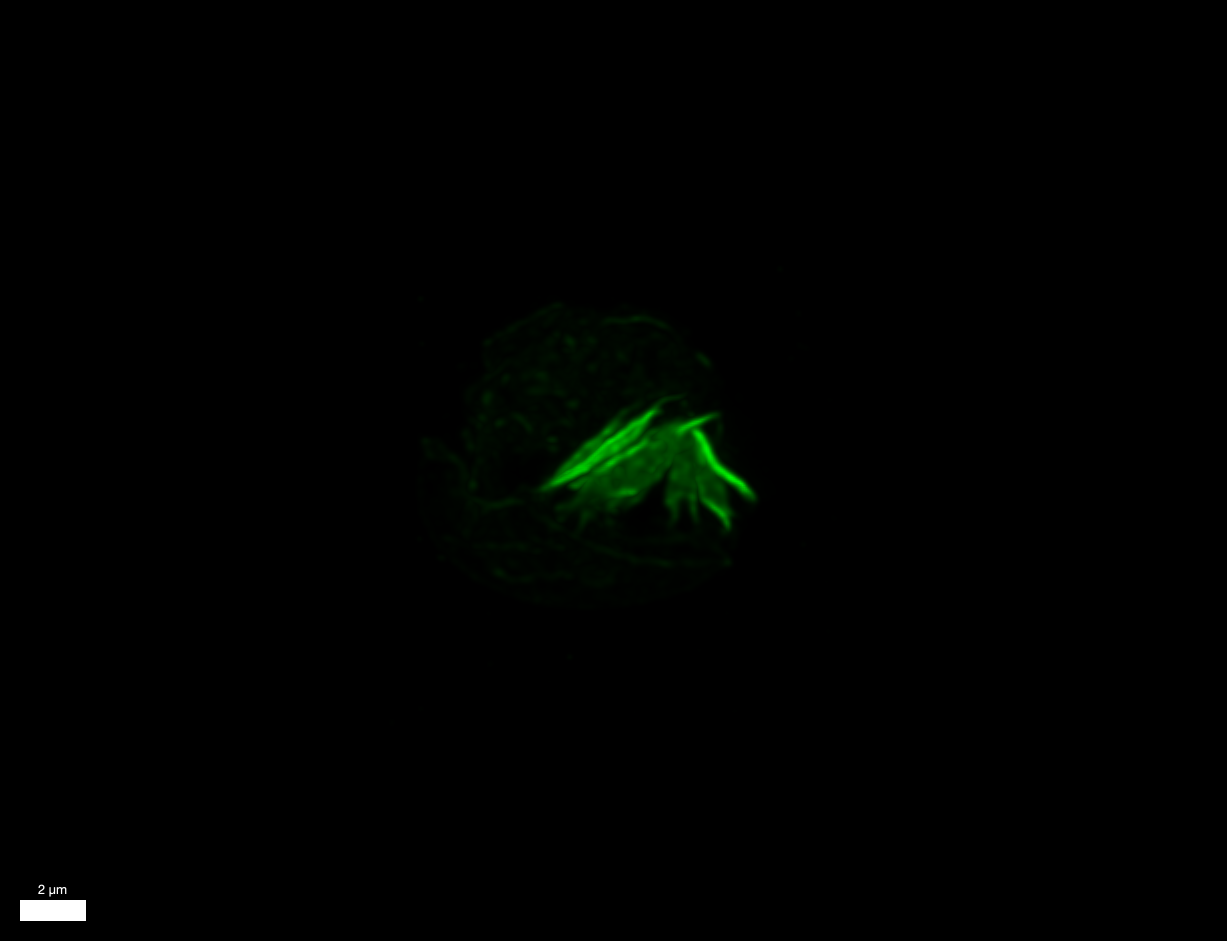

Supplement: Supplementary file 5 — Source data Fig. 5 [file 44319_2024_159_MOESM5_ESM.zip › EMBOR-2023-58207V1_SourceDataForFig5/5G/Tube1Flox:Flox/EMBOR-2023-58207V1_SourceDataForFig5GLate_alpha tubulin.png]

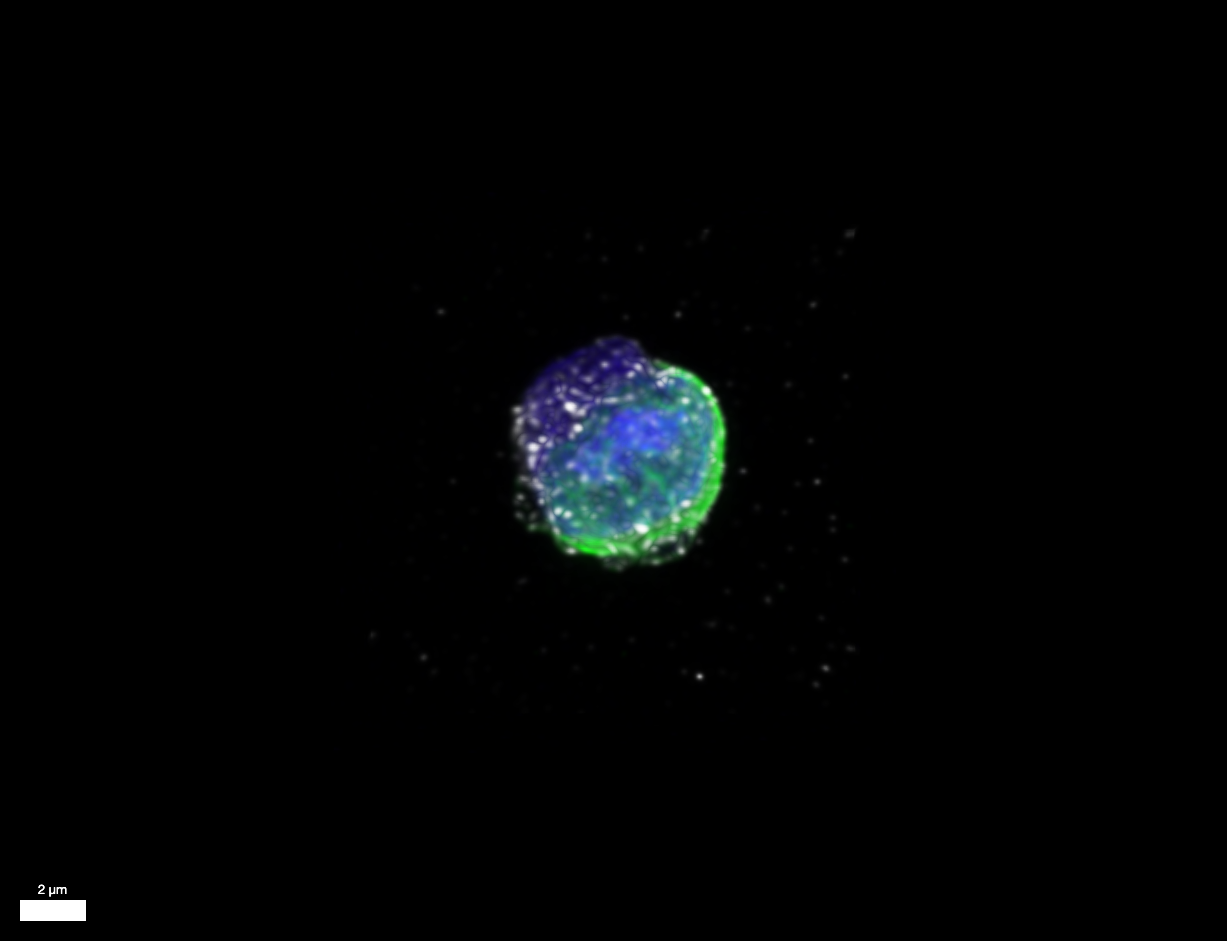

Supplement: Supplementary file 5 — Source data Fig. 5 [file 44319_2024_159_MOESM5_ESM.zip › EMBOR-2023-58207V1_SourceDataForFig5/5G/Tube1Flox:Flox/EMBOR-2023-58207V1_SourceDataForFig5GEarly_DAPI:Merged.tif]

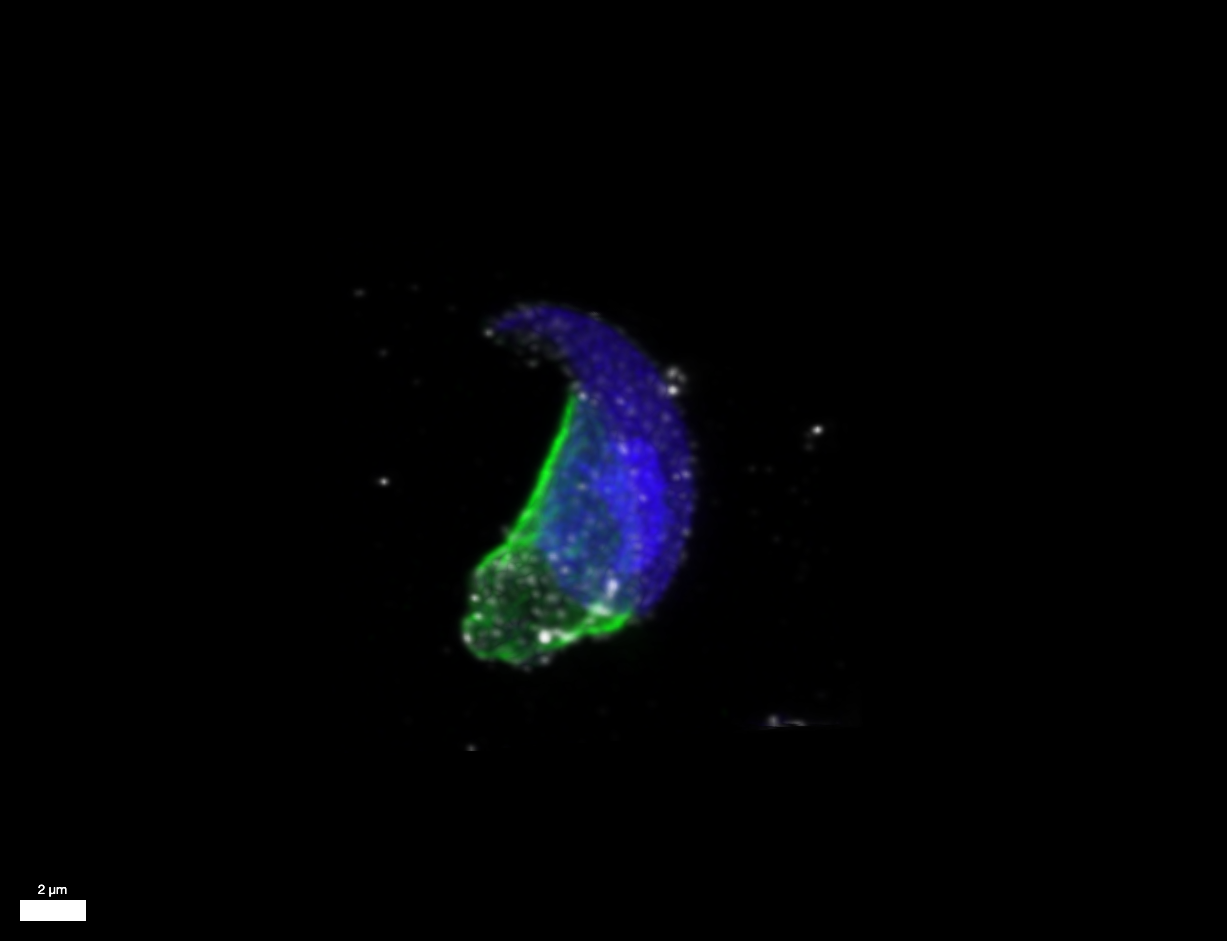

Supplement: Supplementary file 5 — Source data Fig. 5 [file 44319_2024_159_MOESM5_ESM.zip › EMBOR-2023-58207V1_SourceDataForFig5/5G/Tube1Flox:Flox/EMBOR-2023-58207V1_SourceDataForFig5GMid_DAPI:Merged.tif]

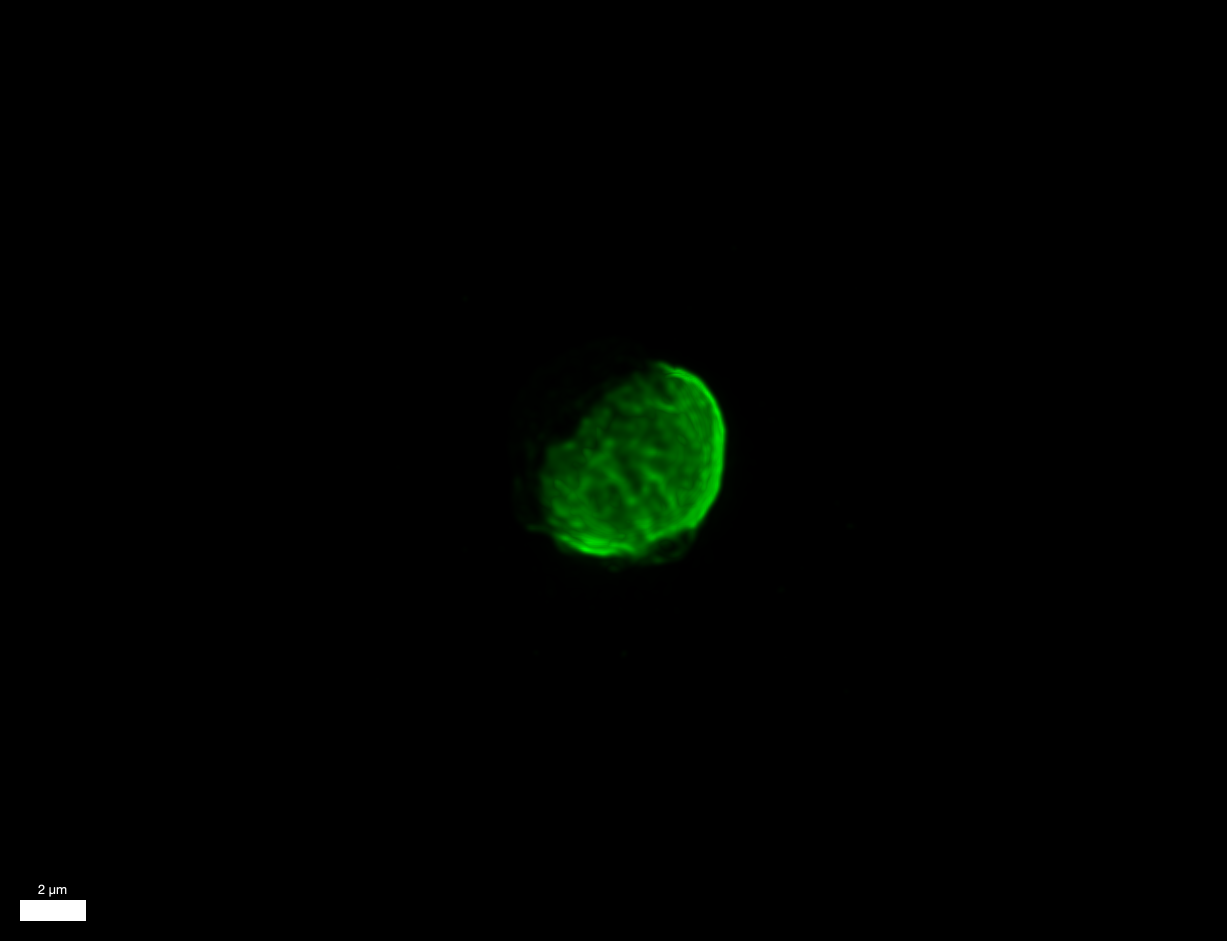

Supplement: Supplementary file 5 — Source data Fig. 5 [file 44319_2024_159_MOESM5_ESM.zip › EMBOR-2023-58207V1_SourceDataForFig5/5G/Tube1Flox:Flox/EMBOR-2023-58207V1_SourceDataForFig5GEarly_alpha tubulin.png]

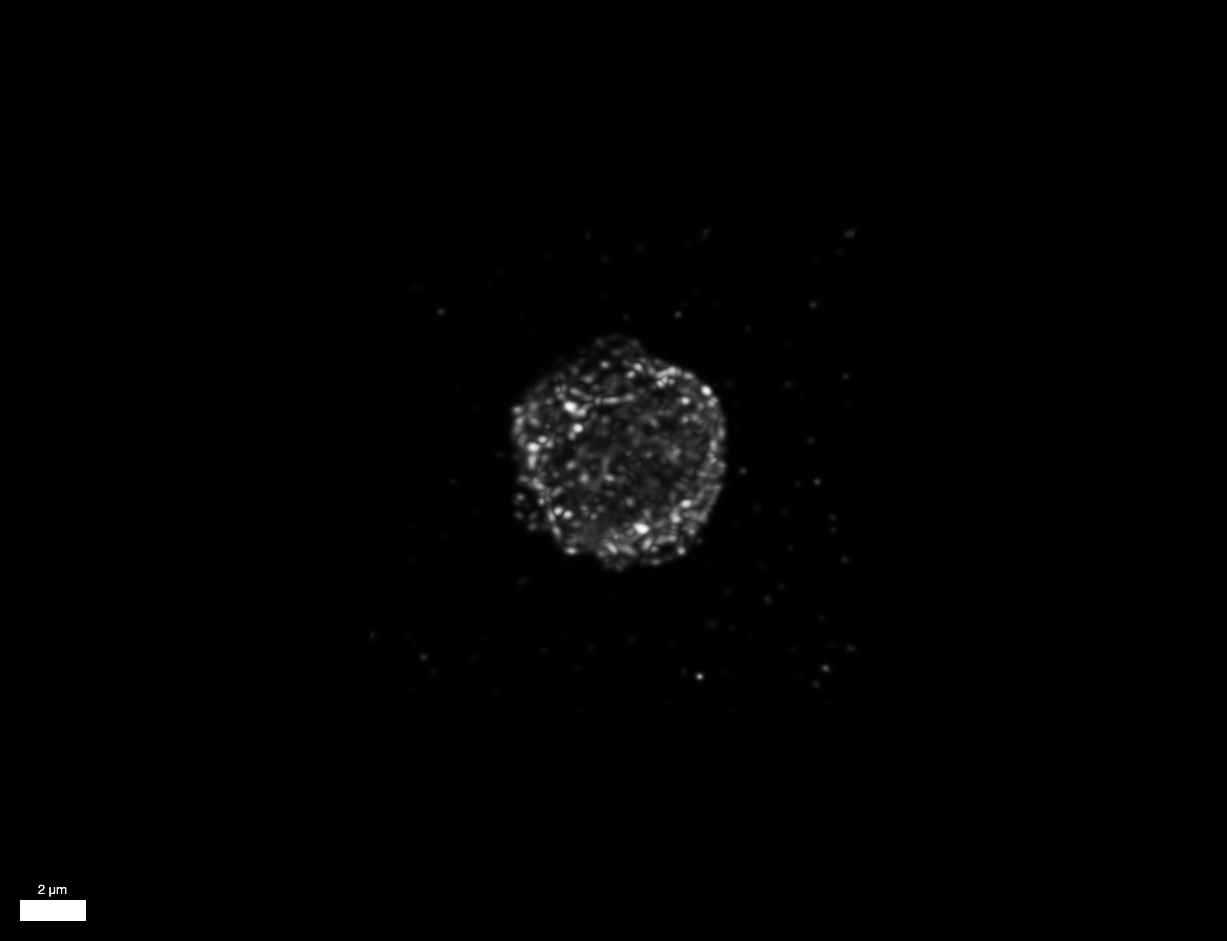

Supplement: Supplementary file 5 — Source data Fig. 5 [file 44319_2024_159_MOESM5_ESM.zip › EMBOR-2023-58207V1_SourceDataForFig5/5G/Tube1Flox:Flox/EMBOR-2023-58207V1_SourceDataForFig5GEarly_KATNB1.png]

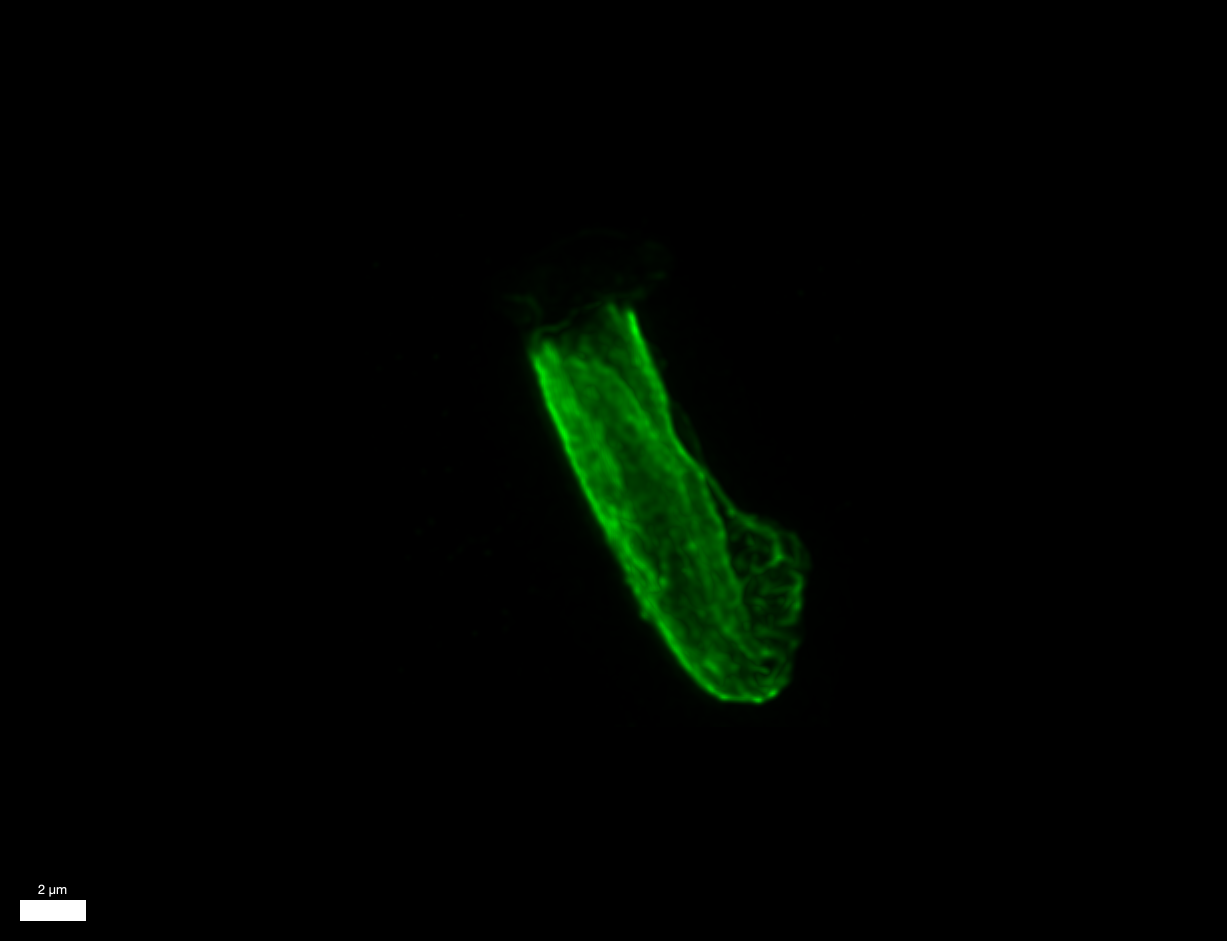

Supplement: Supplementary file 5 — Source data Fig. 5 [file 44319_2024_159_MOESM5_ESM.zip › EMBOR-2023-58207V1_SourceDataForFig5/5G/Tube1GCKO:GCKO/EMBOR-2023-58207V1_SourceDataForFig5GMid_alpha tubulin.png]

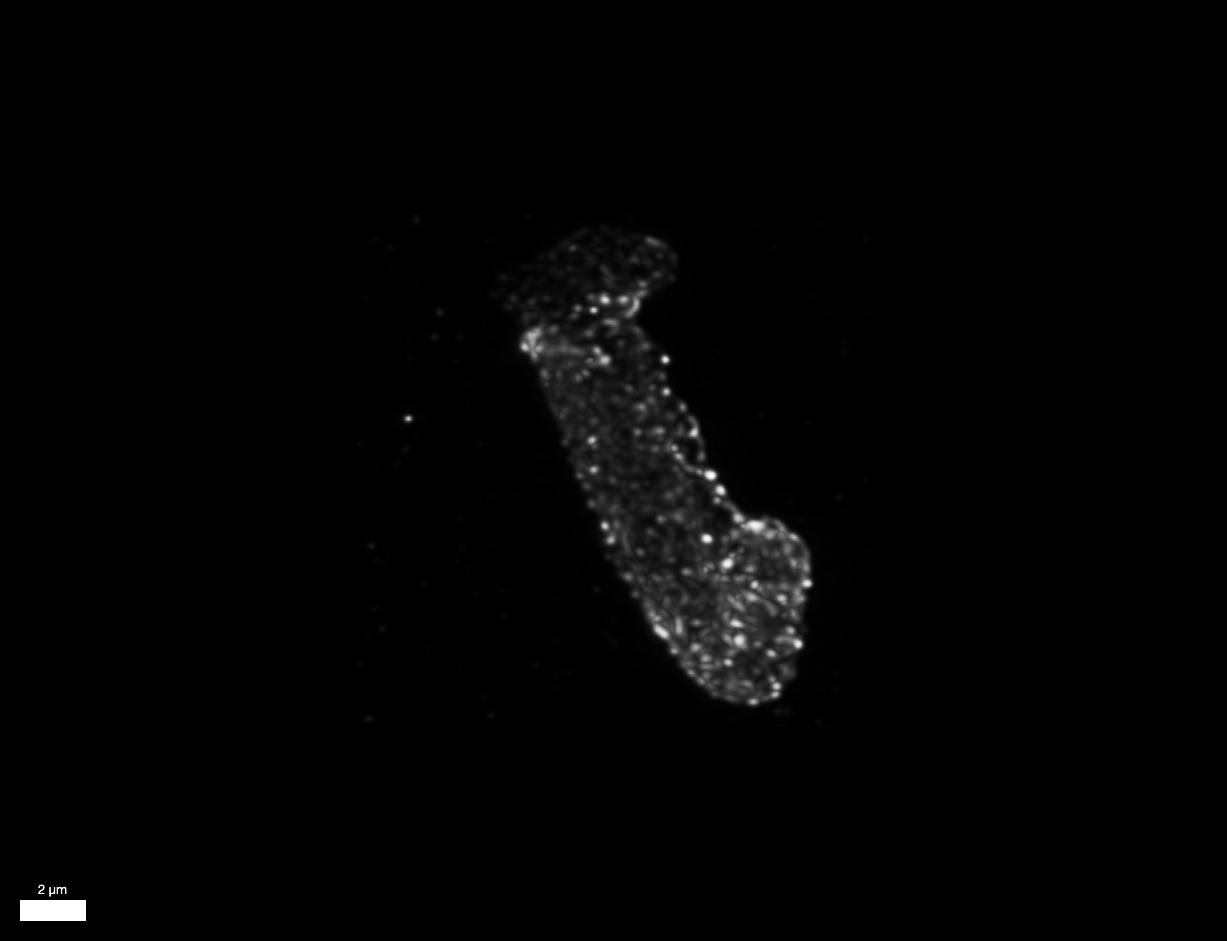

Supplement: Supplementary file 5 — Source data Fig. 5 [file 44319_2024_159_MOESM5_ESM.zip › EMBOR-2023-58207V1_SourceDataForFig5/5G/Tube1GCKO:GCKO/EMBOR-2023-58207V1_SourceDataForFig5GMid_KATNB1.png]

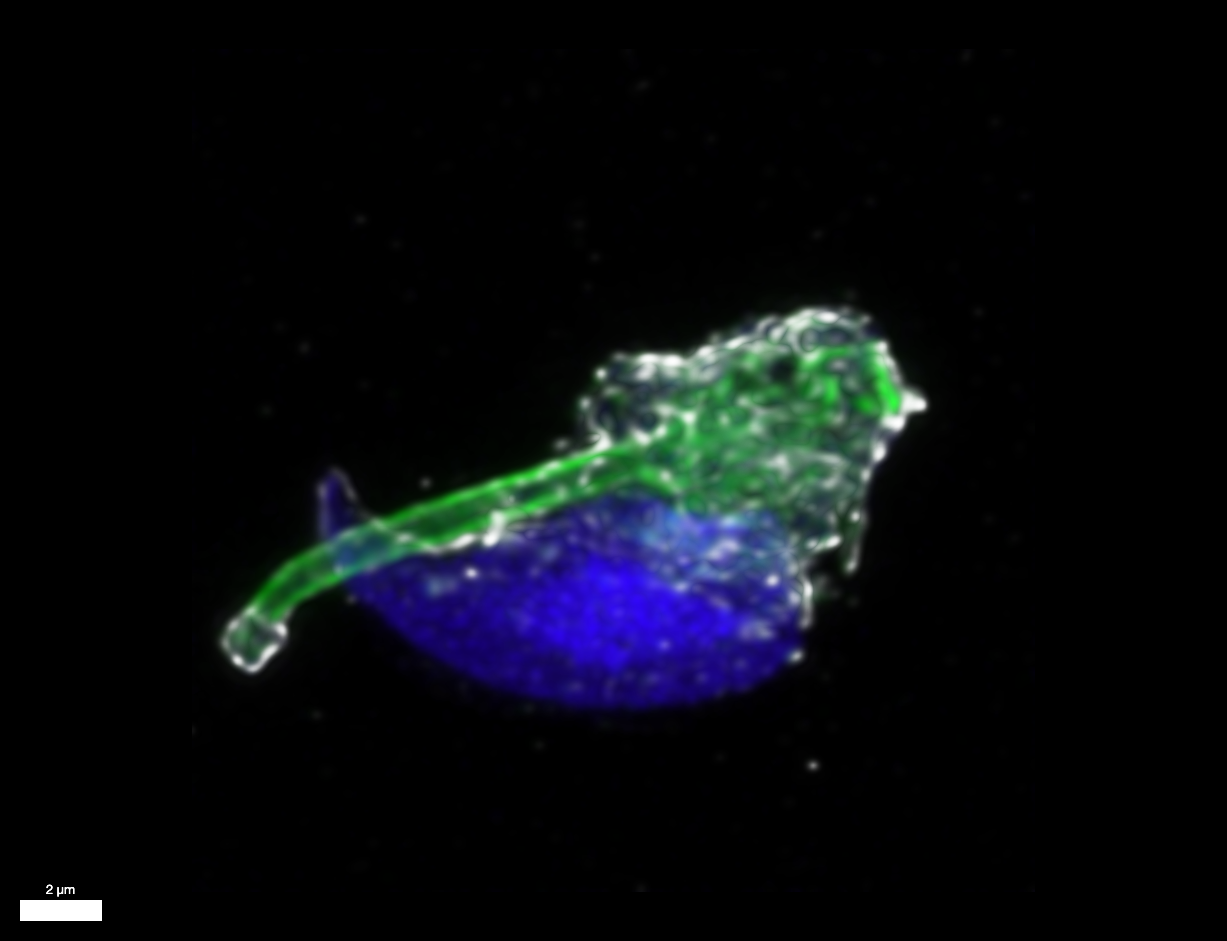

Supplement: Supplementary file 5 — Source data Fig. 5 [file 44319_2024_159_MOESM5_ESM.zip › EMBOR-2023-58207V1_SourceDataForFig5/5G/Tube1GCKO:GCKO/EMBOR-2023-58207V1_SourceDataForFig5GLate_DAPI:Merged.tif]

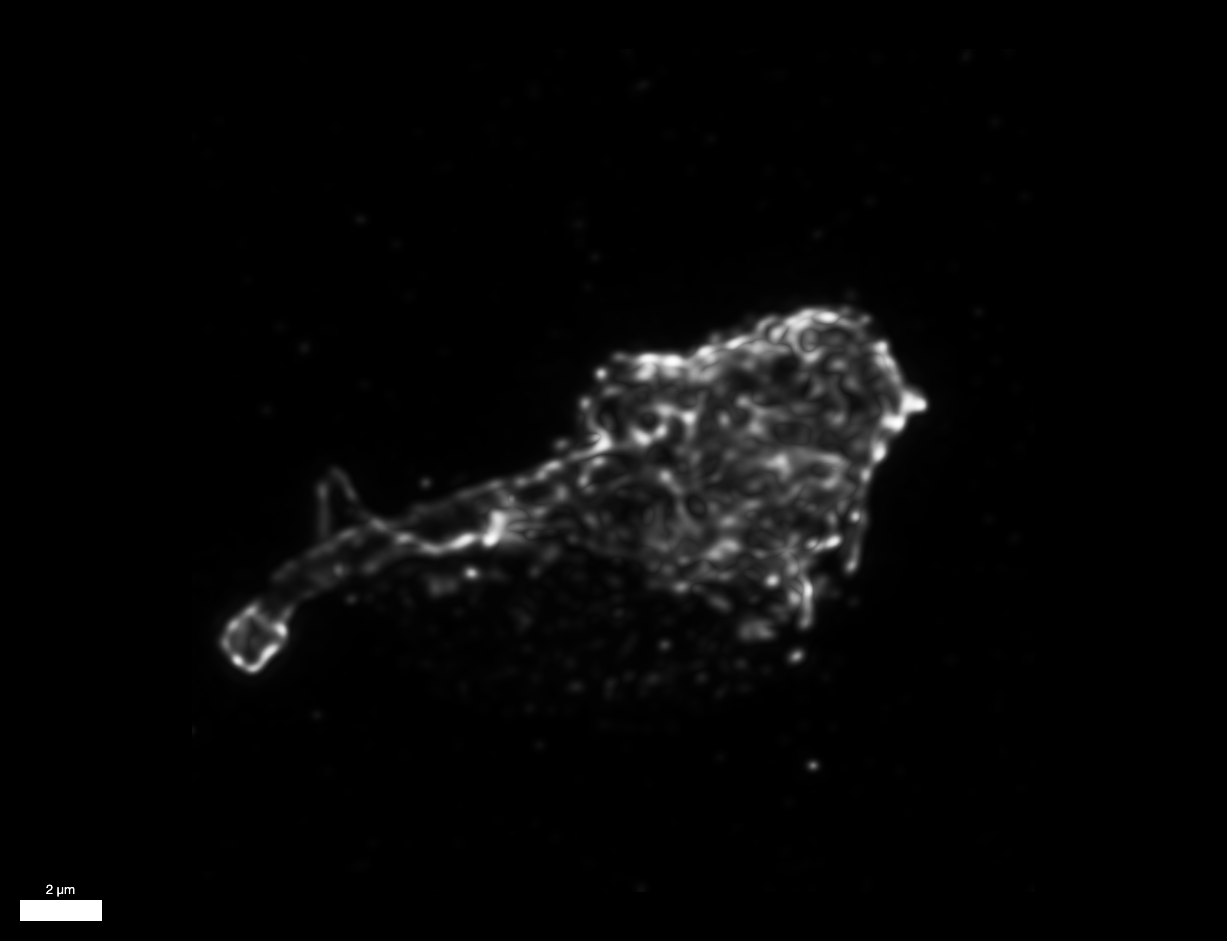

Supplement: Supplementary file 5 — Source data Fig. 5 [file 44319_2024_159_MOESM5_ESM.zip › EMBOR-2023-58207V1_SourceDataForFig5/5G/Tube1GCKO:GCKO/EMBOR-2023-58207V1_SourceDataForFig5GLate_KATNB1.png]

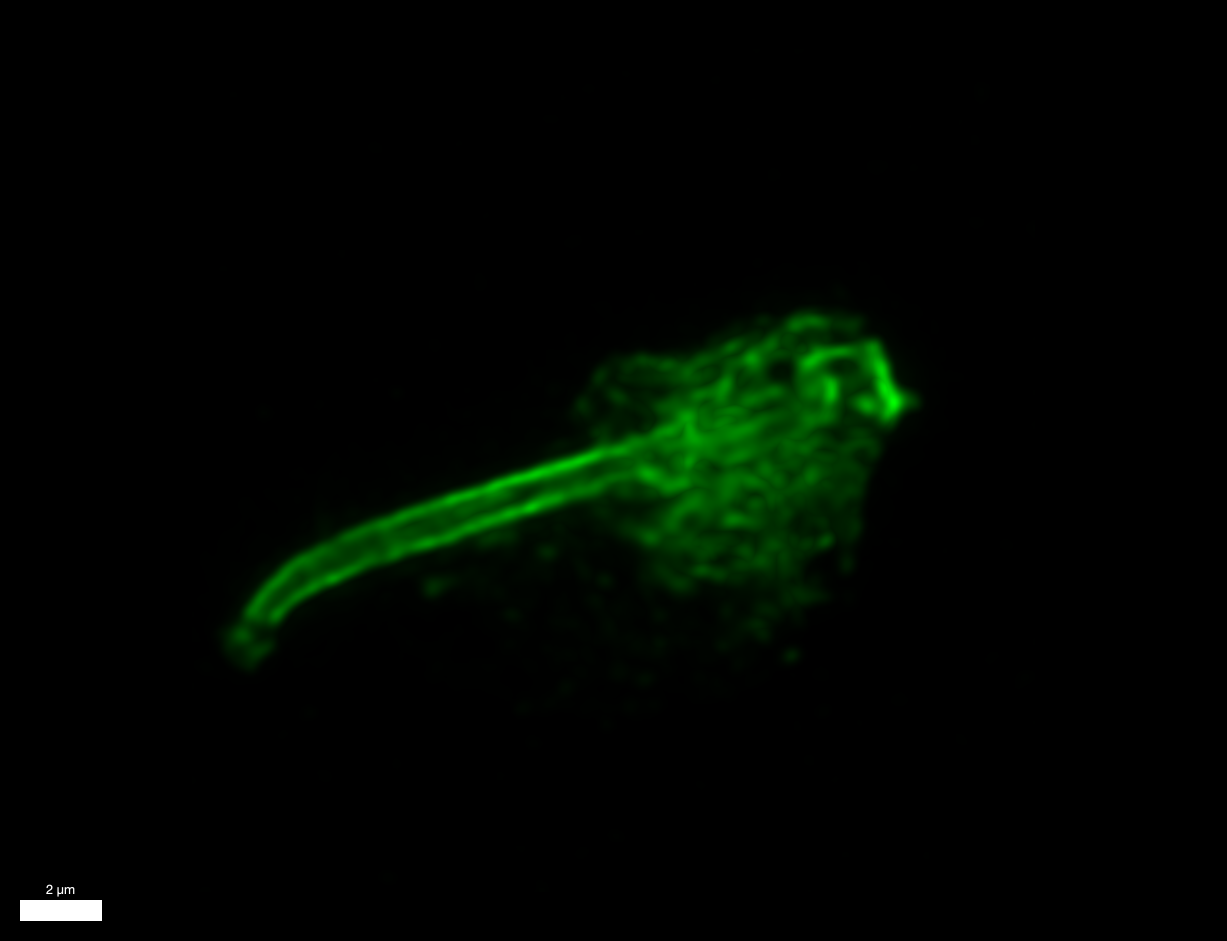

Supplement: Supplementary file 5 — Source data Fig. 5 [file 44319_2024_159_MOESM5_ESM.zip › EMBOR-2023-58207V1_SourceDataForFig5/5G/Tube1GCKO:GCKO/EMBOR-2023-58207V1_SourceDataForFig5GLate_alpha tubulin.png]

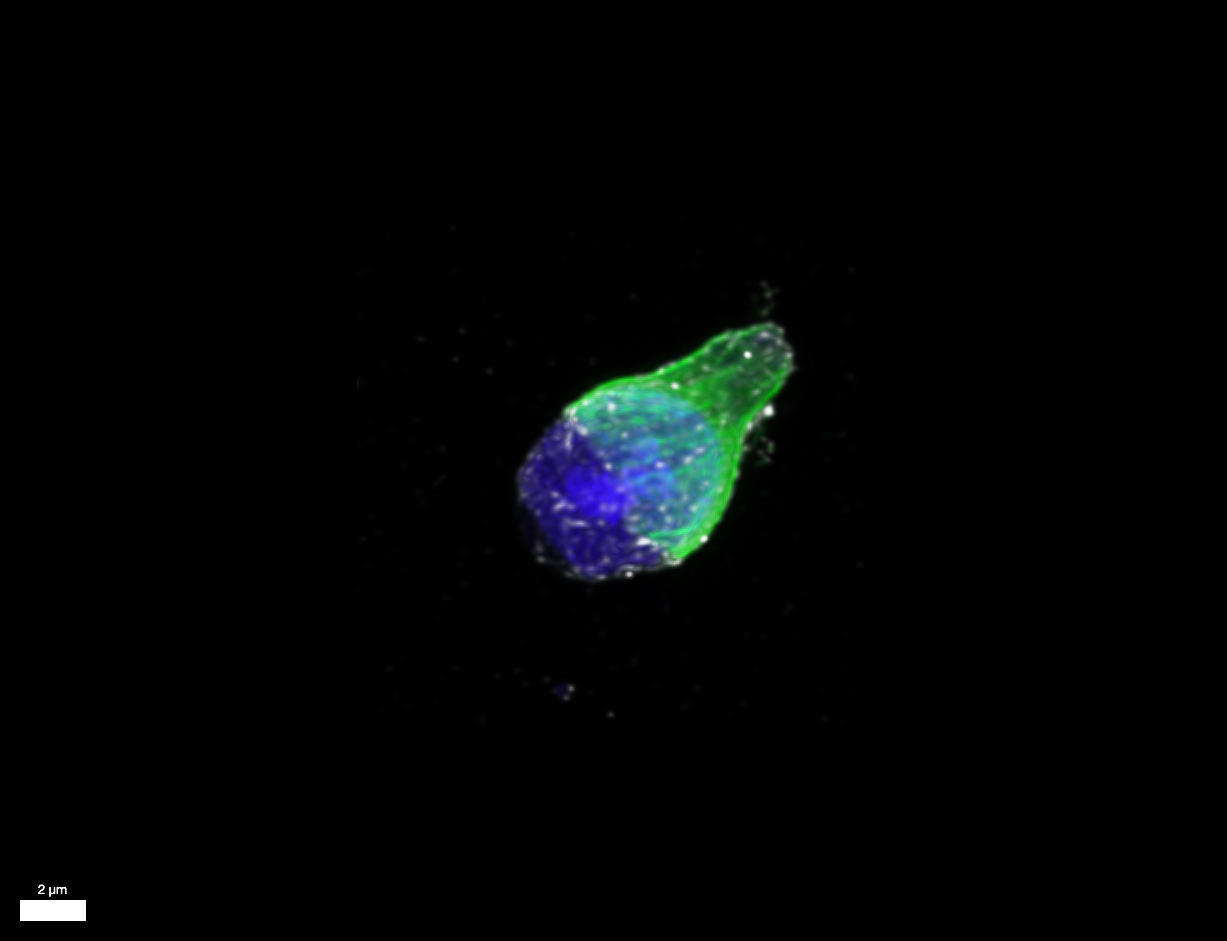

Supplement: Supplementary file 5 — Source data Fig. 5 [file 44319_2024_159_MOESM5_ESM.zip › EMBOR-2023-58207V1_SourceDataForFig5/5G/Tube1GCKO:GCKO/EMBOR-2023-58207V1_SourceDataForFig5GEarly_DAPI:Merged.tif]

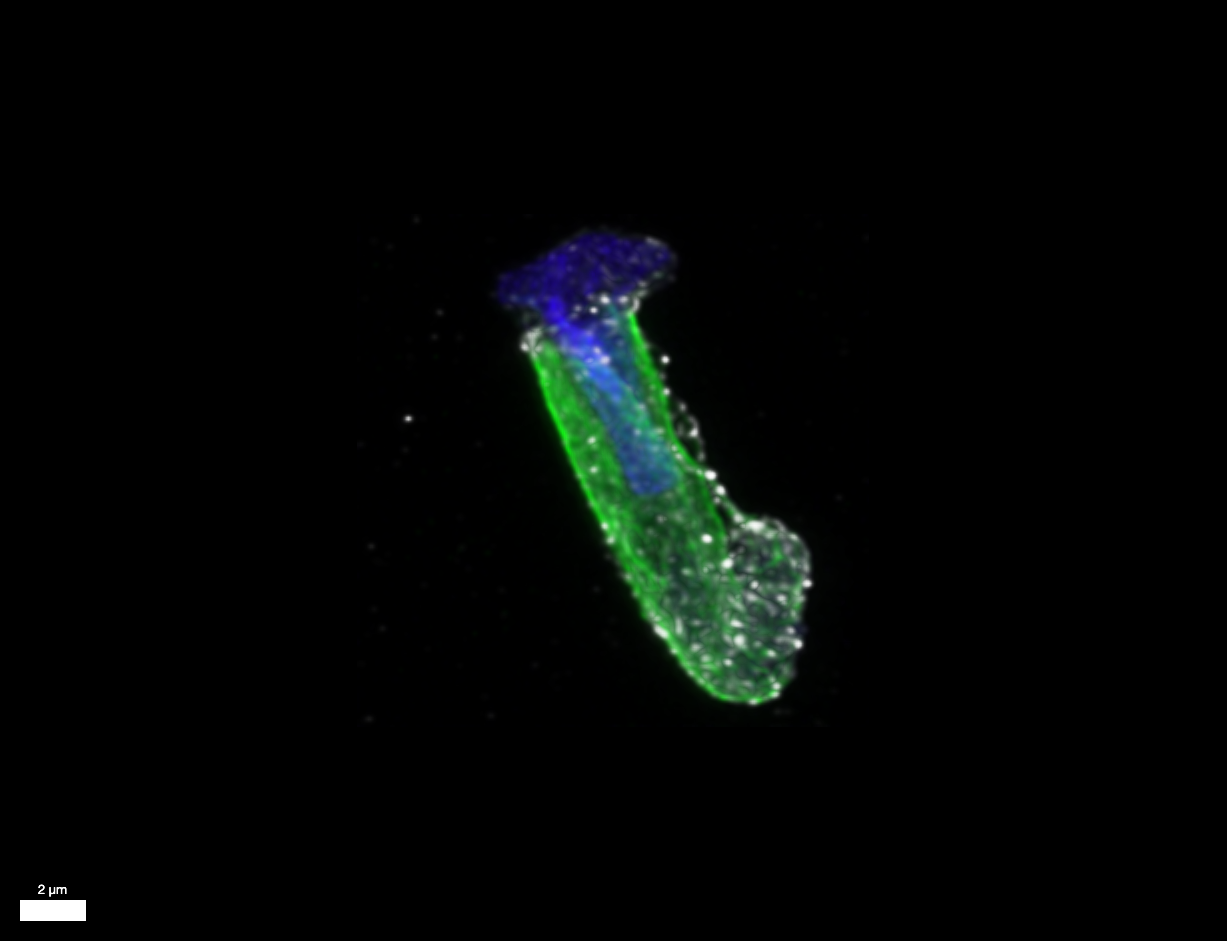

Supplement: Supplementary file 5 — Source data Fig. 5 [file 44319_2024_159_MOESM5_ESM.zip › EMBOR-2023-58207V1_SourceDataForFig5/5G/Tube1GCKO:GCKO/EMBOR-2023-58207V1_SourceDataForFig5GMid_DAPI:Merged.tif]

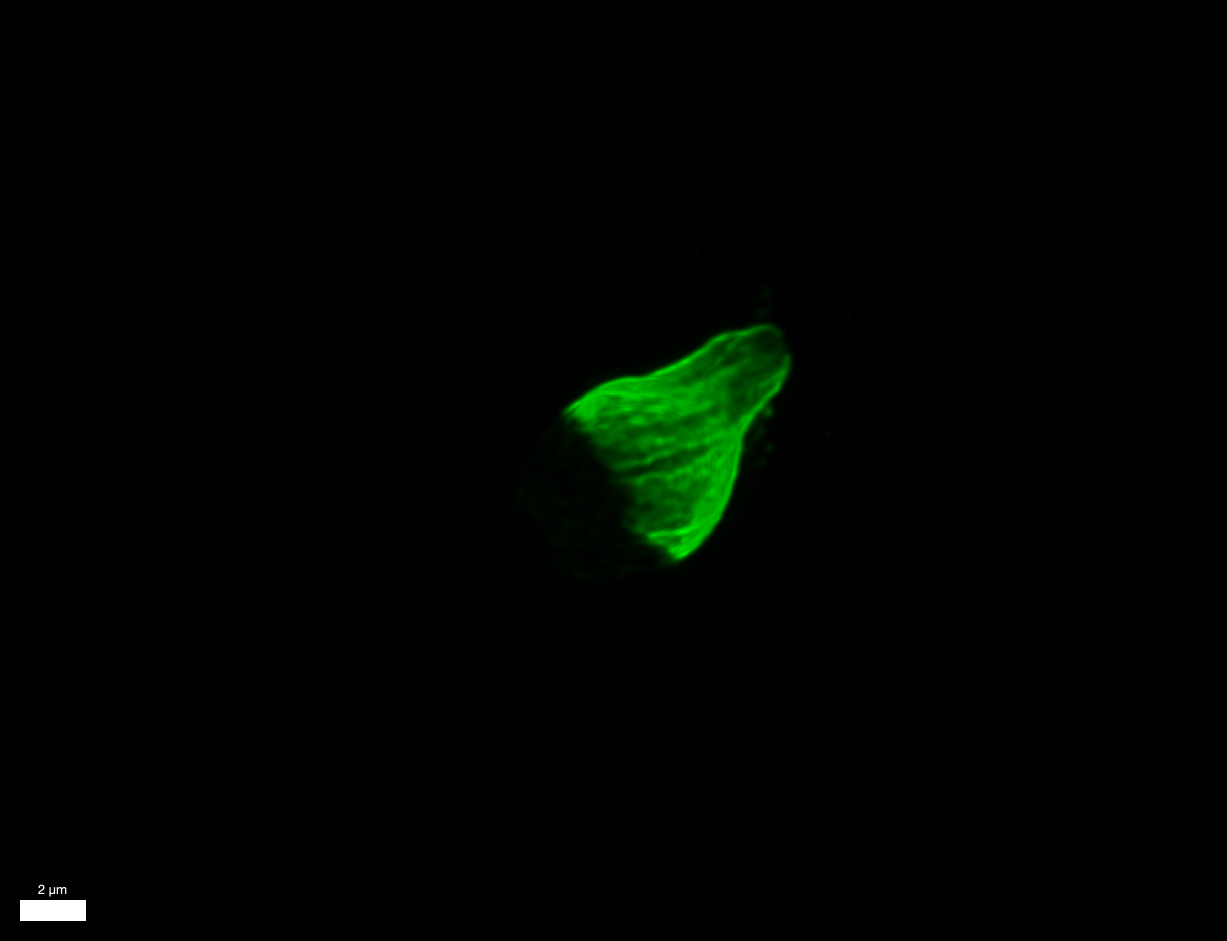

Supplement: Supplementary file 5 — Source data Fig. 5 [file 44319_2024_159_MOESM5_ESM.zip › EMBOR-2023-58207V1_SourceDataForFig5/5G/Tube1GCKO:GCKO/EMBOR-2023-58207V1_SourceDataForFig5GEarly_alpha tubulin.png]

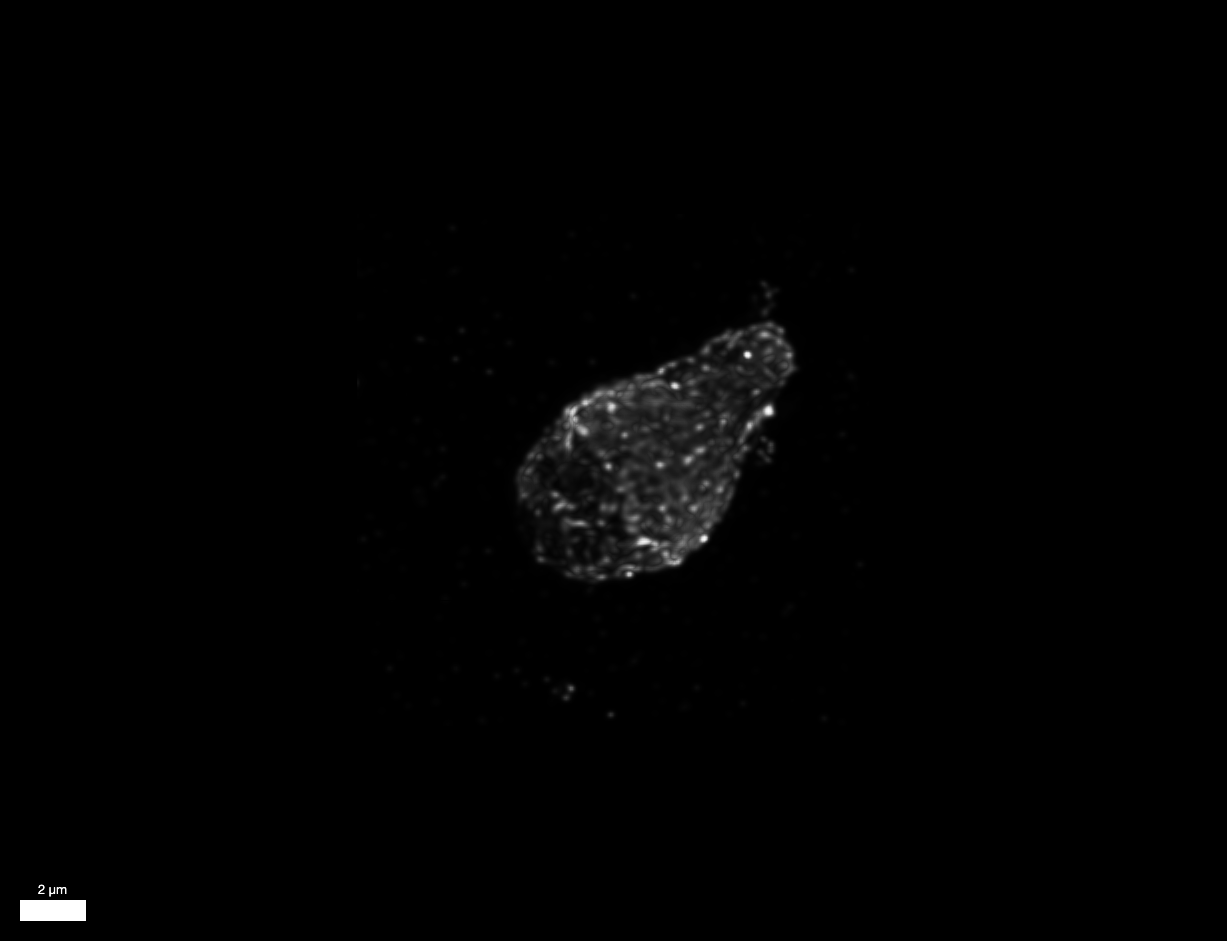

Supplement: Supplementary file 5 — Source data Fig. 5 [file 44319_2024_159_MOESM5_ESM.zip › EMBOR-2023-58207V1_SourceDataForFig5/5G/Tube1GCKO:GCKO/EMBOR-2023-58207V1_SourceDataForFig5GEarly_KATNB1.png]

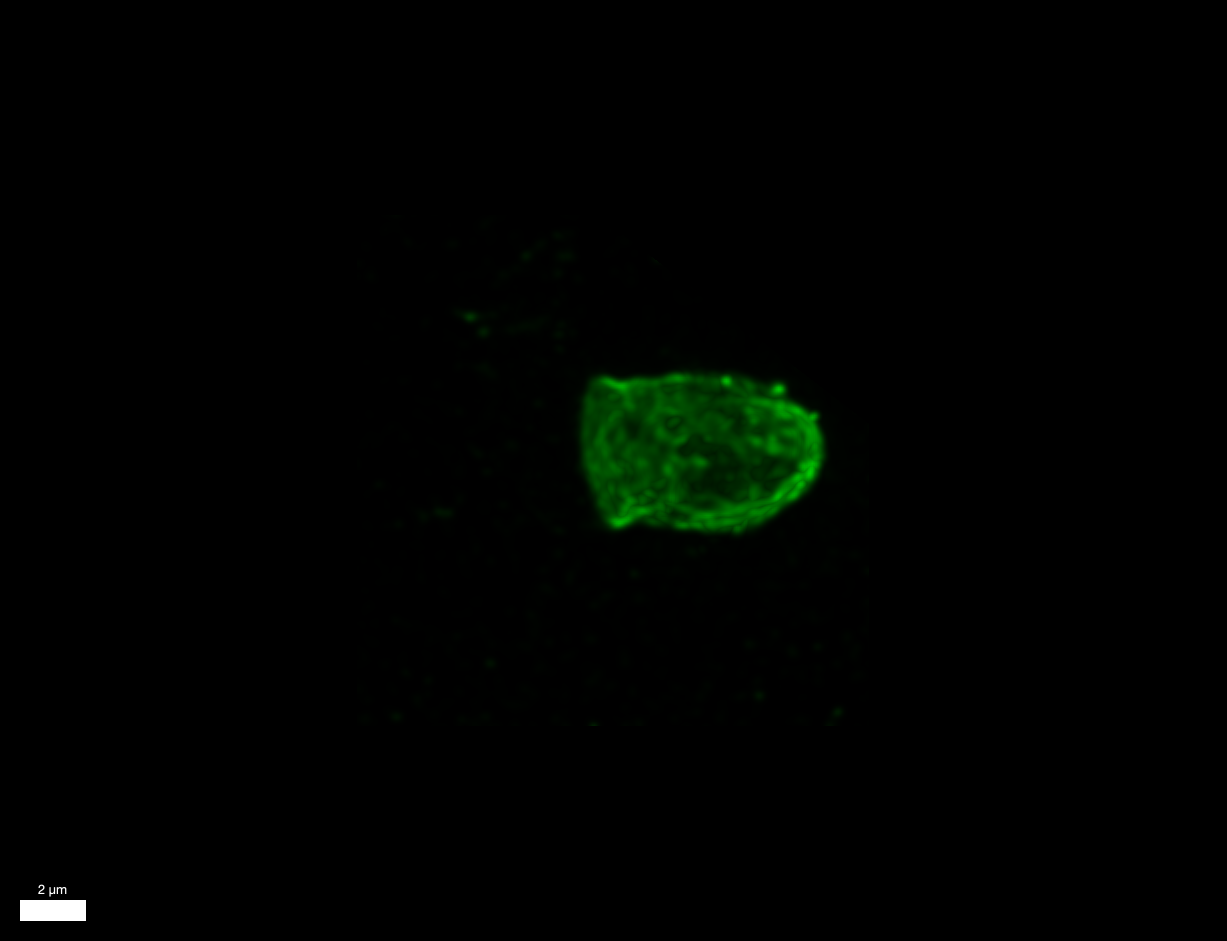

Supplement: Supplementary file 5 — Source data Fig. 5 [file 44319_2024_159_MOESM5_ESM.zip › EMBOR-2023-58207V1_SourceDataForFig5/5A/Tube1Flox:Flox/EMBOR-2023-58207V1_SourceDataForFig5ALate_alpha tubulin.png]

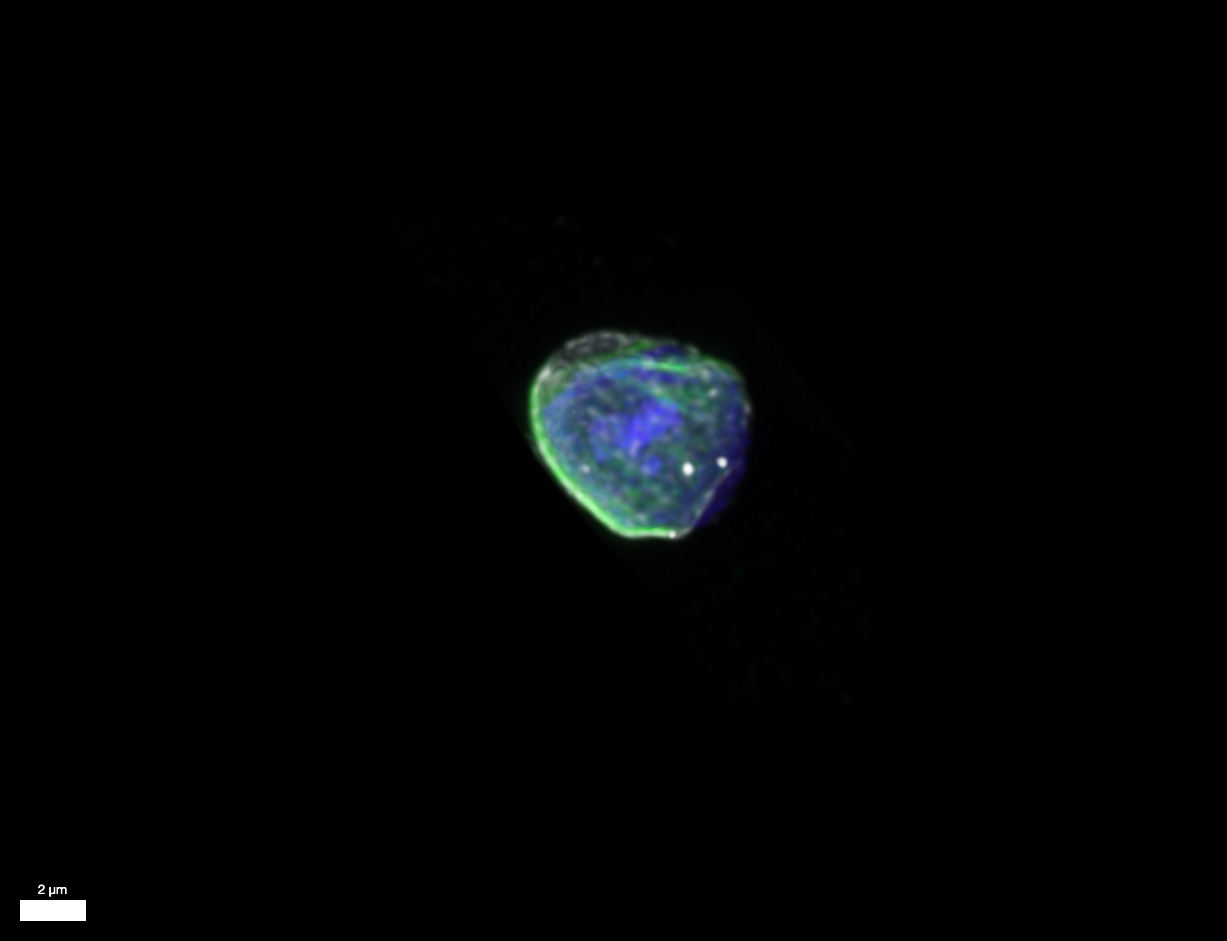

Supplement: Supplementary file 5 — Source data Fig. 5 [file 44319_2024_159_MOESM5_ESM.zip › EMBOR-2023-58207V1_SourceDataForFig5/5A/Tube1Flox:Flox/EMBOR-2023-58207V1_SourceDataForFig5AEarly_DAPI:Merged.tif]

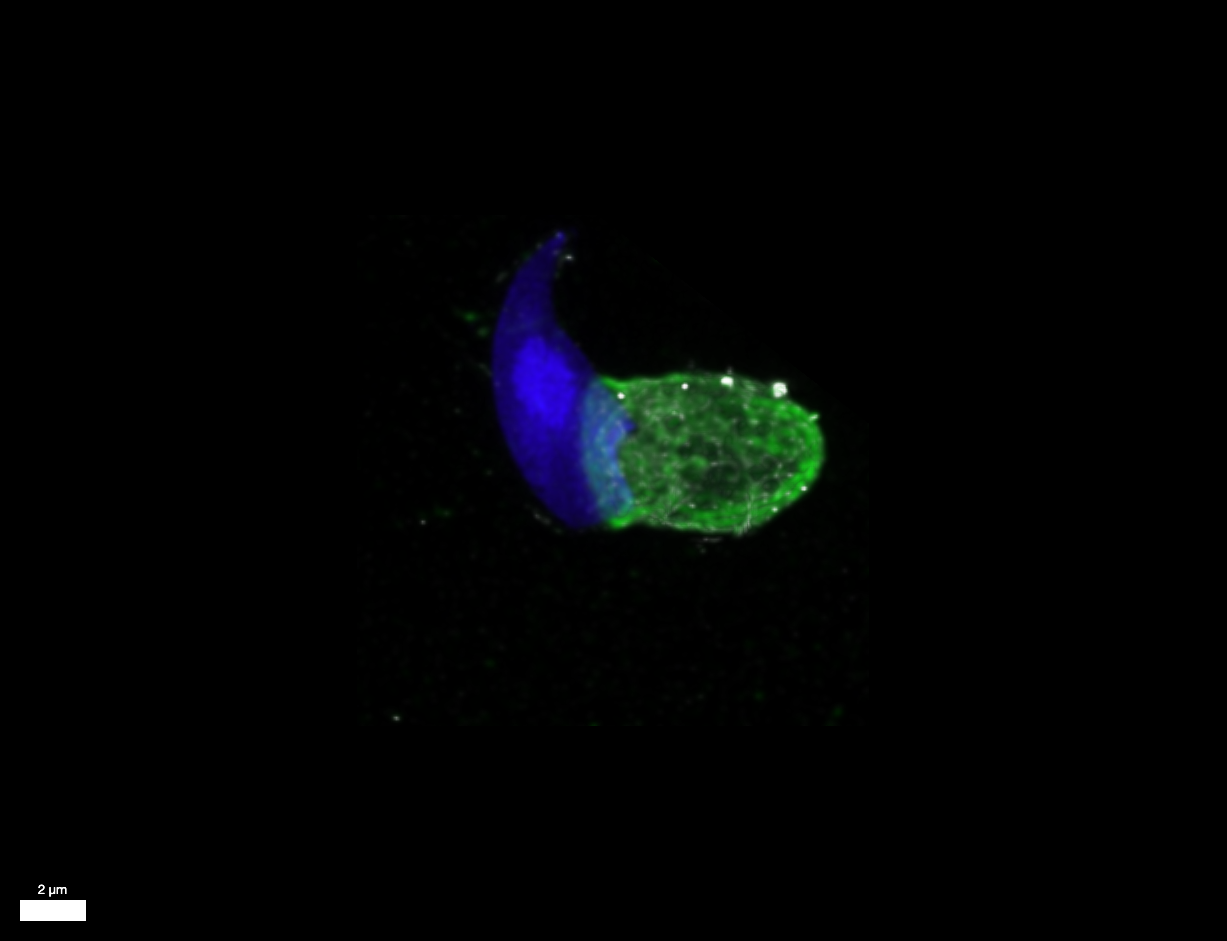

Supplement: Supplementary file 5 — Source data Fig. 5 [file 44319_2024_159_MOESM5_ESM.zip › EMBOR-2023-58207V1_SourceDataForFig5/5A/Tube1Flox:Flox/EMBOR-2023-58207V1_SourceDataForFig5ALate_DAPI:Merged.tif]

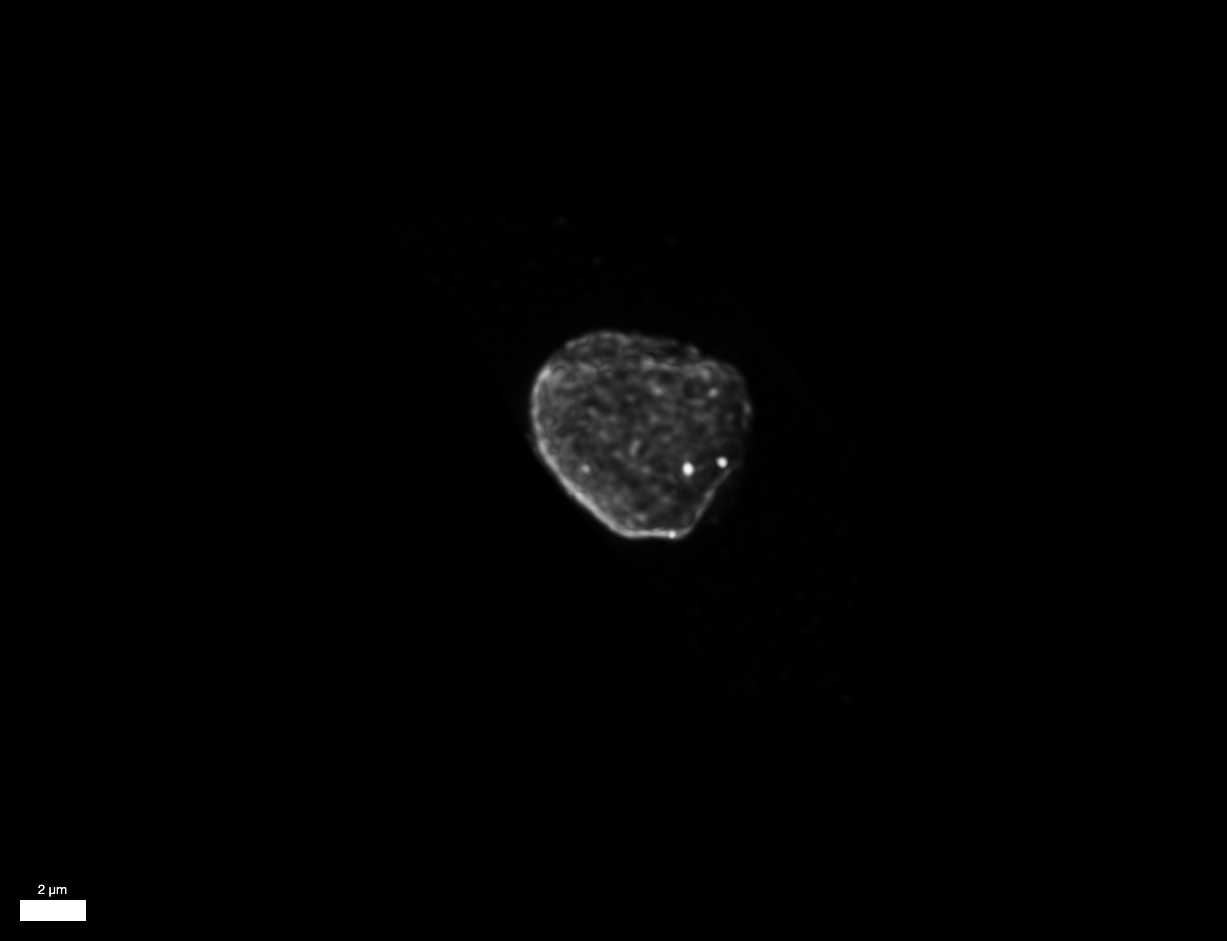

Supplement: Supplementary file 5 — Source data Fig. 5 [file 44319_2024_159_MOESM5_ESM.zip › EMBOR-2023-58207V1_SourceDataForFig5/5A/Tube1Flox:Flox/EMBOR-2023-58207V1_SourceDataForFig5AEarly_KATNA1.png]

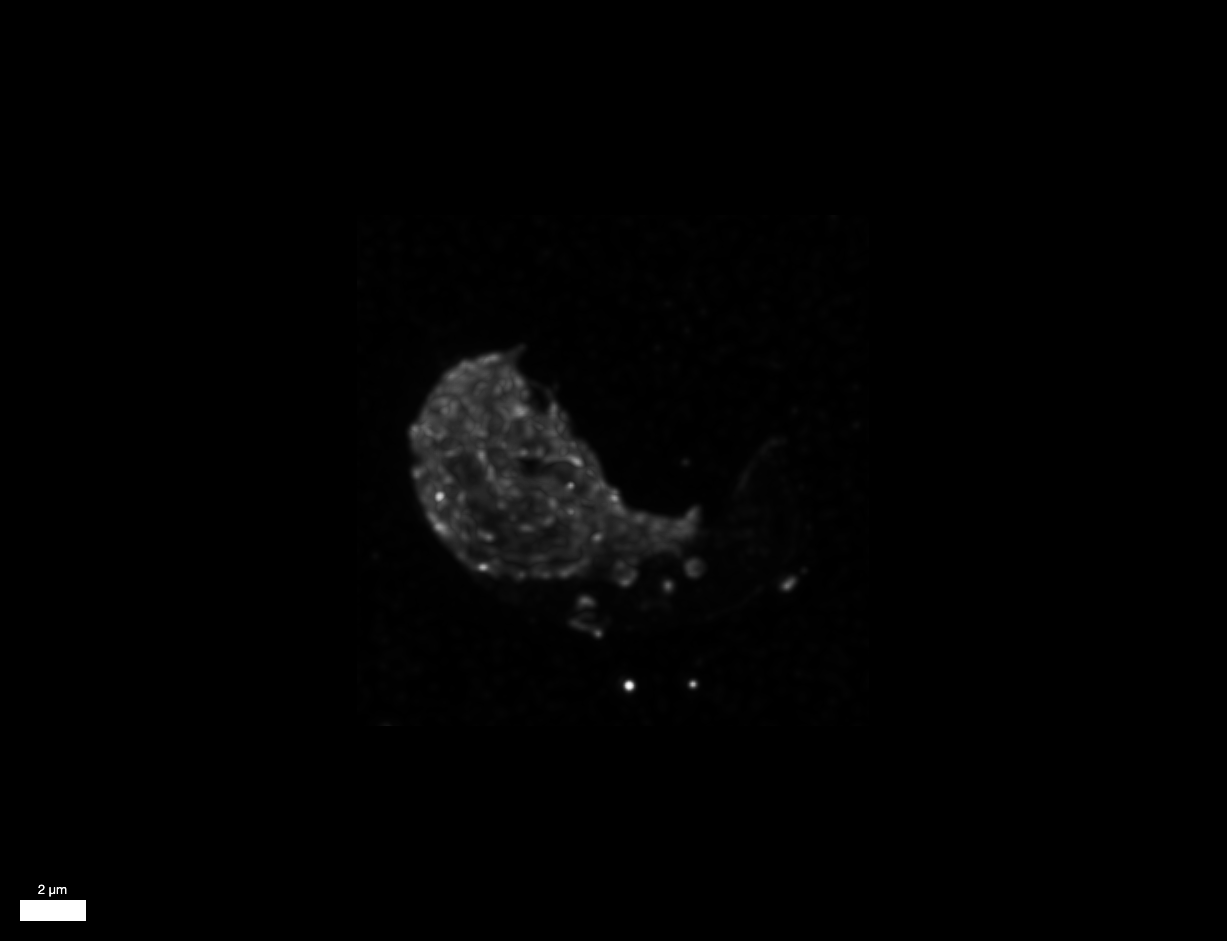

Supplement: Supplementary file 5 — Source data Fig. 5 [file 44319_2024_159_MOESM5_ESM.zip › EMBOR-2023-58207V1_SourceDataForFig5/5A/Tube1Flox:Flox/EMBOR-2023-58207V1_SourceDataForFig5AMid_KATNA1.png]

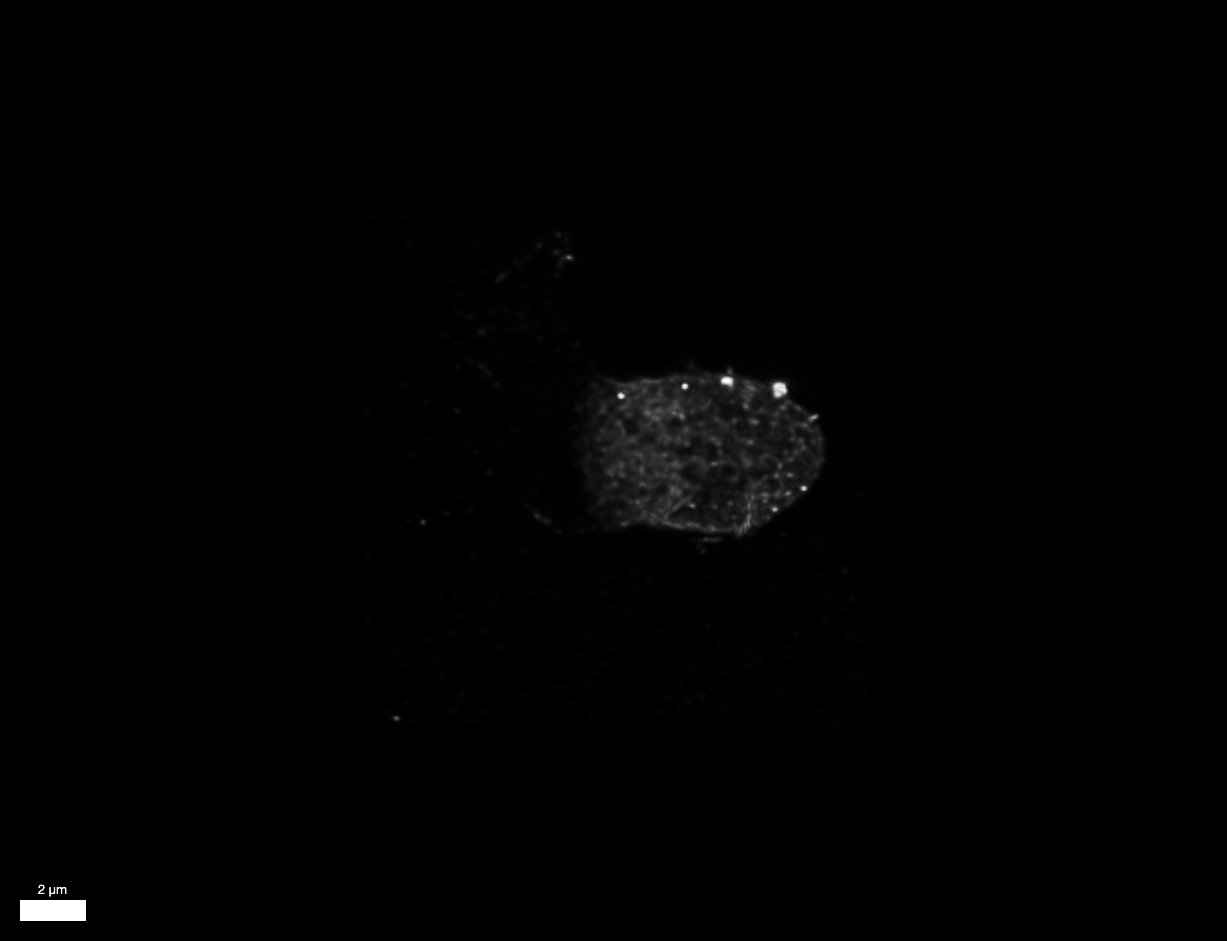

Supplement: Supplementary file 5 — Source data Fig. 5 [file 44319_2024_159_MOESM5_ESM.zip › EMBOR-2023-58207V1_SourceDataForFig5/5A/Tube1Flox:Flox/EMBOR-2023-58207V1_SourceDataForFig5ALate_KATNA1.png]

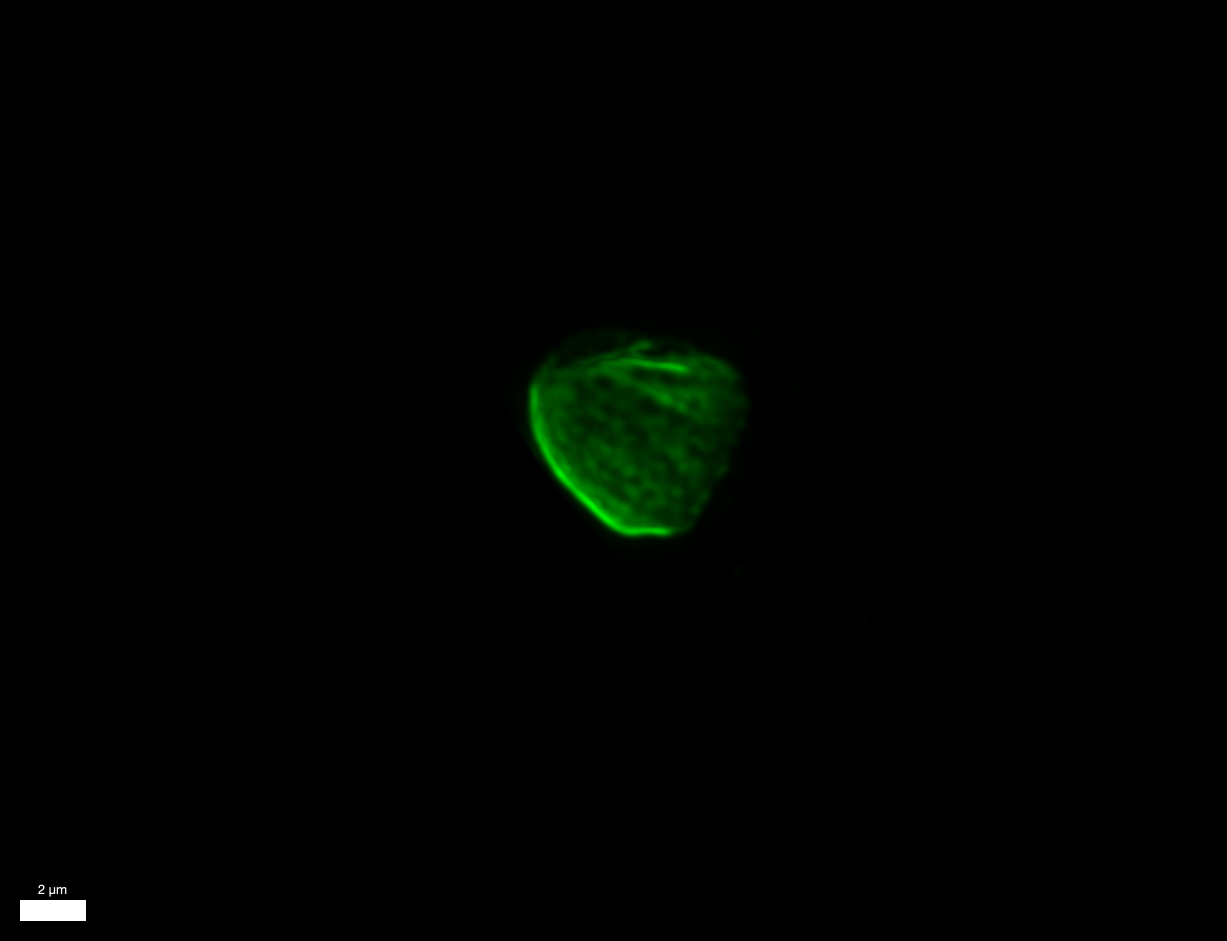

Supplement: Supplementary file 5 — Source data Fig. 5 [file 44319_2024_159_MOESM5_ESM.zip › EMBOR-2023-58207V1_SourceDataForFig5/5A/Tube1Flox:Flox/EMBOR-2023-58207V1_SourceDataForFig5AEarly_alpha tubulin.png]

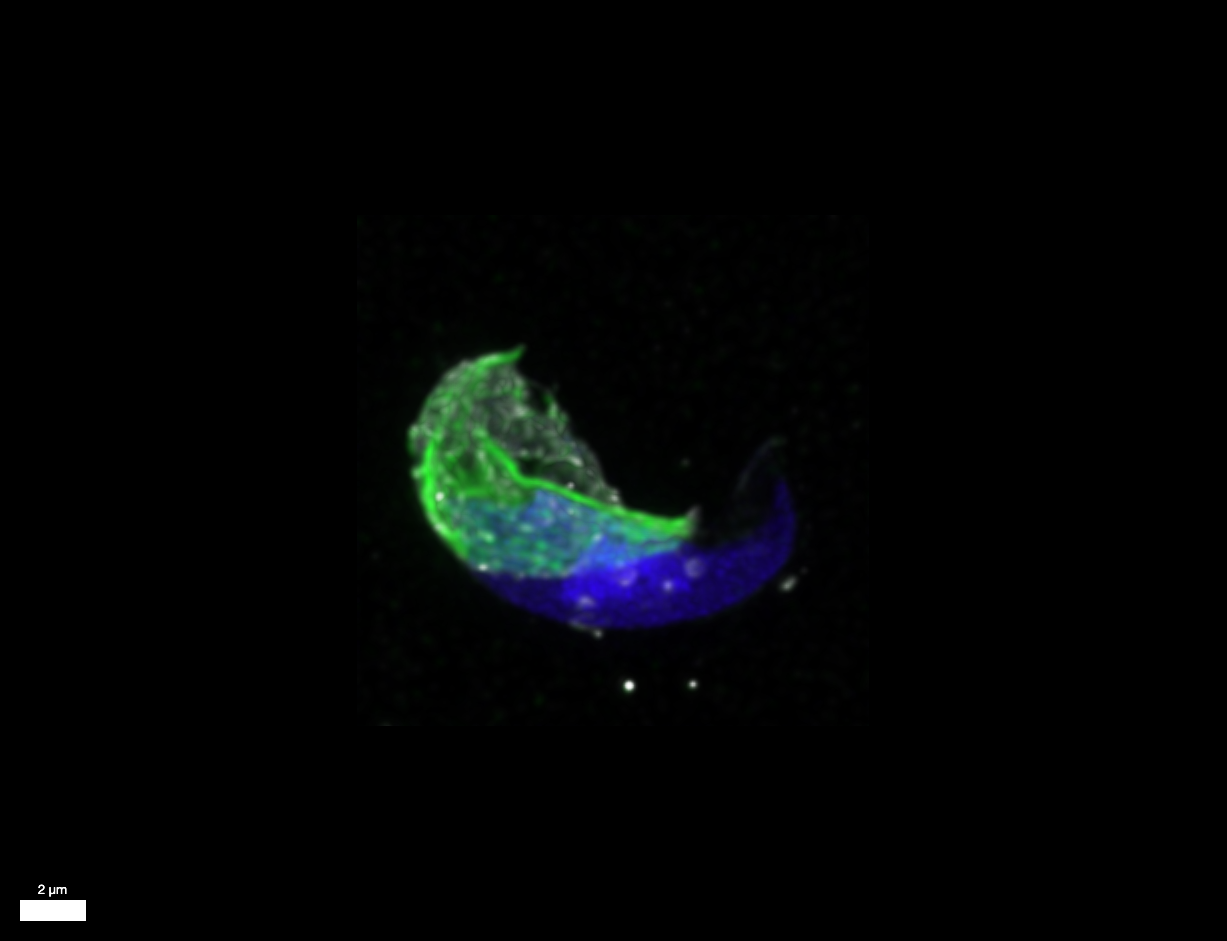

Supplement: Supplementary file 5 — Source data Fig. 5 [file 44319_2024_159_MOESM5_ESM.zip › EMBOR-2023-58207V1_SourceDataForFig5/5A/Tube1Flox:Flox/EMBOR-2023-58207V1_SourceDataForFig5AMid_DAPI:Merged.tif]

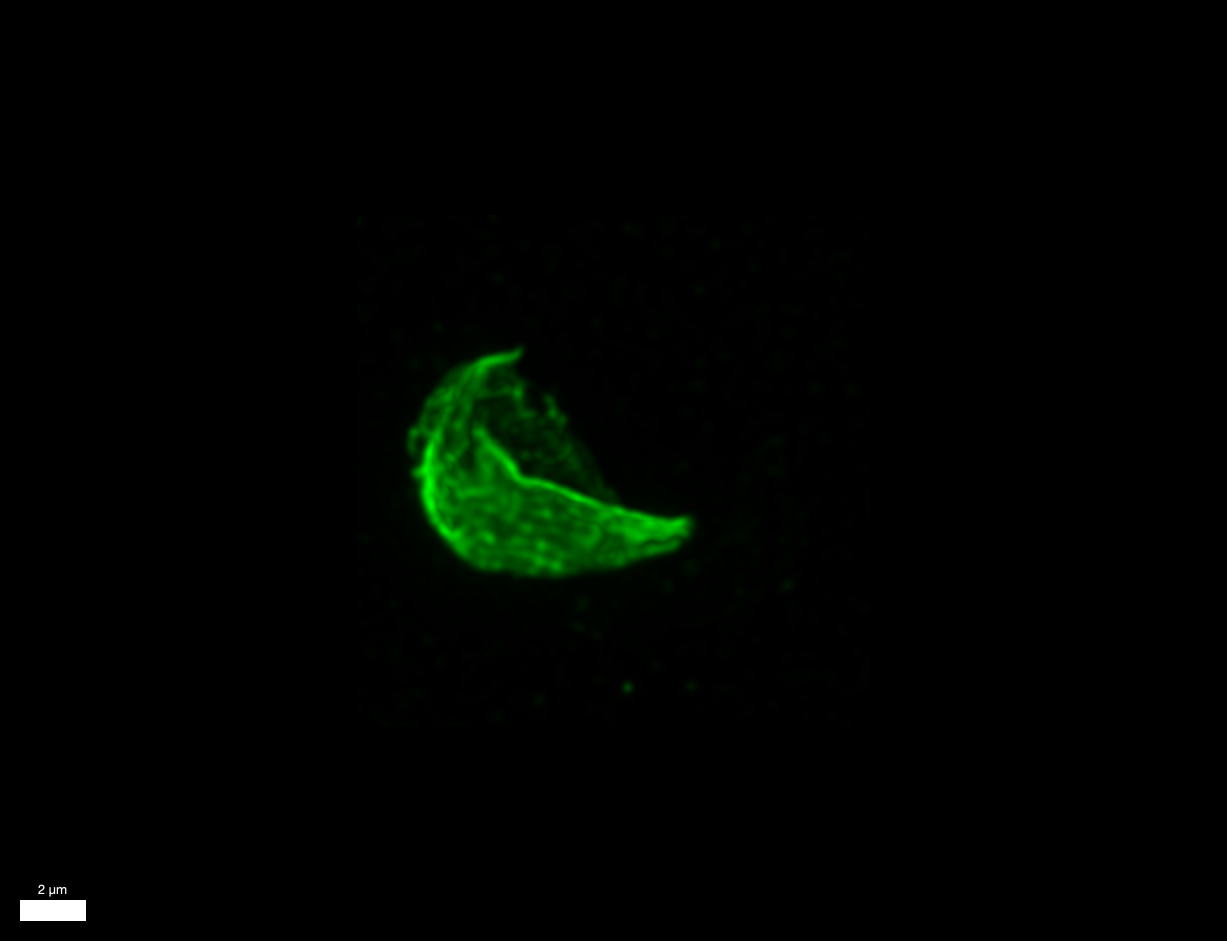

Supplement: Supplementary file 5 — Source data Fig. 5 [file 44319_2024_159_MOESM5_ESM.zip › EMBOR-2023-58207V1_SourceDataForFig5/5A/Tube1Flox:Flox/EMBOR-2023-58207V1_SourceDataForFig5AMid_alpha tubulin.png]

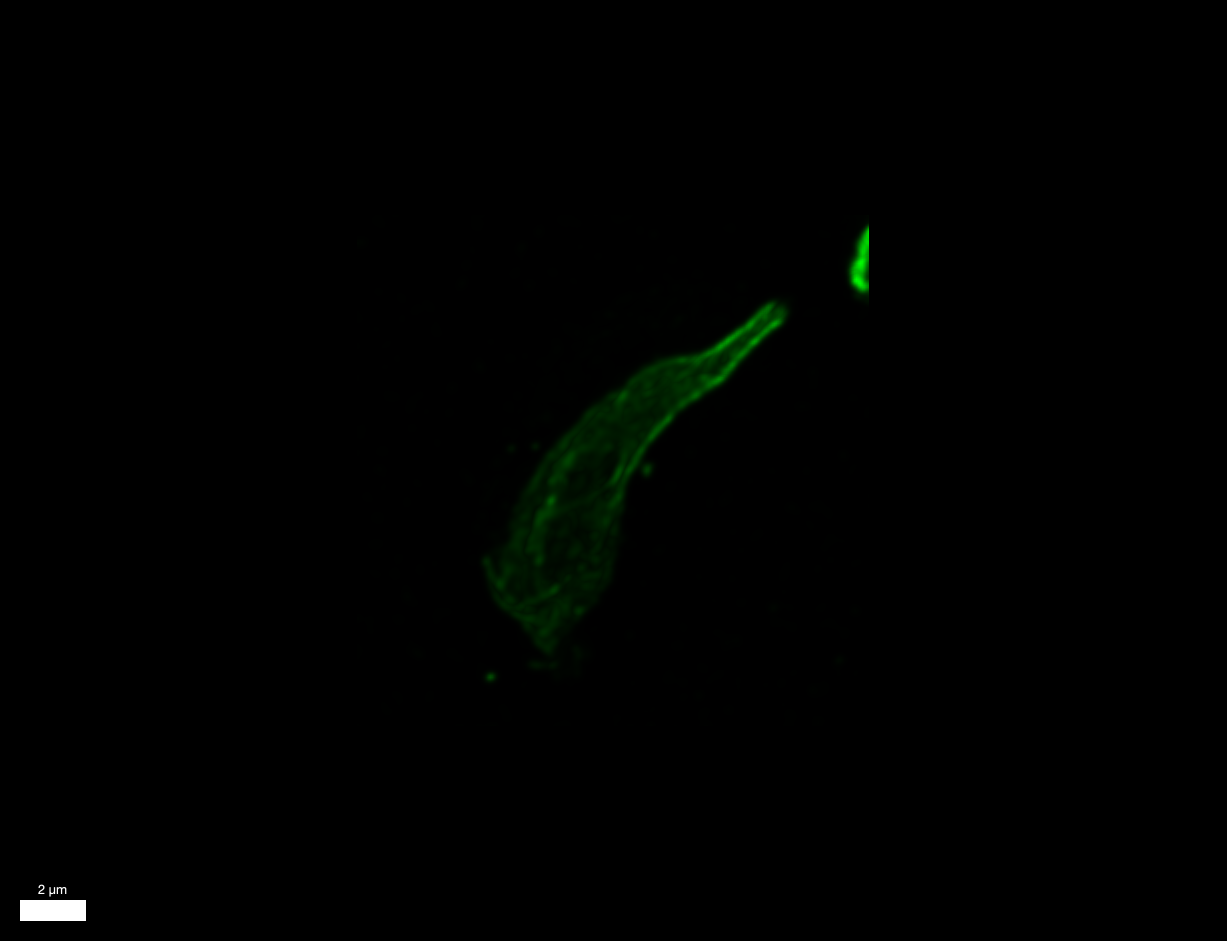

Supplement: Supplementary file 5 — Source data Fig. 5 [file 44319_2024_159_MOESM5_ESM.zip › EMBOR-2023-58207V1_SourceDataForFig5/5A/Tube1GCKO:GCKO/EMBOR-2023-58207V1_SourceDataForFig5ALate_alpha tubulin.png]

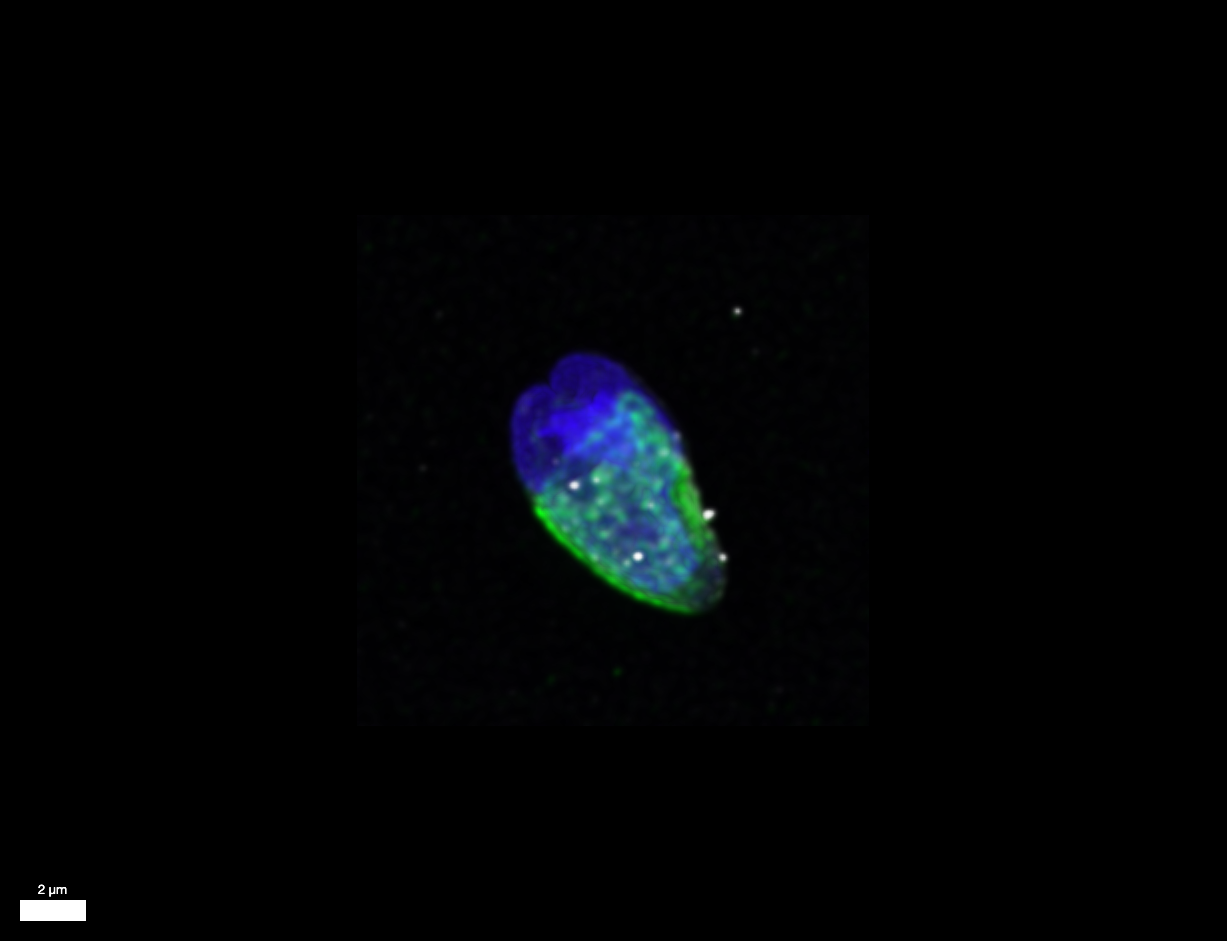

Supplement: Supplementary file 5 — Source data Fig. 5 [file 44319_2024_159_MOESM5_ESM.zip › EMBOR-2023-58207V1_SourceDataForFig5/5A/Tube1GCKO:GCKO/EMBOR-2023-58207V1_SourceDataForFig5AEarly_DAPI:Merged.tif]

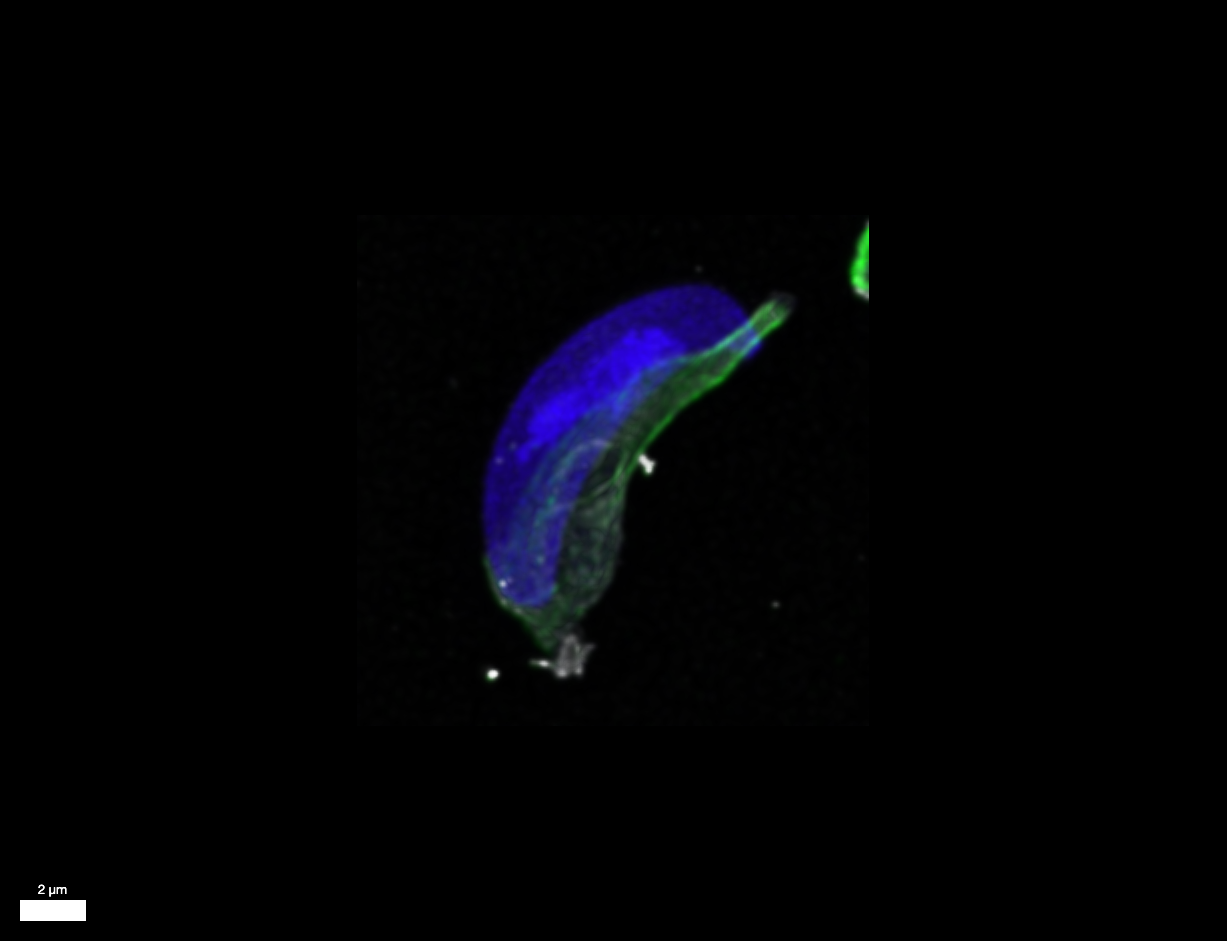

Supplement: Supplementary file 5 — Source data Fig. 5 [file 44319_2024_159_MOESM5_ESM.zip › EMBOR-2023-58207V1_SourceDataForFig5/5A/Tube1GCKO:GCKO/EMBOR-2023-58207V1_SourceDataForFig5ALate_DAPI:Merged.tif]

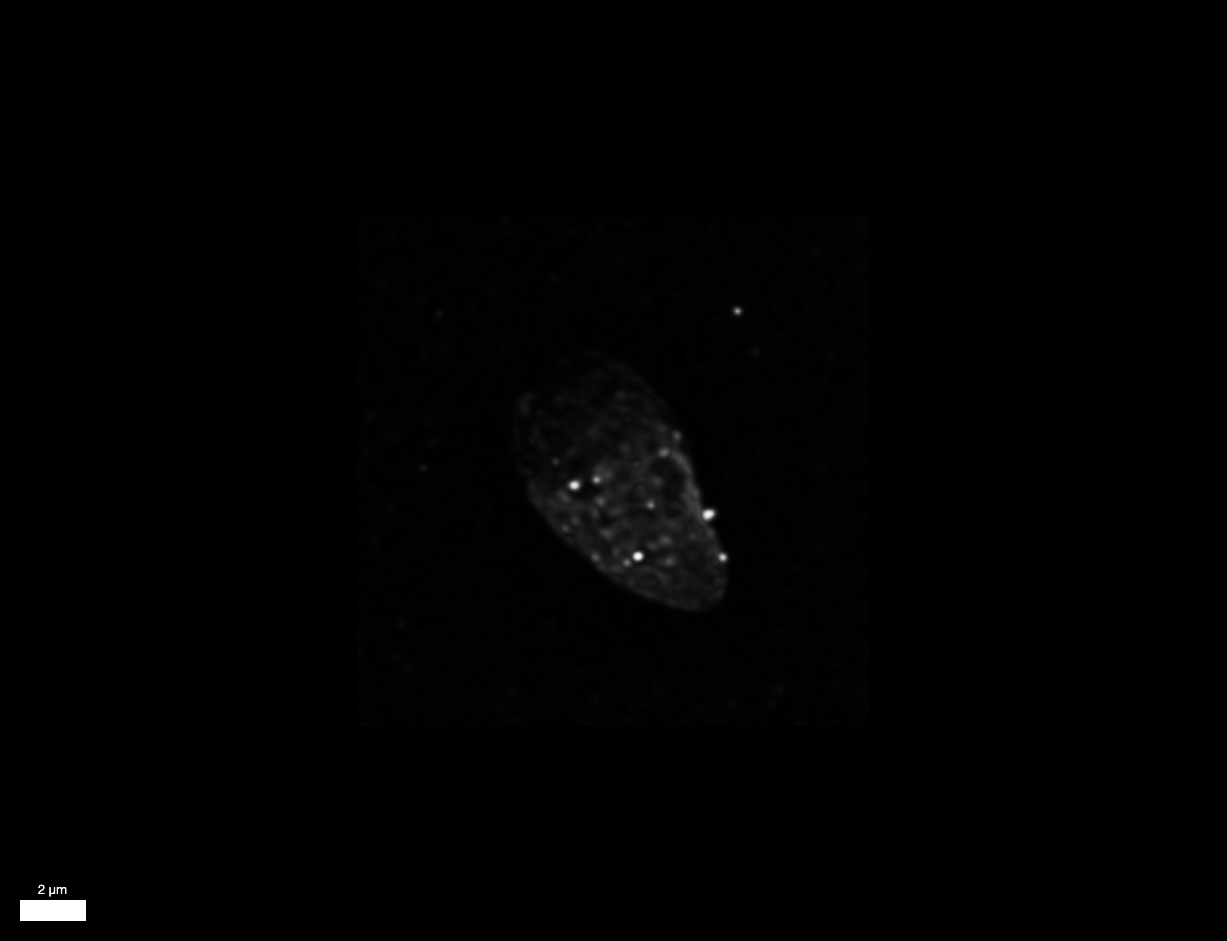

Supplement: Supplementary file 5 — Source data Fig. 5 [file 44319_2024_159_MOESM5_ESM.zip › EMBOR-2023-58207V1_SourceDataForFig5/5A/Tube1GCKO:GCKO/EMBOR-2023-58207V1_SourceDataForFig5AEarly_KATNA1.png]

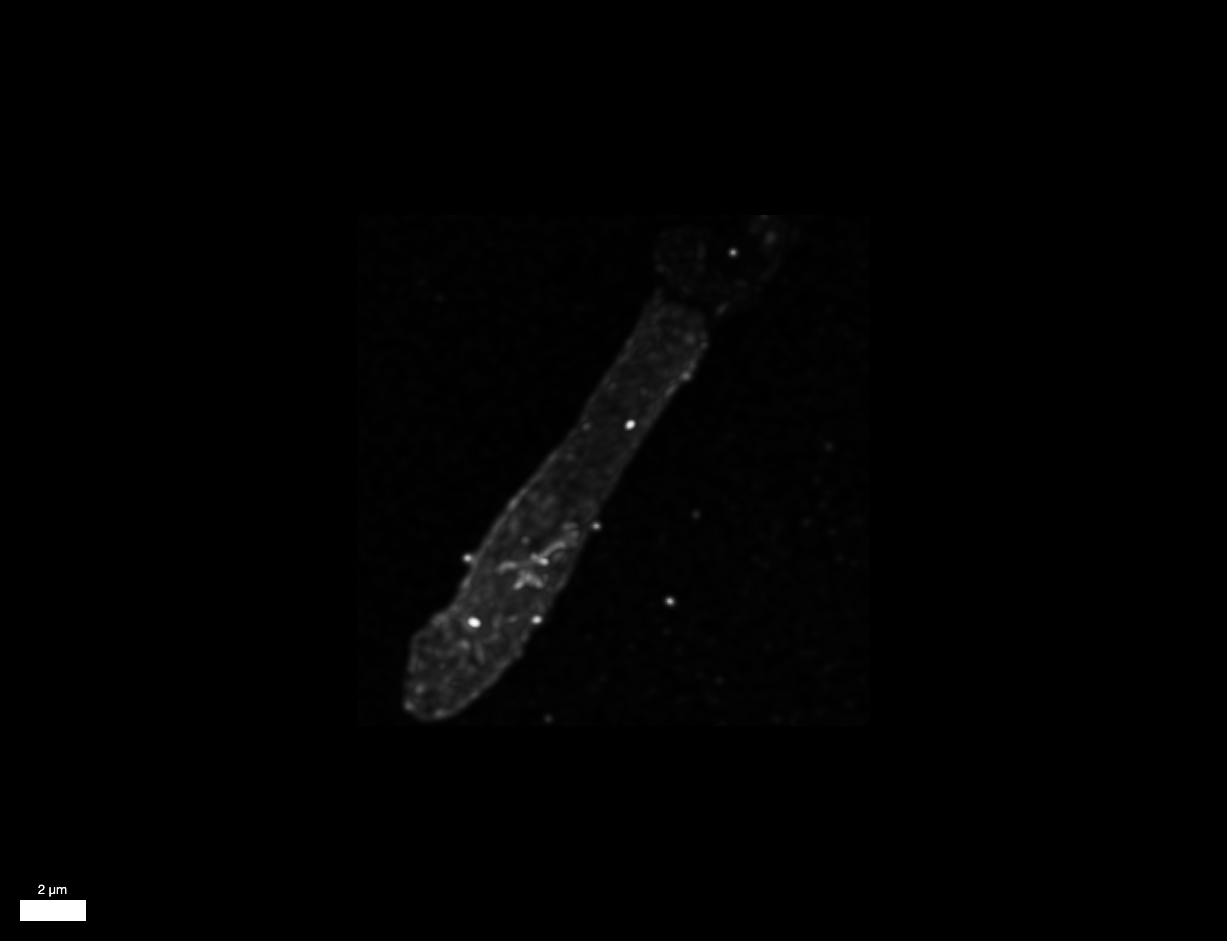

Supplement: Supplementary file 5 — Source data Fig. 5 [file 44319_2024_159_MOESM5_ESM.zip › EMBOR-2023-58207V1_SourceDataForFig5/5A/Tube1GCKO:GCKO/EMBOR-2023-58207V1_SourceDataForFig5AMid_KATNA1.png]

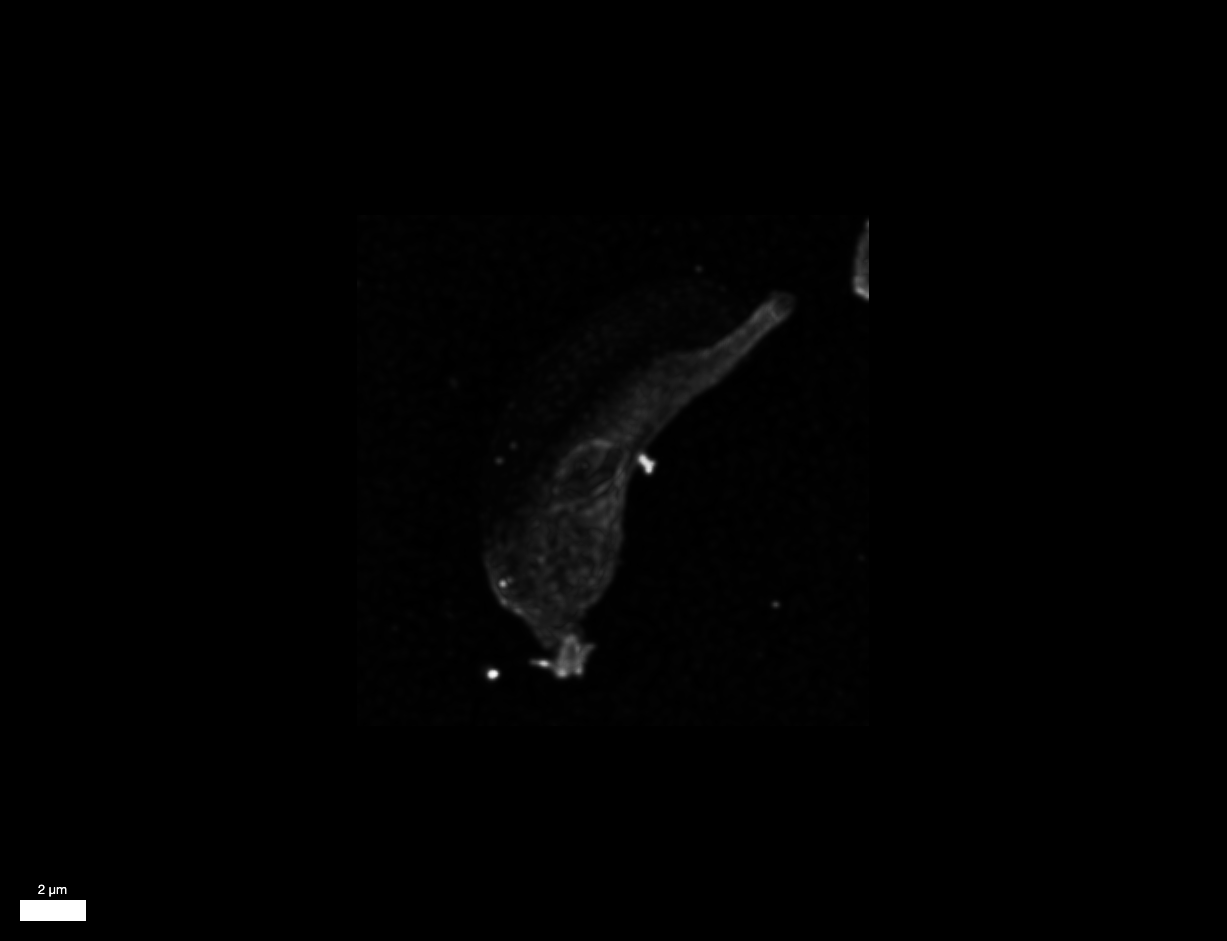

Supplement: Supplementary file 5 — Source data Fig. 5 [file 44319_2024_159_MOESM5_ESM.zip › EMBOR-2023-58207V1_SourceDataForFig5/5A/Tube1GCKO:GCKO/EMBOR-2023-58207V1_SourceDataForFig5ALate_KATNA1.png]

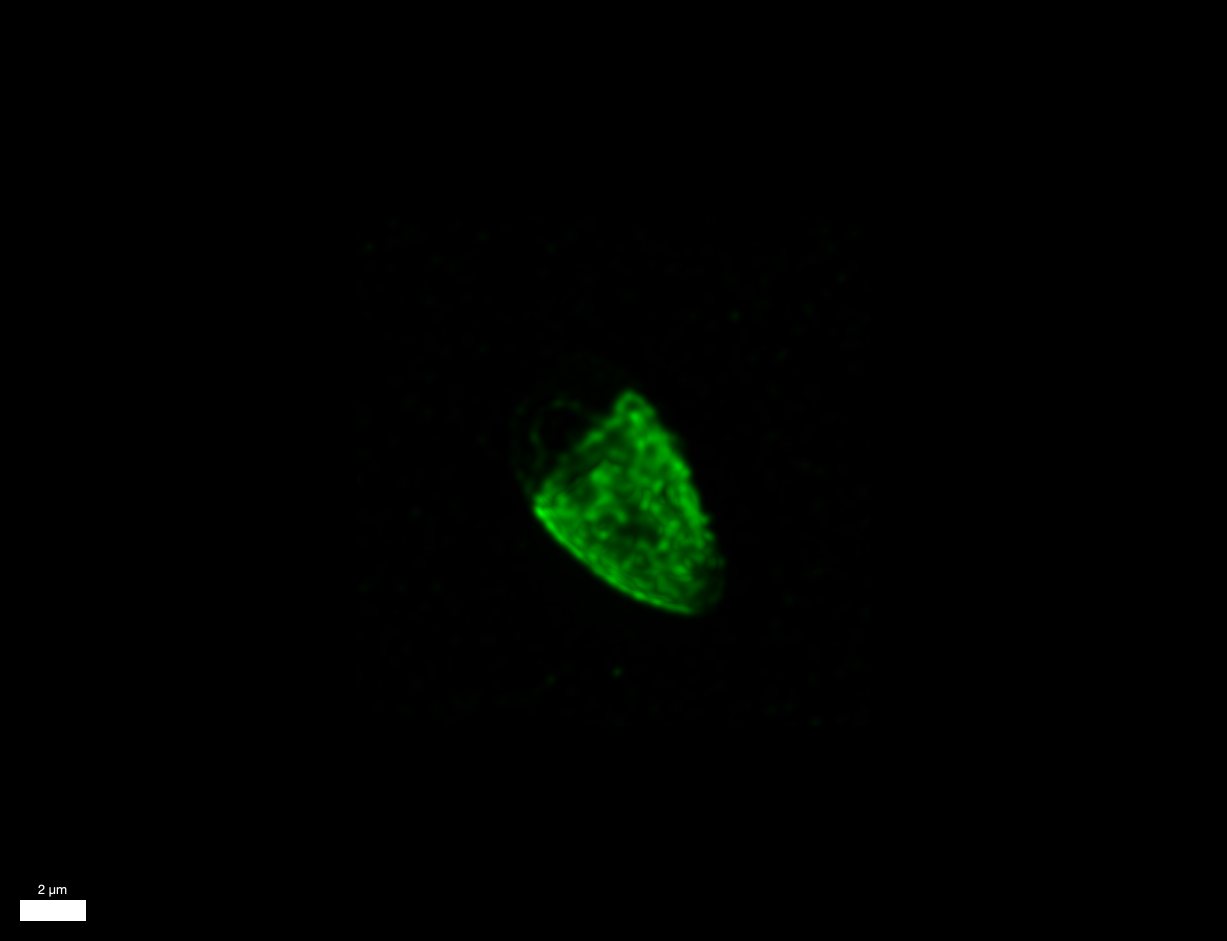

Supplement: Supplementary file 5 — Source data Fig. 5 [file 44319_2024_159_MOESM5_ESM.zip › EMBOR-2023-58207V1_SourceDataForFig5/5A/Tube1GCKO:GCKO/EMBOR-2023-58207V1_SourceDataForFig5AEarly_alpha tubulin.png]

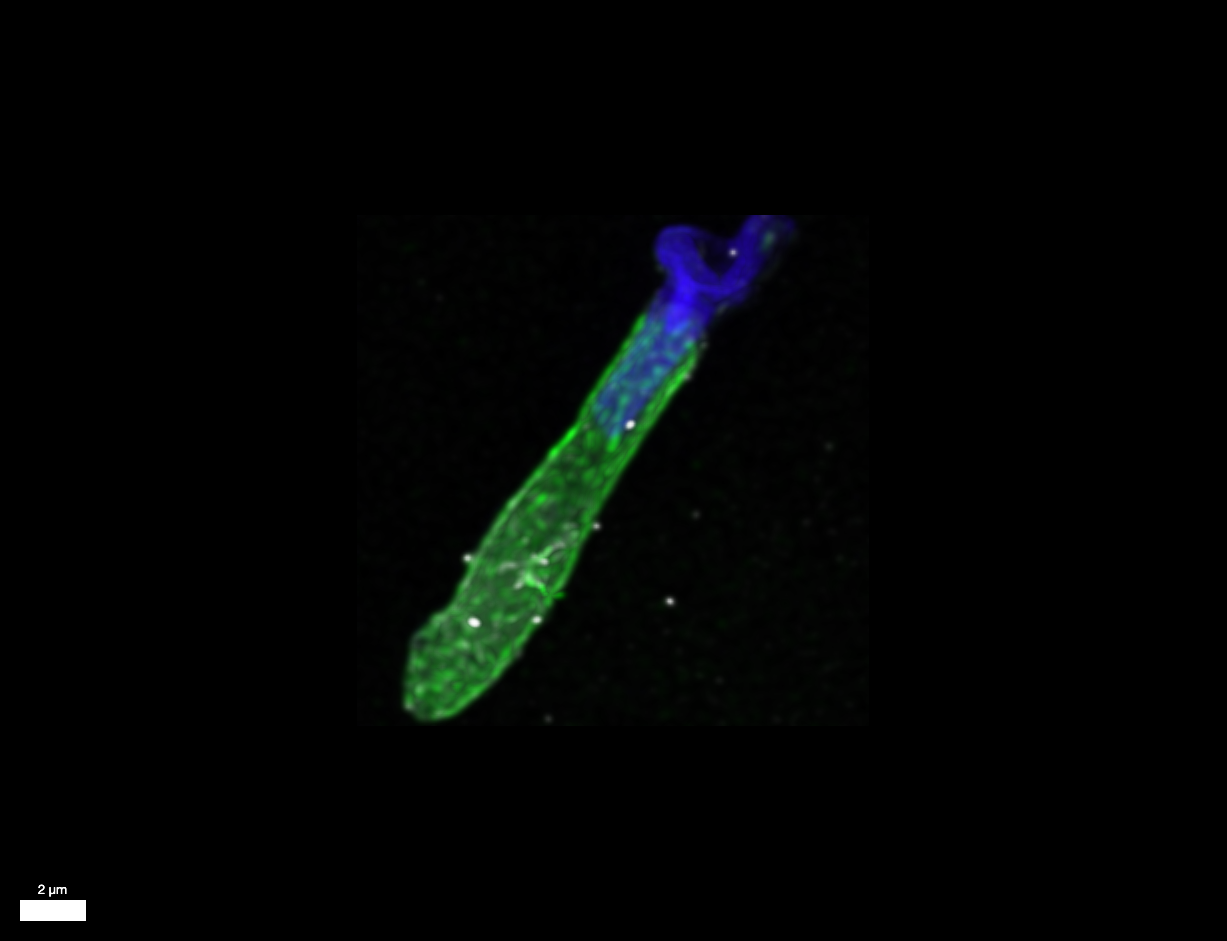

Supplement: Supplementary file 5 — Source data Fig. 5 [file 44319_2024_159_MOESM5_ESM.zip › EMBOR-2023-58207V1_SourceDataForFig5/5A/Tube1GCKO:GCKO/EMBOR-2023-58207V1_SourceDataForFig5AMid_DAPI:Merged.tif]

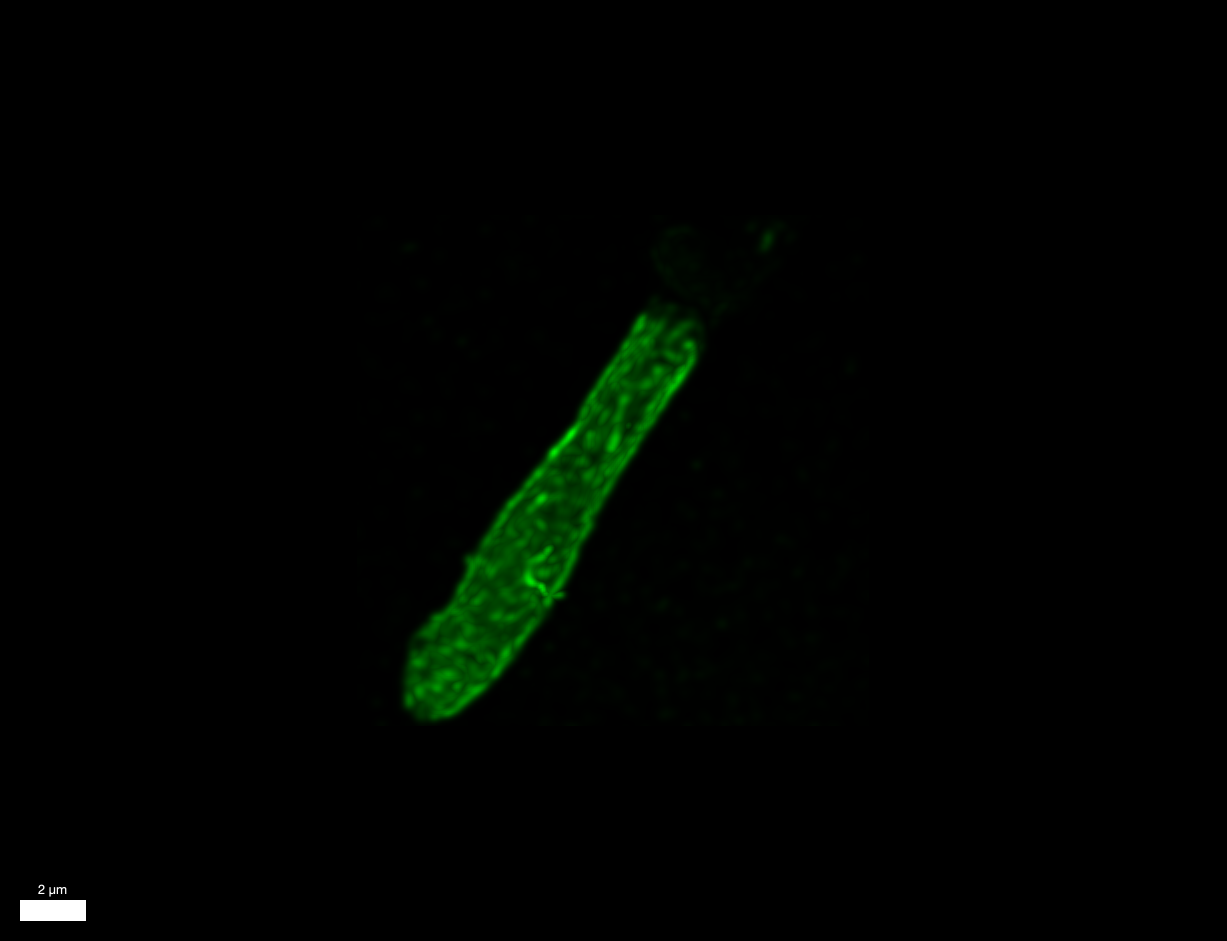

Supplement: Supplementary file 5 — Source data Fig. 5 [file 44319_2024_159_MOESM5_ESM.zip › EMBOR-2023-58207V1_SourceDataForFig5/5A/Tube1GCKO:GCKO/EMBOR-2023-58207V1_SourceDataForFig5AMid_alpha tubulin.png]

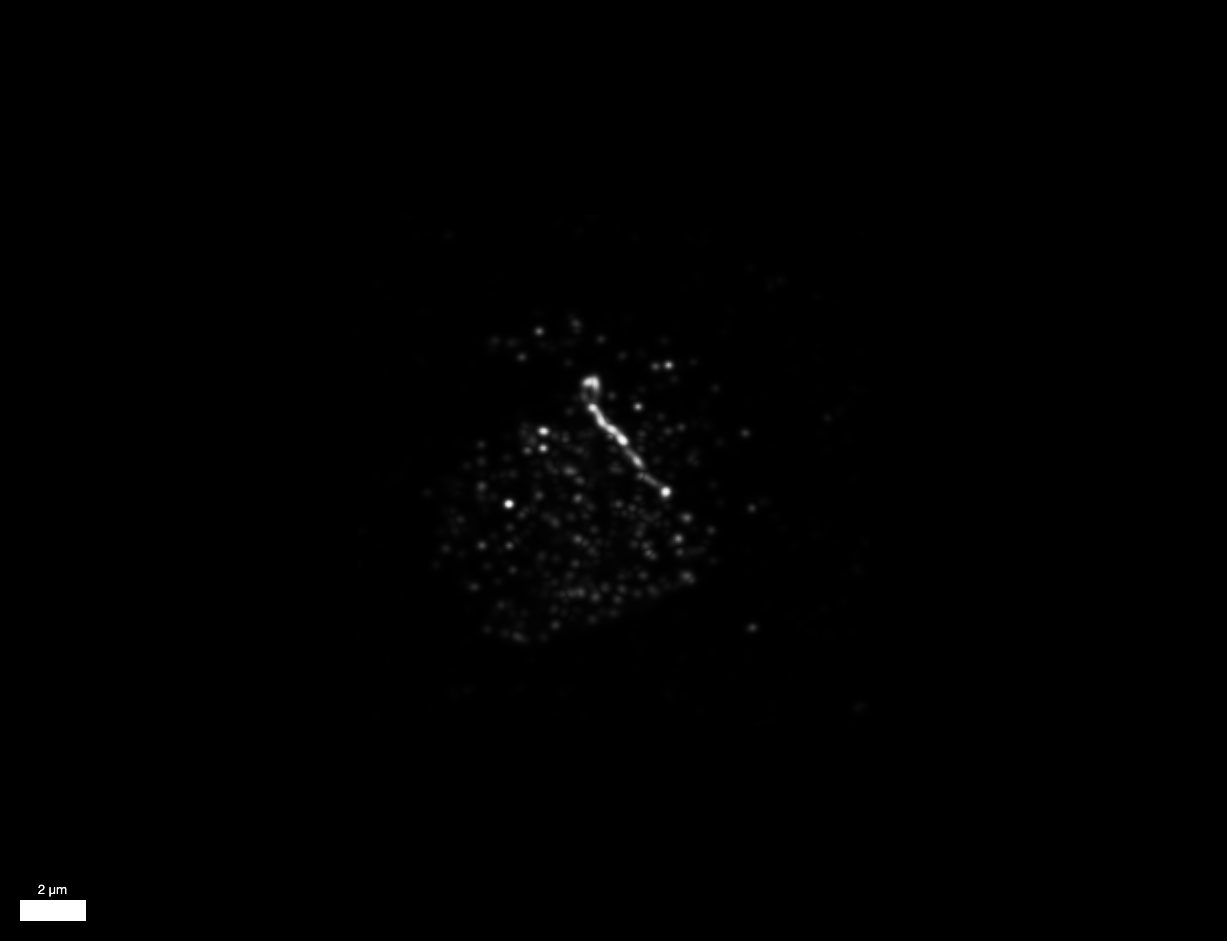

Supplement: Supplementary file 5 — Source data Fig. 5 [file 44319_2024_159_MOESM5_ESM.zip › EMBOR-2023-58207V1_SourceDataForFig5/5C/Tube1Flox:Flox/EMBOR-2023-58207V1_SourceDataForFig5CMid_KATNAL1.png]

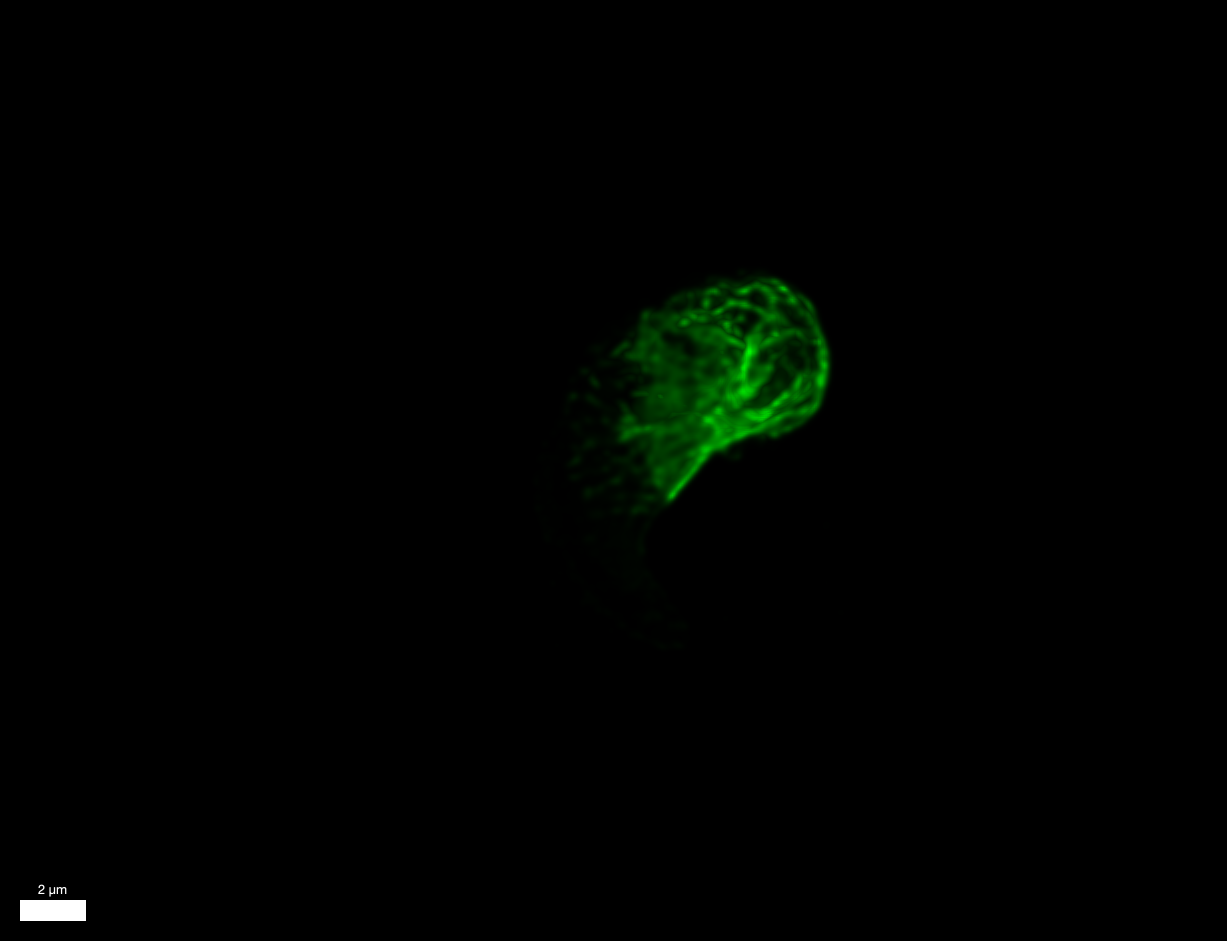

Supplement: Supplementary file 5 — Source data Fig. 5 [file 44319_2024_159_MOESM5_ESM.zip › EMBOR-2023-58207V1_SourceDataForFig5/5C/Tube1Flox:Flox/EMBOR-2023-58207V1_SourceDataForFig5CLate_alpha tubulin.png]

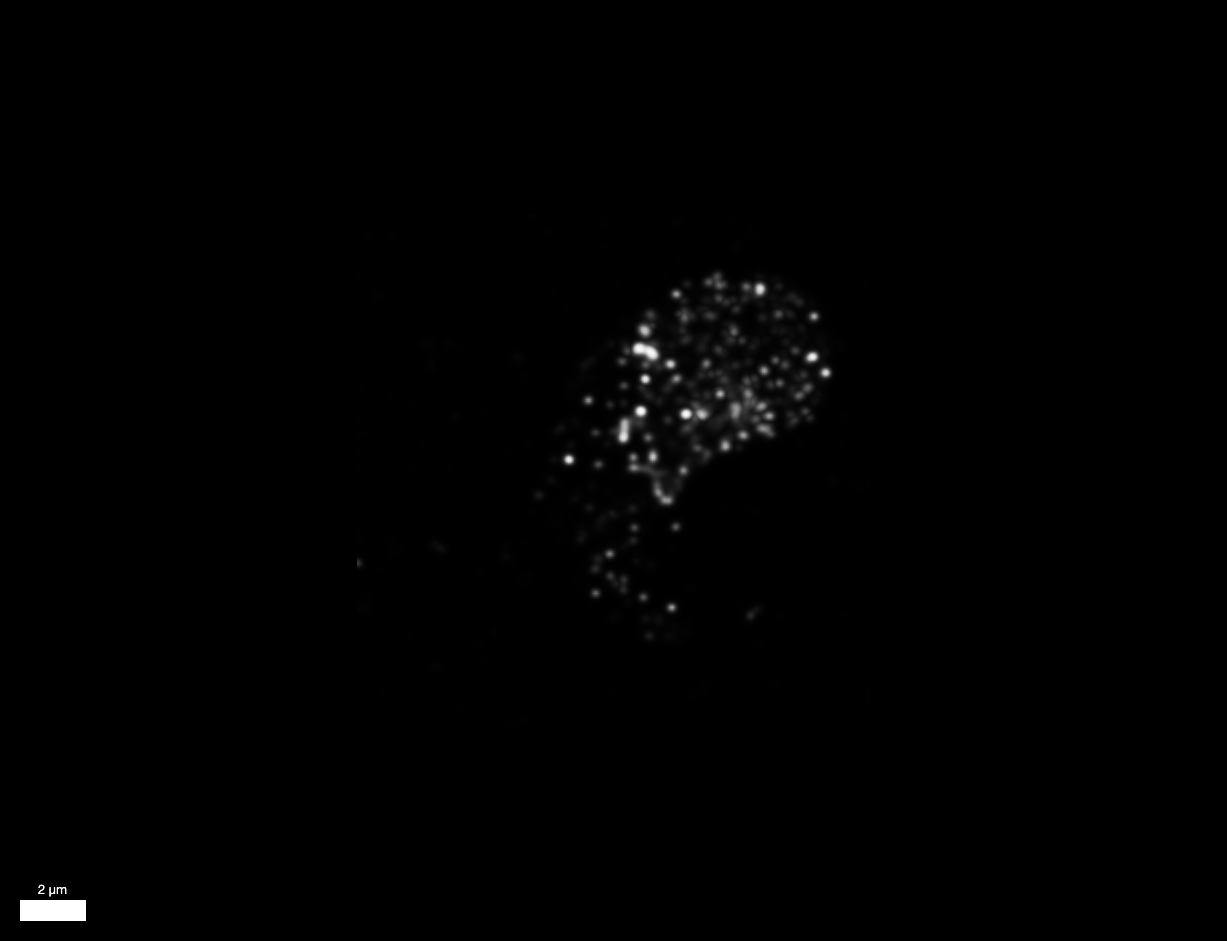

Supplement: Supplementary file 5 — Source data Fig. 5 [file 44319_2024_159_MOESM5_ESM.zip › EMBOR-2023-58207V1_SourceDataForFig5/5C/Tube1Flox:Flox/EMBOR-2023-58207V1_SourceDataForFig5CLate_KATNAL1.png]

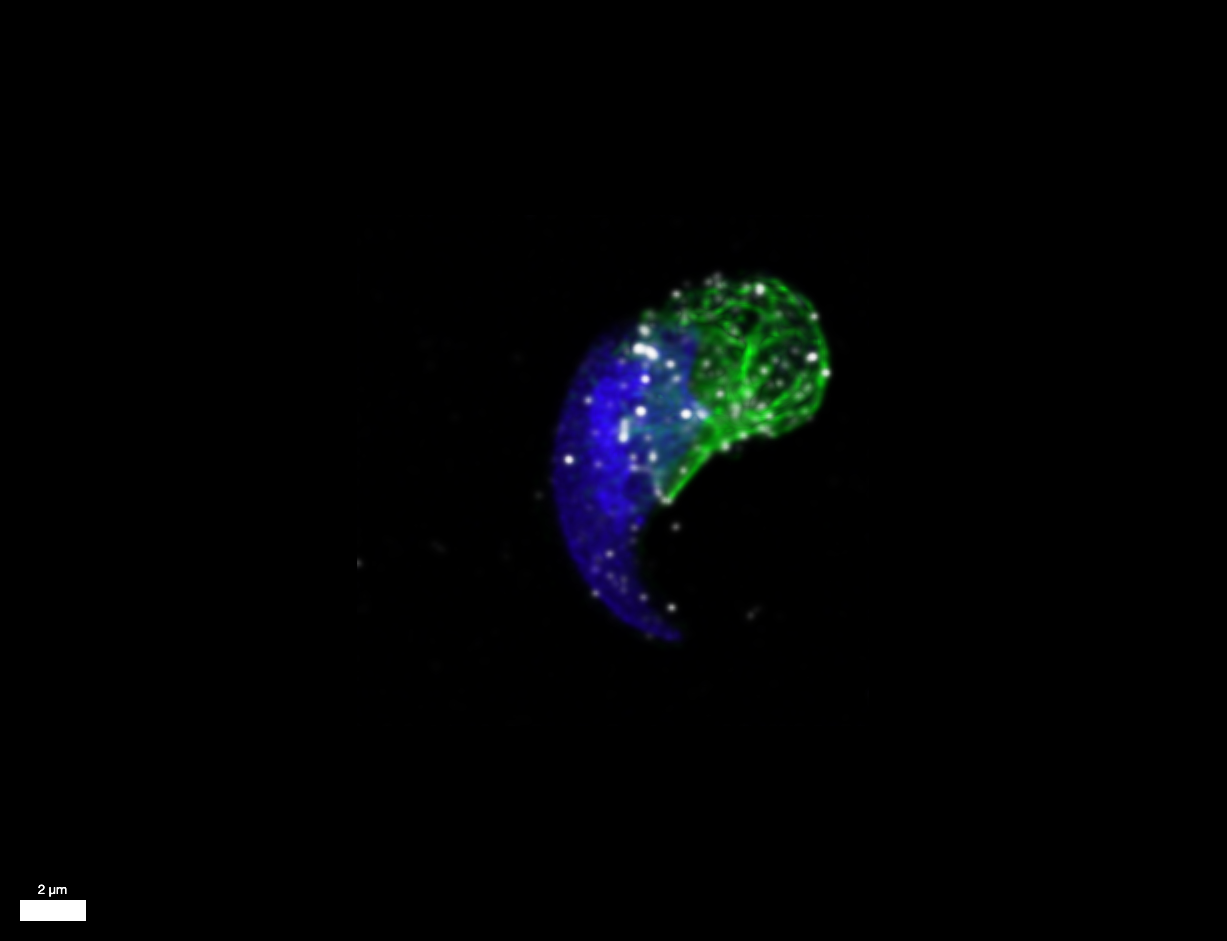

Supplement: Supplementary file 5 — Source data Fig. 5 [file 44319_2024_159_MOESM5_ESM.zip › EMBOR-2023-58207V1_SourceDataForFig5/5C/Tube1Flox:Flox/EMBOR-2023-58207V1_SourceDataForFig5CLate_DAPI:Merged.tif]

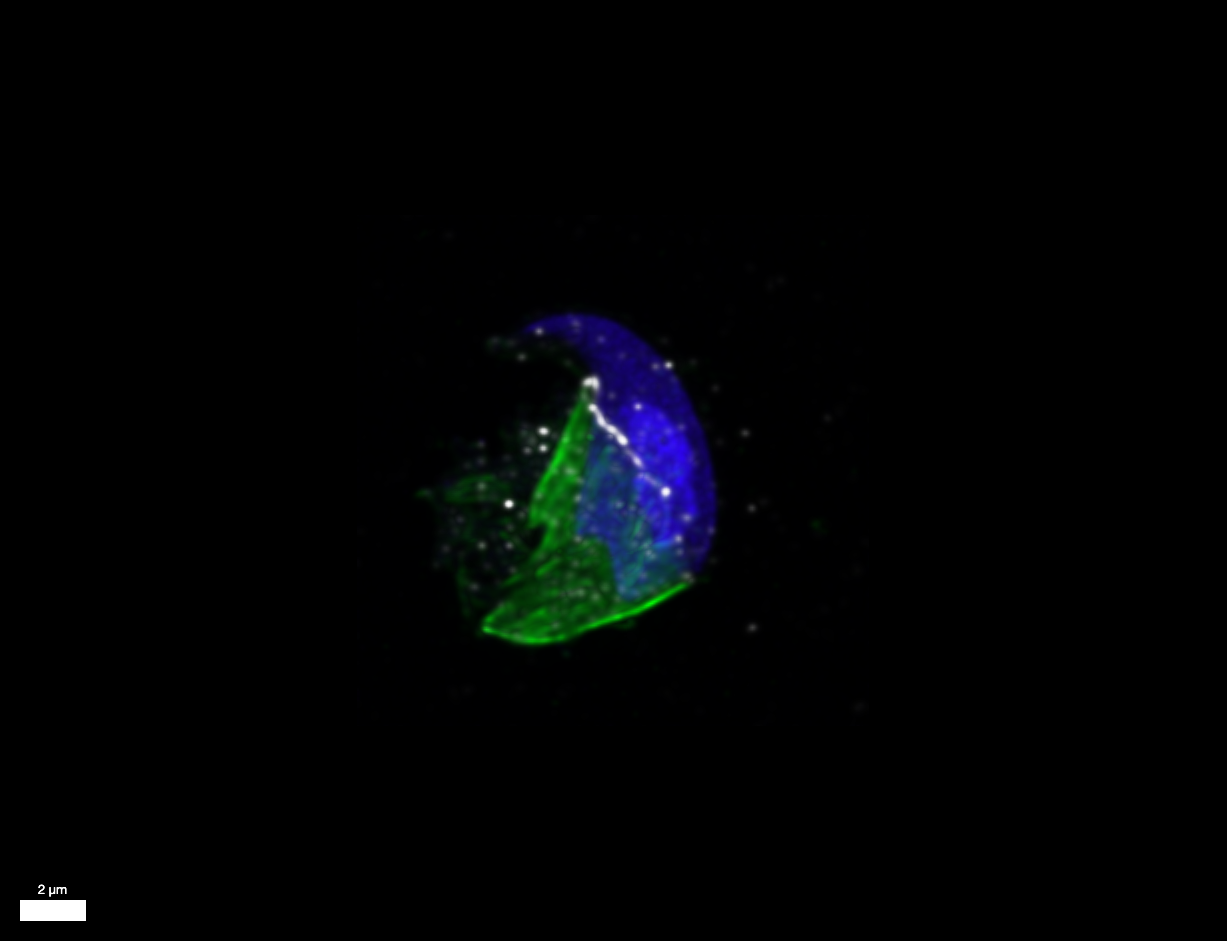

Supplement: Supplementary file 5 — Source data Fig. 5 [file 44319_2024_159_MOESM5_ESM.zip › EMBOR-2023-58207V1_SourceDataForFig5/5C/Tube1Flox:Flox/EMBOR-2023-58207V1_SourceDataForFig5CMid_DAPI:Merged.tif]

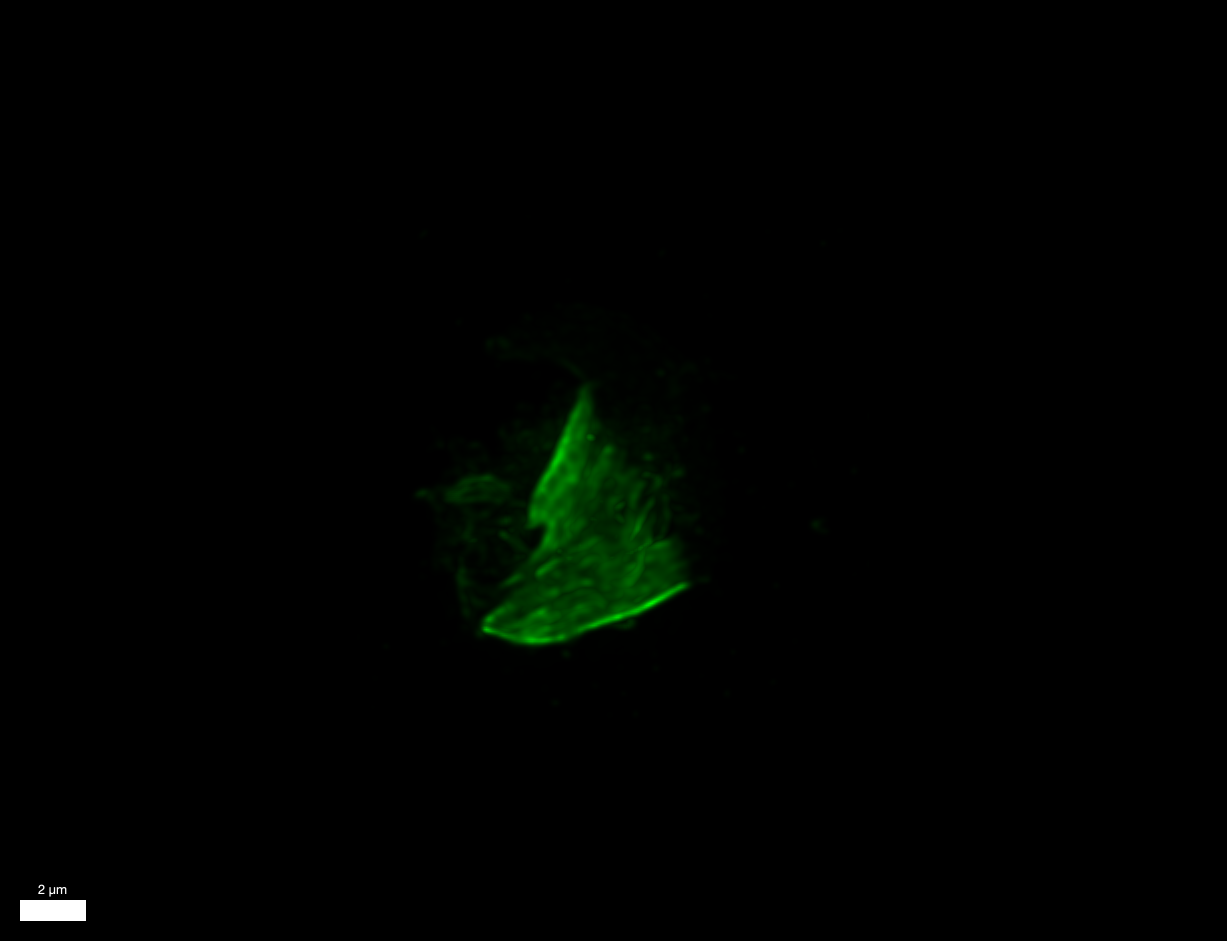

Supplement: Supplementary file 5 — Source data Fig. 5 [file 44319_2024_159_MOESM5_ESM.zip › EMBOR-2023-58207V1_SourceDataForFig5/5C/Tube1Flox:Flox/EMBOR-2023-58207V1_SourceDataForFig5CMid_alpha tubulin.png]

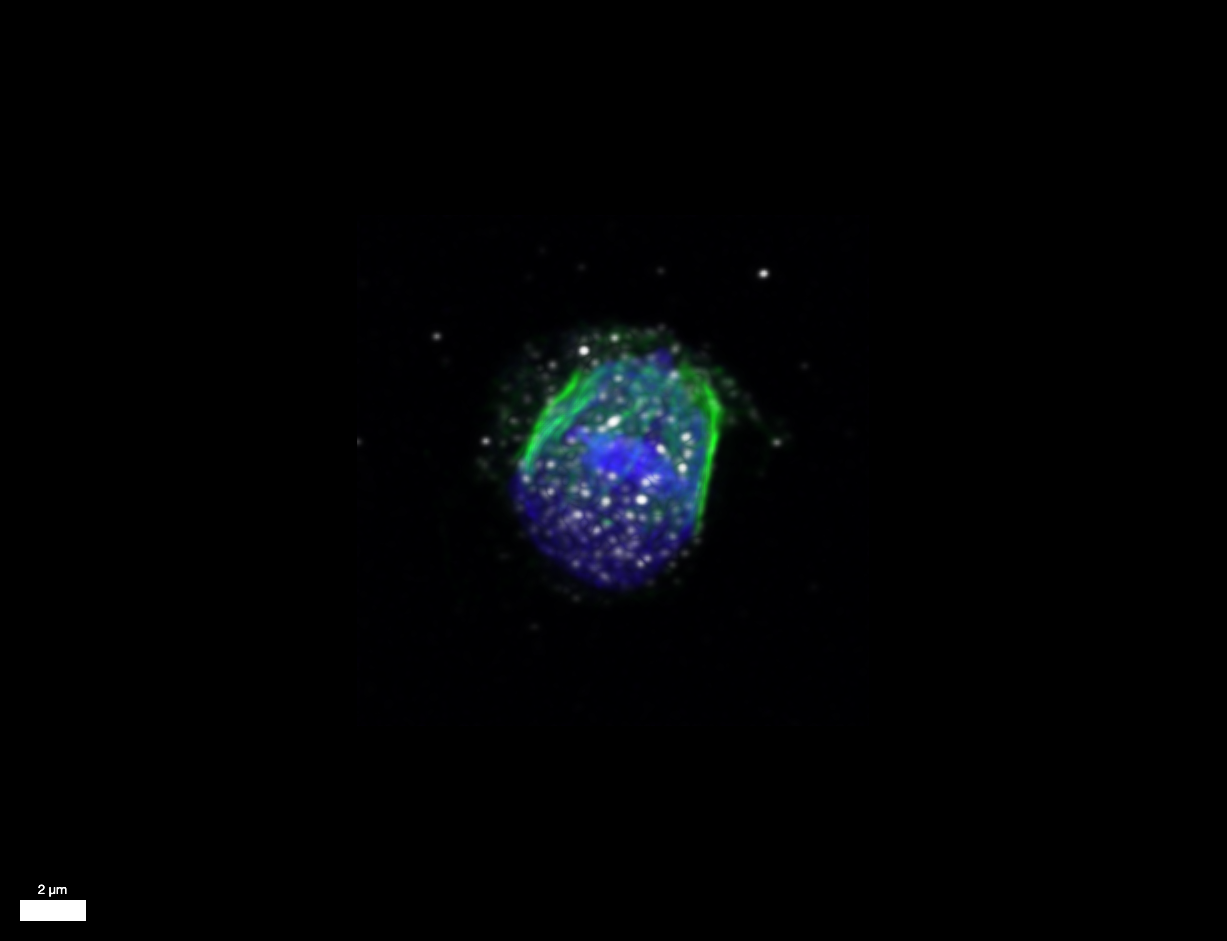

Supplement: Supplementary file 5 — Source data Fig. 5 [file 44319_2024_159_MOESM5_ESM.zip › EMBOR-2023-58207V1_SourceDataForFig5/5C/Tube1Flox:Flox/EMBOR-2023-58207V1_SourceDataForFig5CEarly_DAPI:Merged.tif]
