# Supplementary material for: Epsilon tubulin is an essential determinant of microtubule-based structures in male germ cells
Source: EMBO Rep. 2024 May 21;25(6):14. doi: 10.1038/s44319-024-00159-w (PMC11169422; doi:10.1038/s44319-024-00159-w)
Supplement: Supplementary file 2 — Source data Fig. 2 [file 44319_2024_159_MOESM2_ESM.zip › EMBOR-2023-58207V1_SourceDataForFig2/README_Fig. 2D-H.rtf]

Image brightness and contrast was uniformly adjusted in Adobe Photoshop and images were rotated and cropped to show only one cell in each final figure.
