# Supplementary material for: Epsilon tubulin is an essential determinant of microtubule-based structures in male germ cells
Source: EMBO Rep. 2024 May 21;25(6):14. doi: 10.1038/s44319-024-00159-w (PMC11169422; doi:10.1038/s44319-024-00159-w)
Supplement: Supplementary file 4 — Source data Fig. 4 [file 44319_2024_159_MOESM4_ESM.zip › EMBOR-2023-58207V1_SourceDataForFig4/README_Fig. 4.rtf]

Images were rotated and/or reflected during arrangement for consistency across images within each panel and a white balance was applied in Adobe Photoshop. Each image was cropped to display one region of interest in the field of view.
