# Supplementary figures and images for: Epsilon tubulin is an essential determinant of microtubule-based structures in male germ cells (part 2 of 2)
Source: EMBO Rep. 2024 May 21;25(6):14. doi: 10.1038/s44319-024-00159-w (PMC11169422; doi:10.1038/s44319-024-00159-w)

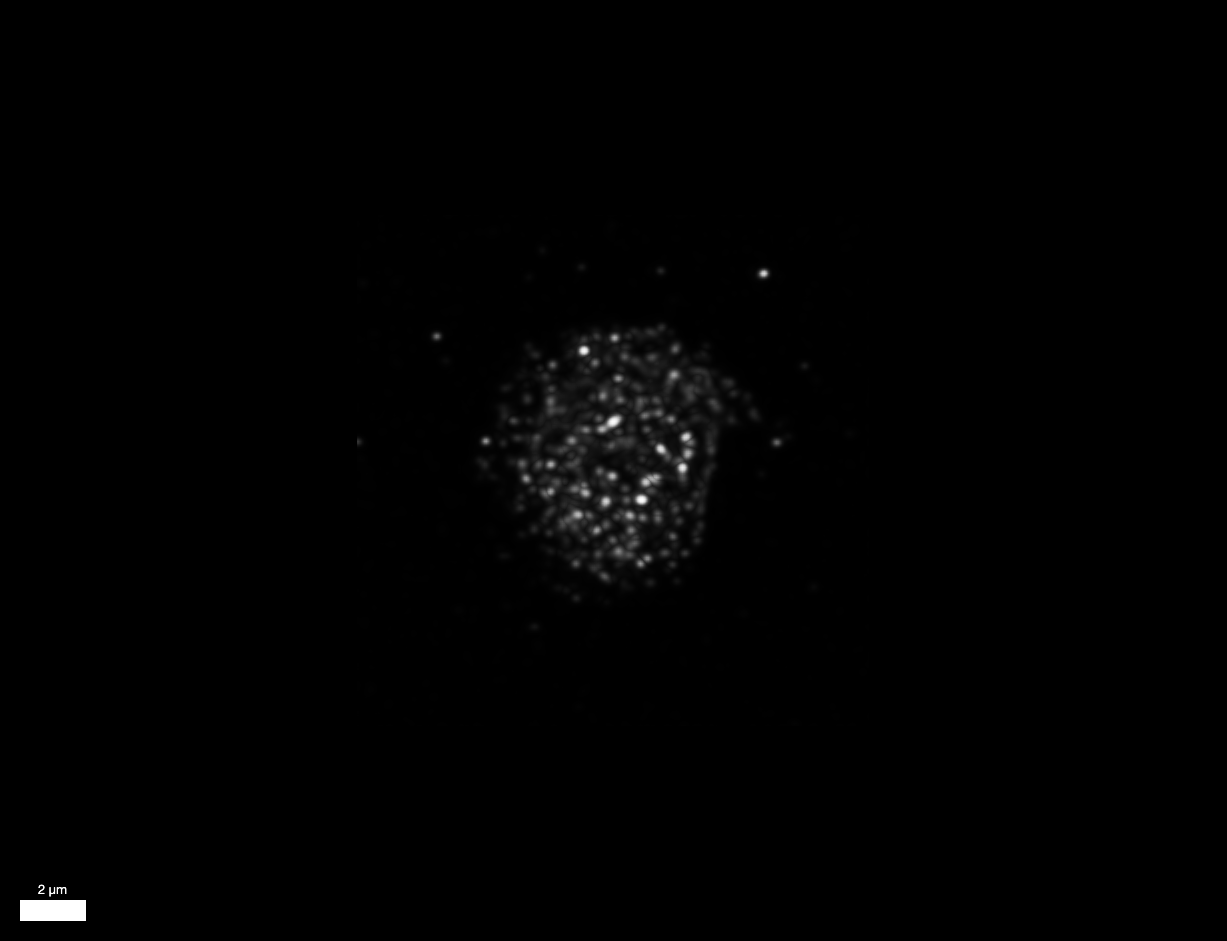

Supplement: Supplementary file 5 — Source data Fig. 5 [file 44319_2024_159_MOESM5_ESM.zip › EMBOR-2023-58207V1_SourceDataForFig5/5C/Tube1Flox:Flox/EMBOR-2023-58207V1_SourceDataForFig5CEarly_KATNAL1.png]

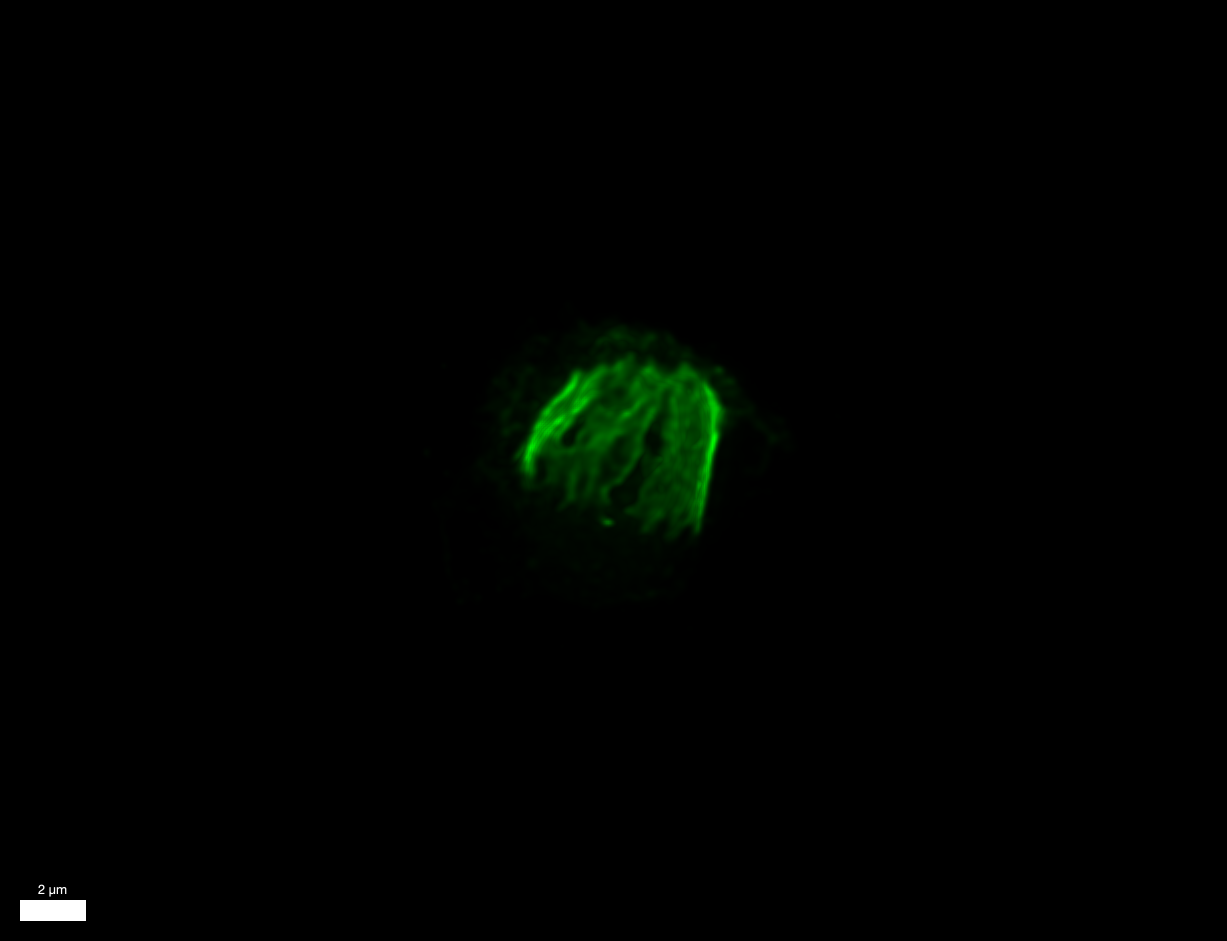

Supplement: Supplementary file 5 — Source data Fig. 5 [file 44319_2024_159_MOESM5_ESM.zip › EMBOR-2023-58207V1_SourceDataForFig5/5C/Tube1Flox:Flox/EMBOR-2023-58207V1_SourceDataForFig5CEarly_alpha tubulin.png]

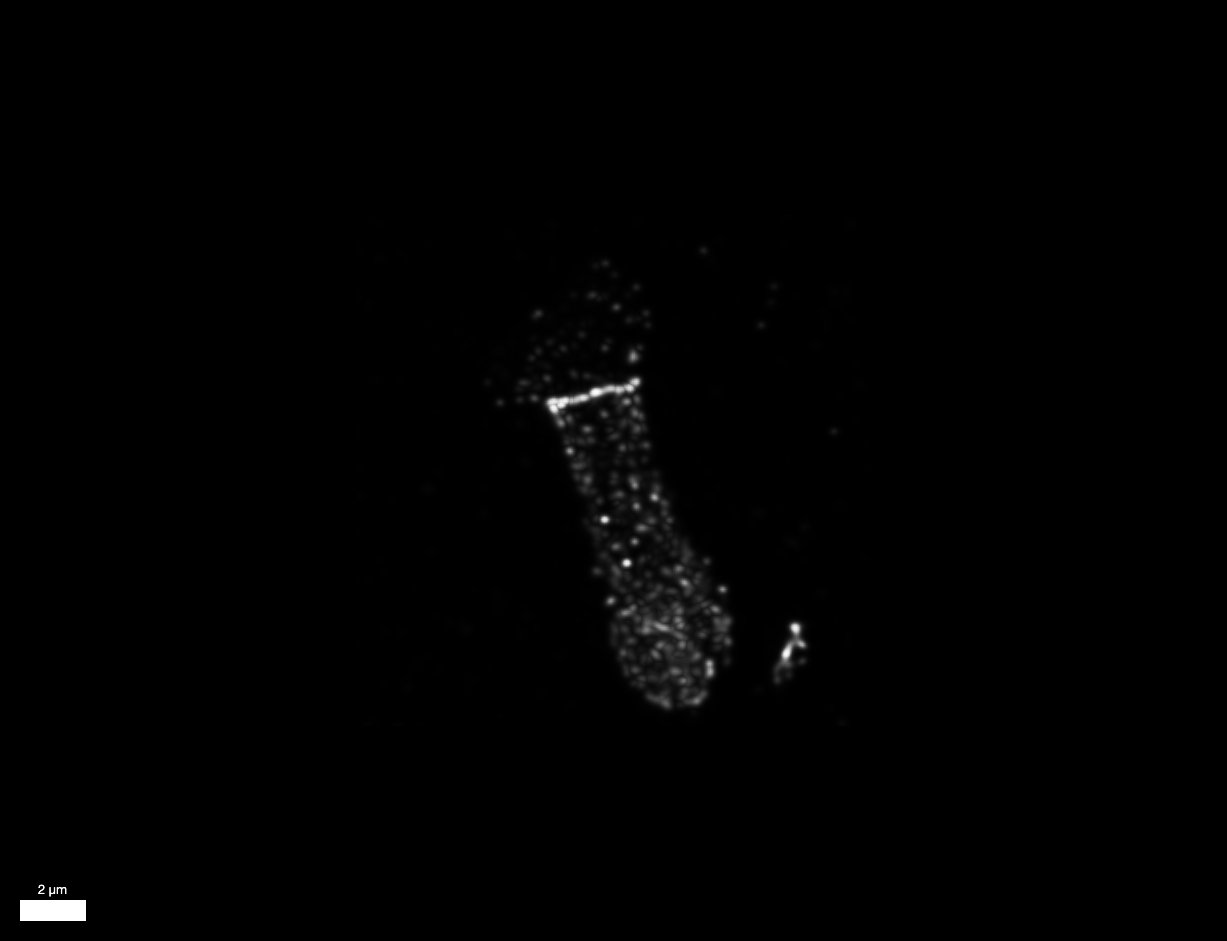

Supplement: Supplementary file 5 — Source data Fig. 5 [file 44319_2024_159_MOESM5_ESM.zip › EMBOR-2023-58207V1_SourceDataForFig5/5C/Tube1GCKO:GCKO/EMBOR-2023-58207V1_SourceDataForFig5CMid_KATNAL1.png]

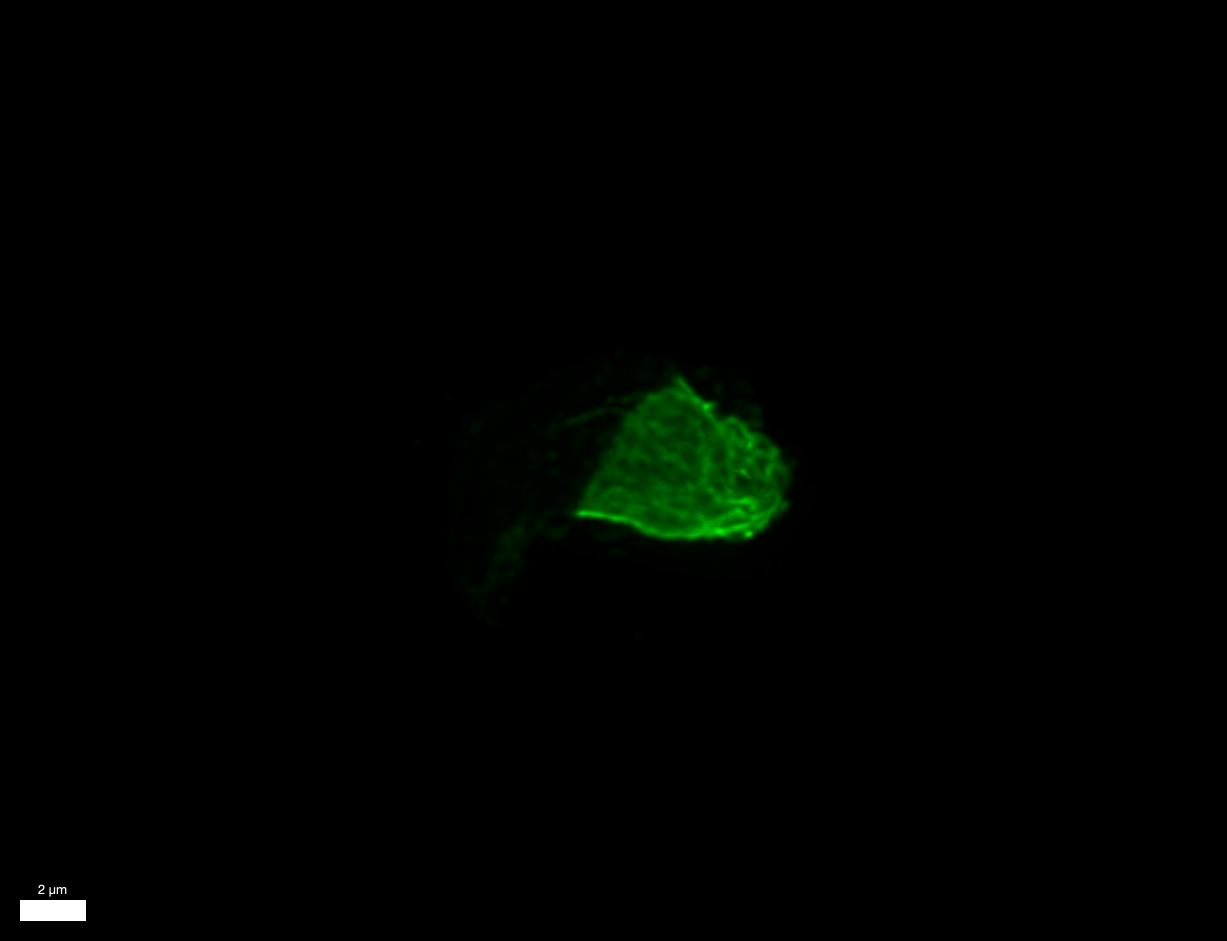

Supplement: Supplementary file 5 — Source data Fig. 5 [file 44319_2024_159_MOESM5_ESM.zip › EMBOR-2023-58207V1_SourceDataForFig5/5C/Tube1GCKO:GCKO/EMBOR-2023-58207V1_SourceDataForFig5CLate_alpha tubulin.png]

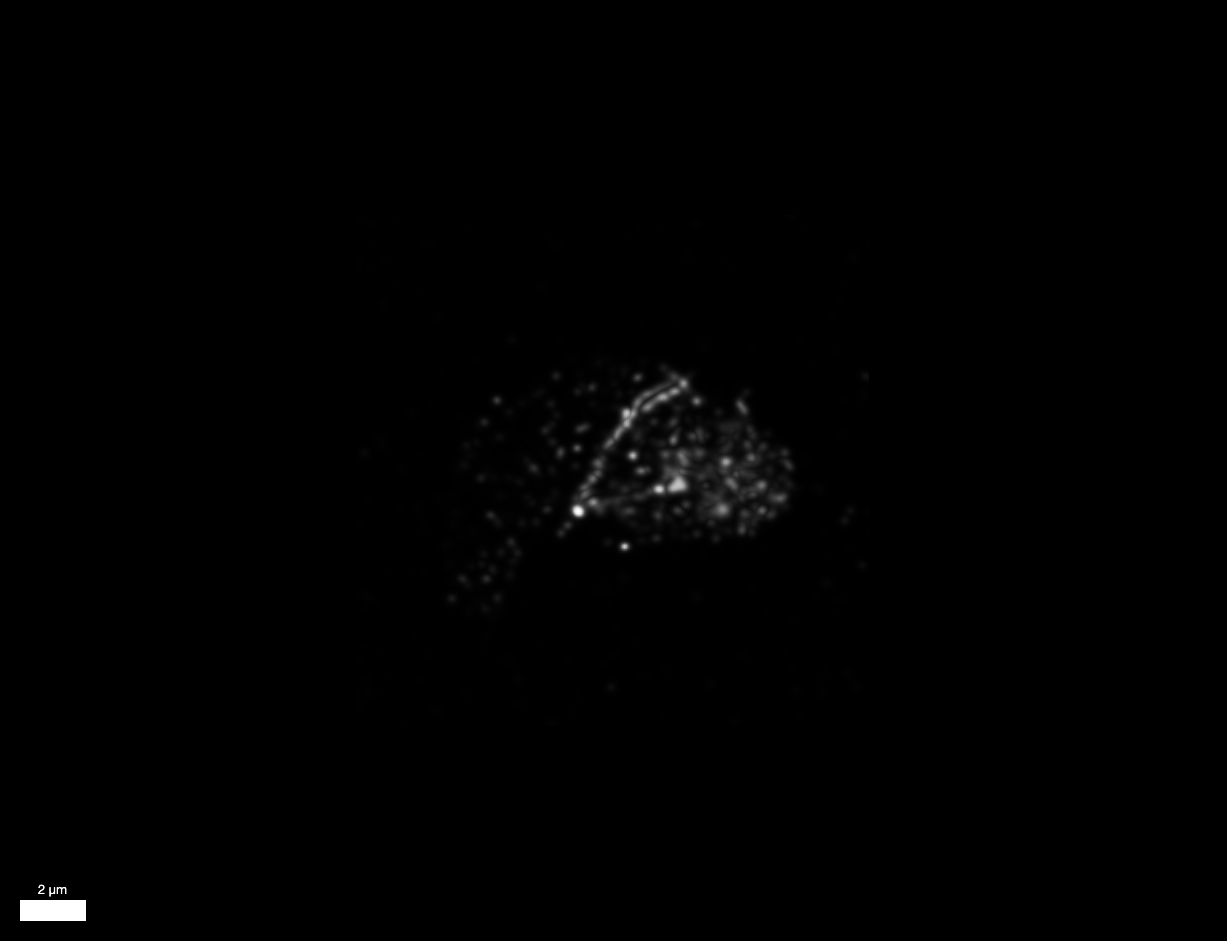

Supplement: Supplementary file 5 — Source data Fig. 5 [file 44319_2024_159_MOESM5_ESM.zip › EMBOR-2023-58207V1_SourceDataForFig5/5C/Tube1GCKO:GCKO/EMBOR-2023-58207V1_SourceDataForFig5CLate_KATNAL1.png]

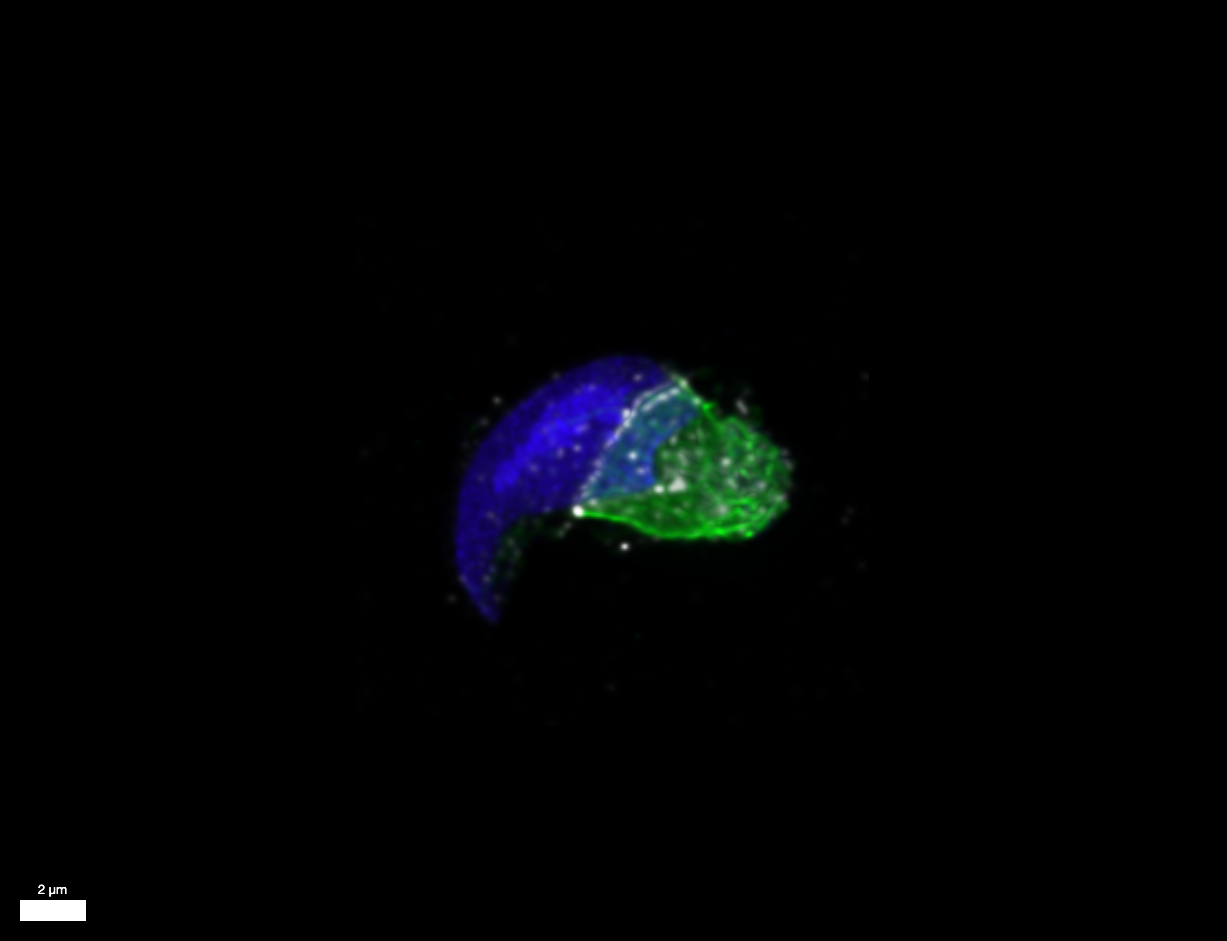

Supplement: Supplementary file 5 — Source data Fig. 5 [file 44319_2024_159_MOESM5_ESM.zip › EMBOR-2023-58207V1_SourceDataForFig5/5C/Tube1GCKO:GCKO/EMBOR-2023-58207V1_SourceDataForFig5CLate_DAPI:Merged.tif]

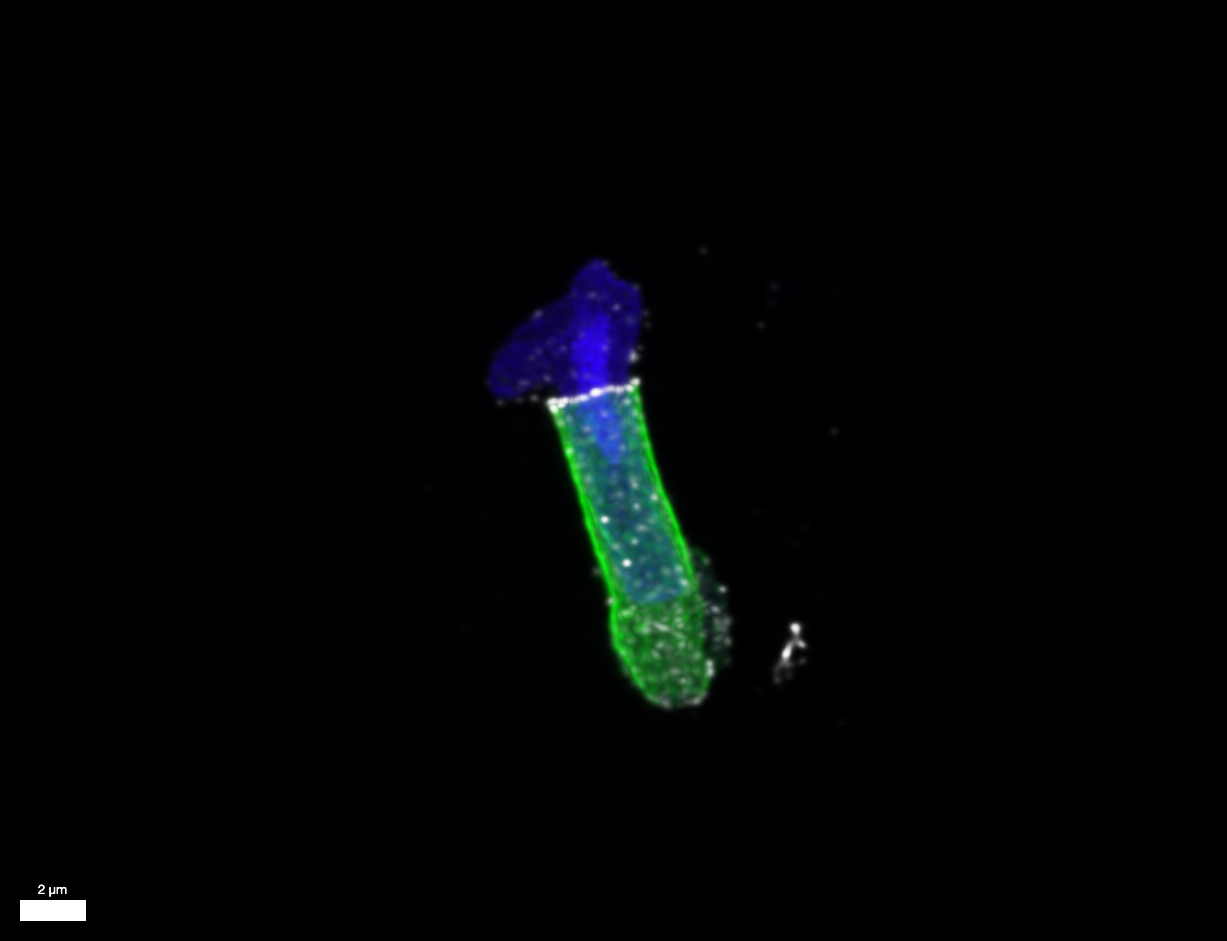

Supplement: Supplementary file 5 — Source data Fig. 5 [file 44319_2024_159_MOESM5_ESM.zip › EMBOR-2023-58207V1_SourceDataForFig5/5C/Tube1GCKO:GCKO/EMBOR-2023-58207V1_SourceDataForFig5CMid_DAPI:Merged.tif]

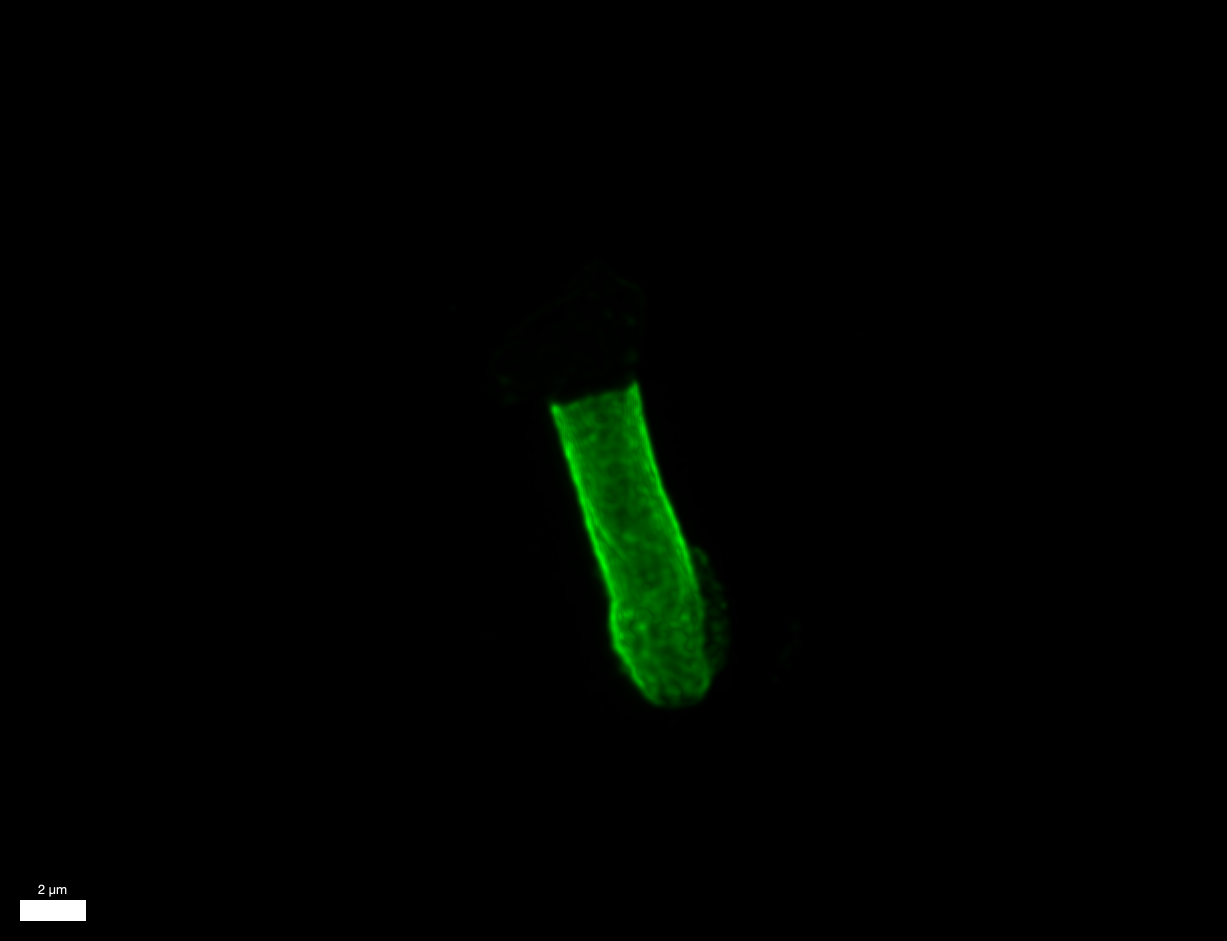

Supplement: Supplementary file 5 — Source data Fig. 5 [file 44319_2024_159_MOESM5_ESM.zip › EMBOR-2023-58207V1_SourceDataForFig5/5C/Tube1GCKO:GCKO/EMBOR-2023-58207V1_SourceDataForFig5CMid_alpha tubulin.png]

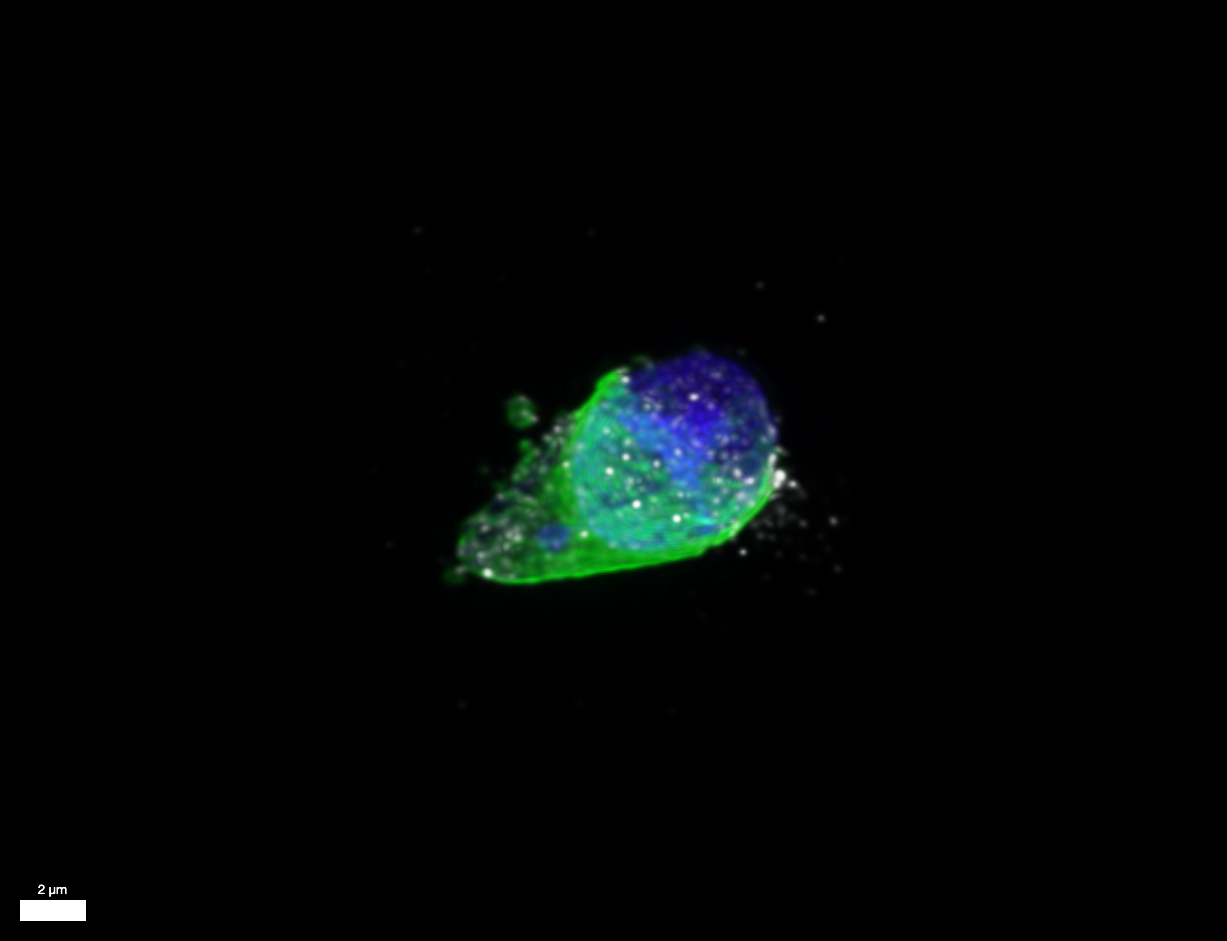

Supplement: Supplementary file 5 — Source data Fig. 5 [file 44319_2024_159_MOESM5_ESM.zip › EMBOR-2023-58207V1_SourceDataForFig5/5C/Tube1GCKO:GCKO/EMBOR-2023-58207V1_SourceDataForFig5CEarly_DAPI:Merged.tif]

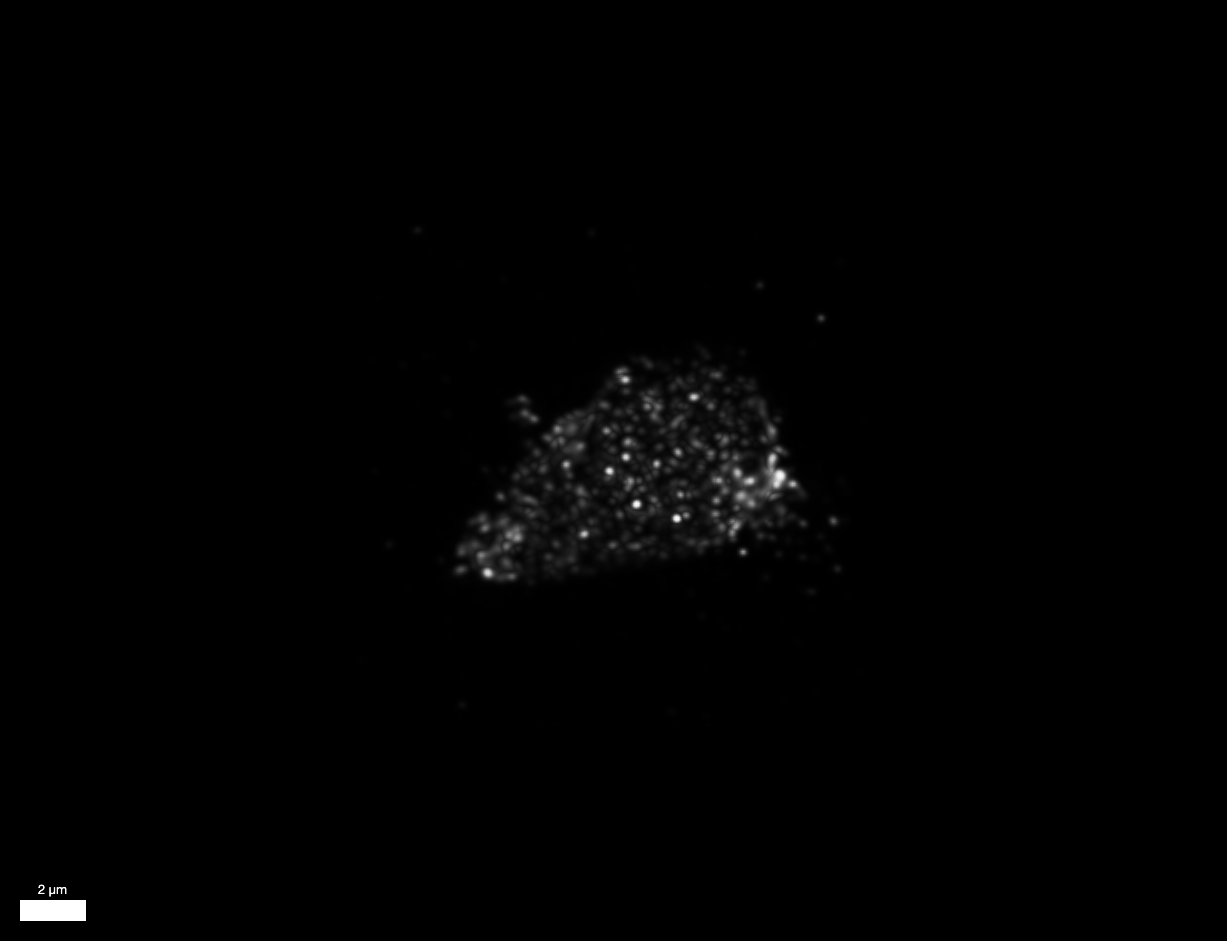

Supplement: Supplementary file 5 — Source data Fig. 5 [file 44319_2024_159_MOESM5_ESM.zip › EMBOR-2023-58207V1_SourceDataForFig5/5C/Tube1GCKO:GCKO/EMBOR-2023-58207V1_SourceDataForFig5CEarly_KATNAL1.png]

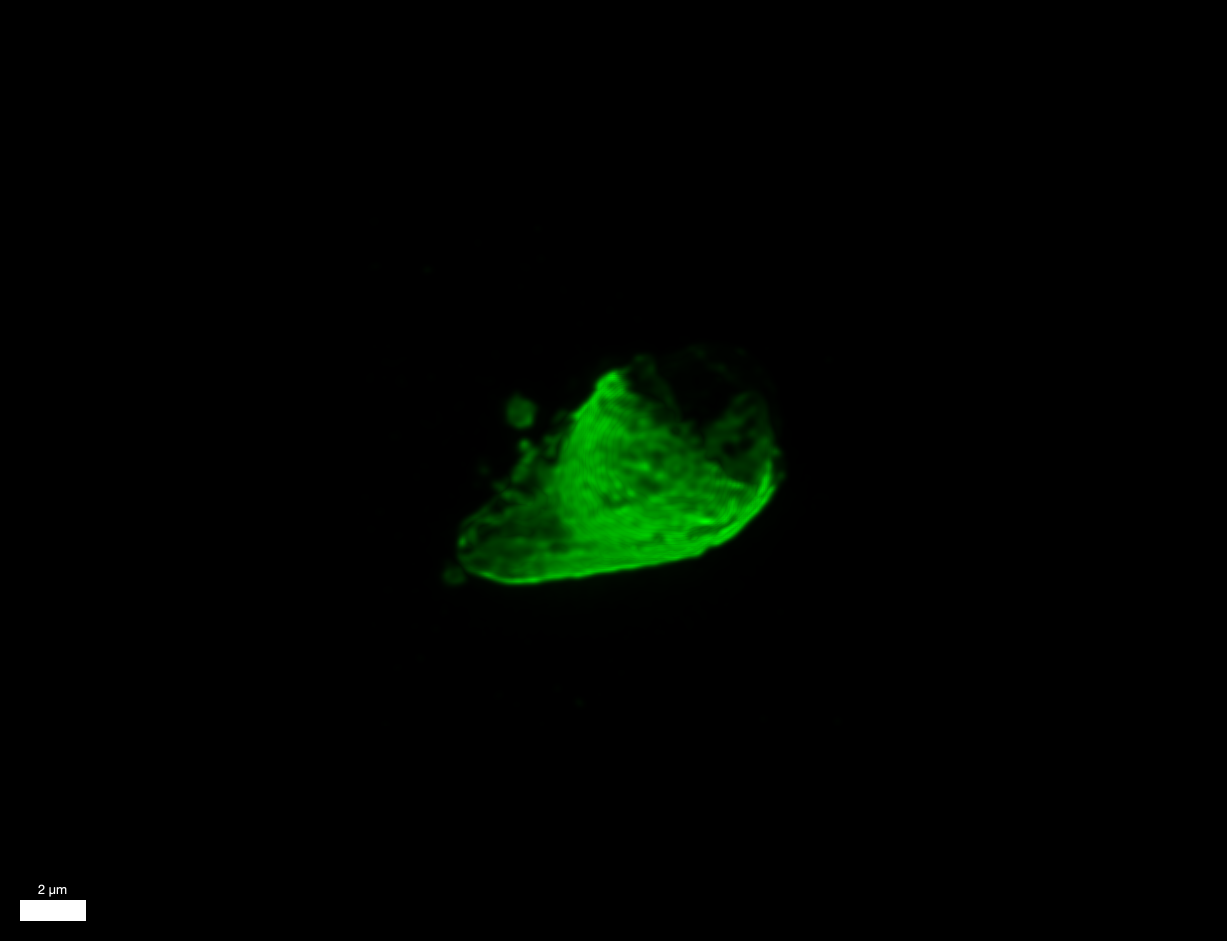

Supplement: Supplementary file 5 — Source data Fig. 5 [file 44319_2024_159_MOESM5_ESM.zip › EMBOR-2023-58207V1_SourceDataForFig5/5C/Tube1GCKO:GCKO/EMBOR-2023-58207V1_SourceDataForFig5CEarly_alpha tubulin.png]

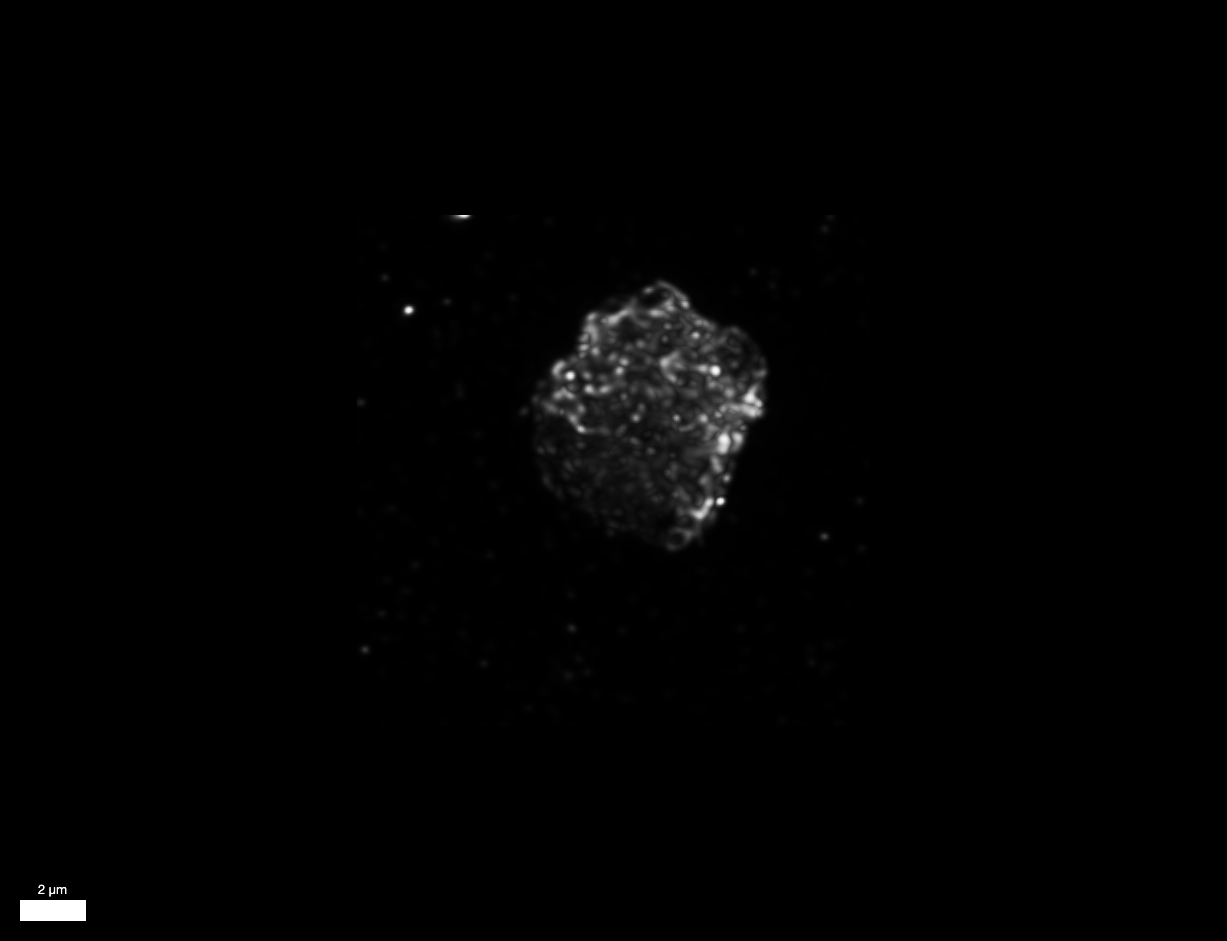

Supplement: Supplementary file 5 — Source data Fig. 5 [file 44319_2024_159_MOESM5_ESM.zip › EMBOR-2023-58207V1_SourceDataForFig5/5E/Tube1Flox:Flox/EMBOR-2023-58207V1_SourceDataForFig5EEarly_KATNAL2.png]

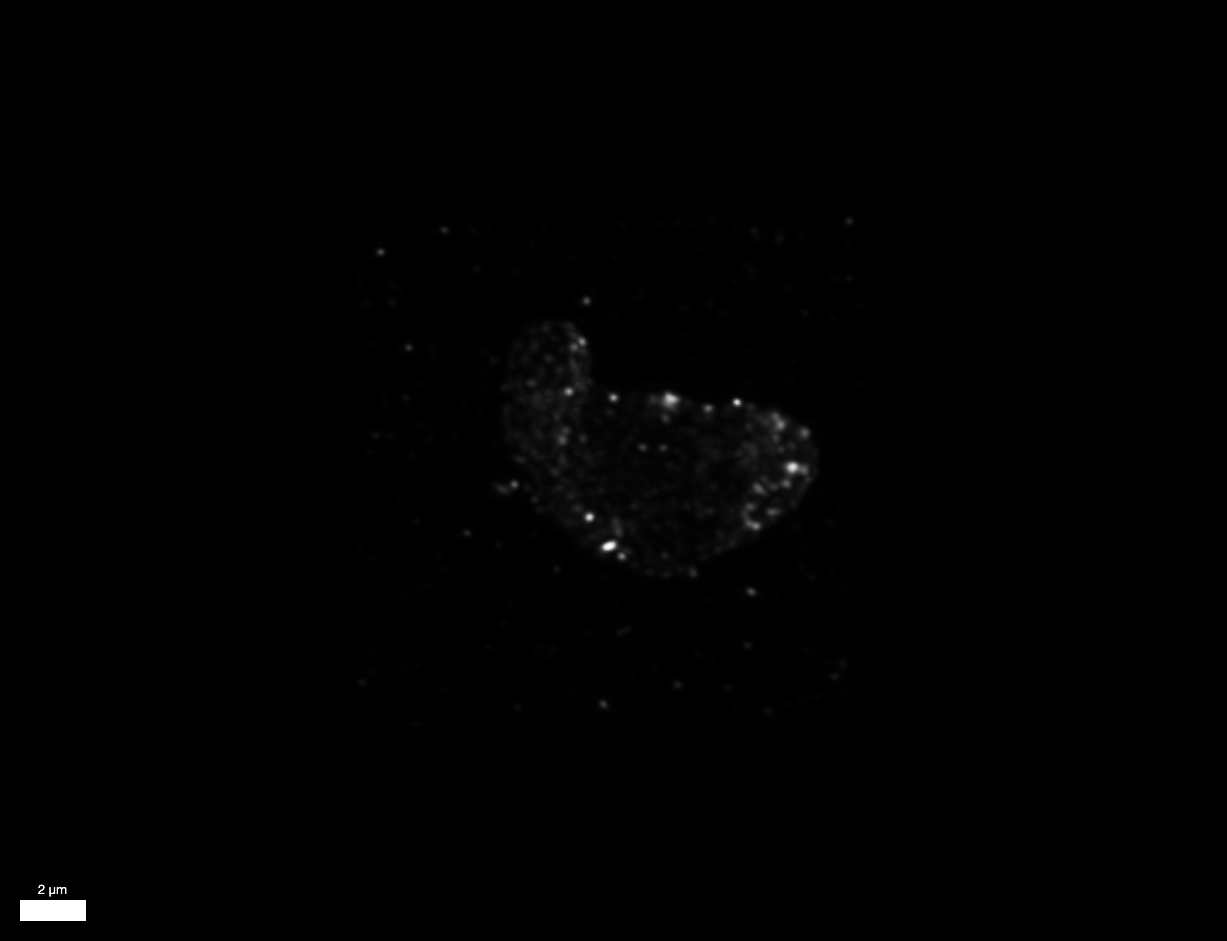

Supplement: Supplementary file 5 — Source data Fig. 5 [file 44319_2024_159_MOESM5_ESM.zip › EMBOR-2023-58207V1_SourceDataForFig5/5E/Tube1Flox:Flox/EMBOR-2023-58207V1_SourceDataForFig5EMid_KATNAL2.png]

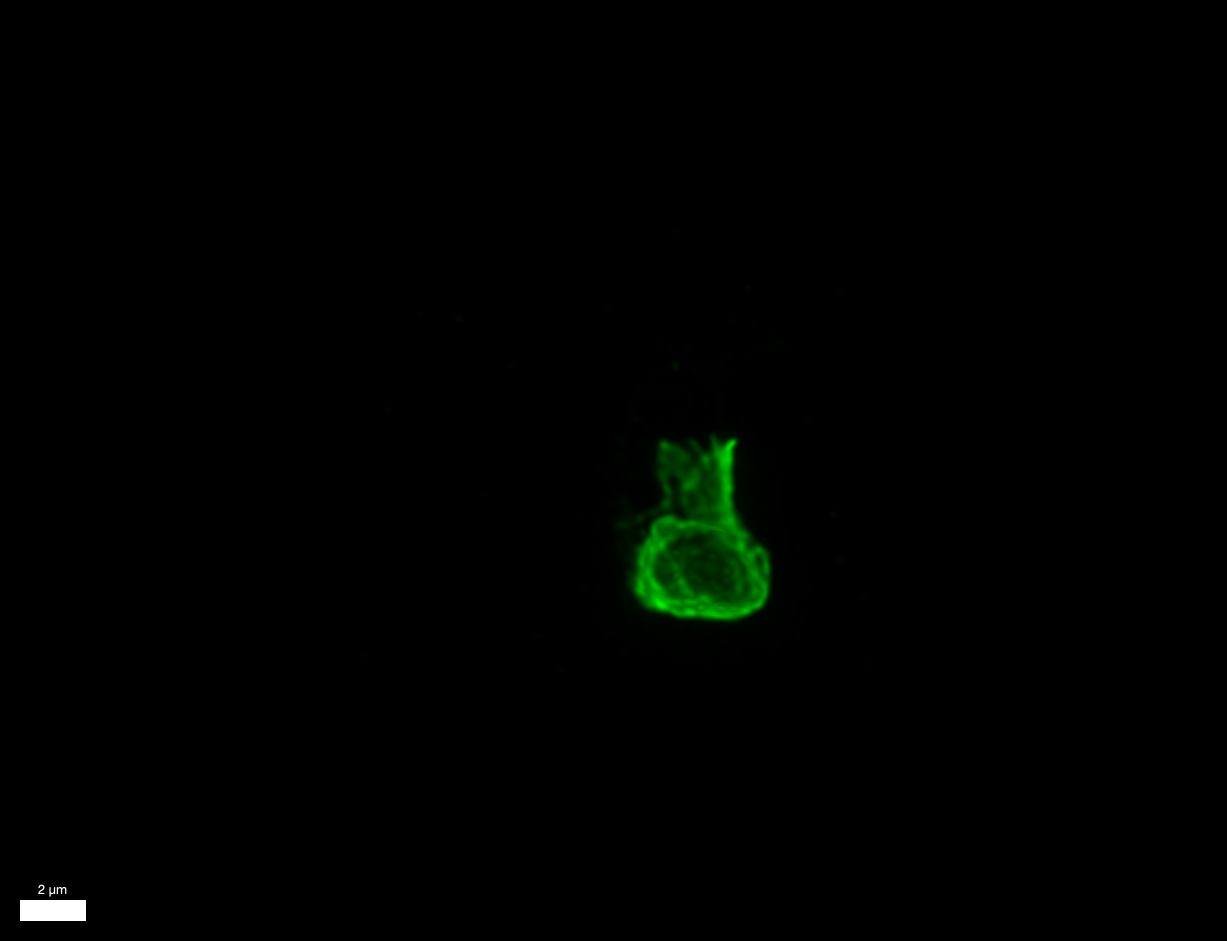

Supplement: Supplementary file 5 — Source data Fig. 5 [file 44319_2024_159_MOESM5_ESM.zip › EMBOR-2023-58207V1_SourceDataForFig5/5E/Tube1Flox:Flox/EMBOR-2023-58207V1_SourceDataForFig5ELate_alpha tubulin.png]

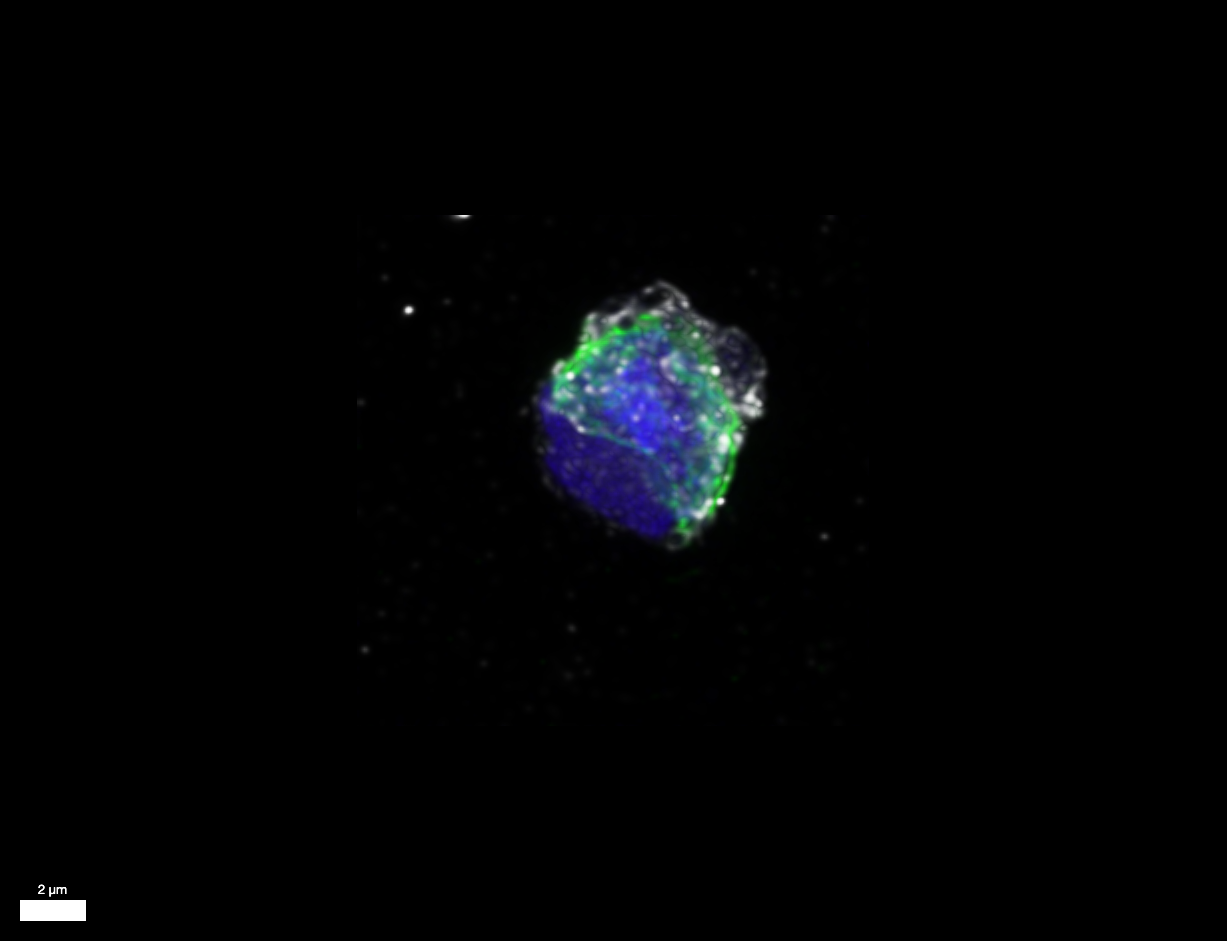

Supplement: Supplementary file 5 — Source data Fig. 5 [file 44319_2024_159_MOESM5_ESM.zip › EMBOR-2023-58207V1_SourceDataForFig5/5E/Tube1Flox:Flox/EMBOR-2023-58207V1_SourceDataForFig5EEarly_DAPI:Merged.tif]

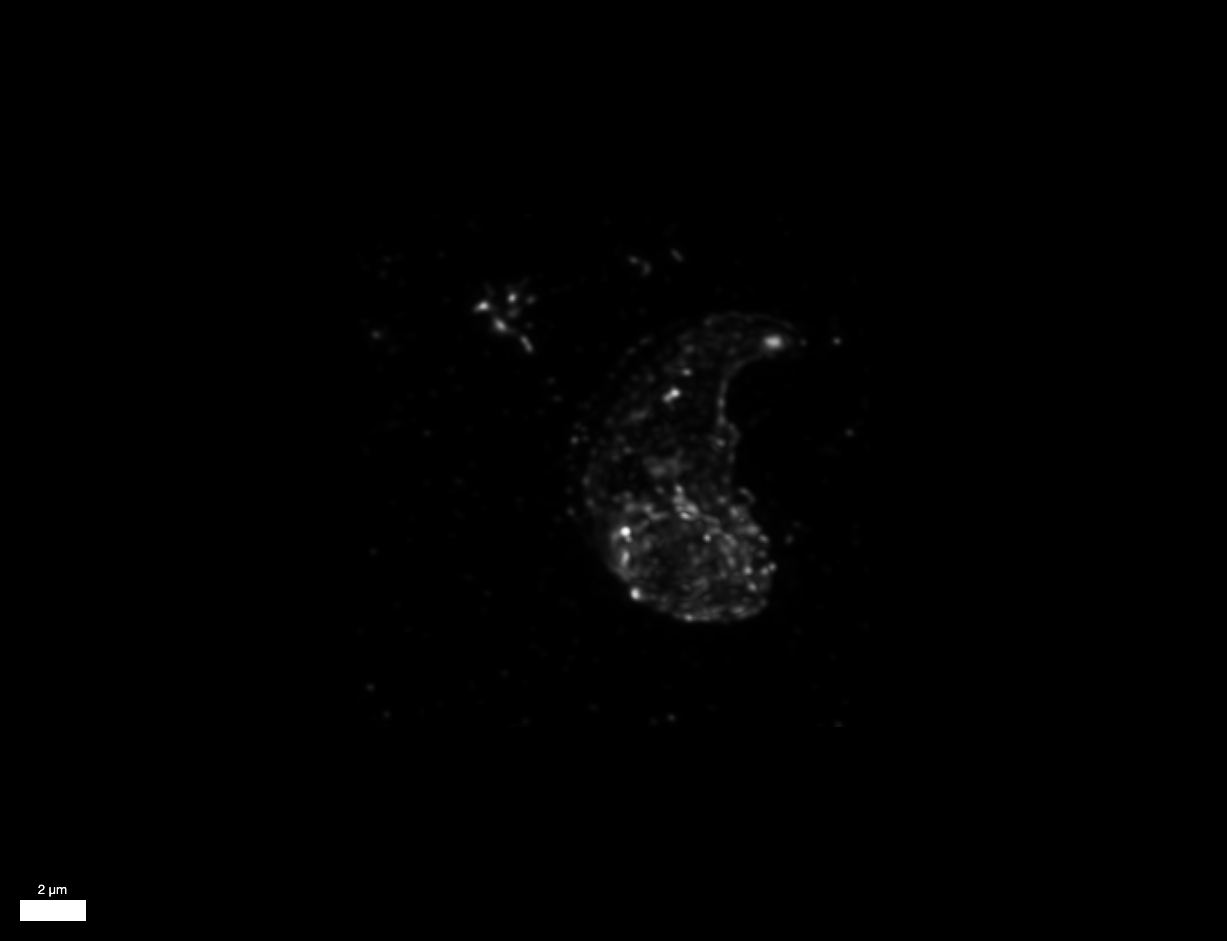

Supplement: Supplementary file 5 — Source data Fig. 5 [file 44319_2024_159_MOESM5_ESM.zip › EMBOR-2023-58207V1_SourceDataForFig5/5E/Tube1Flox:Flox/EMBOR-2023-58207V1_SourceDataForFig5ELate_KATNAL2.png]

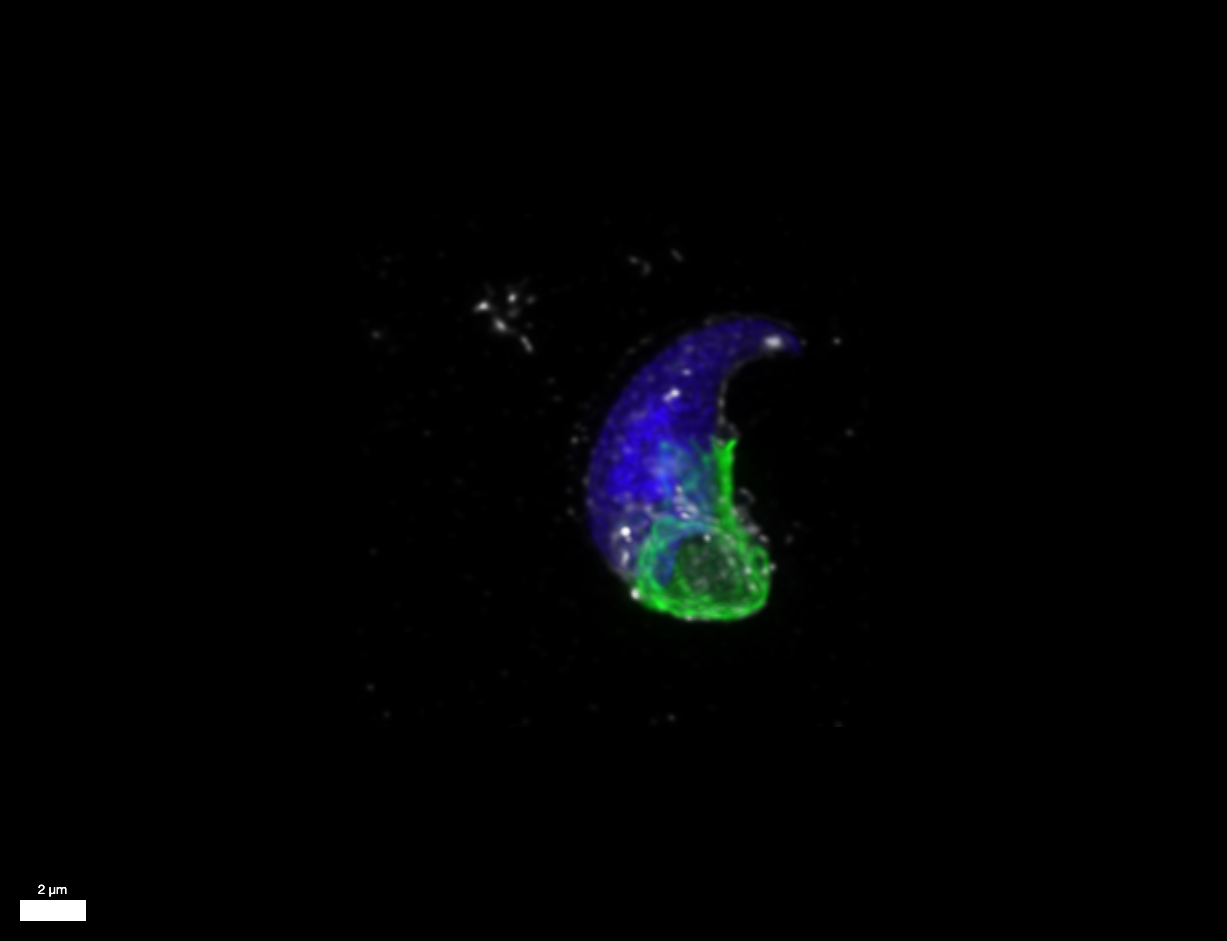

Supplement: Supplementary file 5 — Source data Fig. 5 [file 44319_2024_159_MOESM5_ESM.zip › EMBOR-2023-58207V1_SourceDataForFig5/5E/Tube1Flox:Flox/EMBOR-2023-58207V1_SourceDataForFig5ELate_DAPI:Merged.tif]

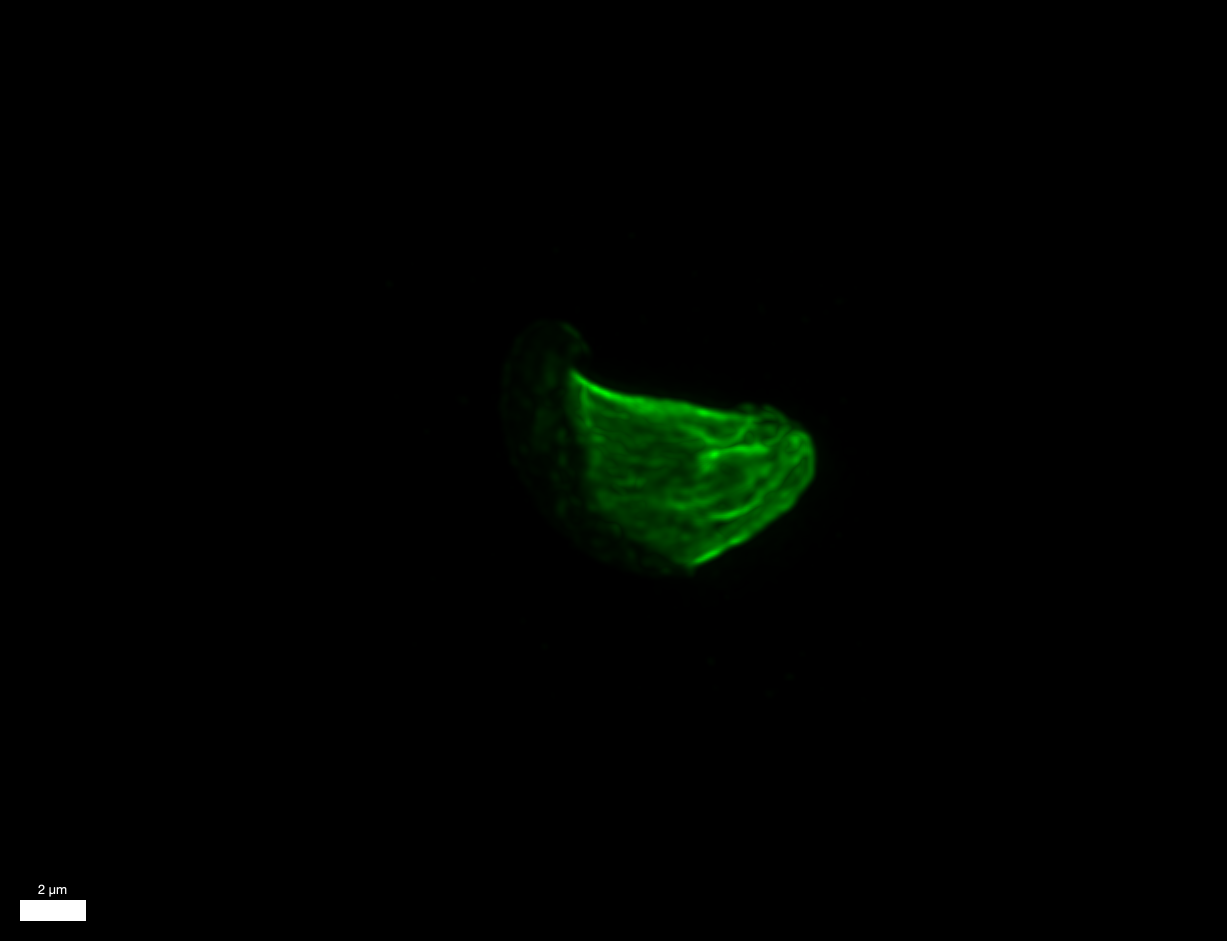

Supplement: Supplementary file 5 — Source data Fig. 5 [file 44319_2024_159_MOESM5_ESM.zip › EMBOR-2023-58207V1_SourceDataForFig5/5E/Tube1Flox:Flox/EMBOR-2023-58207V1_SourceDataForFig5EMid_alpha tubulin.png]

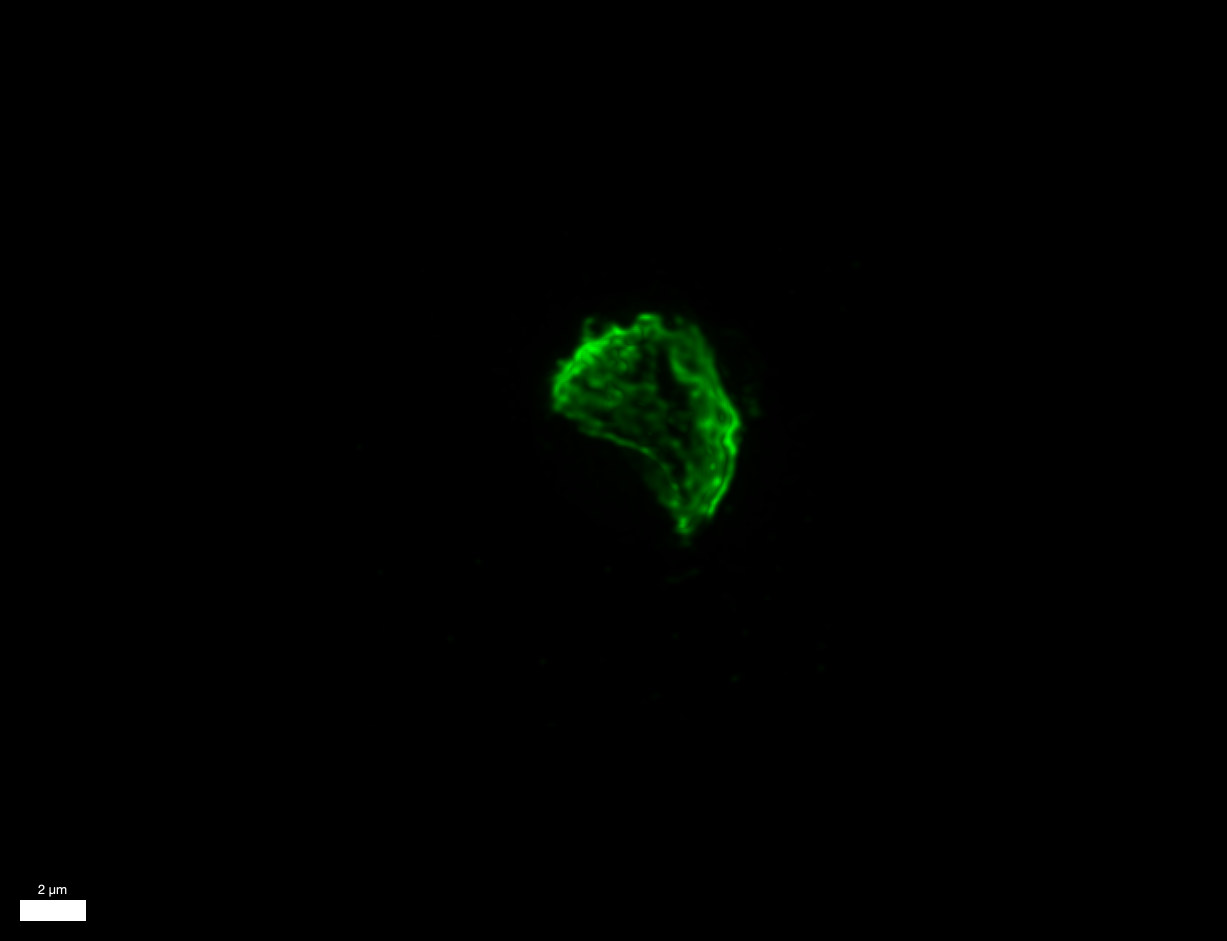

Supplement: Supplementary file 5 — Source data Fig. 5 [file 44319_2024_159_MOESM5_ESM.zip › EMBOR-2023-58207V1_SourceDataForFig5/5E/Tube1Flox:Flox/EMBOR-2023-58207V1_SourceDataForFig5EEarly_alpha tubulin.png]

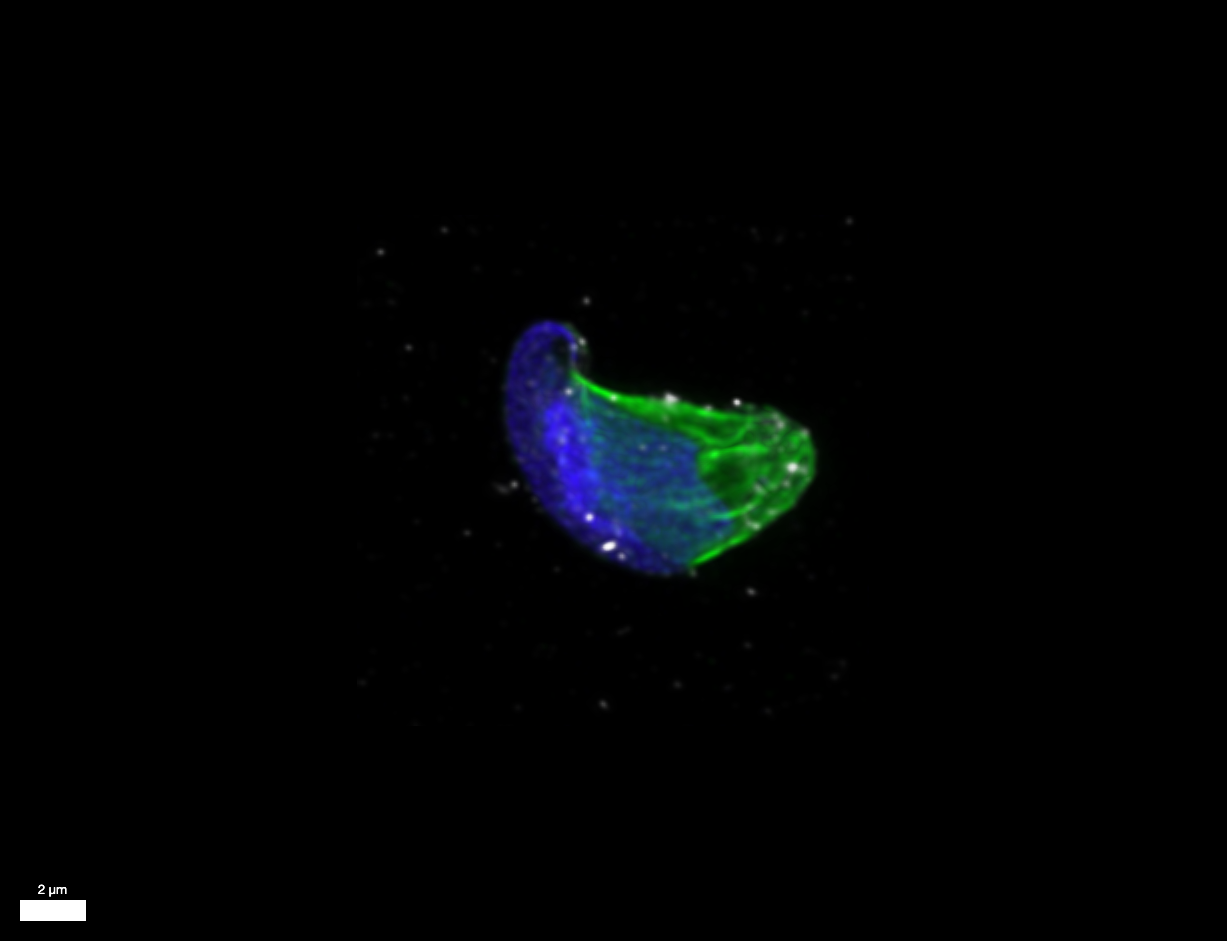

Supplement: Supplementary file 5 — Source data Fig. 5 [file 44319_2024_159_MOESM5_ESM.zip › EMBOR-2023-58207V1_SourceDataForFig5/5E/Tube1Flox:Flox/EMBOR-2023-58207V1_SourceDataForFig53Mid_DAPI:Merged.tif]

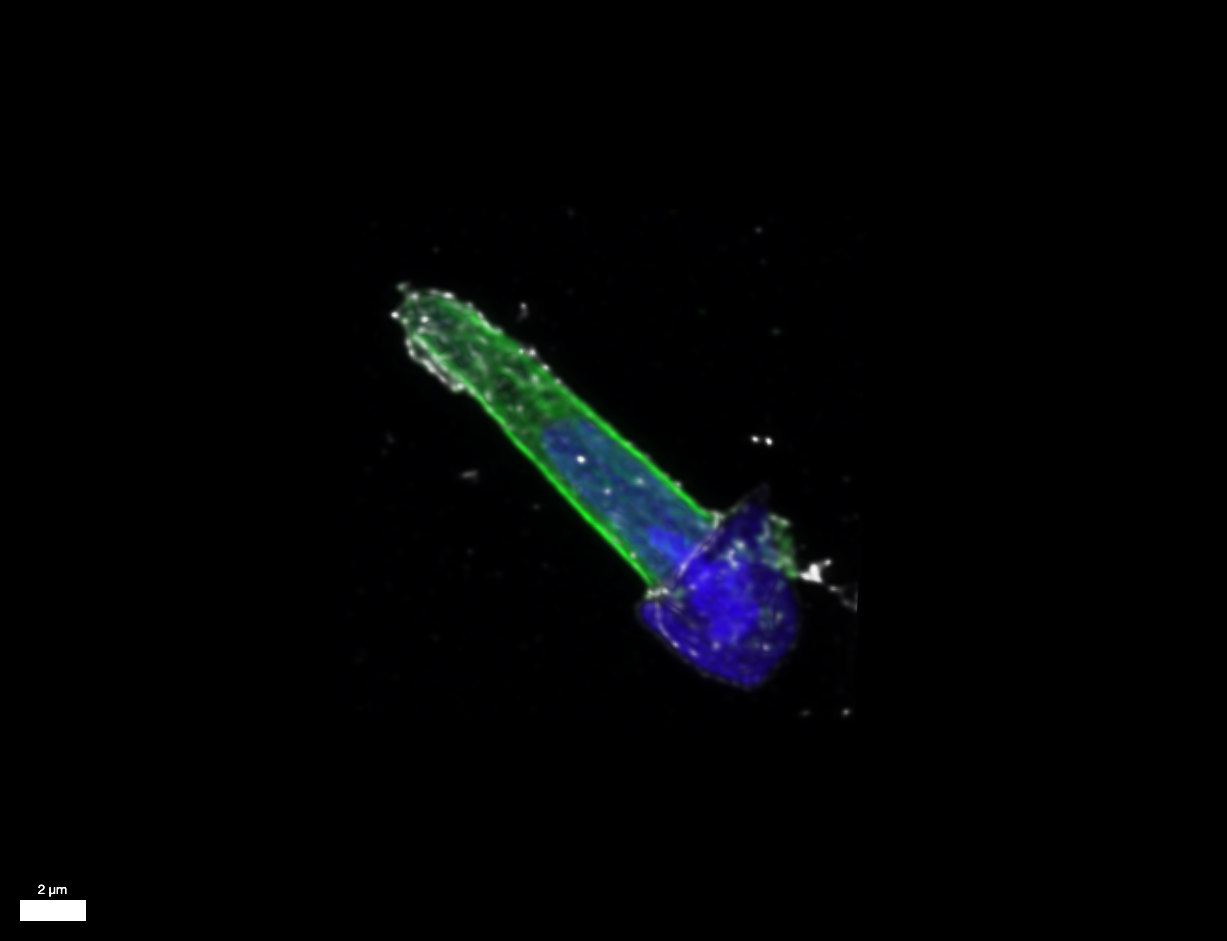

Supplement: Supplementary file 5 — Source data Fig. 5 [file 44319_2024_159_MOESM5_ESM.zip › EMBOR-2023-58207V1_SourceDataForFig5/5E/Tube1GCKO:GCKO/EMBOR-2023-58207V1_SourceDataForFig5EMid_DAPI:Merged.tif]

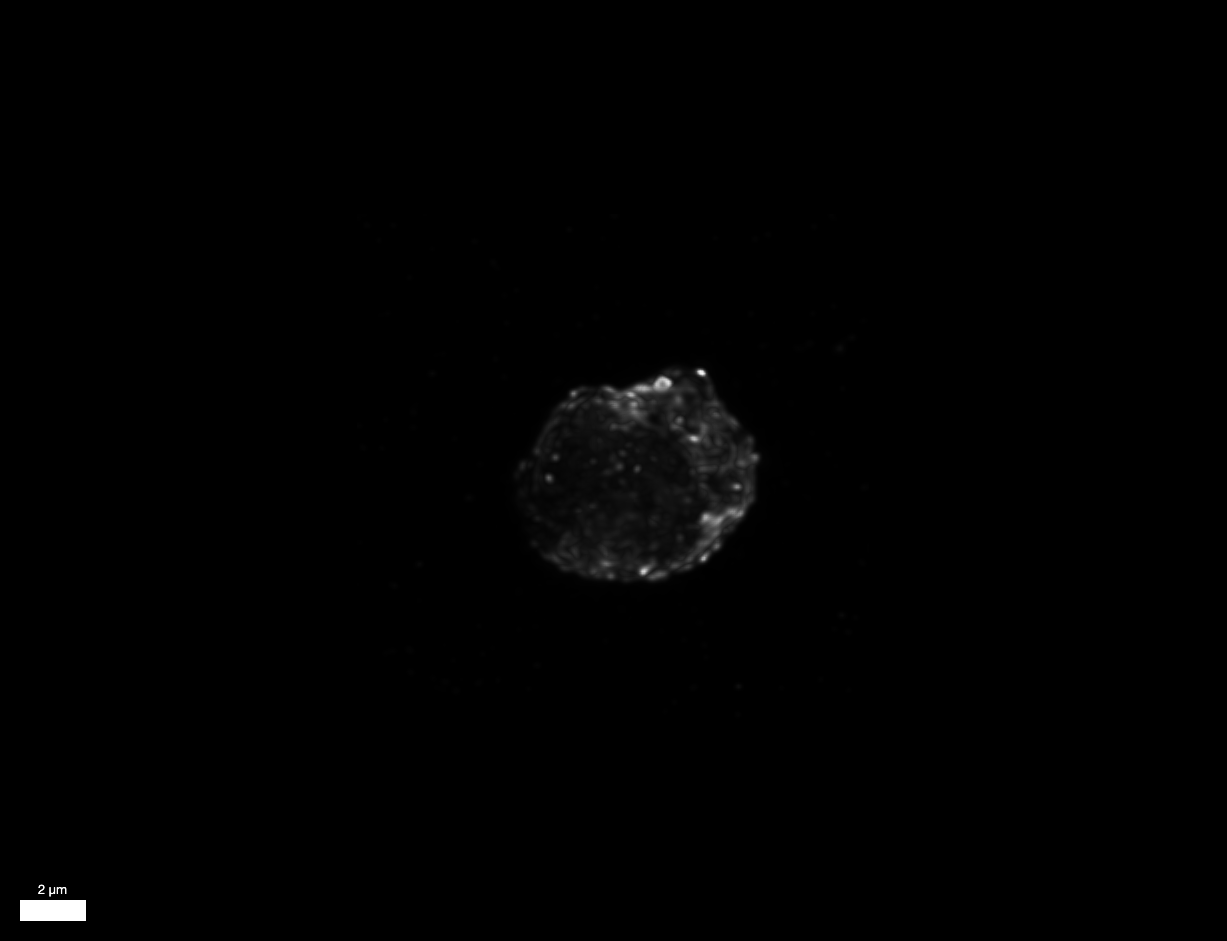

Supplement: Supplementary file 5 — Source data Fig. 5 [file 44319_2024_159_MOESM5_ESM.zip › EMBOR-2023-58207V1_SourceDataForFig5/5E/Tube1GCKO:GCKO/EMBOR-2023-58207V1_SourceDataForFig5EEarly_KATNAL2.png]

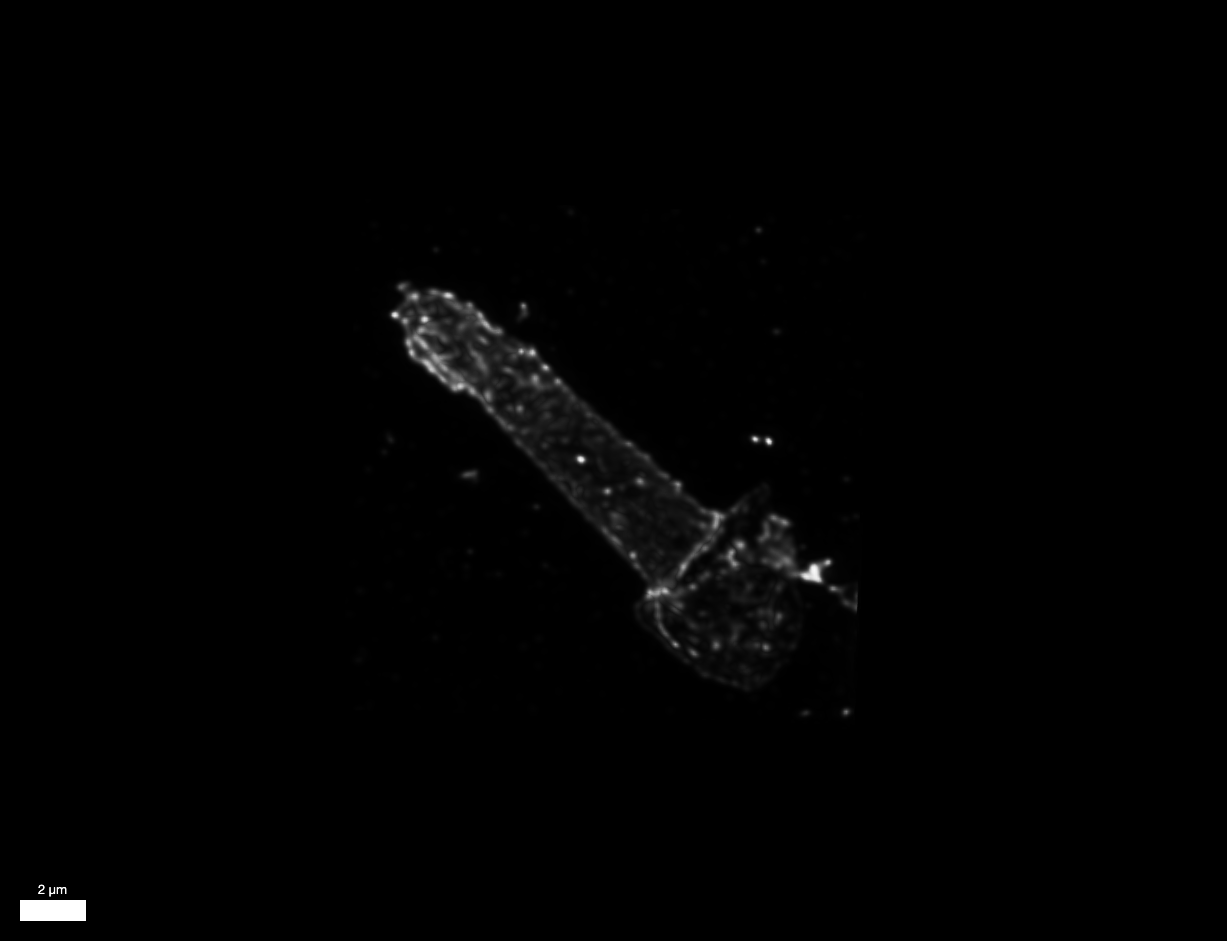

Supplement: Supplementary file 5 — Source data Fig. 5 [file 44319_2024_159_MOESM5_ESM.zip › EMBOR-2023-58207V1_SourceDataForFig5/5E/Tube1GCKO:GCKO/EMBOR-2023-58207V1_SourceDataForFig5EMid_KATNAL2.png]

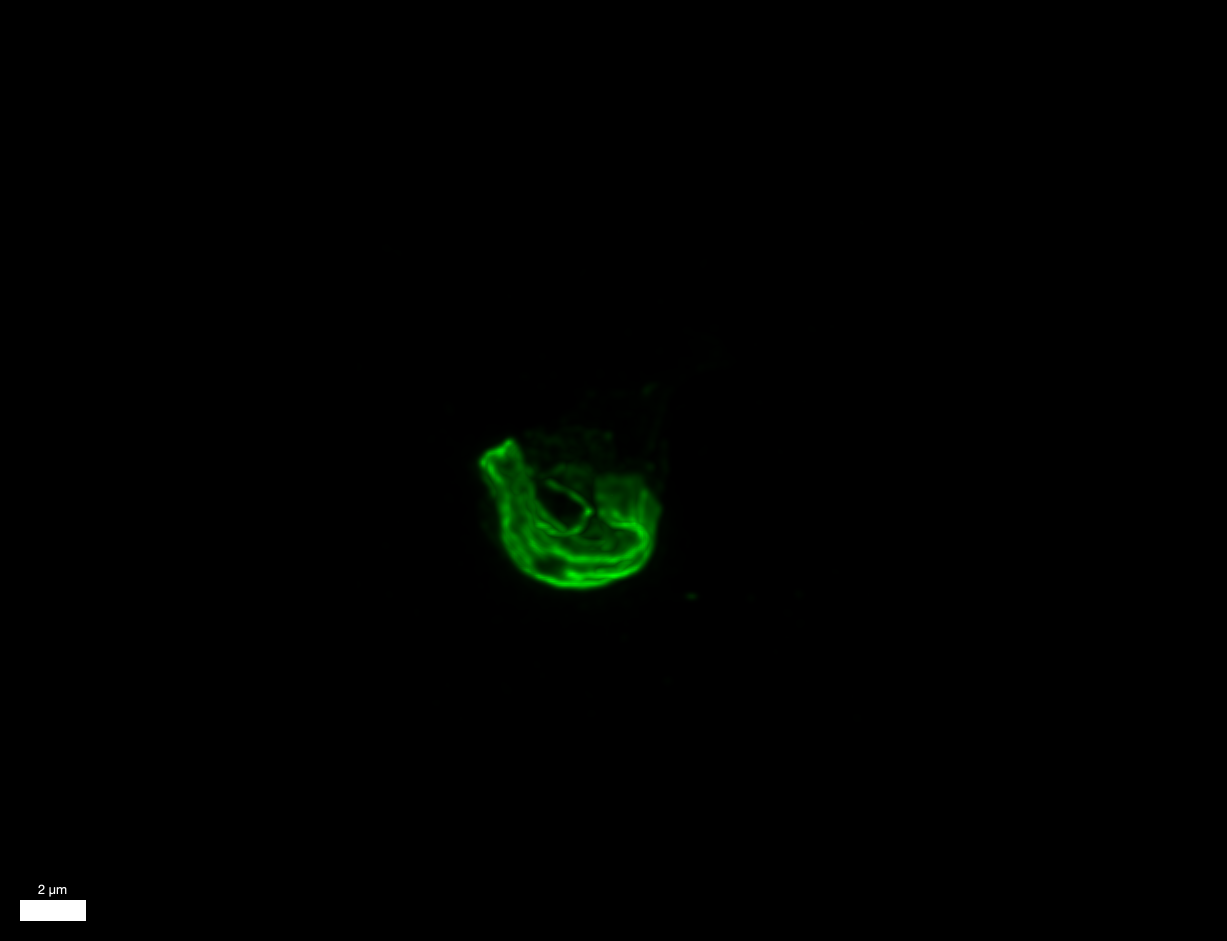

Supplement: Supplementary file 5 — Source data Fig. 5 [file 44319_2024_159_MOESM5_ESM.zip › EMBOR-2023-58207V1_SourceDataForFig5/5E/Tube1GCKO:GCKO/EMBOR-2023-58207V1_SourceDataForFig5ELate_alpha tubulin.png]

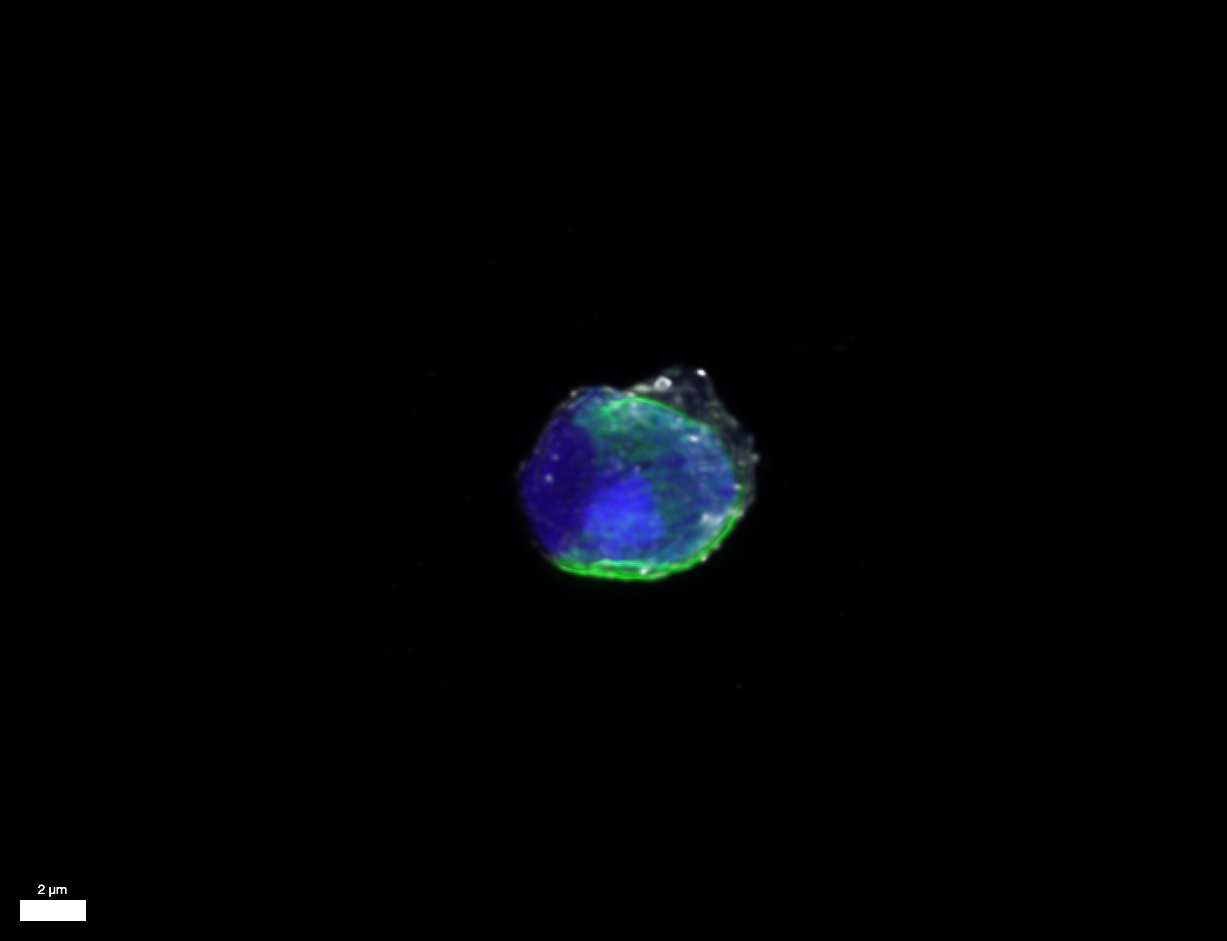

Supplement: Supplementary file 5 — Source data Fig. 5 [file 44319_2024_159_MOESM5_ESM.zip › EMBOR-2023-58207V1_SourceDataForFig5/5E/Tube1GCKO:GCKO/EMBOR-2023-58207V1_SourceDataForFig5EEarly_DAPI:Merged.tif]

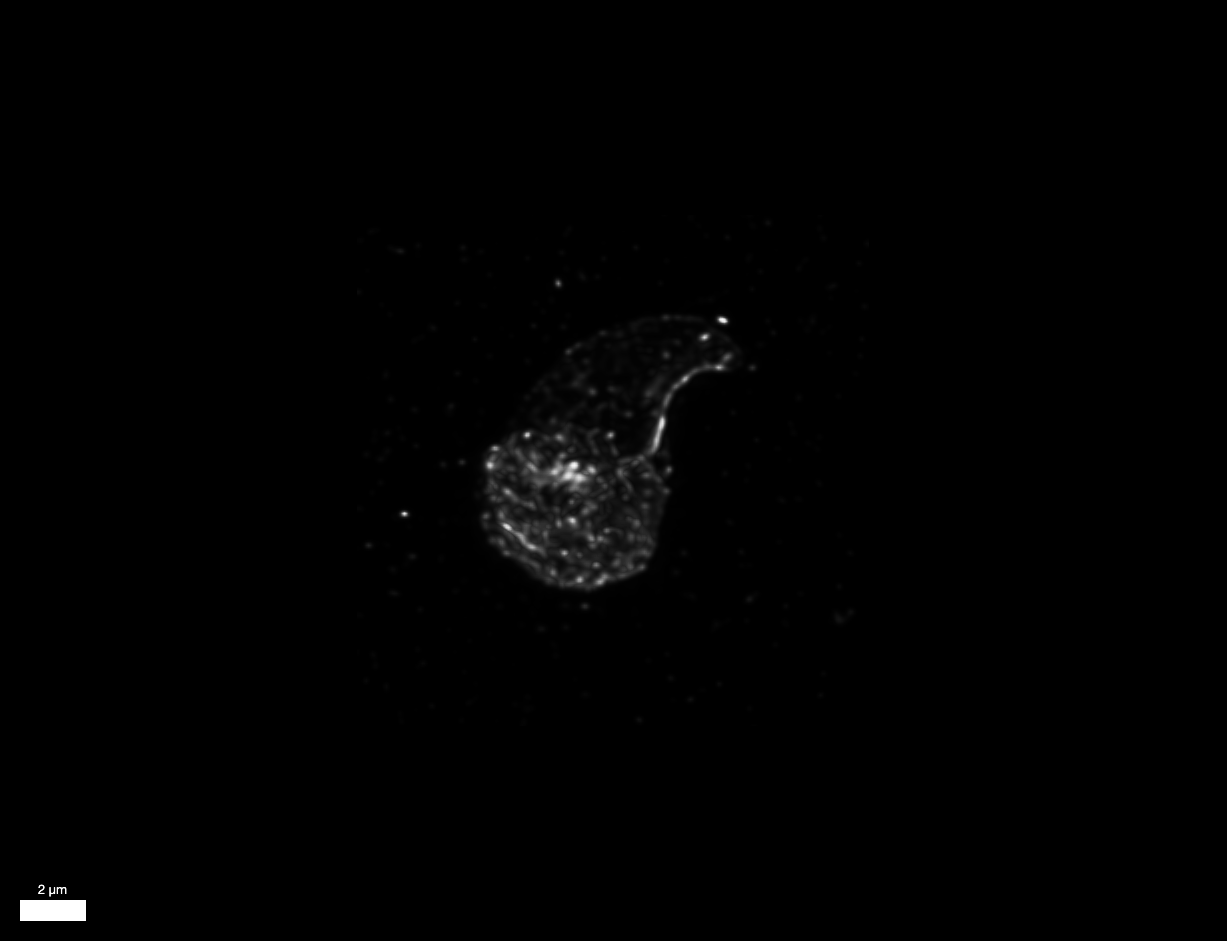

Supplement: Supplementary file 5 — Source data Fig. 5 [file 44319_2024_159_MOESM5_ESM.zip › EMBOR-2023-58207V1_SourceDataForFig5/5E/Tube1GCKO:GCKO/EMBOR-2023-58207V1_SourceDataForFig5ELate_KATNAL2.png]

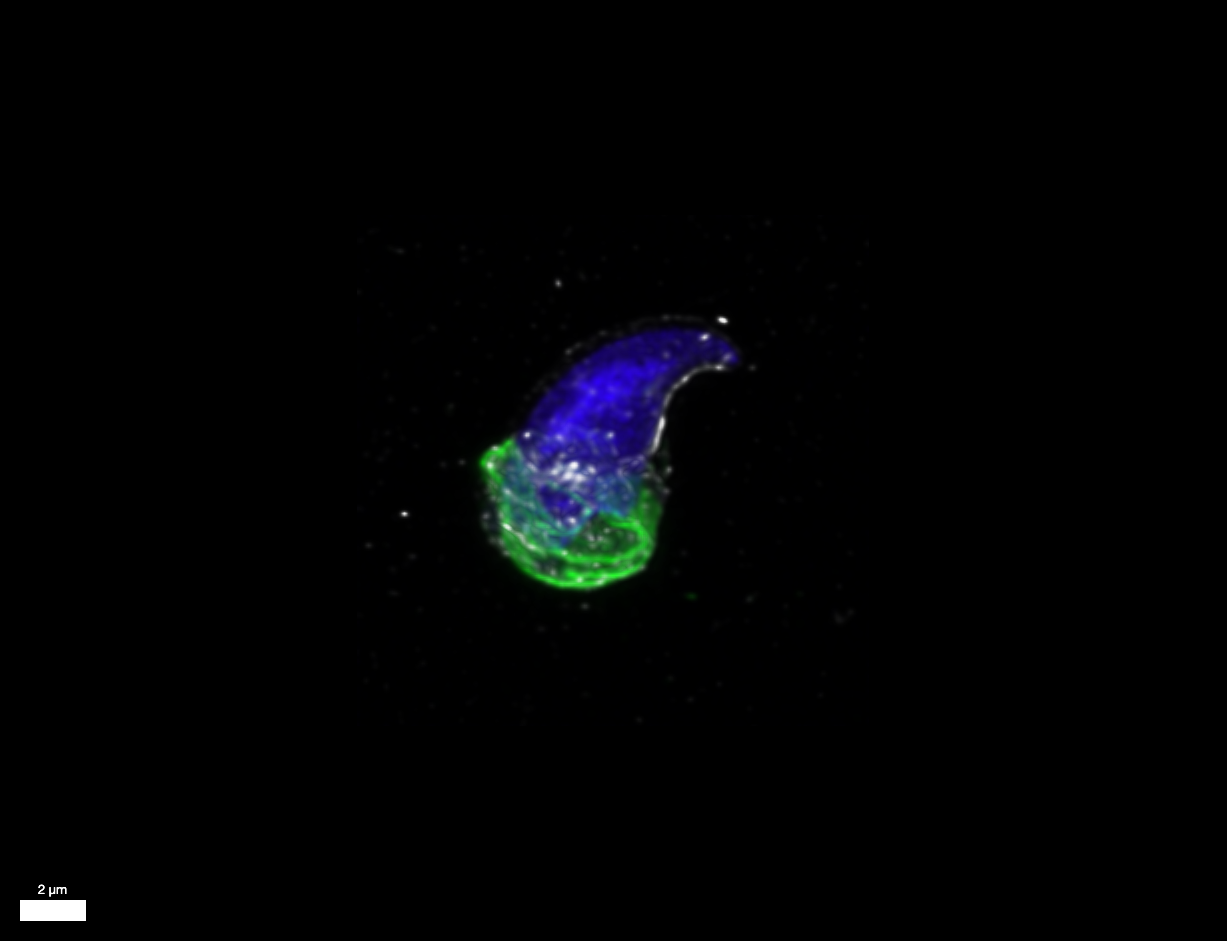

Supplement: Supplementary file 5 — Source data Fig. 5 [file 44319_2024_159_MOESM5_ESM.zip › EMBOR-2023-58207V1_SourceDataForFig5/5E/Tube1GCKO:GCKO/EMBOR-2023-58207V1_SourceDataForFig5ELate_DAPI:Merged.tif]

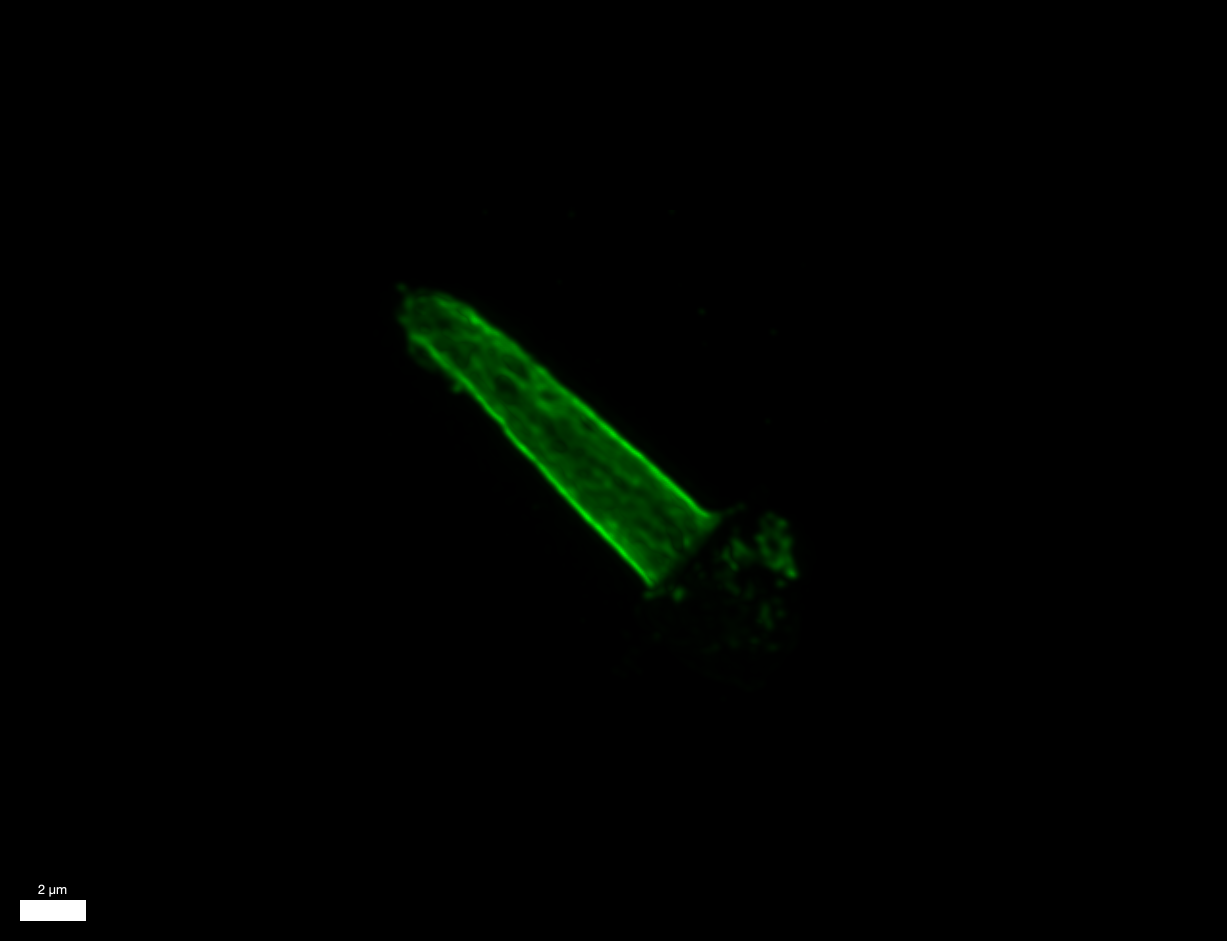

Supplement: Supplementary file 5 — Source data Fig. 5 [file 44319_2024_159_MOESM5_ESM.zip › EMBOR-2023-58207V1_SourceDataForFig5/5E/Tube1GCKO:GCKO/EMBOR-2023-58207V1_SourceDataForFig5EMid_alpha tubulin.png]

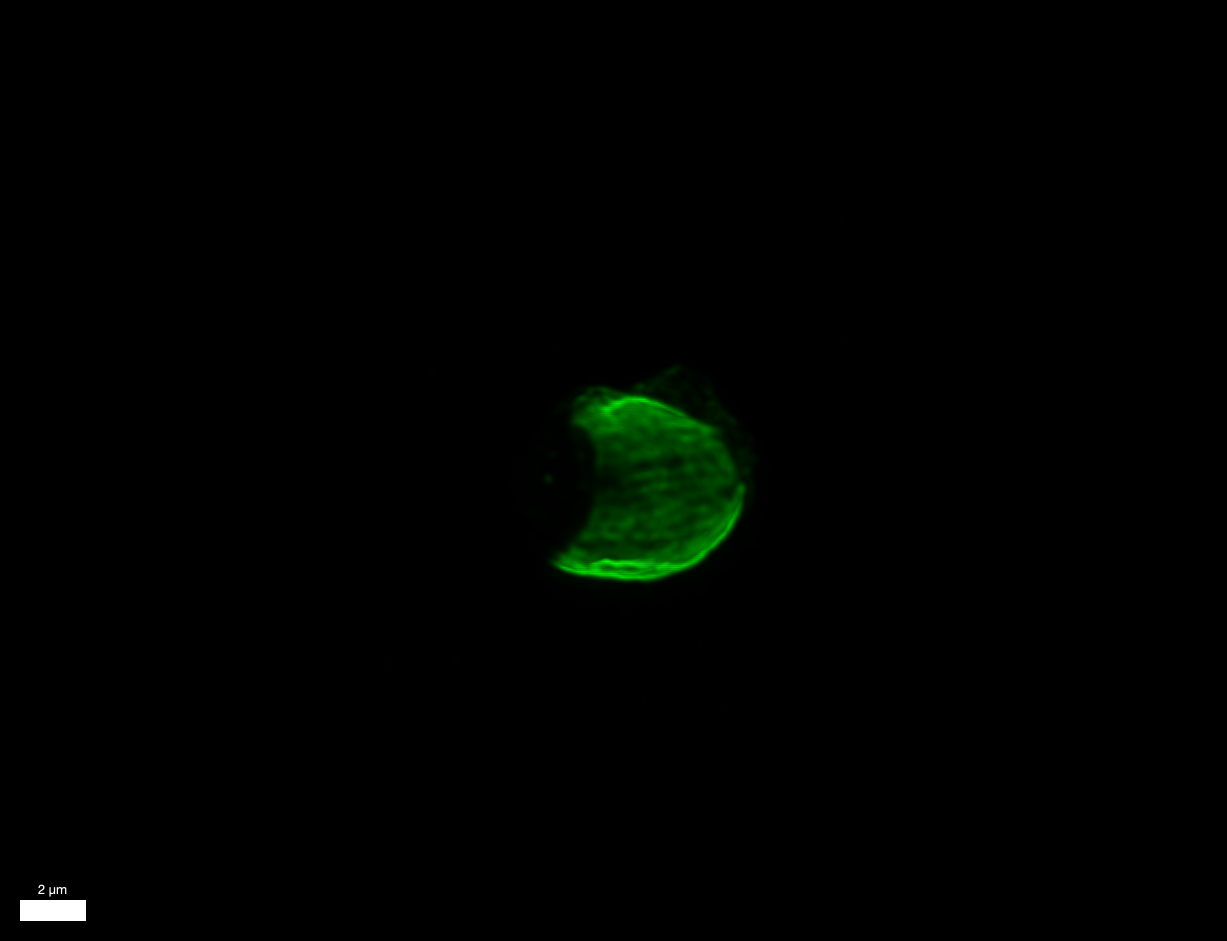

Supplement: Supplementary file 5 — Source data Fig. 5 [file 44319_2024_159_MOESM5_ESM.zip › EMBOR-2023-58207V1_SourceDataForFig5/5E/Tube1GCKO:GCKO/EMBOR-2023-58207V1_SourceDataForFig5EEarly_alpha tubulin.png]

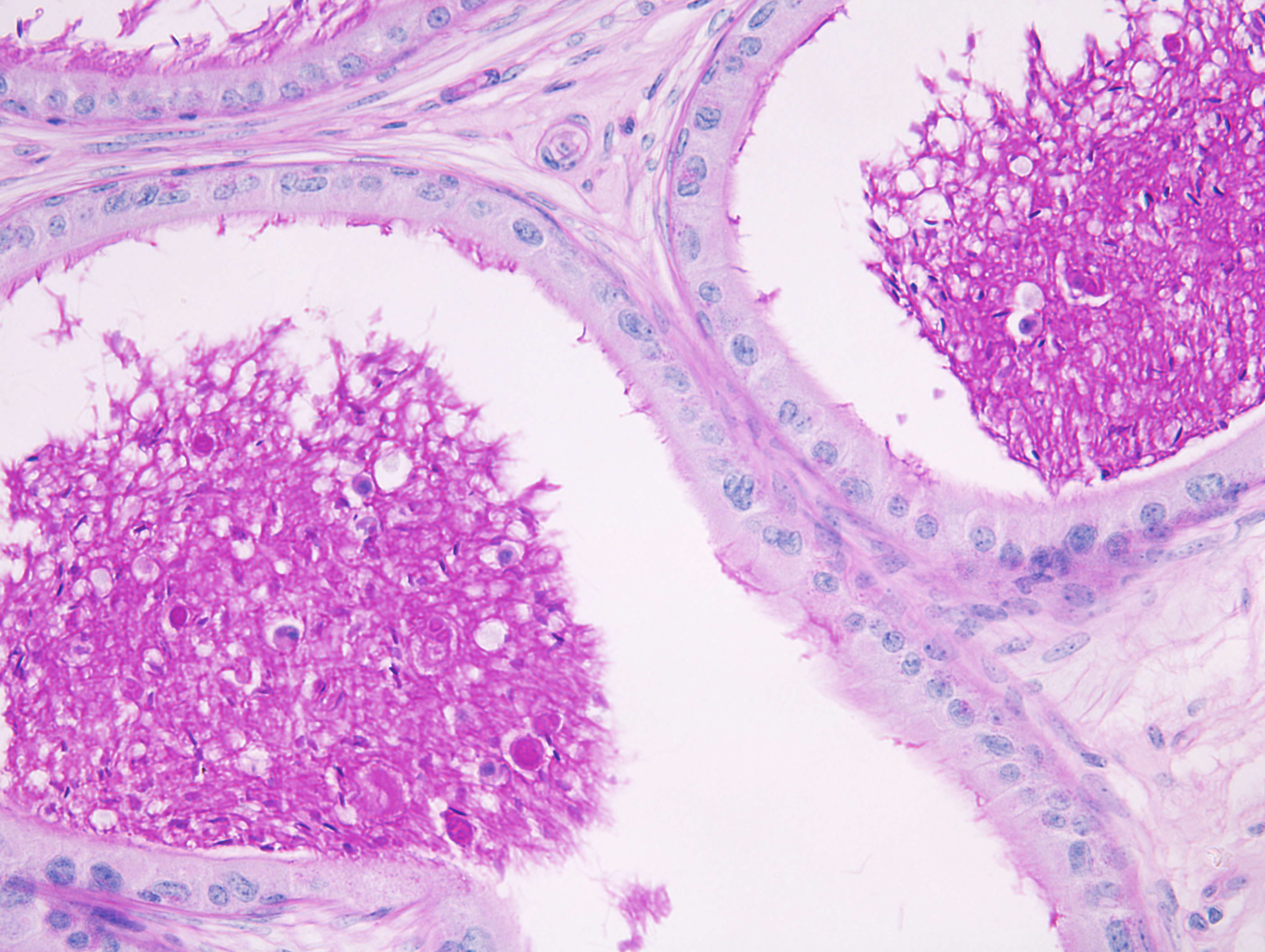

Supplement: Supplementary file 7 — Figure Source Data EV2 [file 44319_2024_159_MOESM7_ESM.zip › EMBOR-2023-58207V1_SourceDataForExpandedView_Figure EV2/EV2E/EMBOR-2023-58207V1_SourceDataForFigEV2E_GCKO.tif]

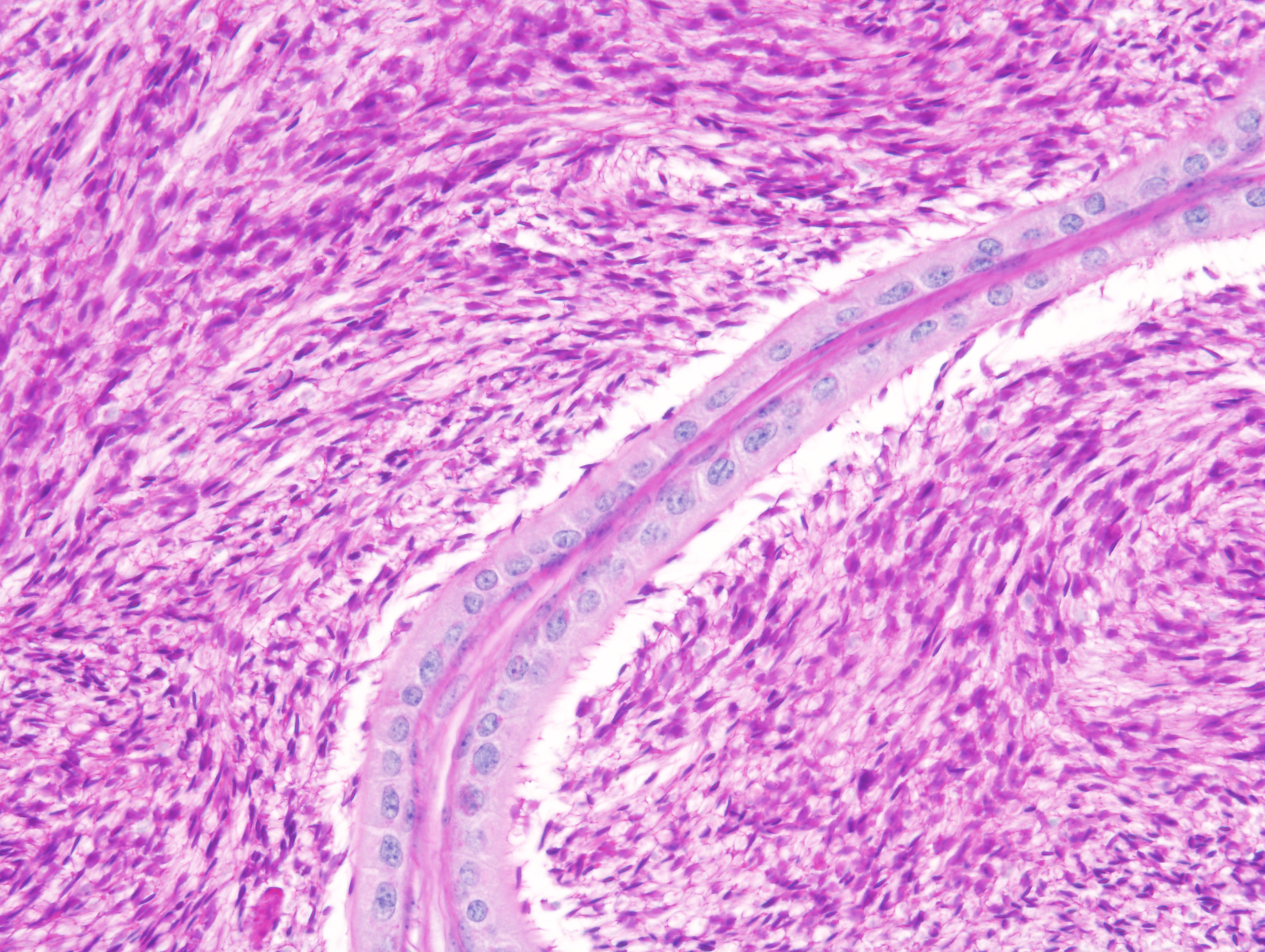

Supplement: Supplementary file 7 — Figure Source Data EV2 [file 44319_2024_159_MOESM7_ESM.zip › EMBOR-2023-58207V1_SourceDataForExpandedView_Figure EV2/EV2E/EMBOR-2023-58207V1_SourceDataForFigEV2E_Flox.tif]

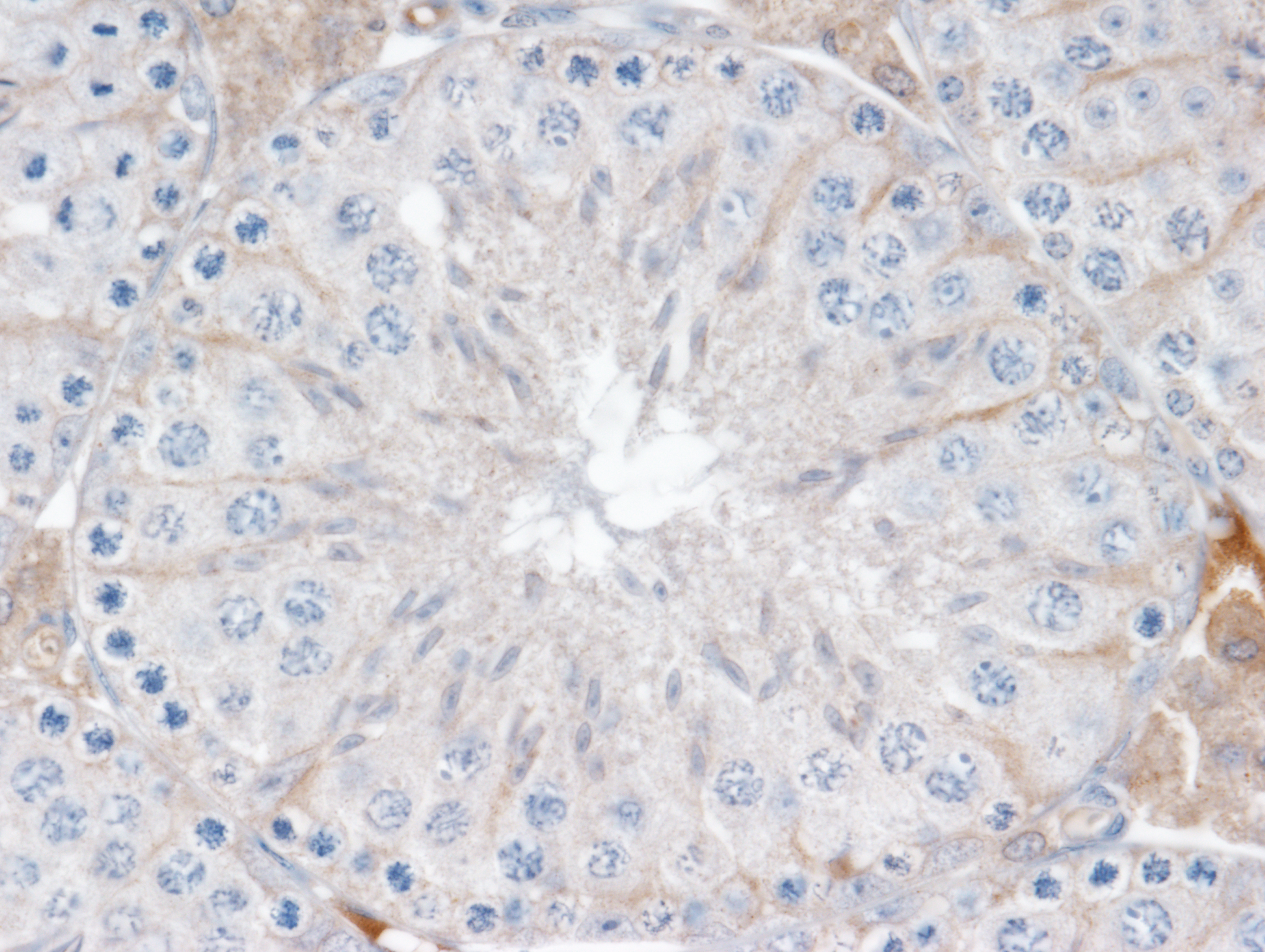

Supplement: Supplementary file 7 — Figure Source Data EV2 [file 44319_2024_159_MOESM7_ESM.zip › EMBOR-2023-58207V1_SourceDataForExpandedView_Figure EV2/EV2D/EMBOR-2023-58207V1_SourceDataForFigEV2D_Flox-X.tif]

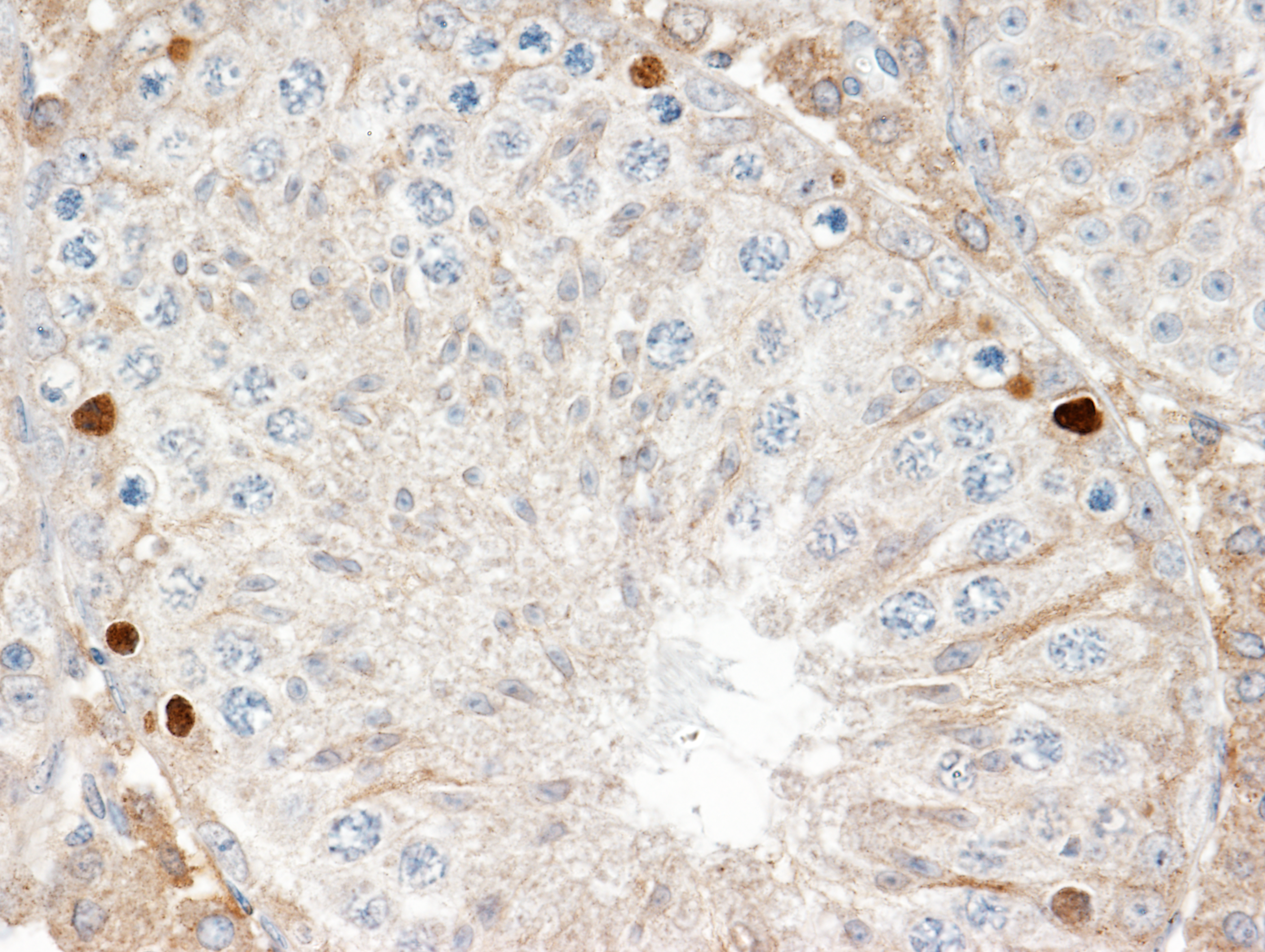

Supplement: Supplementary file 7 — Figure Source Data EV2 [file 44319_2024_159_MOESM7_ESM.zip › EMBOR-2023-58207V1_SourceDataForExpandedView_Figure EV2/EV2D/EMBOR-2023-58207V1_SourceDataForFigEV2D_GCKO-X.tif]

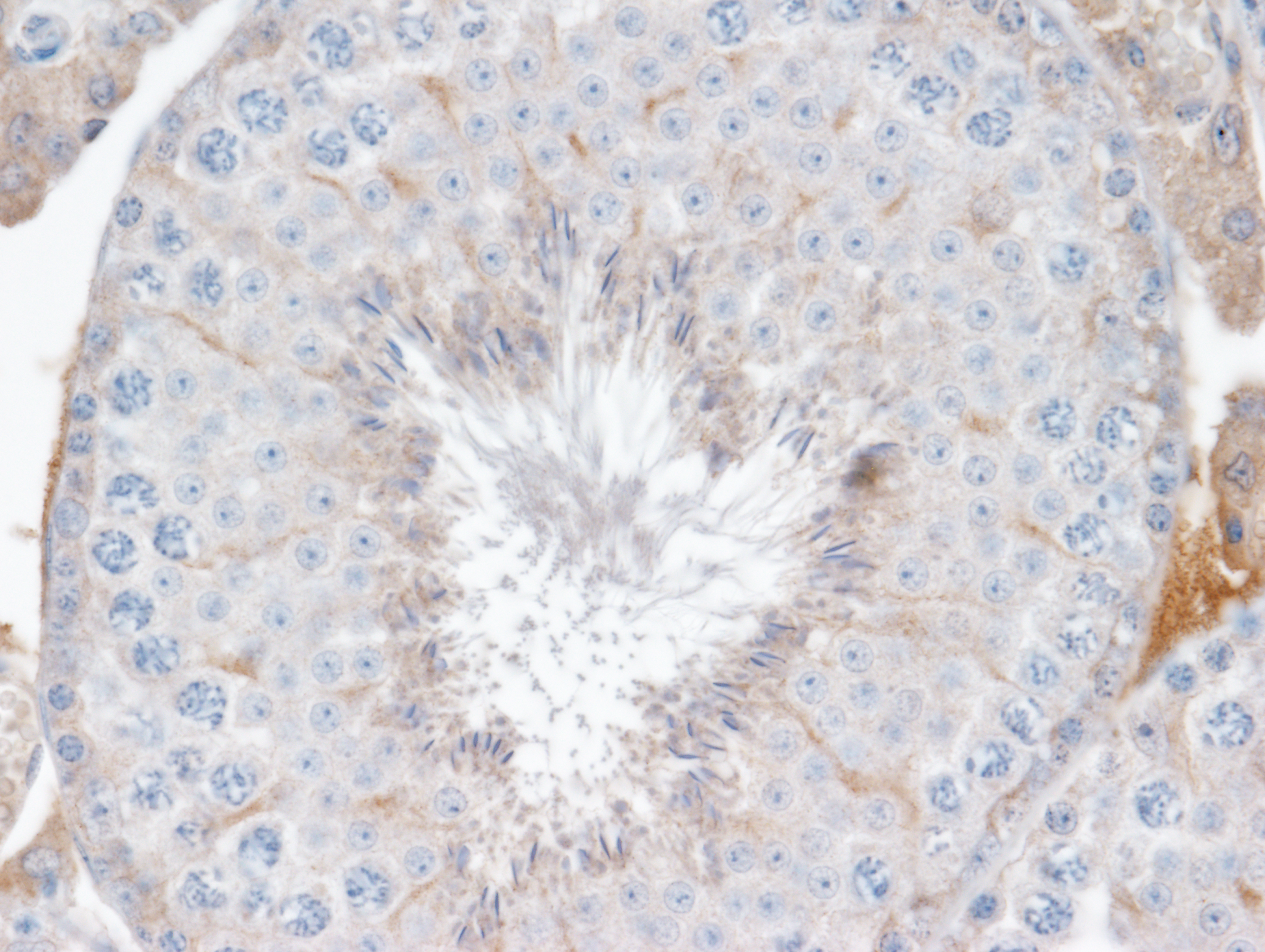

Supplement: Supplementary file 7 — Figure Source Data EV2 [file 44319_2024_159_MOESM7_ESM.zip › EMBOR-2023-58207V1_SourceDataForExpandedView_Figure EV2/EV2D/EMBOR-2023-58207V1_SourceDataForFigEV2D_Flox-VII.tif]

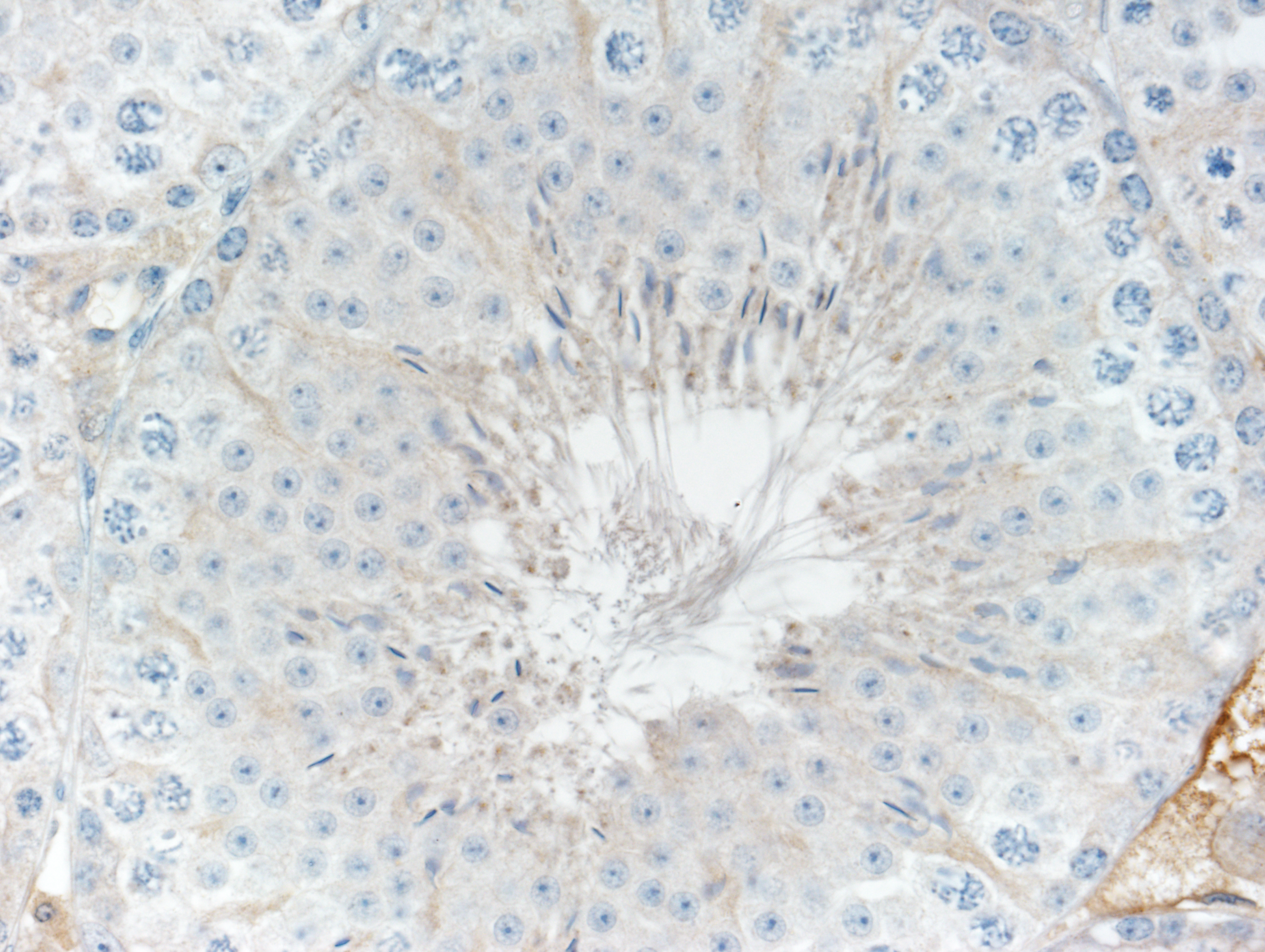

Supplement: Supplementary file 7 — Figure Source Data EV2 [file 44319_2024_159_MOESM7_ESM.zip › EMBOR-2023-58207V1_SourceDataForExpandedView_Figure EV2/EV2D/EMBOR-2023-58207V1_SourceDataForFigEV2D_Flox-VI.tif]

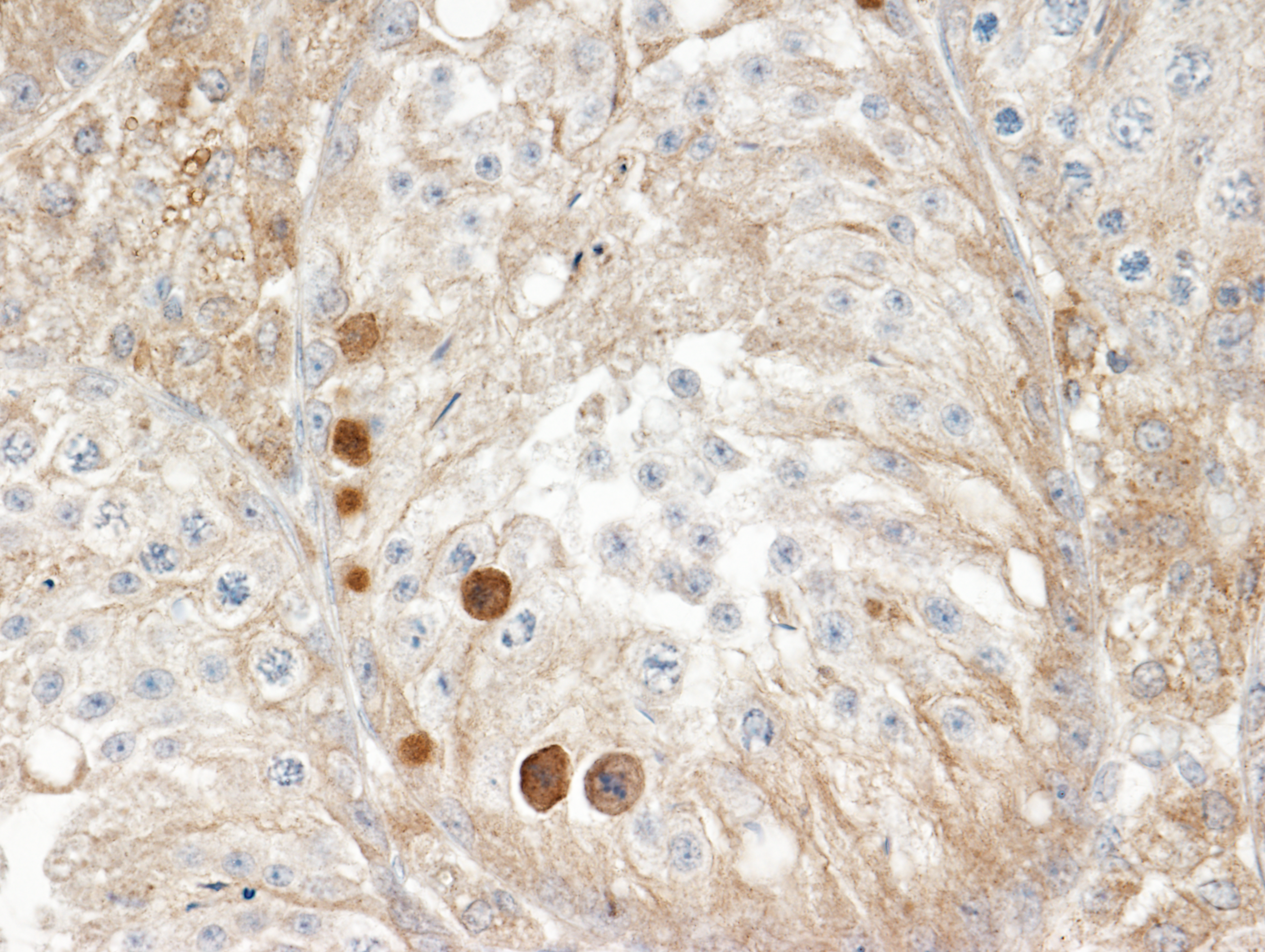

Supplement: Supplementary file 7 — Figure Source Data EV2 [file 44319_2024_159_MOESM7_ESM.zip › EMBOR-2023-58207V1_SourceDataForExpandedView_Figure EV2/EV2D/EMBOR-2023-58207V1_SourceDataForFigEV2D_GCKO-XI.tif]

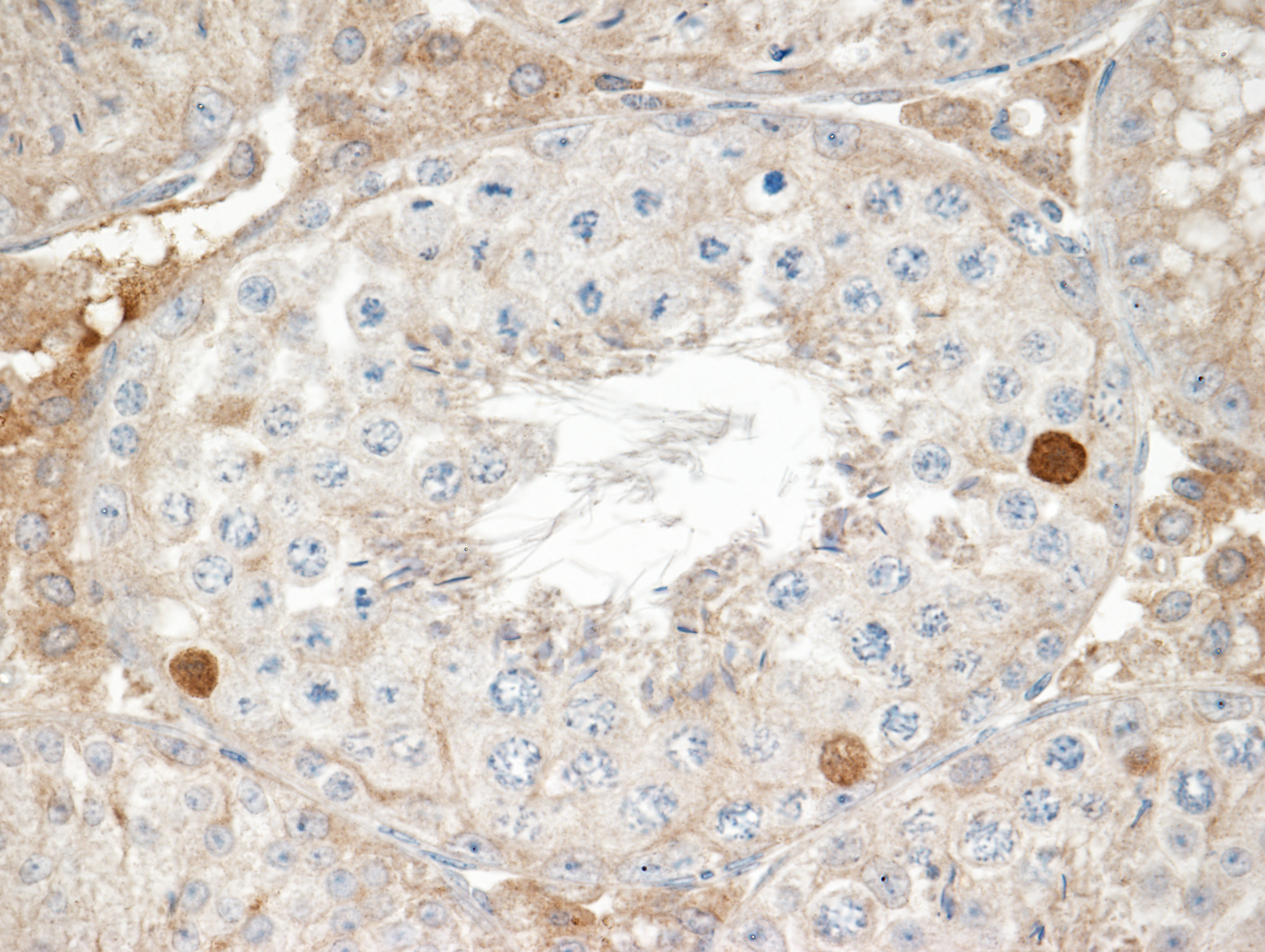

Supplement: Supplementary file 7 — Figure Source Data EV2 [file 44319_2024_159_MOESM7_ESM.zip › EMBOR-2023-58207V1_SourceDataForExpandedView_Figure EV2/EV2D/EMBOR-2023-58207V1_SourceDataForFigEV2D_GCKO-VI.tif]

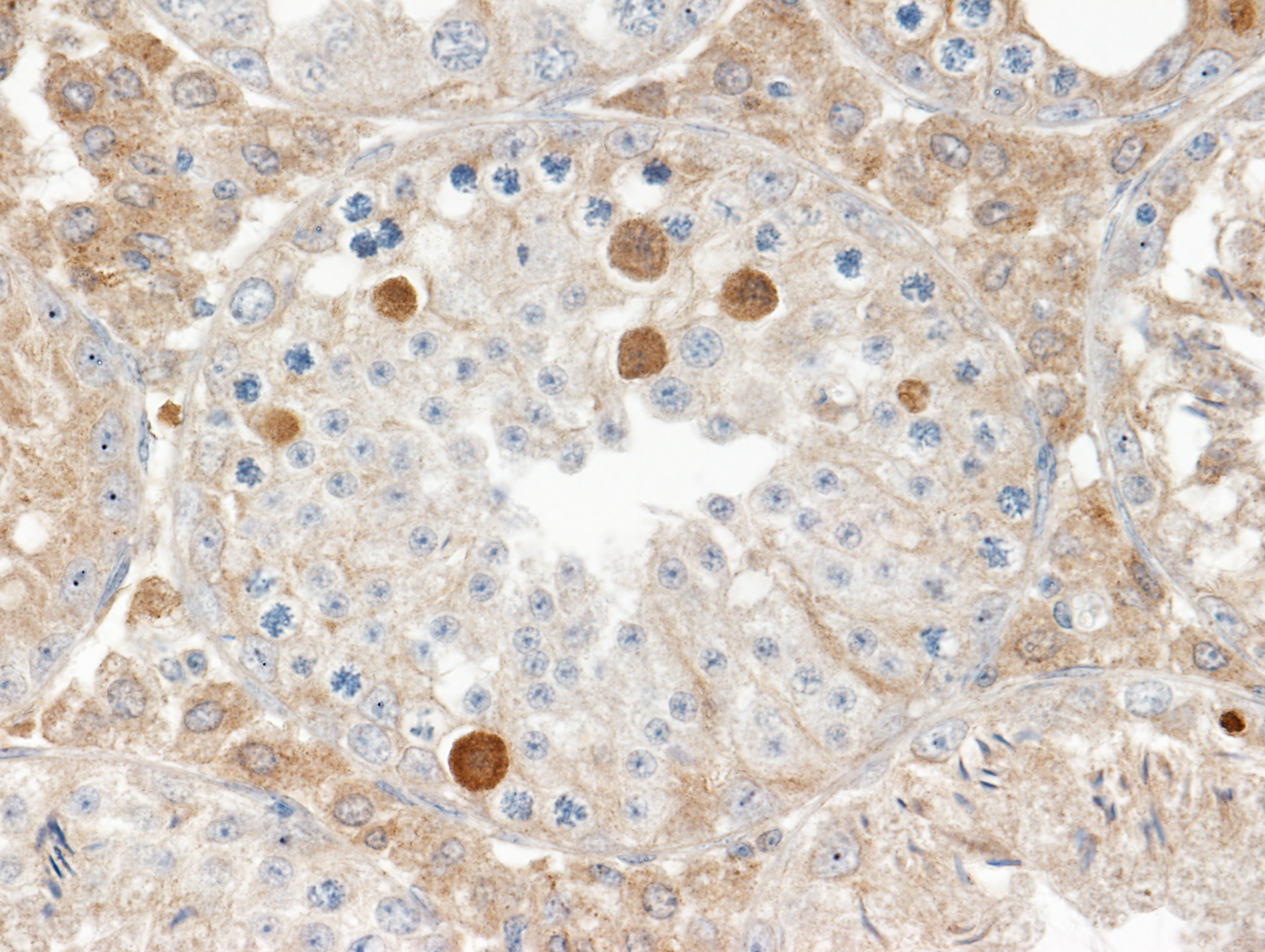

Supplement: Supplementary file 7 — Figure Source Data EV2 [file 44319_2024_159_MOESM7_ESM.zip › EMBOR-2023-58207V1_SourceDataForExpandedView_Figure EV2/EV2D/EMBOR-2023-58207V1_SourceDataForFigEV2D_GCKO-VII.tif]

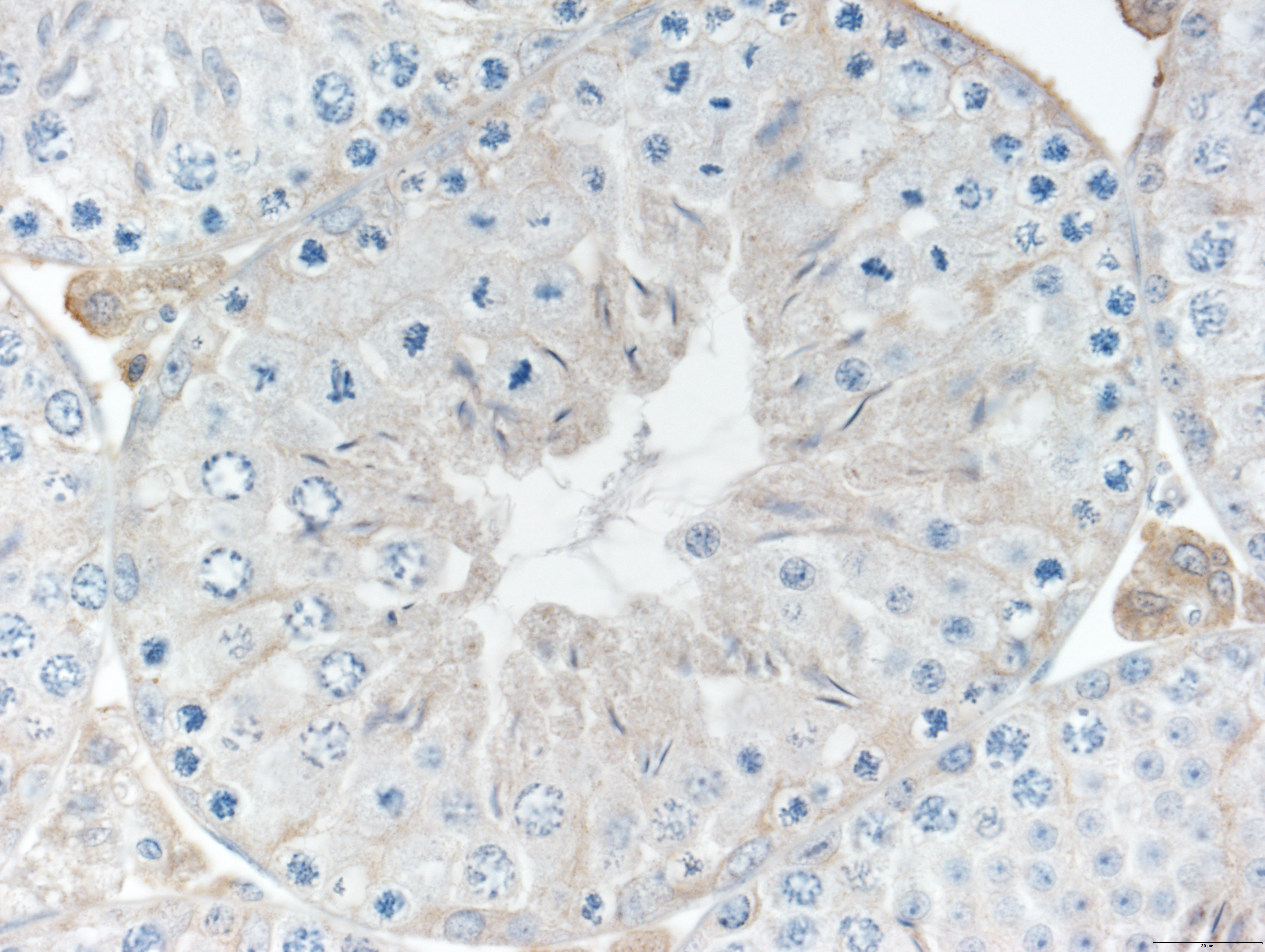

Supplement: Supplementary file 7 — Figure Source Data EV2 [file 44319_2024_159_MOESM7_ESM.zip › EMBOR-2023-58207V1_SourceDataForExpandedView_Figure EV2/EV2D/EMBOR-2023-58207V1_SourceDataForFigEV2D_Flox-XII.tif]

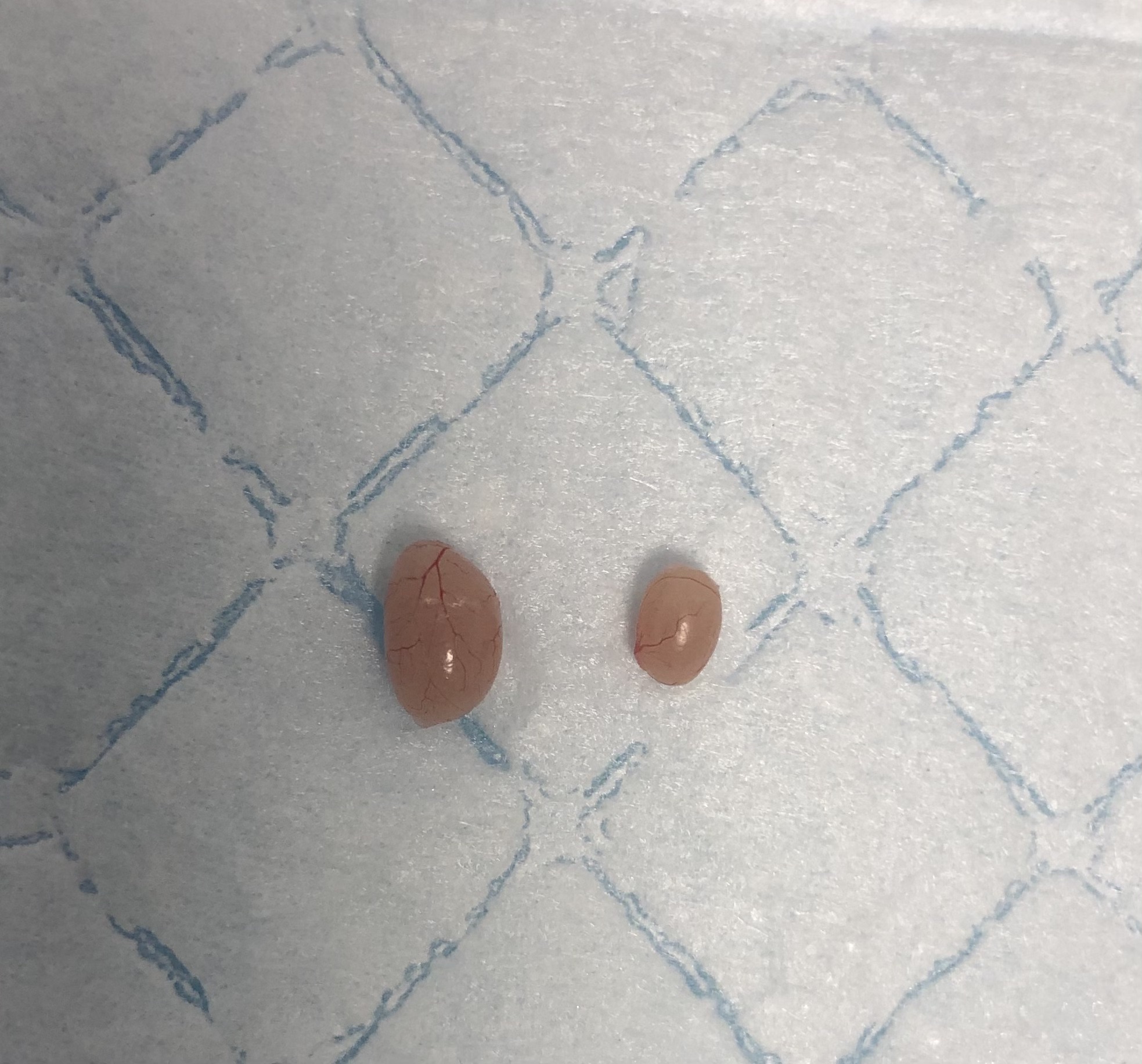

Supplement: Supplementary file 7 — Figure Source Data EV2 [file 44319_2024_159_MOESM7_ESM.zip › EMBOR-2023-58207V1_SourceDataForExpandedView_Figure EV2/EV2A/EMBOR-2023-58207V1_SourceDataForFigEV2A.jpeg]

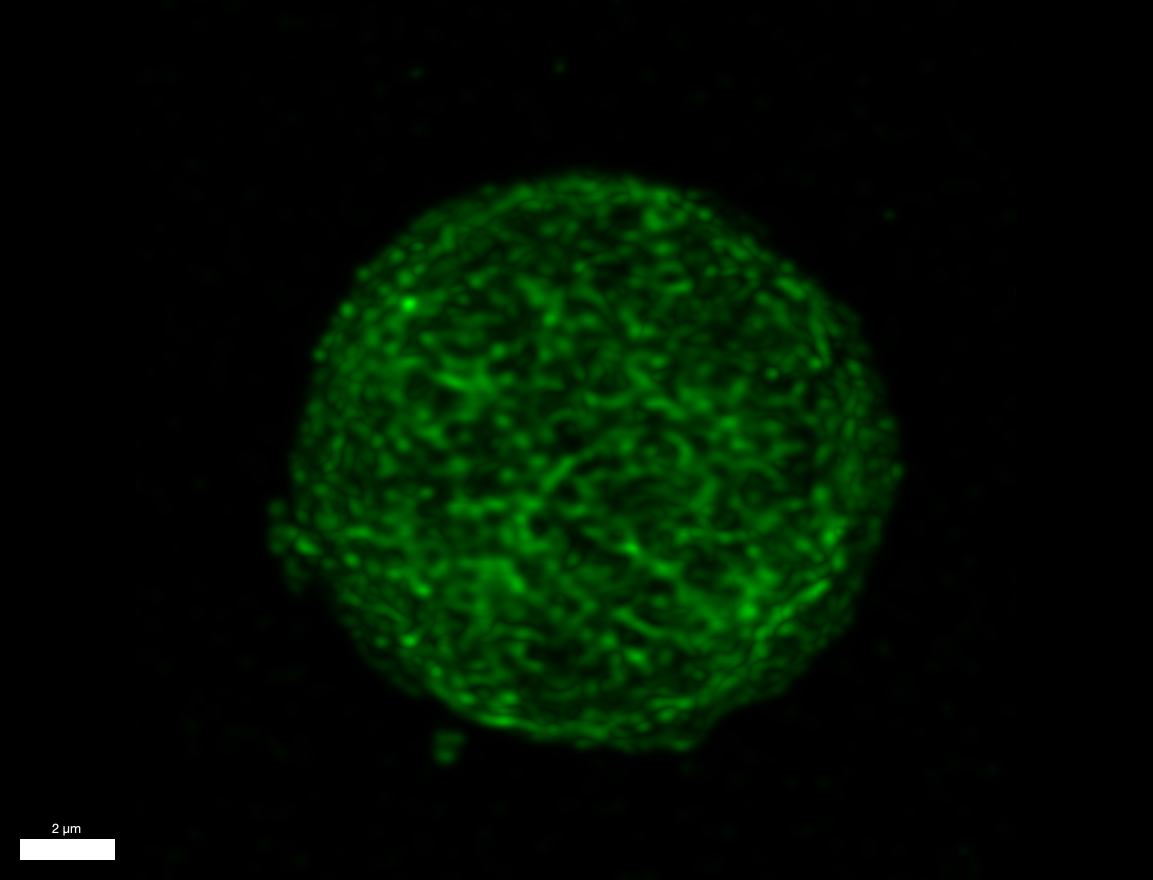

Supplement: Supplementary file 8 — Figure Source Data EV3 [file 44319_2024_159_MOESM8_ESM.zip › EMBOR-2023-58207V1_SourceDataForExpandedView_Figure EV3 /EV3C/EMBOR-2023-58207V1_SourceDataForFigEV3C_Tube1GCKO:GCKO_b-tubulin.tif]

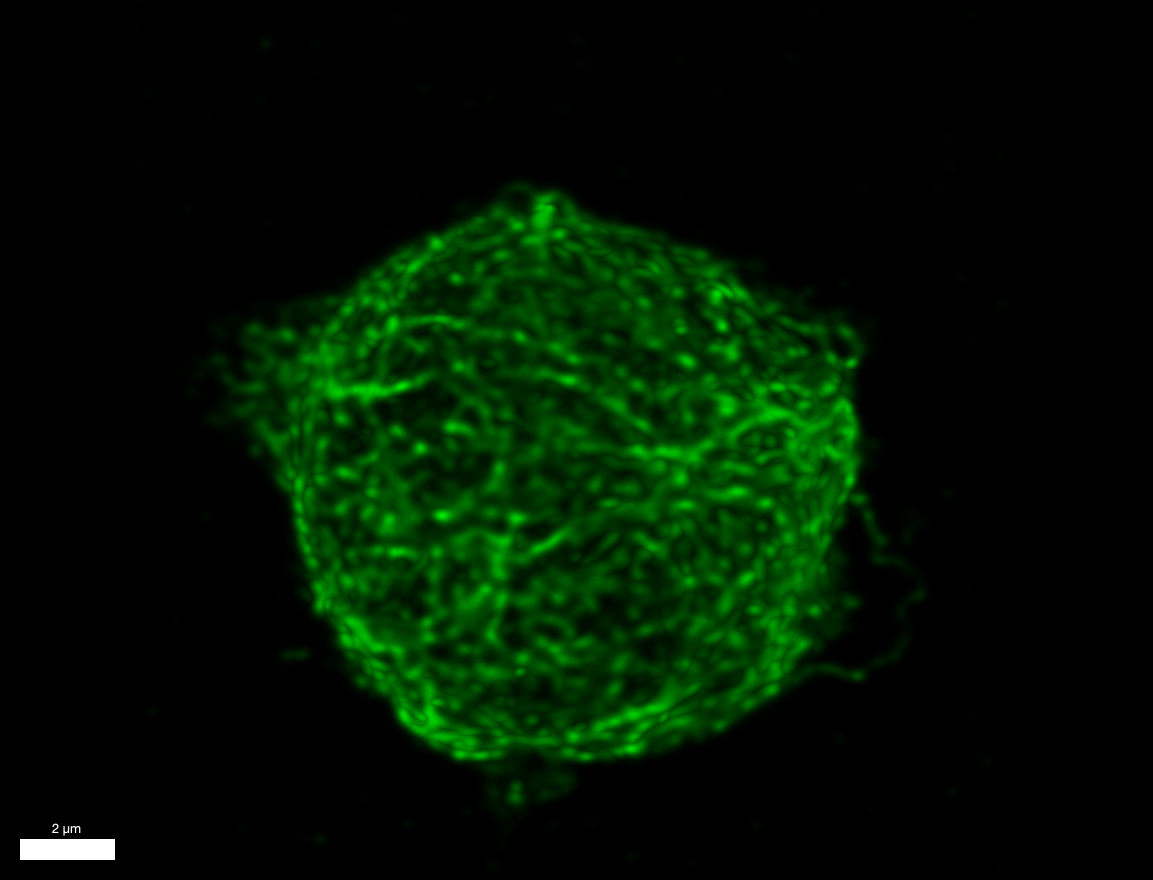

Supplement: Supplementary file 8 — Figure Source Data EV3 [file 44319_2024_159_MOESM8_ESM.zip › EMBOR-2023-58207V1_SourceDataForExpandedView_Figure EV3 /EV3C/EMBOR-2023-58207V1_SourceDataForFigEV3C_Tube1Flox:Flox_b-tubulin.tif]

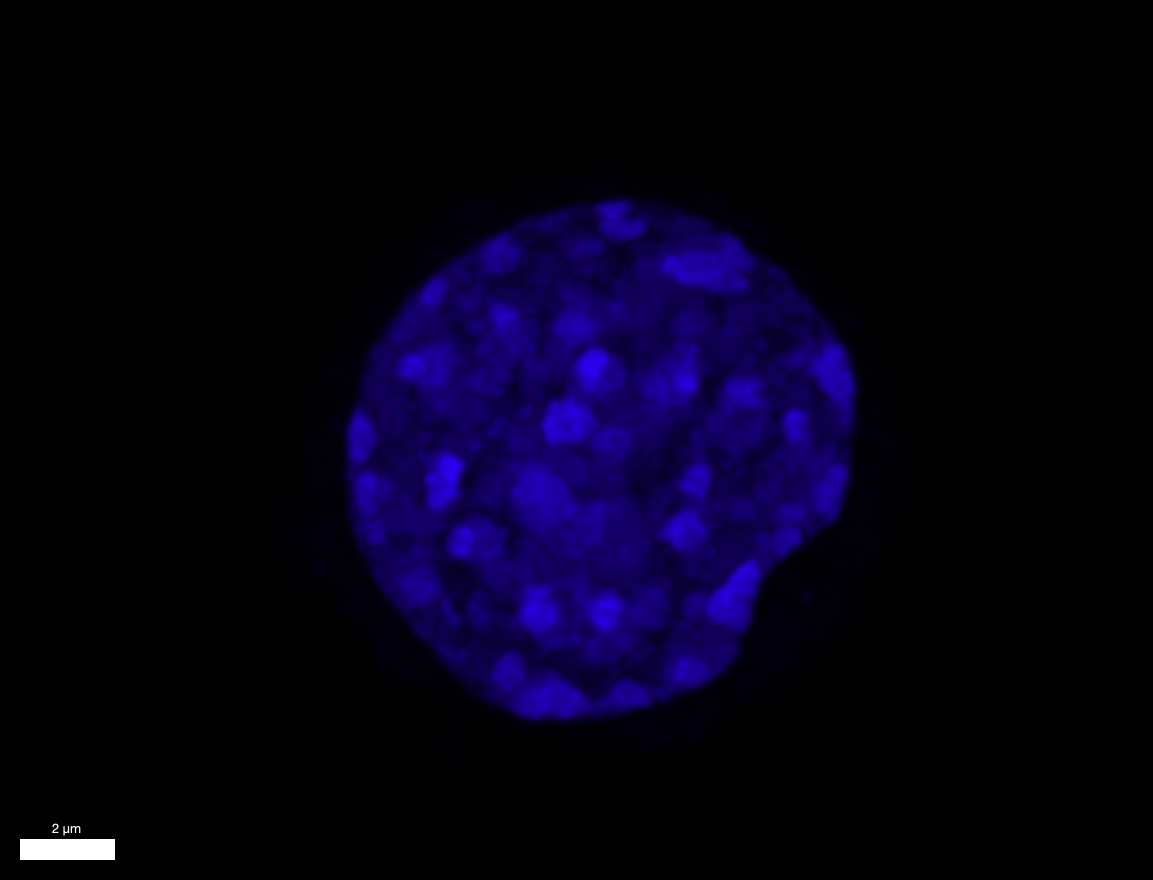

Supplement: Supplementary file 8 — Figure Source Data EV3 [file 44319_2024_159_MOESM8_ESM.zip › EMBOR-2023-58207V1_SourceDataForExpandedView_Figure EV3 /EV3C/EMBOR-2023-58207V1_SourceDataForFigEV3C_Tube1GCKO:GCKO_DAPI.tif]

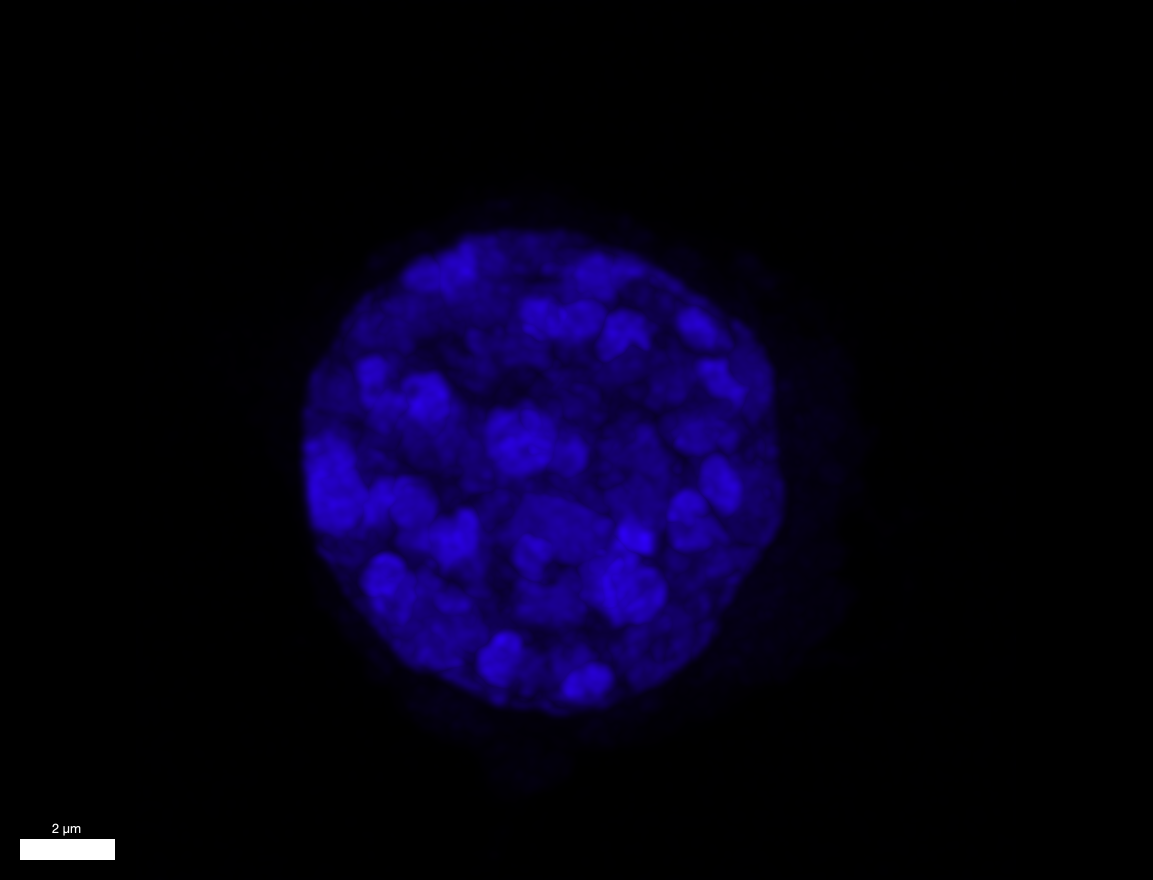

Supplement: Supplementary file 8 — Figure Source Data EV3 [file 44319_2024_159_MOESM8_ESM.zip › EMBOR-2023-58207V1_SourceDataForExpandedView_Figure EV3 /EV3C/EMBOR-2023-58207V1_SourceDataForFigEV3C_Tube1Flox:Flox_DAPI.tif]

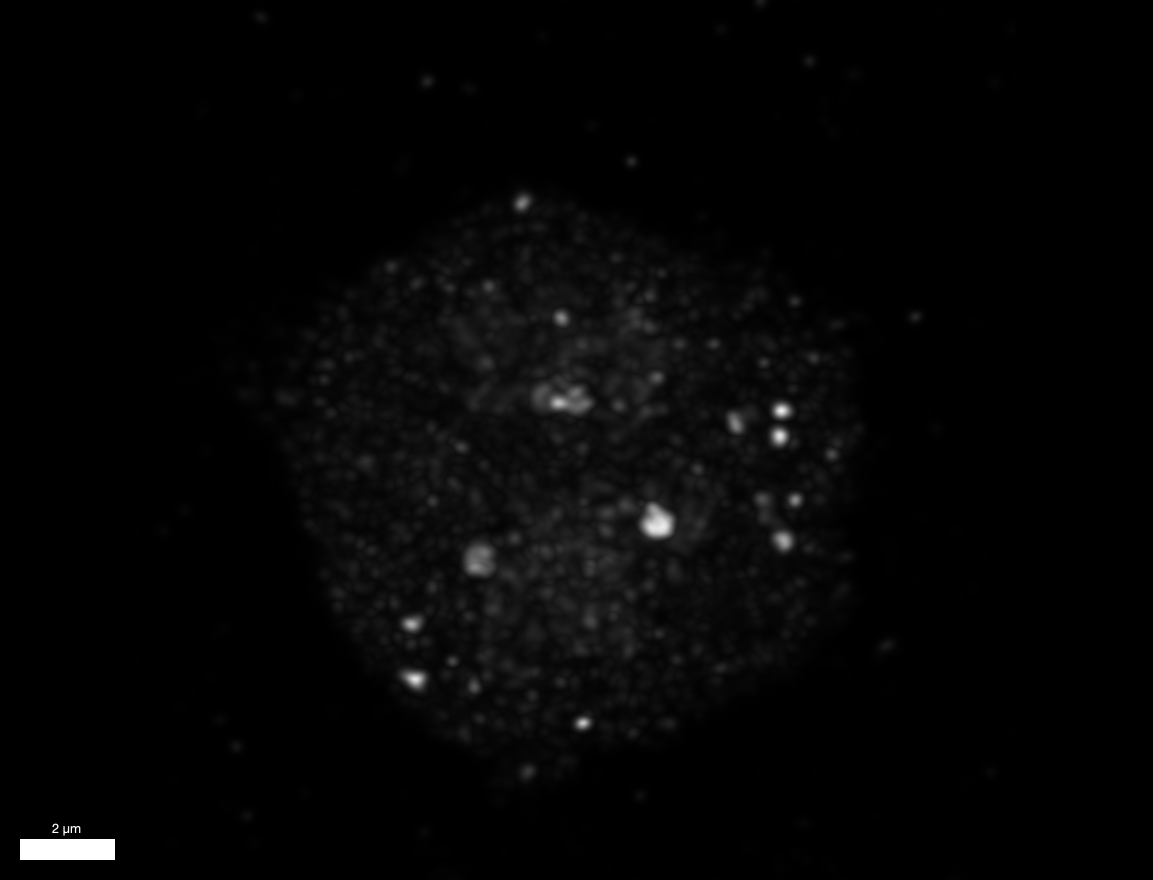

Supplement: Supplementary file 8 — Figure Source Data EV3 [file 44319_2024_159_MOESM8_ESM.zip › EMBOR-2023-58207V1_SourceDataForExpandedView_Figure EV3 /EV3C/EMBOR-2023-58207V1_SourceDataForFigEV3C_Tube1Flox:Flox_SYCP3.tif]

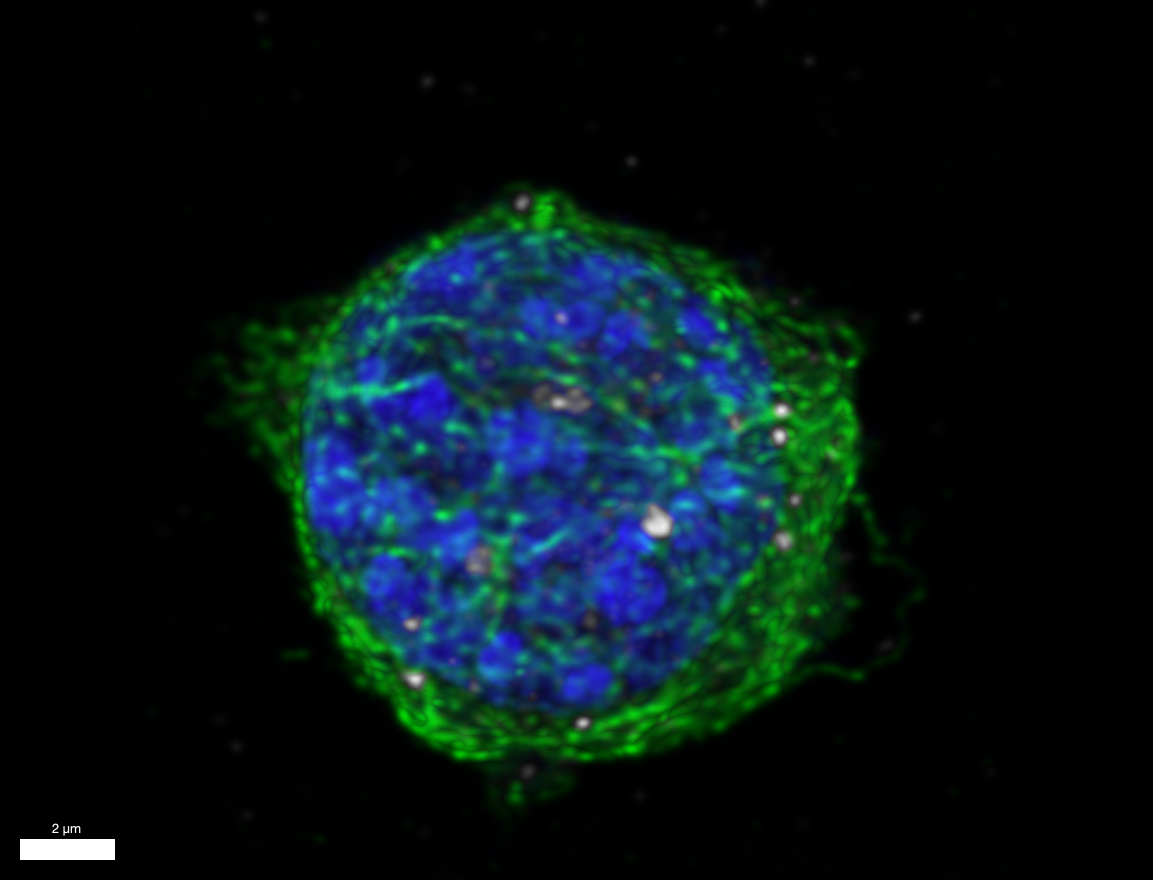

Supplement: Supplementary file 8 — Figure Source Data EV3 [file 44319_2024_159_MOESM8_ESM.zip › EMBOR-2023-58207V1_SourceDataForExpandedView_Figure EV3 /EV3C/EMBOR-2023-58207V1_SourceDataForFigEV3C_Tube1Flox:Flox_merge.tif]

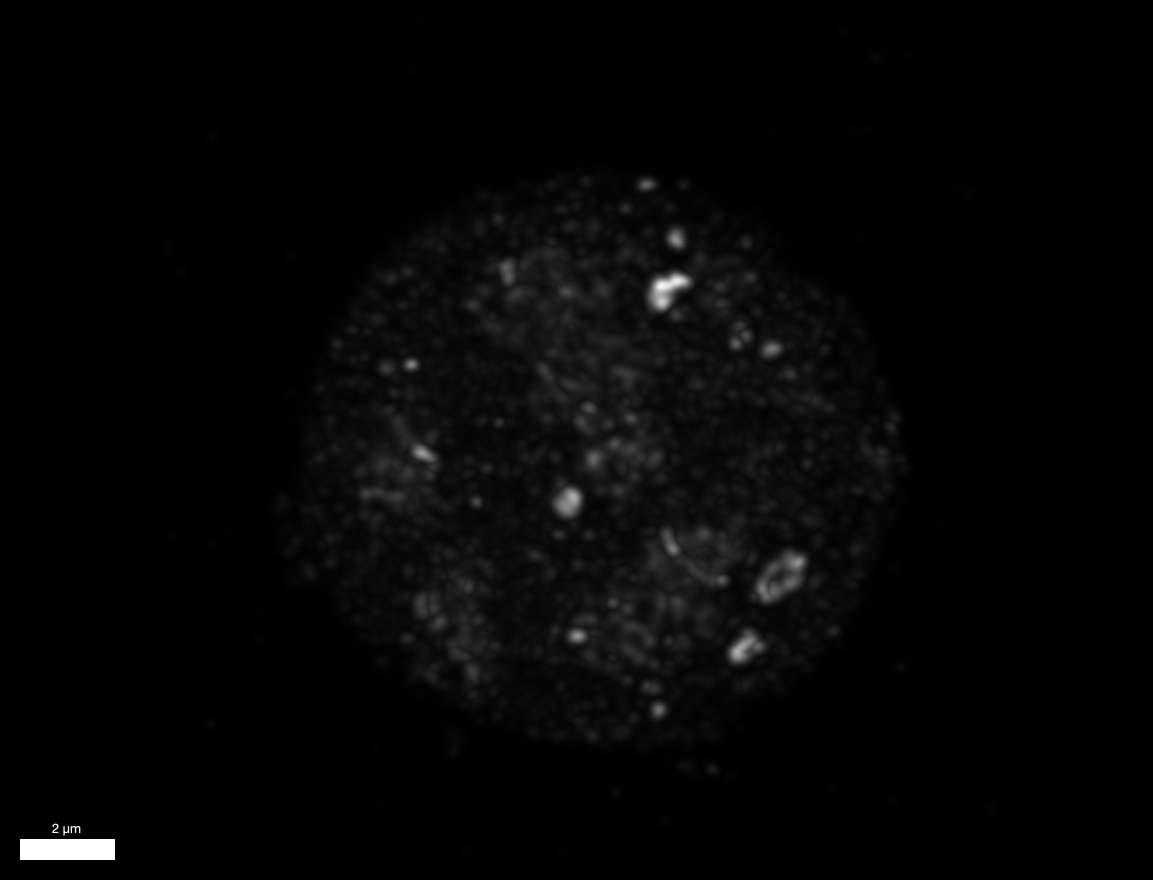

Supplement: Supplementary file 8 — Figure Source Data EV3 [file 44319_2024_159_MOESM8_ESM.zip › EMBOR-2023-58207V1_SourceDataForExpandedView_Figure EV3 /EV3C/EMBOR-2023-58207V1_SourceDataForFigEV3C_Tube1GCKO:GCKO_SYCP3.tif]

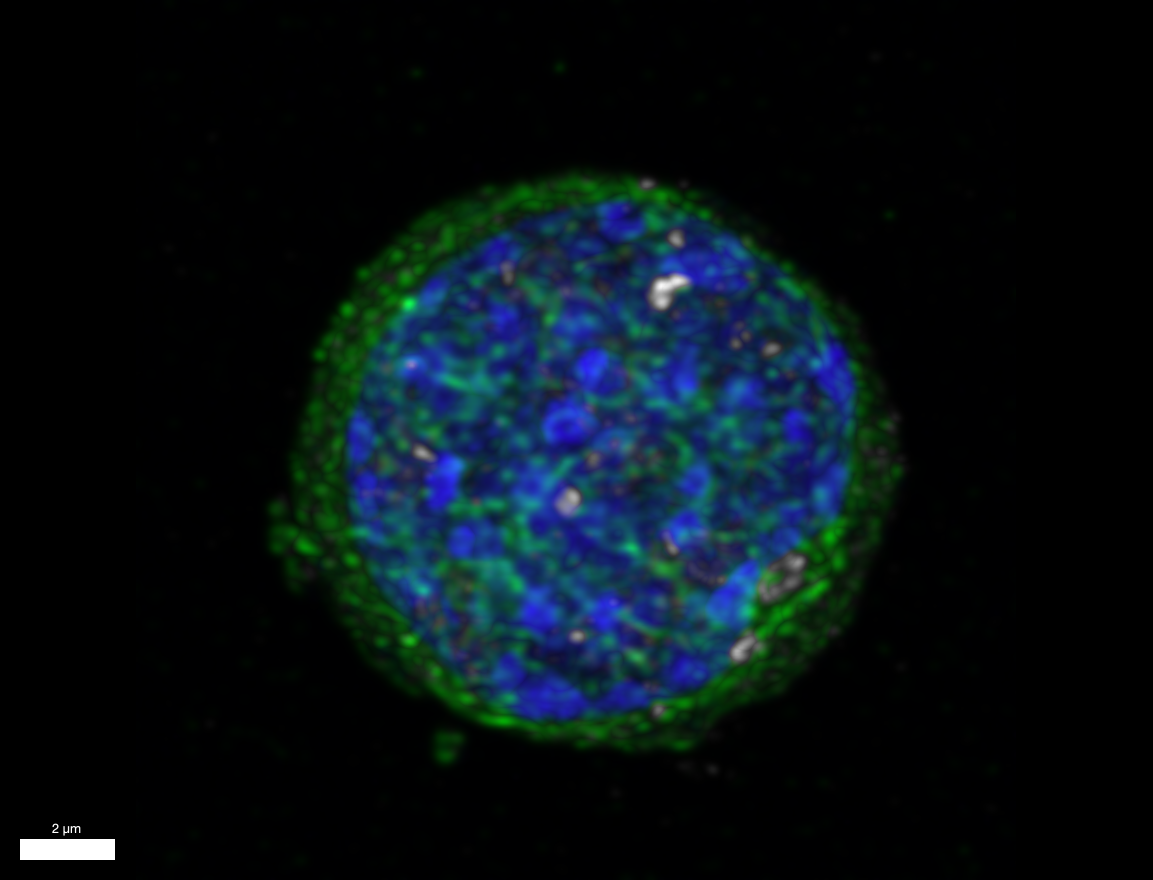

Supplement: Supplementary file 8 — Figure Source Data EV3 [file 44319_2024_159_MOESM8_ESM.zip › EMBOR-2023-58207V1_SourceDataForExpandedView_Figure EV3 /EV3C/EMBOR-2023-58207V1_SourceDataForFigEV3C_Tube1GCKO:GCKO_merge.tif]

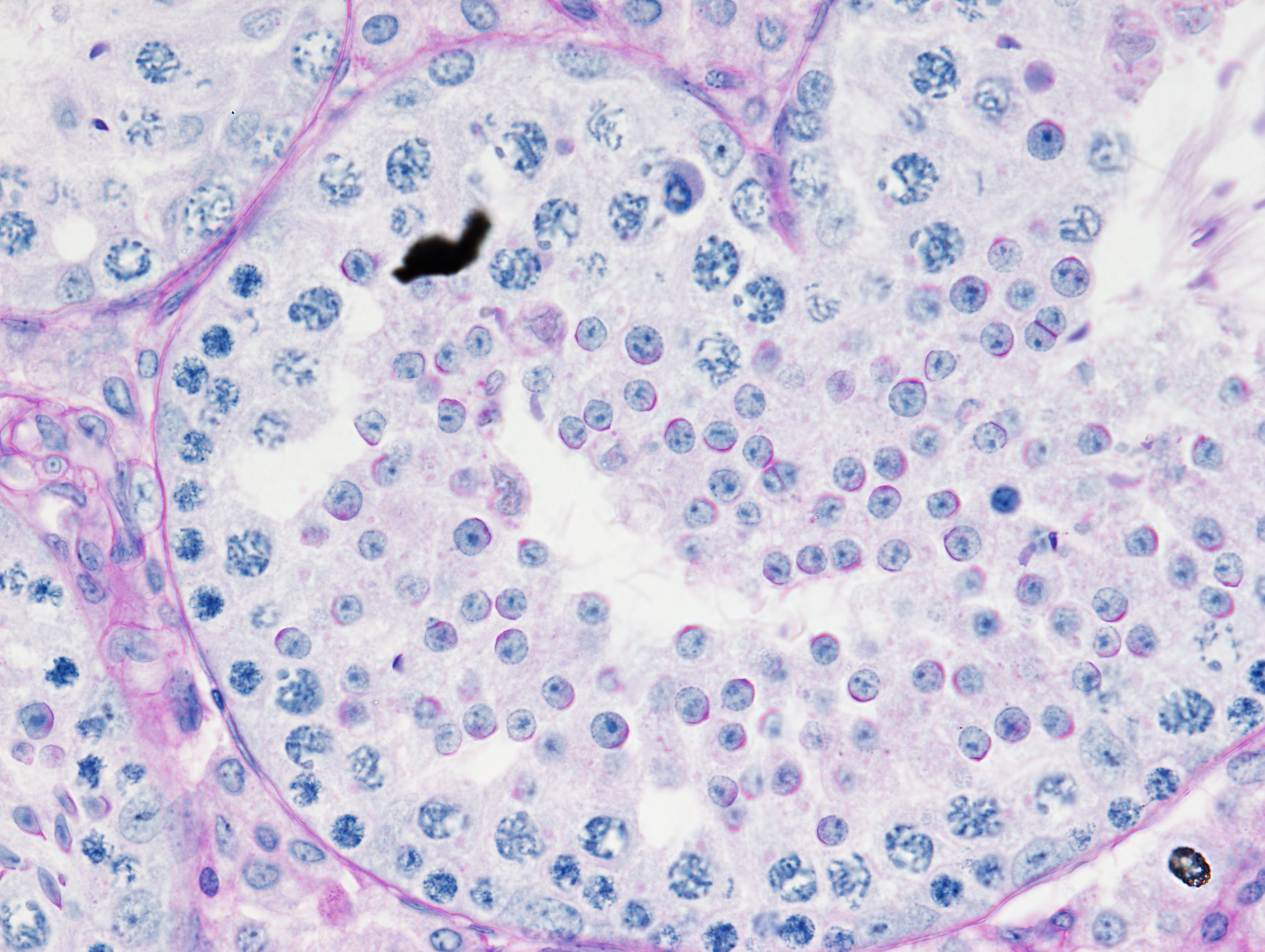

Supplement: Supplementary file 8 — Figure Source Data EV3 [file 44319_2024_159_MOESM8_ESM.zip › EMBOR-2023-58207V1_SourceDataForExpandedView_Figure EV3 /EV3B/EMBOR-2023-58207V1_SourceDataForFigEV3B_GCKO.tif]

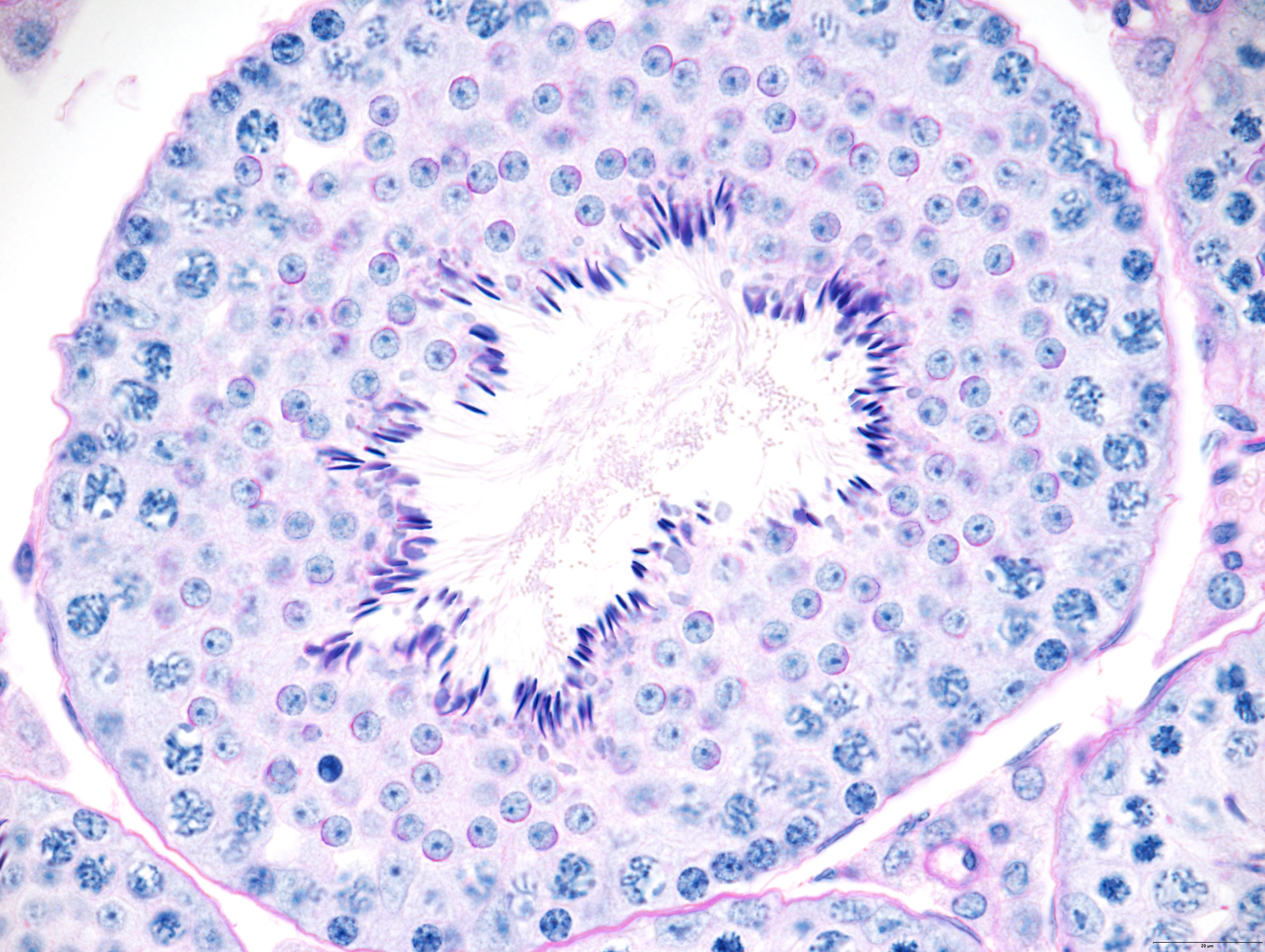

Supplement: Supplementary file 8 — Figure Source Data EV3 [file 44319_2024_159_MOESM8_ESM.zip › EMBOR-2023-58207V1_SourceDataForExpandedView_Figure EV3 /EV3B/EMBOR-2023-58207V1_SourceDataForFigEV3B_Flox.tif]

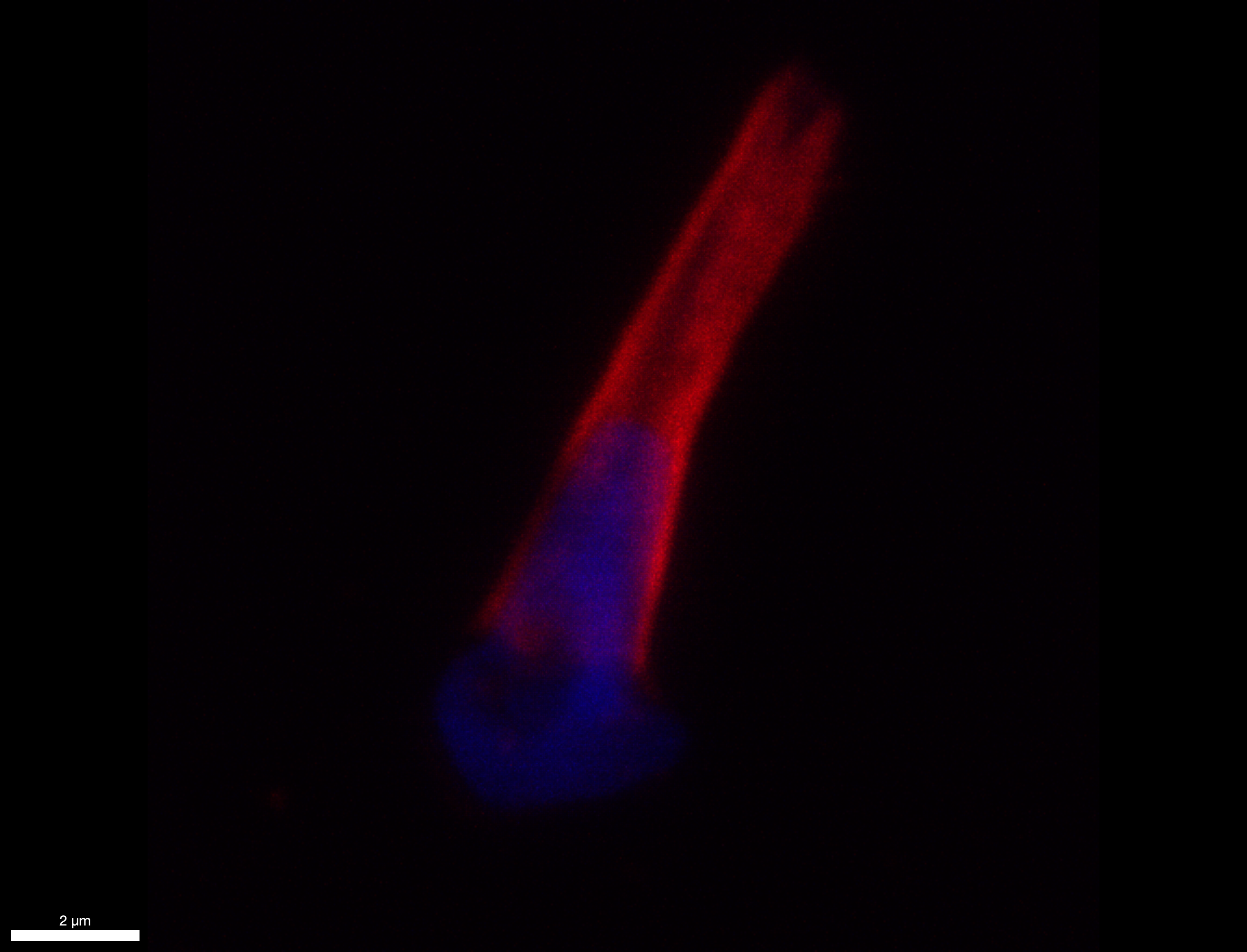

Supplement: Supplementary file 10 — Figure Source Data EV5 [file 44319_2024_159_MOESM10_ESM.zip › EMBOR-2023-58207V1_SourceDataForExpandedView_Figure EV5/EMBOR-2023-58207V1_SourceDataForFigEV5_Tube1GCKO:GCKO_merged.tif]

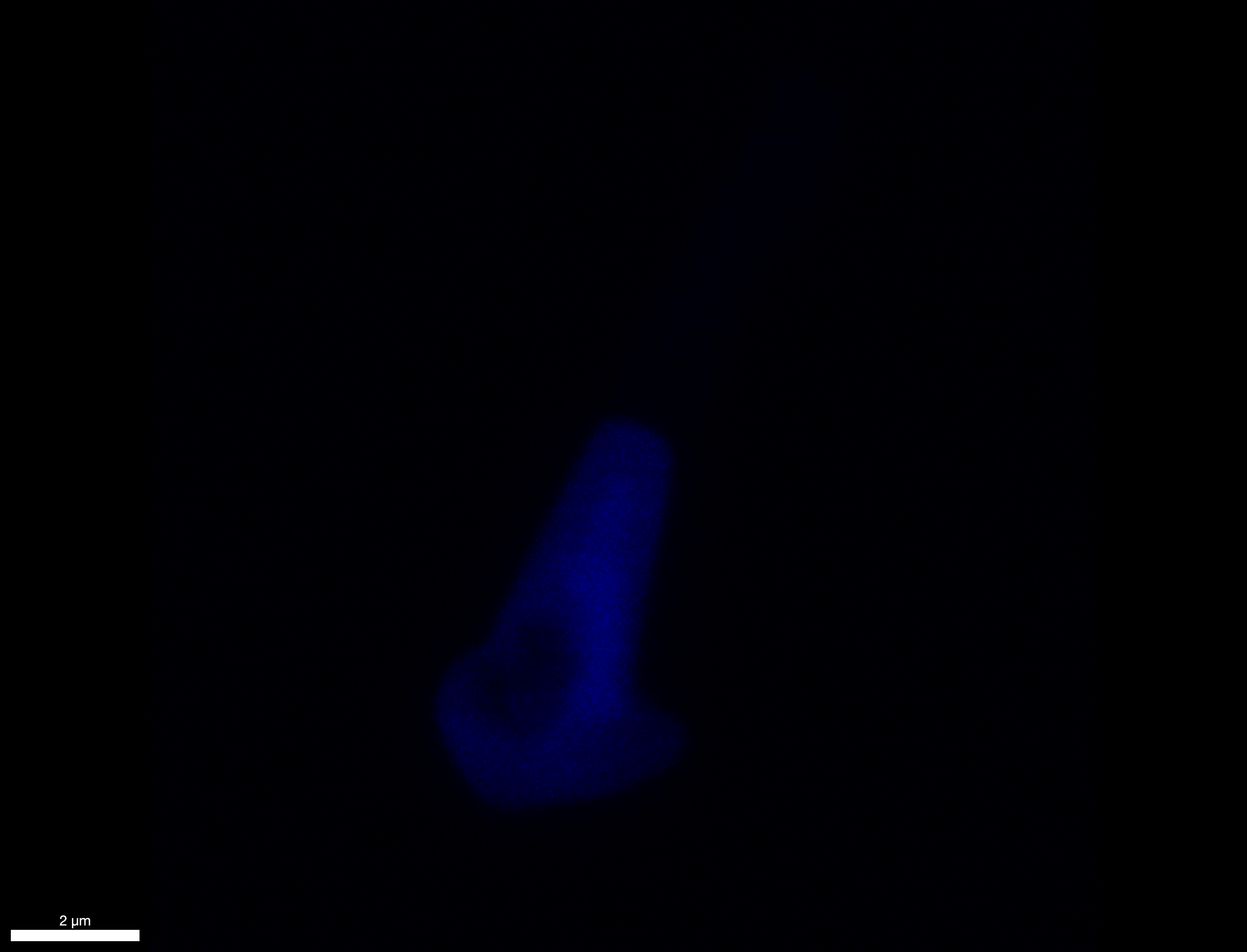

Supplement: Supplementary file 10 — Figure Source Data EV5 [file 44319_2024_159_MOESM10_ESM.zip › EMBOR-2023-58207V1_SourceDataForExpandedView_Figure EV5/EMBOR-2023-58207V1_SourceDataForFigEV5_Tube1GCKO:GCKO_DAPI.tif]

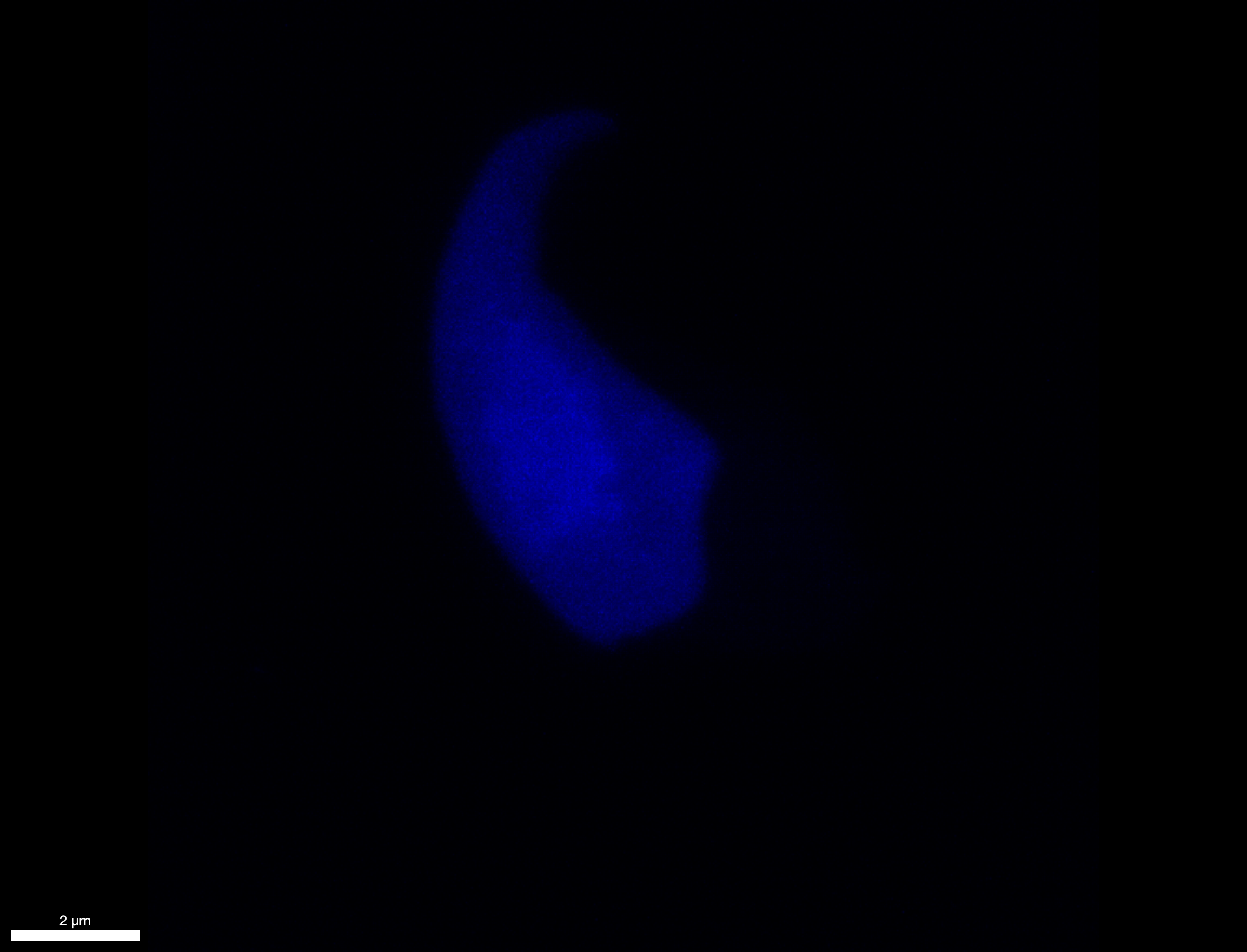

Supplement: Supplementary file 10 — Figure Source Data EV5 [file 44319_2024_159_MOESM10_ESM.zip › EMBOR-2023-58207V1_SourceDataForExpandedView_Figure EV5/EMBOR-2023-58207V1_SourceDataForFigEV5_Tube1Flox:Flox_DAPI.tif]

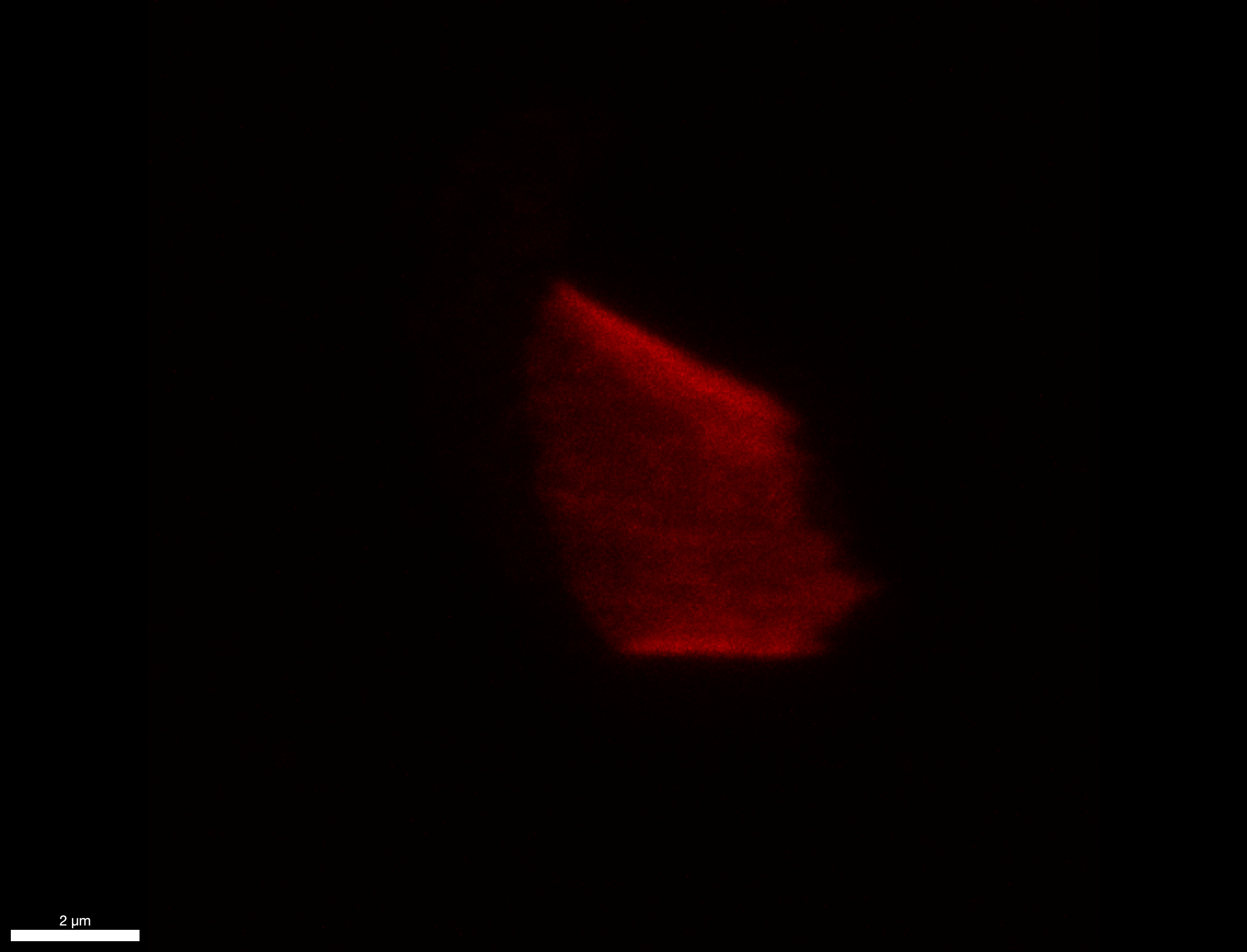

Supplement: Supplementary file 10 — Figure Source Data EV5 [file 44319_2024_159_MOESM10_ESM.zip › EMBOR-2023-58207V1_SourceDataForExpandedView_Figure EV5/EMBOR-2023-58207V1_SourceDataForFigEV5_Tube1Flox:Flox_acetylated tubulin.tif]

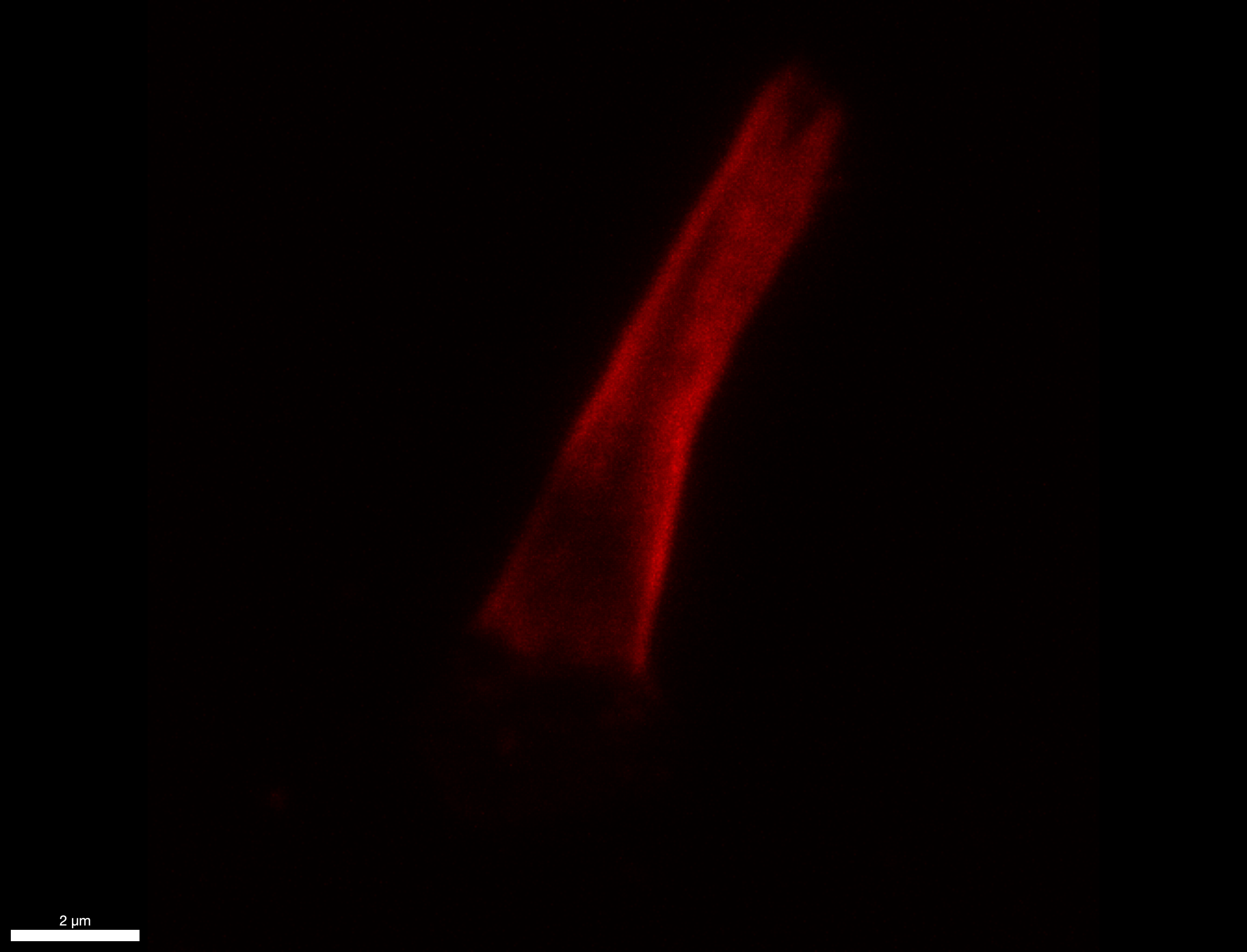

Supplement: Supplementary file 10 — Figure Source Data EV5 [file 44319_2024_159_MOESM10_ESM.zip › EMBOR-2023-58207V1_SourceDataForExpandedView_Figure EV5/EMBOR-2023-58207V1_SourceDataForFigEV5_Tube1GCKO:GCKO_acetylated tubulin.tif]

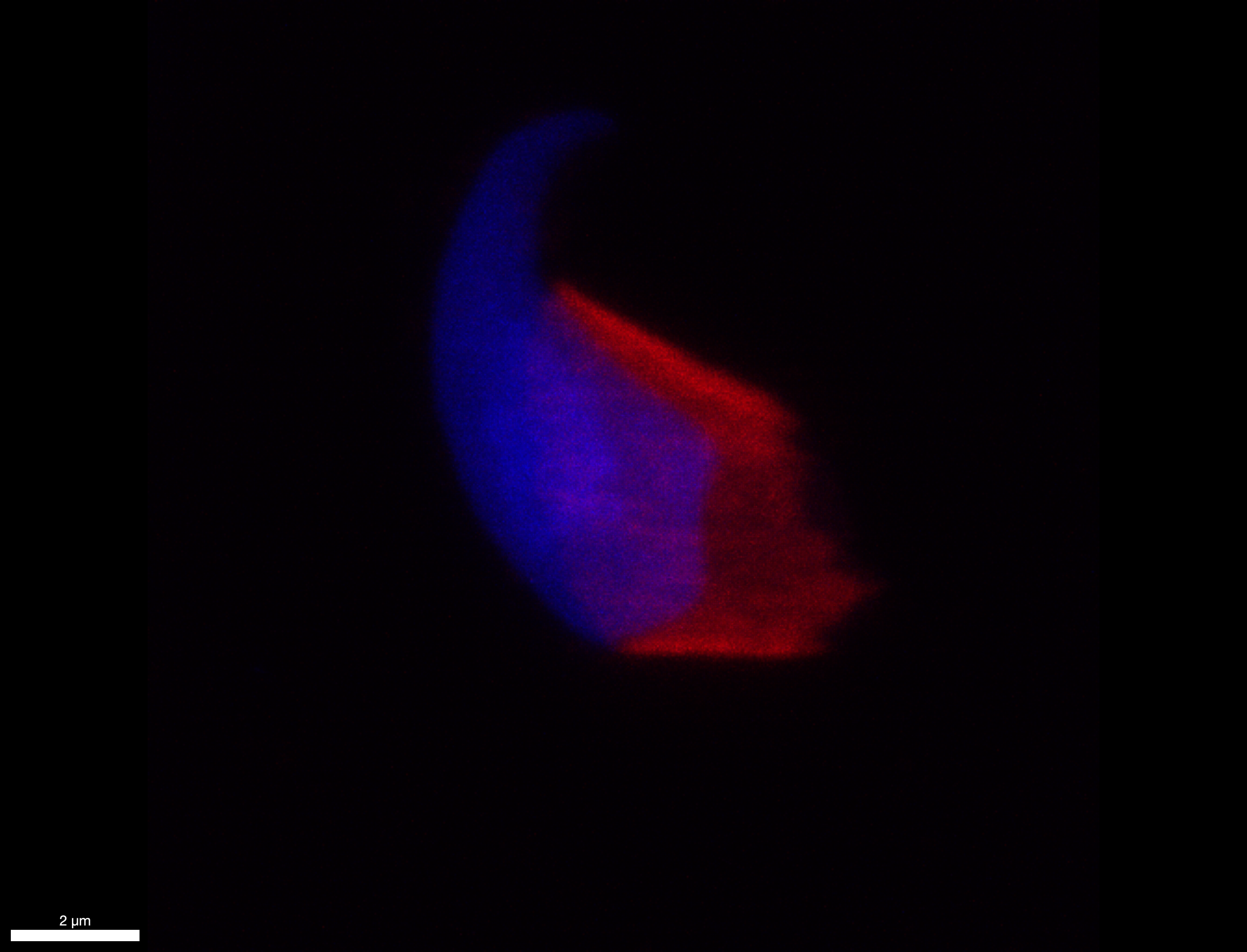

Supplement: Supplementary file 10 — Figure Source Data EV5 [file 44319_2024_159_MOESM10_ESM.zip › EMBOR-2023-58207V1_SourceDataForExpandedView_Figure EV5/EMBOR-2023-58207V1_SourceDataForFigEV5_Tube1Flox:Flox_merged.tif]
