# Supplementary material for: Epsilon tubulin is an essential determinant of microtubule-based structures in male germ cells
Source: EMBO Rep. 2024 May 21;25(6):14. doi: 10.1038/s44319-024-00159-w (PMC11169422; doi:10.1038/s44319-024-00159-w)
Supplement: Supplementary file 12 — Expanded View Figures [file 44319_2024_159_MOESM12_ESM.pdf]

# Expanded View Figures

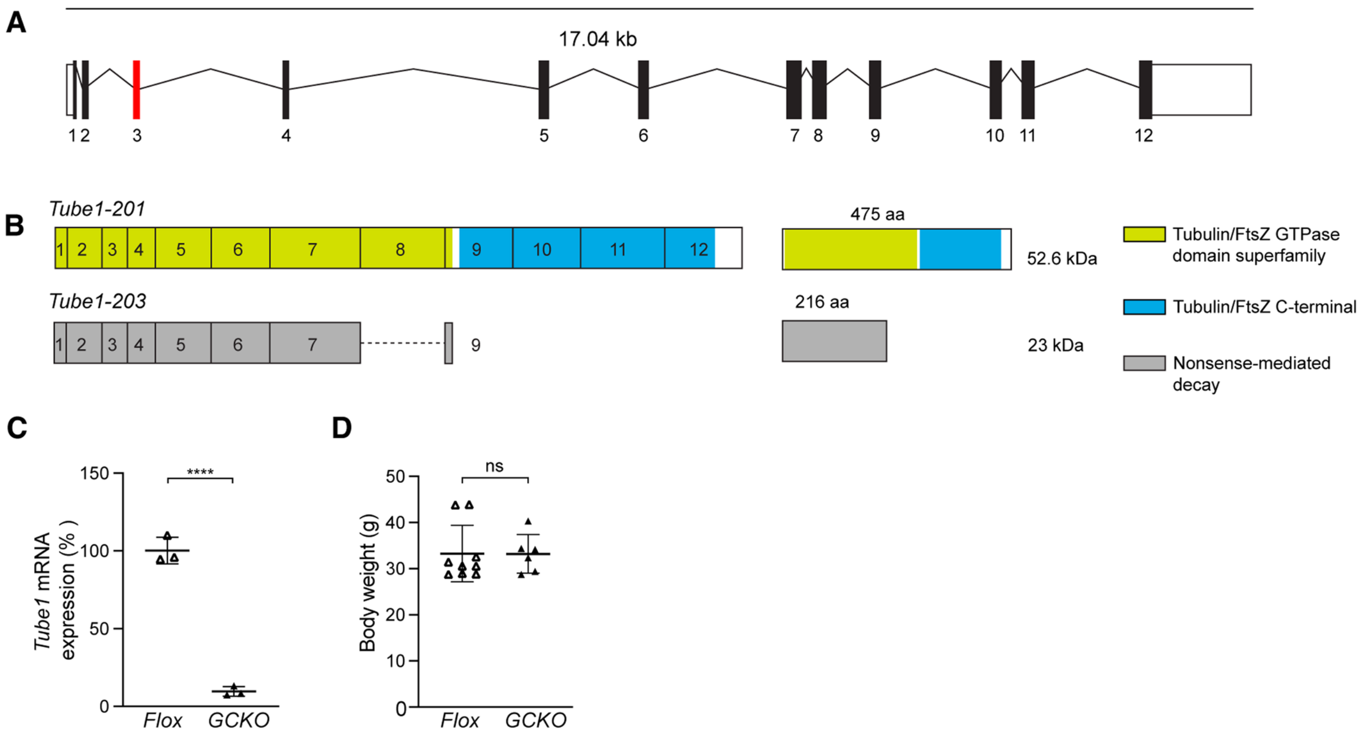


**Figure EV1. Creation of the *Tube1*$^{GCKO/GCKO}$ mouse model and *Tube1* expression.**

(**A**) The *Tube1* gene consists of 12 exons, with length shown in kilobases (kb). Exons are boxes and introns are lines. Exon 3 targeted for deletion in the male germline highlighted in red. (**B**) Processed transcripts of *Tube1* gene as named in Ensembl (ENSMUSG00000019845). *Tube1-201* is the only predicted protein coding transcript, whereas *Tube1-203* undergoes nonsense-mediated decay (Cunningham et al, 2021). (**C**) *Tube1* mRNA expression in STAPUT purified spermatocytes as percentage of control from *Tube1*$^{Flox/Flox}$ (*Flox*) and *Tube1*$^{GCKO/GCKO}$ (*GCKO*) mice, with *Ppia* as a reference gene ($n = 3$ animals/genotype, 3 technical replicates; unpaired t-test, $P < 0.0001$, ****). (**D**) Body weight comparison (grams, g) between *Tube1*$^{Flox/Flox}$ and *Tube1*$^{GCKO/GCKO}$ male mice ($n = 9$ *Flox* and $n = 6$ *GCKO* animals, 1 technical replicate; Mann–Whitney U test, P = ns (non-significant)). Data information: For each data panel, results are from one experiment. In (**C**, **D**), data are presented as mean ± SD). Source data are available online for this figure.

 © The Author(s)

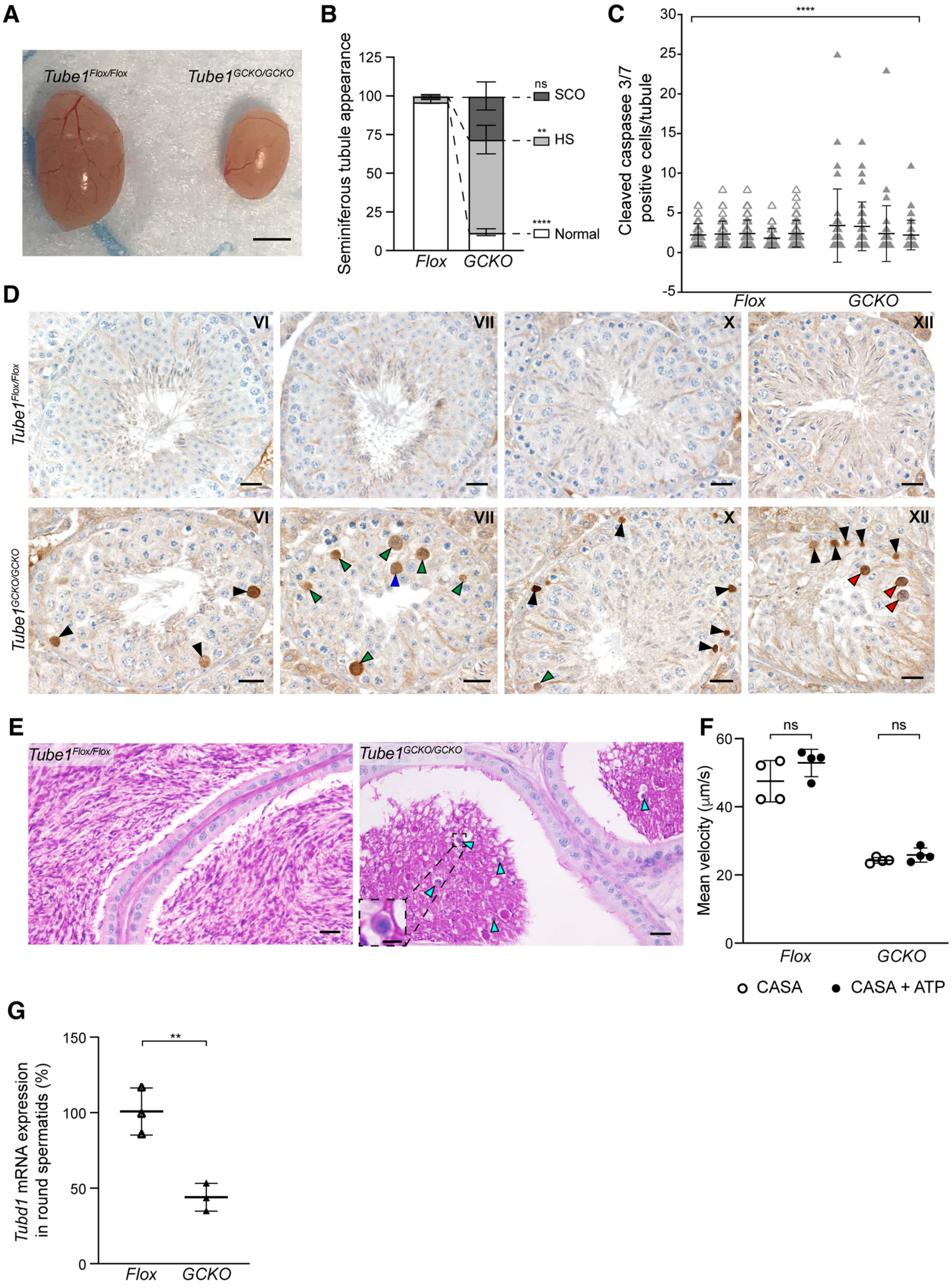
A
Tube1Flox/Flox
Tube1GCKO/GCKO
B
Seminiferous tubule appearance
125
100
75
50
25
0
Flox
GCKO
ns
SCO
**
HS
****
Normal
C
Cleaved caspasee 3/7 positive cells/tubule
30
25
20
15
10
5
0
-5
****
Flox
GCKO
D
Tube1Flox/Flox
Tube1GCKO/GCKO
VI
VII
X
XII
E
Tube1Flox/Flox
Tube1GCKO/GCKO
F
Mean velocity (μm/s)
60
40
20
0
ns
ns
Flox
GCKO
CASA
CASA + ATP
G
Tubd1 mRNA expression in round spermatids (%)
150
100
50
0
**
Flox
GCKO

◀ **Figure EV2. TUBE1 is required for spermatogenesis in the mouse.**

(**A**) Representative images of a testis from *Tube1*$^{Flox/Flox}$ and *Tube1*$^{GCKO/GCKO}$ mice. Scale bars: 2 mm. (**B**) Distribution of normal spermatogenesis, hypospermatogenesis (HS), or Sertoli cell only (SCO) tubules observed within seminiferous tubule cross sections from *Tube1*$^{Flox/Flox}$ (*Flox*) and *Tube1*$^{GCKO/GCKO}$ (*GCKO*) mice ($n = 3$ animals/genotype, 1 technical replicate; unpaired t-test(s), $P < 0.0001$ (Normal), ****, $P = 0.0080$ (Hypo, Welch's correction), **, and Mann–Whitney U test, $P$ = ns (non-significant; SCO)). (**C**) Average number of cleaved Caspase 3 and/or 7 positive cells per seminiferous tubule, per mouse from *Tube1*$^{Flox/Flox}$ (*Flox*) and *Tube1*$^{GCKO/GCKO}$ (*GCKO*) mice ($n = 5$ *Flox* and $n = 4$ *GCKO* animals, 1 technical replicate; generalized linear model with zero-inflated negative binomial distribution, $P < 0.0001$, ****). (**D**) Staged cross sections of seminiferous tubules stained for cleaved Caspase 3 and 7. Caspase-positive cells identified as spermatogonia (black arrowheads), pachytene spermatocytes (green arrowheads), round spermatid (blue arrowhead) and metaphase spermatocytes (red arrowheads) ($n = 5$ *Flox* and $n = 4$ *GCKO* animals). Scale bars: 20 µm. (**E**) PAS-stained epididymis sections illustrating germ cell sloughing in *Tube1*$^{GCKO/GCKO}$ mice (cyan arrowheads) ($n = 3$ animals/genotype). Scale bars: 20 µm; 5 µm inset. (**F**) Mean velocity (µm/s) of *Flox* and *GCKO* epididymal sperm measured using CASA, before (CASA) and after (CASA + ATP) the addition of 55 µg/ml ATP ($n = 3$ animals/genotype, 1 technical replicate; paired t-test(s), $P$ = ns (non-significant; *Flox*, *GCKO*)). (**G**) *Tubd1* mRNA expression in STAPUT purified round spermatids as percentage of control from *Tube1*$^{Flox/Flox}$ (*Flox*) and *Tube1*$^{GCKO/GCKO}$ (*GCKO*) mice, with *Hprt1* and *Ppia* as reference genes ($n = 3$ animals/genotype, 3 technical replicates; unpaired t-test, $P = 0.0055$, **). Data information: For each data panel, results are from one experiment. In (**B**, **C**, **F**, **G**), data are presented as mean ± SD). Scale bars: 20 µm; 5 µm inset. Source data are available online for this figure.

 © The Author(s)

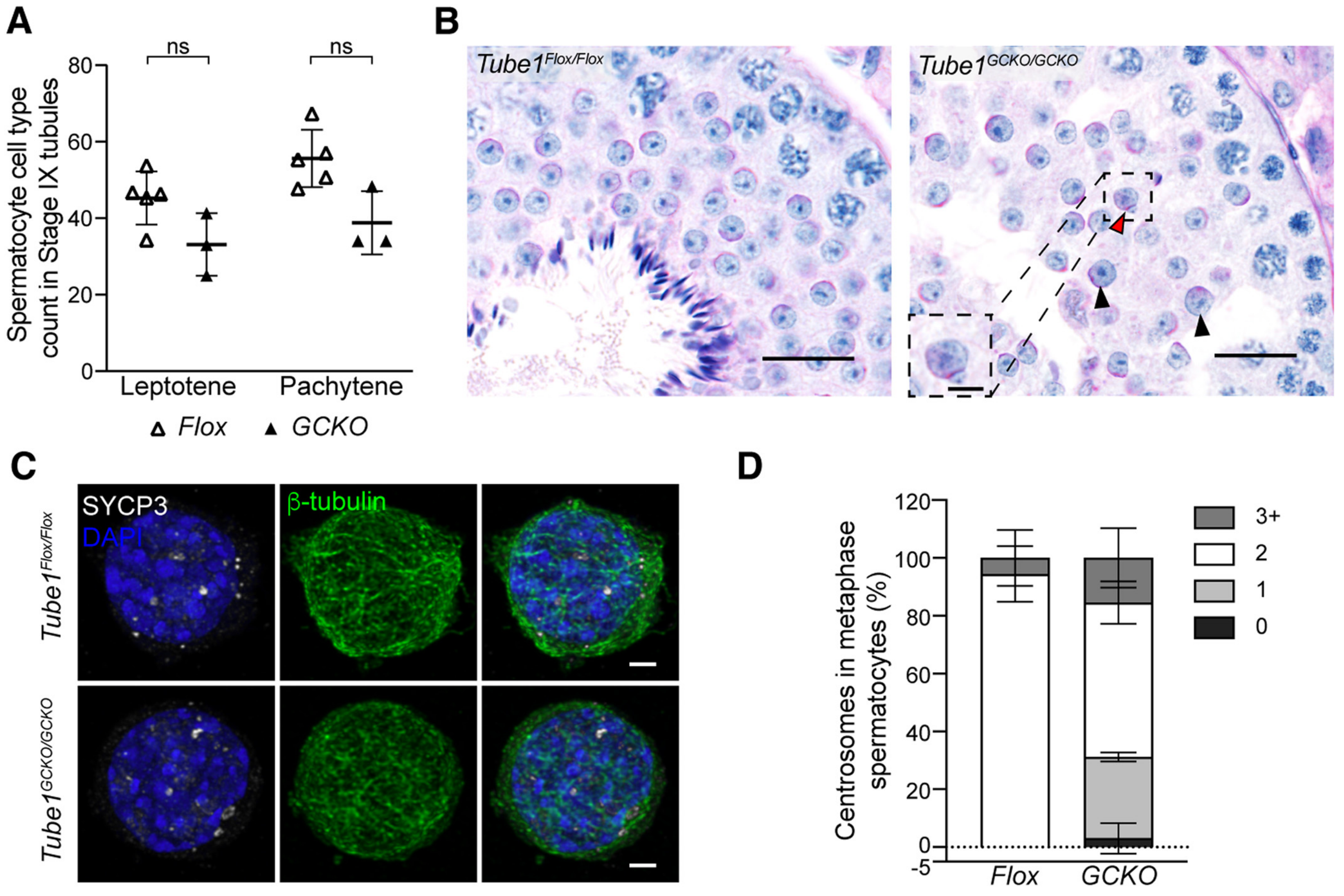


**Figure EV3. The beginning of meiosis prophase I is not affected by TUBE1 loss-of-function.**

(**A**) Average number of leptotene and pachytene spermatocytes in Stage IX seminiferous tubules in *Tube1*$^{Flox/Flox}$ (*Flox*) and *Tube1*$^{GCKO/GCKO}$ (*GCKO*) mice ($n = 5$ *Flox* and $n = 3$ *GCKO* animals, 3 seminiferous tubules examined per animal; unpaired t-test (leptotene), Mann-Whitney U test (pachytene), $P$ = ns (non-significant)). Data are presented as mean ± SD. (**B**) Cross section of seminiferous tubules with abnormally large round spermatid nuclei in *Tube1*$^{GCKO/GCKO}$ (black arrowheads) and a binucleated spermatid with one small nucleus and one large nucleus (red arrowheads) ($n = 3$ animals/genotype). Scale bars: 20 μm. (**C**) Isolated *Tube1*$^{Flox/Flox}$ and *Tube1*$^{GCKO/GCKO}$ leptotene spermatocytes immunolabeled to visualize synaptonemal complex protein 3 (SYCP3, white) and β-tubulin (green) and co-stained with DAPI to visualize DNA (blue). Images are deconvolved and represent 3D maximum intensity projections ($n = 3$ *Flox* and $n = 3$ *GCKO* animals). Scale bars: 2 μm. (**D**) Percentage distribution of centrosome number in metaphase spermatocytes from *Tube1*$^{Flox/Flox}$ and *Tube1*$^{GCKO/GCKO}$ mice ($n = 3$ animals/genotype, 6–9 *Tube1*$^{Flox/Flox}$ and 10–11 *Tube1*$^{GCKO/GCKO}$ cells analyzed/animal). Data are presented as mean ± SD. Source data are available online for this figure.

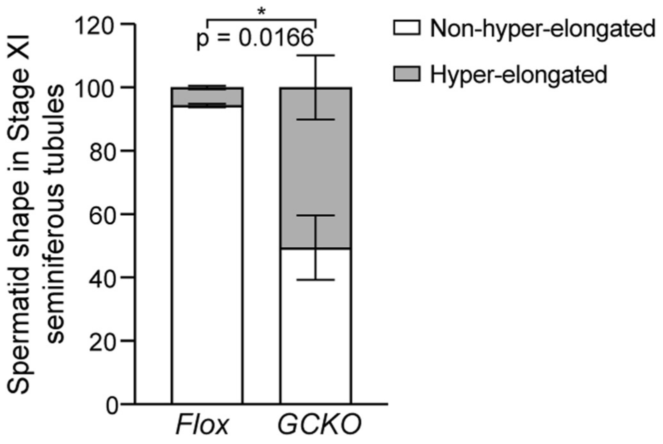


**Figure EV4. Increase in hyper-elongated spermatid nuclei in the absence of TUBE1.**

Distribution of non-hyper-elongated and hyper-elongated spermatid shapes observed within seminiferous tubule cross sections from *Tube1*$^{Flox/Flox}$ (*Flox*) and *Tube1*$^{GCKO/GCKO}$ (*GCKO*) mice ($n = 3$ animals/genotype, 1 technical replicate; unpaired t-test, $P = 0.0166$ (Welch's correction), *). Results are from one experiment. Source data are available online for this figure.

 © The Author(s)

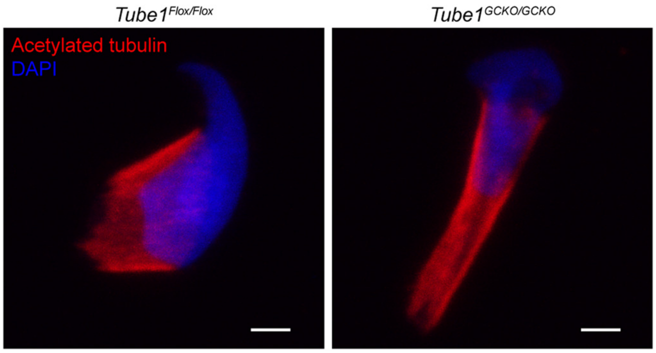


**Figure EV5. Manchette acetylated tubulin is not affected by the absence of TUBE1.**

Isolated elongating spermatids from *Tube1*$^{Flox/Flox}$ and *Tube1*$^{GCKO/GCKO}$ mice, immunolabeled for acetylated tubulin (red) and counterstained with DAPI (blue) ($n = 3$ animals/genotype). Scale bars: 2 μm. Results are from one experiment. Source data are available online for this figure.
